# Electronic structure analysis of electrochemical CO_2_ reduction by iron-porphyrins reveals basic requirements for design of catalysts bearing non-innocent ligands†

**DOI:** 10.1039/d2sc01863b

**Published:** 2022-06-29

**Authors:** Maxime Tarrago, Shengfa Ye, Frank Neese

**Affiliations:** Max-Planck-Institut für Kohlenforschung Kaiser-Wilhelm-Platz 1 D-45470 Mülheim an der Ruhr Germany shengfa.ye@dicp.ac.cn frank.neese@kofo.mpg.de; State Key Laboratory of Catalysis, Dalian Institute of Chemical Physics, Chinese Academy of Sciences Dalian 116023 China

## Abstract

Electrocatalytic CO_2_ reduction is a possible solution to the increasing CO_2_ concentration in the earth’s atmosphere, because it enables storage of energy while using the harmful CO_2_ feedstock as a starting material. Notably, iron(ii) tetraphenylporphyrin, [Fe^II^(TPP)]^0^ (TPP^2−^ = tetraphenylporphyrin tetra-anion diradical), and its derivatives have been established as one of the most promising families of homogeneous catalysts for CO_2_ reduction into CO. Our earlier work has demonstrated that [Fe(TPP)]^2−^, a catalytically active species, is best described as an Fe(ii) center antiferromagnetically coupled with a TPP^4−^ diradical. In fact, [Fe(TPP)]^2−^ represents a prototypical example of a diverse array of highly efficient molecular catalysts that feature non-innocent ligands. To obtain valuable insights for future catalyst design, their outstanding catalytic performance warrants an investigation aimed at elucidating the role played by the ligand non-innocence in the reaction. To this end, the reactivity of [Fe(TPP)]^2−^ was first investigated in detail by using density functional theory calculations, and the theoretical results were then validated by reproducing available experimental kinetic and thermodynamic data. Further in-depth analyses pinpointed the electronic-structure feature of the non-innocent TPP ligand that is responsible for the high efficiency of the reaction. Finally, we analyzed the electronic-structure evolution found for the reactions catalyzed by ten related representative non-innocent systems. Our results revealed that for the reactions under consideration, the reducing equivalents are stored on the non-innocent ligand, while CO_2_ functionalization takes place at the metal center. Therefore, all of the transformations invariably entail two synchronized electron-transfer events: (1) a metal-to-CO_2_ transfer and (2) a ligand-to-metal electron transfer. The former is affected by σ-donation from the metal d_*z*^2^_ orbital to the CO_2_
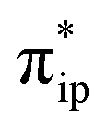
 orbital, and the latter is facilitated by orbital coupling between the ligand and the metal center. Our results suggested that ligand non-innocence plays a fundamental role in stabilizing highly active intermediates while realizing high product selectivity for CO_2_ reduction and that the metal–ligand cooperativity is essential to the high reaction kinetics. On the basis of these findings, we proposed fundamental requirements for design of catalysts with non-innocent ligands.

## Abstract 2

This computational study elucidates the role played by ligand non-innocence in CO_2_ reduction on the basis of analyses of electronic structure evolution of highly efficient iron-tetraphenylporphyrin and ten other transition metal complexes, all bearing non-innocent ligands.

## Graphical abstract

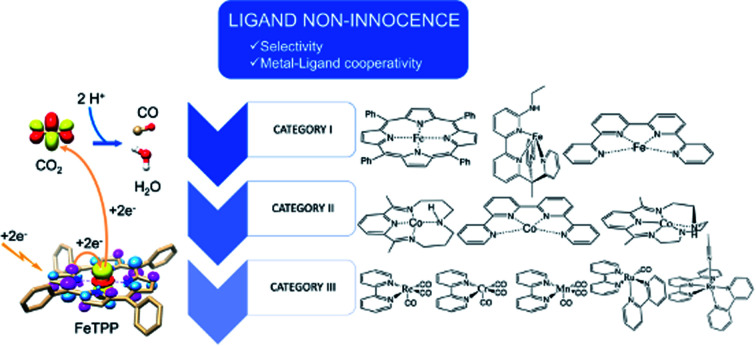

## Introduction

Over the course of the last 30 years, the mean atmospheric CO_2_ concentration has increased by 20% and reached a new record of 411 ppm in 2020.^1^ On one hand, CO_2_ is a major greenhouse gas and largely contributes to global warming. On the other hand, CO_2_ is a ubiquitous C1 feedstock that can be used to produce value-added chemicals and biofuels, thereby closing the hydrocarbon cycle.^2^ However, due to its high thermodynamic stability and kinetic inertness, CO_2_ functionalization typically requires not only an external energy input, but also, more importantly, appropriate catalysts.

**Scheme 1:**
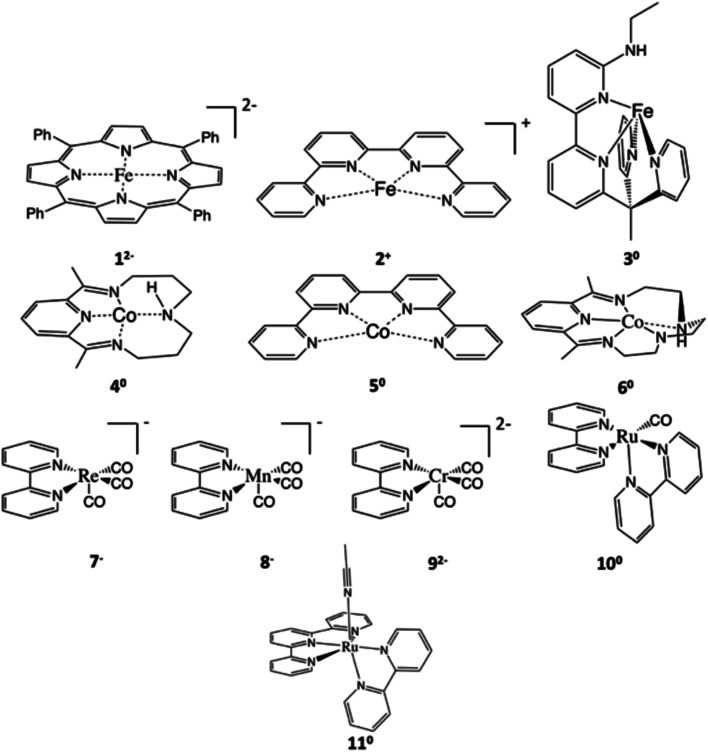
Representative examples of CO_2_ reduction catalysts studied in this work. Systems supported by non-innocent ligands include 1^2−^,8,16 [M(qpy)]^*n*+^ (M = Fe (2^+^), Co (5^0^), qpy = quaterpyridine),^12^ [Fe(bpy^NHEt^PY2Me)]^0^ (3^0^, bpy^NHEt^PY2Me = 6′-(1,1-di(pyridin-2-yl)ethyl)-*N*-ethyl-[2,2′-bipyridin]-6-amine),^19^ [Co(N_4_H)]^0^ (4^0^, N_4_H = 2,12-dimethyl-3,7,11,17-tetraazabicyclo[11.3.1]-heptadeca-1(7),2,11,13,15-pentane),^14^ [Co(L)]^0^ (6^0^, L = 2,13-dimethyl-3,6,9,12,18-pentaazabicyclo-[12.3.1]octadeca-1(18),2,12,14,16-pentaene),^13^ [M(bpy)(CO)_3_]^*n*−^ (M = Re (7^−^), Mn (8^−^), Cr (9^2−^); bpy = bipyridine),^15,26,27^ [Ru(bpy)_2_(CO)]^0^ (10^0^),^28^ and [Ru(tpy)(bpy)]^0^ (11^0^, tpy = terpyridine).^20^ All species are labelled as Y*^z^*, where *z* corresponds to the charge of the active species prior to CO_2_ binding in the catalytic cycle.

Recently, tremendous effort has been devoted to developing efficient catalysts for photo-^3^ and electro-chemical^4^ CO_2_ reduction generating CO, formic acid, oxalate, methanol and other hydrocarbon compounds.^2*b*,5^ In particular, recent research effort has been directed towards catalysts containing earth-abundant base metals for economic reasons. Besides heterogeneous catalysts, homogeneous molecular systems also attract much attention,^6^ because such systems often can provide considerable mechanistic insights on reactions. Typically, mechanistic understanding can be used to direct systematic tuning of available systems and, more importantly, rational design of new catalysts with higher efficiency and product selectivity. To date, a plethora of homogeneous catalysts have been reported in the literature. Among them, [Fe(TPP)] (1, TPP^2−^ = tetraphenylporphyrinate dianion, Scheme 1) and its derivatives exhibit one of the highest catalytic performances in dimethylformamide (DMF), *i.e.* relatively low overpotentials (−1.64 V *vs.* the standard calomel electrode, SCE, for [Fe(TPP)]), high turnover frequencies and a faradaic efficiency for CO generation close to 100% in the presence of sufficiently weak acid.^7,8^

**Scheme 2:**
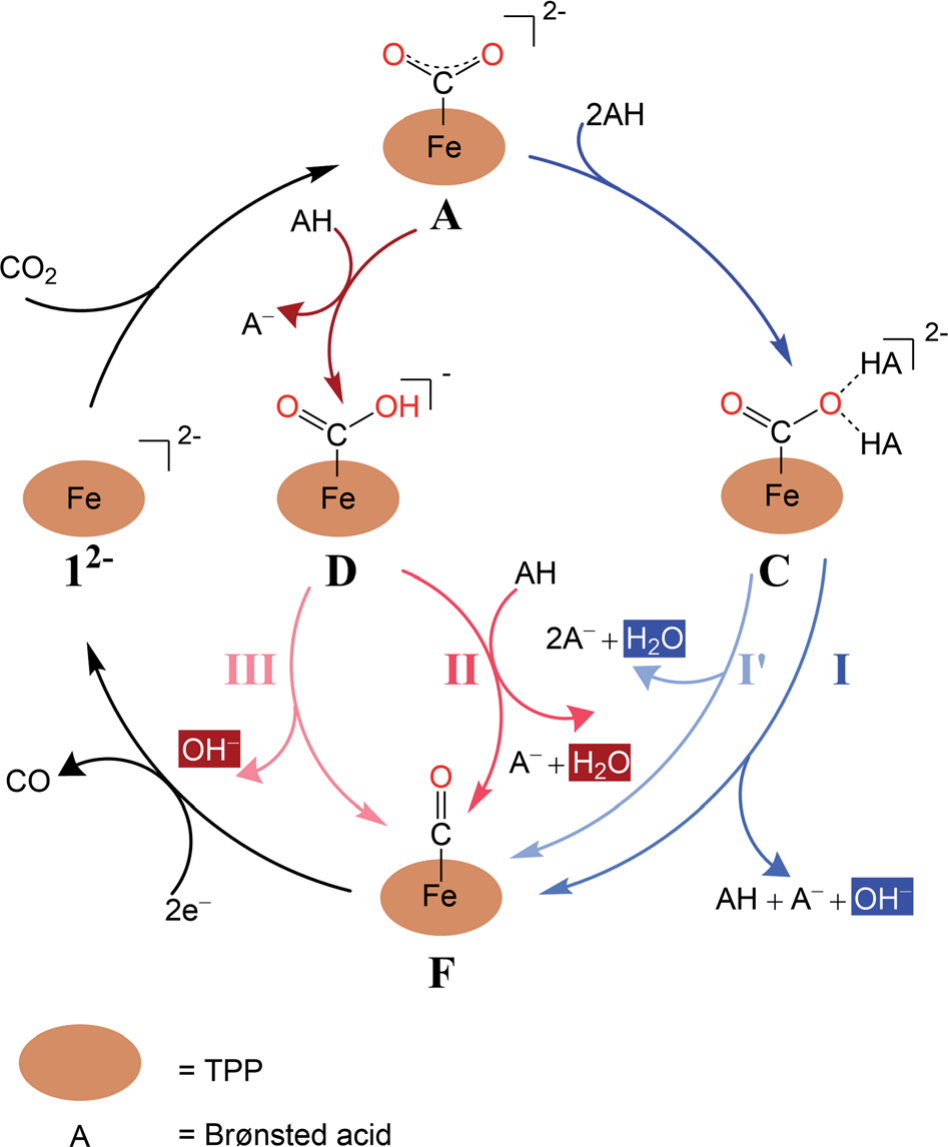
Four mechanisms of CO_2_-to-CO conversion catalyzed by Fe(TPP) investigated in the present work.

Experimental findings suggested that the active species responsible for CO_2_ conversion is [Fe(TPP)]^2−^ (1^2−^), a formal Fe^0^ complex, generated by two-electron reduction of 1.^8^ It is generally accepted that the reaction is initiated by CO_2_ binding to 1^2−^ to yield an η^1^-CO_2_ adduct (Scheme 2); however, the following transformation is rather controversial. An earlier experimental study^8*b*^ reported by Costentin and coworkers suggested that formation of two hydrogen bonds between the leaving O atom of the CO_2_ complex and two Brønsted acids activates the C–O bond being cleaved (pathway I in Scheme 2). Subsequently, the C–O bond scission is accompanied by a single proton transfer, thereby yielding an Fe^II^-carbonyl species and releasing a hydroxide anion. Although this mechanistic hypothesis likely accounted for the influence of the p*K*_a_ value of the Brønsted acid on the reaction rate,^8*b*^ the postulated tri-molecular reaction is expected to suffer from a prohibitively large positive entropic term.^9^ Theoretical calculations^10^ instead pointed out that the η^1^-CO_2_ adduct first gets protonated to afford a metallacarboxylic acid, which then undergoes C–O bond breaking concerted with second protonation, ultimately furnishing an Fe^II^-carbonyl adduct and H_2_O (pathway B in Scheme 2). Similar mechanisms have been proposed in the literature for CO_2_ reduction mediated by related systems.^11–15^

To understand reaction mechanisms, thoroughly elucidating the electronic structures of key intermediates is typically a prerequisite. Our earlier work using a combined spectroscopic and computational approach unequivocally revealed that 1^2−^ in fact contains an intermediate spin ferrous center (*S*_Fe_ = 1) that is antiferromagnetically coupled with a TPP^4−^ diradical (*S*_TPP_ = 1), thereby yielding an overall singlet ground state, *viz.* [Fe^II^(TPP˙˙^4−^)]^2−^.^16^ As such, the two electrons used to reduce CO_2_ are stored in the non-innocent TPP ligand rather than the Fe center. However, the earlier mechanistic investigations^10^ did not clarify the role of TPP in the CO_2_ transformation by 1^2−^. Furthermore, a range of homogeneous CO_2_ activation catalysts reported thus far are supported by well-known redox active non-innocent ligands, such as porphyrins,^8^ corroles,^17^ pyridine-diimines^13,14,18^ polypyridines,^4*b*,15,19,20^ and N-heterocyclic carbenes^21^ to name a few. The reactions with such a diverse array of systems thus give rise to an intriguing question about how the non-innocent ligand interacts with the metal center to trigger the two-electron CO_2_ reduction to CO, because apparently not all coordination-unsaturated transition metal complexes bearing non-innocent ligands are capable of activating CO_2_. In fact, although ligand non-innocence has been intensely discussed in general catalysis,^22–25^ its implication with respect to CO_2_ functionalization has received relatively little attention.

The present work serves a dual purpose. We first present a detailed analysis of the electronic-structure evolution in the course of CO_2_-to-CO conversion catalyzed by 1^2−^, which shows that the non-innocent nature of TPP is at the core of the high catalytic activity. Then, we analyze the electronic structure changes of the same reaction mediated by ten related catalysts, which also exhibit excellent catalytic performances (Table S1†). They include eight metal–polypyridine systems and two metal–pyridine-diimine derivatives, all containing well-known non-innocent ligands (Scheme 1). Comparison of all systems under consideration enables us to identify the crucial role of non-innocent ligands in CO_2_ activation. On the basis of this, we propose fundamental electronic structure requirements for CO_2_ reduction with non-innocent systems, which can be used as a guideline for future design of similar catalytic systems.

## Computational details

All calculations were performed using the ORCA 4.2 program package.^29^ For geometry optimizations and frequency calculations, the hybrid B3LYP density functional^30^ was used in combination with the Def2-TZVP basis set for iron and the first coordination sphere, and the Def2-SVP basis set for all remaining atoms,^31^ referred to as the B3LYP/Def2-TZVP/Def2-SVP level of theory hereafter. Tight geometry convergence settings and default SCF convergence settings were used for all geometry optimizations. The final electronic energies were computed with the B3LYP functional in combination with the Def2-TZVPP basis set for all atoms (referred to as the B3LYP/Def2-TZVPP level of theory hereafter). Default SCF convergence settings were employed. To account for solvation effects and non-covalent dispersion interactions, the solvation model C-PCM^32^ for DMF and the Grimme’s D3BJ dispersion corrections were employed, respectively, for all calculations.^33,34^ Grid level 5 was used for all the calculations. All calculations were accelerated by using the RIJCOSX approximation.^35^

The initial guesses of transition state geometries were obtained at the B3LYP/Def2-TZVP/Def2-SVP level of theory by running relaxed surface scans in the sensible normal modes and/or by calculating using the nudged elastic band method,^36^ particularly for delicate transition states. The approximate transition state geometries were then optimized by maximizing the energy in a given normal mode and minimizing the energy in all other normal modes. Subsequent frequency analyses showed that local minima have no imaginary frequency, and transition states have only one imaginary frequency, occasionally besides an additional imaginary frequency of less than 16 cm^−1^ (see the ESI†). Despite our repeated attempts, this residual frequency could not be removed but is attributed to numerical noise owing to its magnitude.

Enthalpies were calculated by adding the zero-point energy (*E*_ZPE_) and the thermal energy at 298 K (*E*_th_) calculated at the aforementioned level of theory to the electronic energy (*E*_el_) calculated at the B3LYP/Def2-TZVPP level of theory. A *k*_B_*T* term was also added to account for the PV term in an ideal gas approximation. To estimate more accurate energies of intermediates featuring antiferromagnetic spin coupling between the ligand and the metal center, an electronic energy correction (Δ*E*_el_) calculated by using the method proposed by Malrieu and Trinquier^37^ was added to the energy of the broken-symmetry solution (eqn (1a)). Free energies at 298 K were calculated by adding the electronic, translational, vibrational and rotational entropy contributions multiplied by the temperature to the enthalpy.^38^ In the case of bimolecular reactions in the gas phase, translational entropic contributions typically account for +10 to +15 kcal mol^−1^ to Gibbs free energy changes,^9^ as suggested by our earlier work on O_2_ and CO_2_ association with transition metal centers.^39*d*,40^ Apparently, the gas-phase approximation overestimates the condensed-phase free energy significantly for two reasons. First, translational freedom is largely quenched in the condensed phase, because the volume is occupied by the solvent.^41^ Consequently, the translation entropy, which is directly dependent on the volume accessible to the solute, is affected by the passage from the gas to condensed phase. The subsequent loss of entropy can be estimated by calculating the loss of accessible volume (Δ*S*_AV_).^41^ Second, gas-phase free energy does not account for the cavitation free energy (Δ*G*_cav-disp_). The latter corresponds to the free energy affording the formation of the solvent cage around the solutes. Typically, both effects induce negative Gibbs free energy variations for bimolecular reactions, which partially compensate the large entropic gains calculated in the gas phase. To account for them, we added two correction terms −*T*Δ*S*_AV_ and Δ*G*_cav-disp_ to the free energy computed in the gas phase (eqn (1b)). The former was calculated following a procedure described elsewhere with the van der Waals radii of the solutes and solvent.^41^ The latter was estimated using a linear regression of the cavity surface using the van der Waals radii of the solute.^42^

1a *H* = *E*_el_ + Δ*E*_el_ + *E*_ZPE_ + *E*_th_ + *k*_B_*T*

1b *G* = *H* − *S*_g_*T* − *T*Δ*S*_AV_ + Δ*G*_cav-disp_

All redox potentials against the SCE electrode in DMF were calculated from the Gibbs free energies of the species of the redox couple, according to the formula:2
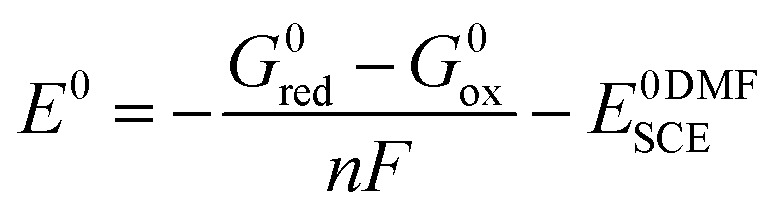
where *n* is the number of electrons in the reduction, *F* the Faraday constant (*F* = 23.061 kcal V^−1^ mol^−1^),^43^ and *E*^0DMF^_SCE_, the absolute potential of the SCE electrode in DMF, is taken as 4.350 V.^44^ The calculated redox potentials associated with the couples ^3^1^0^/^2^1^−^ and ^2^1^−^/^1^1^2−^ (where the left and right superscripts denote the multiplicity and charge, respectively) are −1.17 and −1.76 V, respectively. These values are in reasonable agreement with the experimental values of −1.07 and −1.64 V *vs.* SCE.^7,45–47^ These results thus lend credence to the reliability of our present computational setup.

For orbital visualization analysis, the unrestricted corresponding orbitals (UCO)^48^ with an overlap between the alpha and beta ones greater than 0.95 were localized using the Pipek–Mezey algorithm.^49^ The alpha and beta sets in this subspace were approximated to be identical, and the orbitals thus obtained from the localized subspace were considered as doubly occupied. The singly-occupied UCO and the magnetic orbitals (overlap ranging from 0 to 0.95) were not localized. Among the resulting orbitals, the d orbitals were identified by their predominant Fe character (>70%) according to the molecular orbital (MO) Löwdin population analysis.

## Results and discussion

### Potential energy surfaces

**Fig. 1:**
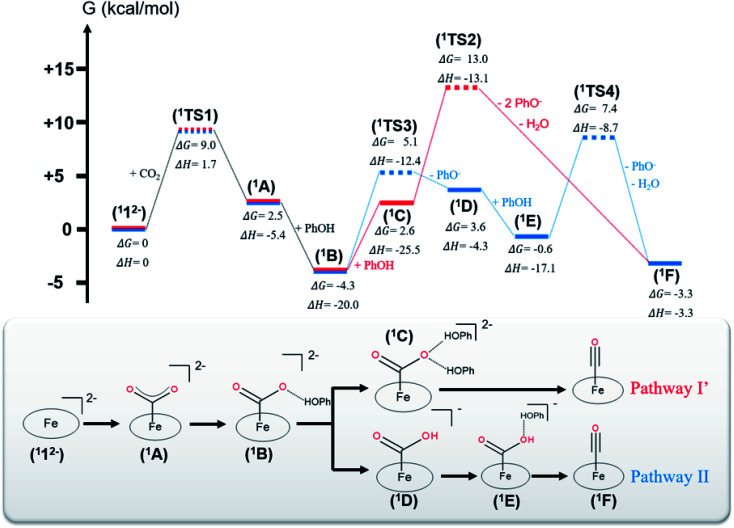
(Top) Energy landscape of the intermediates and transition states involved in the CO_2_-to-CO reduction. Intermediates are displayed in solid lines, and transition states are displayed in dashed lines. Intermediates belonging to pathway I′ are represented by red lines and those belonging to pathway II by blue lines. The Gibbs free energy changes and enthalpy changes were computed with respect to intermediate ^1^1^2−^ and all reactants infinitely separated in DMF. (Bottom) Schematic representations of the intermediates in the order of appearance in pathways I′ (red) and II (blue). Hydrogen bonds are displayed by dashed lines.

Experimental evidence points out that CO_2_ reduction to CO mediated by 1^2−^ is initiated by CO_2_ association with catalytically active 1^2−^, leading to an η^1^-CO_2_ adduct (A in Fig. 1). Accordingly, in the following section, only pathways involving 1^2−^ as the catalytically active species are considered.

The CO_2_ adduct A then undergoes proton-assisted C–O bond cleavage to produce CO and H_2_O in acidic media. As discussed above, the mechanistic details of the C–O bond breaking have not reached a consensus yet.^8*b*,10^ To address this question, we initially tested the mechanistic proposition due to Costentin and coworkers, pathway I schematized in Scheme 2.^8*b*^ In this mechanistic scenario, the interactions of the leaving O atom of A with two phenol (PhOH) molecules (the proton donor employed in the experiments^8*b*^) furnish complex C that features two hydrogen bonds between the CO_2_ moiety and PhOH. The following C–O bond cleavage is concomitant with the transfer of *only* one proton, thereby generating an OH^−^ and the product metal-carbonyl complex [Fe(CO)(TPP)] (F) in the end. The relaxed surface scans (Fig. S2†) in which the C–O distance was systematically varied showed that such a process cannot occur for reasons discussed below. We thus envisioned two more feasible mechanistic scenarios referred to as pathways I′ and II in Fig. 1. In pathway I′, the C–O bond rupture is realized by simultaneous *two* proton transfer from each PhOH to CO_2_ in C, leading to F and H_2_O. In pathway II, A first reacts with one PhOH molecule (rather than two in pathway I′) to form complex B with only one hydrogen bond. After releasing a phenoxide, B transforms into metallacarboxylic acid [Fe(COOH)(TPP)]^−^ (D). Subsequently, D forms a hydrogen bond of its COOH group with an incoming PhOH to convert into complex E, and this intermediate undergoes C–O bond scission assisted by a second proton transfer from PhOH, thereby resulting in F and H_2_O.

Although 1^2−^ was irrefutably identified to feature a singlet ground state,^16^ we investigated the reaction mechanisms on singlet, triplet and quintet potential energy surfaces in order to explore the possibility of the multi-state reactivity.^8*g*^ However, our theoretical results predicted that all aforementioned intermediates in the *S* = 1 and 2 states lie at substantially higher energies than the corresponding diamagnetic ones (Table S2†). Hence, they are very unlikely to participate in the actual reaction (for details, see the ESI†). Therefore, in the present section, we focus on the singlet reaction mechanism. To avoid confusion, hereafter we add a superscript to each species to denote its spin multiplicity.

The two steps leading to the formation of ^1^B are the same for both pathways I′ and II. For the CO_2_ adduct ^1^A, we tested two different CO_2_ binding modes with the Fe center, namely, η^1^-CO_2_ and η^1^-OCO. However, during the geometry optimizations, the η^1^-OCO complex spontaneously decomposes into ^1^1^2−^ and CO_2_. Therefore, the η^1^-CO_2_ adduct is the most appropriate model for ^1^A. DFT calculations predicted that formation of ^1^A traverses a transition state (^1^TS1) with a moderate barrier of Δ*G*^‡^ = +9.0 kcal mol^−1^ and is slightly endergonic with Δ*G* = 2.5 kcal mol^−1^. This positive Δ*G* value originates from the unfavorable entropic term and the limited exothermicity of the CO_2_ association (Δ*H* = −5.4 kcal mol^−1^). The latter finding can be readily traced back to the fact that typically CO_2_ is a weak ligand and the metal–CO_2_ interaction is quite weak.^14*c*,15*d*,39*d*^ In line with this reasoning, the entropy contribution accounts for more than 80% of the barrier. Formation of ^1^B from ^1^A is appreciably exergonic (Δ*G* = −6.8 kcal mol^−1^) and has no detectable barrier (Fig. S2†). In fact, the step is strongly exothermic due to the formation of a hydrogen bond between the CO_2_ molecule and the incoming PhOH (Δ*H* = −14.6 kcal mol^−1^), but this exothermicity is partially balanced by the entropic cost of associating two fragments (^1^A and a PhOH).

In pathway I′, formation of ^1^C from ^1^B was computed to be significantly endergonic (Δ*G* = +6.9 kcal mol^−1^) and barrierless (Fig. S2†). Unlike for the formation of ^1^B, the enthalpy gain relative to the formation of an additional hydrogen bond between the oxygen of the CO_2_ and a PhOH is only moderate (Δ*H* = −4.5 kcal mol^−1^) and does not compensate for the unfavorable entropic cost relative to the association of ^1^B and PhOH. The subsequent step that consists of the C–O bond cleavage in ^1^C followed by the dissociation of the weakly bound H_2_O and PhO^−^ yielding ^1^F was estimated to be moderately exergonic (Δ*G* = −5.9 kcal mol^−1^). The exergonicity arises from the tremendous entropic contribution due to the dissociation of ^1^C into four fragments, *i.e.*^1^F, H_2_O and two phenolates (PhO^−^), even if this step involves a large positive enthalpy change (Δ*H* = +22.2 kcal mol^−1^). The conversion of ^1^C to ^1^F has to overcome a high barrier of Δ*G*^‡^ = +10.4 kcal mol^−1^ (^1^TS2), which can be attributed to lack of enough driving force for the C–O bond cleavage in ^1^C as suggested by the estimated enthalpy change (Δ*H* = +9.4 kcal mol^−1^, Table S10†). Interestingly, the free energy of the final product ^1^F is on par with that of intermediate ^1^B. Despite this, it should be noted that the reaction still proceeds, because the reduction of ^1^F to regenerate the catalyst is driven by the potential applied at the electrode.

In pathway II, the transformation of ^1^B into ^1^D consists of a proton transfer in ^1^B followed by the dissociation of the PhO^−^ from the metallacarboxylic acid. The step is moderately endergonic (Δ*G* = +7.9 kcal mol^−1^), which originates from an unfavorable enthalpy contribution, although ^1^D is stabilized by an intramolecular hydrogen bond between the COOH group and one nitrogen of the porphyrin ligand as indicated by a short N–H interatomic distance of 2.02 Å. This step needs to overcome a sizeable barrier (^1^TS3, Δ*G*^‡^ = +9.4 kcal mol^−1^) that mostly arises from the need of sufficient driving force associated with the proton transfer in ^1^B (Δ*H* = +7.3 kcal mol^−1^, Table S10†). Subsequently, the formation of complex ^1^E from ^1^D is exergonic (Δ*G* = −4.2 kcal mol^−1^), wherein a strong enthalpic contribution is balanced by an unfavorable entropic term, owing to the association of two fragments (^1^D and PhOH). No detectable kinetic barrier could be found for this step (Fig. S2†). The next step that is composed of the C–O bond scission in ^1^E and dissociation of the weakly bound H_2_O and PhO^−^ is slightly exergonic by Δ*G* = −2.7 kcal mol^−1^. The tremendous entropy contribution arising from the dissociation of ^1^E into three fragments (^1^F, PhO^−^ and H_2_O) offsets the positive enthalpy change, Δ*H* = +13.8 kcal mol^−1^, similar to the formation of ^1^F in pathway I′. This transformation needs to pass through a barrier of +8.0 kcal mol^−1^ (^1^TS4) that is on par with that of ^1^TS2 in pathway I′. In analogy to the formation of ^1^F in pathway I′, this barrier can be attributed to the significant endothermicity of the cleavage of the C–O bond in ^1^E (Δ*H* = +6.5 kcal mol^−1^, Table S10†). However, although for both pathways, the barriers leading to ^1^F are commensurate, the energy of ^1^TS2 is +5.6 kcal mol^−1^ above that of ^1^TS4. This difference largely arises from the prohibitive entropic term resulting from the association of ^1^A with two PhOH molecules, because the enthalpy term of ^1^TS2 is 4.4 kcal mol^−1^ lower than that of ^1^TS4.

To investigate whether the second proton transfer is essential to the reaction, we examined another reaction channel, in which the C–O bond cleavage takes place at intermediate ^1^D to release ^1^F and a hydroxide ion (pathway III, Fig. S3†) rather than a water molecule. The C–O bond cleavage is significantly uphill (Δ*G* = +15.2 kcal mol^−1^) due to a prohibitive positive enthalpic change (Δ*H* = +23.6 kcal mol^−1^) and, more importantly, has to cross an unconquerable barrier of Δ*G*^‡^ = +43.3 kcal mol^−1^ (^1^TS5 in Fig. S3†). In comparison with pathways I′ and II, the high enthalpic cost of the bond cleavage in the present case apparently stems from the absence of protons to trap the resulting OH^−^ to produce H_2_O, a thermodynamic sink. As a consequence, the C–O bond breaking involves an exceedingly high kinetic barrier. Our finding thus revealed that the C–O bond cleavage is necessarily concurrent with the formation of a water molecule. Following this reasoning, pathway I advocated by Costentin and co-workers^8*b*^ in which ^1^C directly dissociates into ^1^F, PhO^−^, PhOH and OH^−^ should involve a much higher barrier than that of ^1^TS2 in pathway I′ and can be safely ruled out as being a plausible mechanism.

As shown in Fig. 1, our theoretical results showed that the highest barrier in pathway I (^1^TS2) is 7.9 kcal mol^−1^ higher than that in pathway II (^1^TS3). Hence, the CO_2_ reduction process is most likely to proceed along pathway II. To further strengthen this conclusion, we carried out calculations using the double-hybrid w-B2PLYP functional in conjunction with the Def2-QZVPP basis set (Table S10†). The results indicate that ^1^TS2 is 12.0 kcal mol^−1^ lower in energy than ^1^TS3. The energy differences estimated by both density functionals considerably exceeds the typical error range of density functional theory computations.^50^ This confirms that pathway II is indeed energetically more favorable, congruent with an earlier computational study.^10^ However, the cited work did not investigate the singlet reaction, but only considered the reaction occurring on the triplet and quintet surfaces. Because the estimated barrier differences between the formation of the CO_2_ adduct ^1^A (Δ*G*^‡^ = +9.0 kcal mol^−1^), the first proton transfer (Δ*G*^‡^ = +9.4 kcal mol^−1^) and the C–O bond cleavage in pathway II (Δ*G*^‡^ = +7.9 kcal mol^−1^) fall within the typical uncertainty range of computations with hybrid DFT functionals,^51^ we cannot unequivocally determine which one is the rate-determining step of the entire catalytic process. Despite this uncertainty, our calculations strongly suggest that the C–O bond breaking involves a sizeable barrier and has to be concerted with a proton transfer, both notions consistent with earlier experimental studies.^7,8^

To investigate whether our computational model is consistent with the observed kinetics of the reaction, we computed the turnover frequency of the reaction that requires a complete and precise energy landscape of all intermediates and transition states connecting them.^52^ For pathways I′ and II, the maximum turnover frequency estimated for three different phenol concentrations, [PhOH] = 0.1, 0.75 and 3 M using the method of Costentin and co-workers^53^ is summarized in Table 1 (see the ESI† for details). The turnover frequencies calculated for pathway II are in reasonable agreement with the experimental values, while those for pathway I′ show substantial deviations. More importantly, pathway II is a first-order reaction with respect to [PhOH], consistent with the experiment, whereas pathway I′ is a second-order reaction. Therefore, pathway II qualitatively and quantitatively reproduces the experimental findings, which thus further lends credence to the reliability of our theoretical results, a necessary premise for the following analyses aiming at obtaining qualitative insights into the reaction mechanism.

**Table 1:** Calculated maximum turnover frequencies for pathways I′ and II at three different phenol concentrations, following the method described by Costentin and coworkers. The experimental value obtained from foot-of-the-wave analyses by the same authors is also displayed for comparison

	[PhOH] = 0.1 M	[PhOH] = 0.75 M	[PhOH] = 3 M
Pathway I′	4.3 s^−1^	2.4 × 10^2^ s^−1^	3.9 × 10^3^ s^−1^
Pathway II	3.6 × 10^4^ s^−1^	2.8 × 10^4^ s^−1^	1.1 × 10^6^ s^−1^
Experiment	1.8 × 10^3^ s^−1^	1.5 × 10^4^ s^−1^	1 × 10^5^ s^−1^

We also computationally investigated side reactions that lead to H_2_ and formic acid (for details, see the ESI†). Typically, these reactions start with formation of a metal-hydride species rather than a CO_2_ adduct.^54^ However, the former transformation was observed to suffer from a prohibitive kinetic barrier, compared to the generation of ^1^A. Furthermore, a previous study has pointed out that η^1^-OCO adducts can also be the precursors for producing formic acid.^39*d*^ As specified above, our calculations suggested that such an η^1^-OCO adduct probably cannot exist in the present case. Both findings likely account for the observed high product selectivity of the CO_2_ reduction catalyzed by 1^2−^.

### Electronic structure analysis of the reaction

In the following, the electronic-structure evolution of pathway II was scrutinized. The purpose is to correlate the electronic structure of ^1^1^2−^ with its exceedingly high activity toward CO_2_ functionalization, in particular, to pinpoint the role played by the non-innocent TPP ligand.

**Fig. 2:**
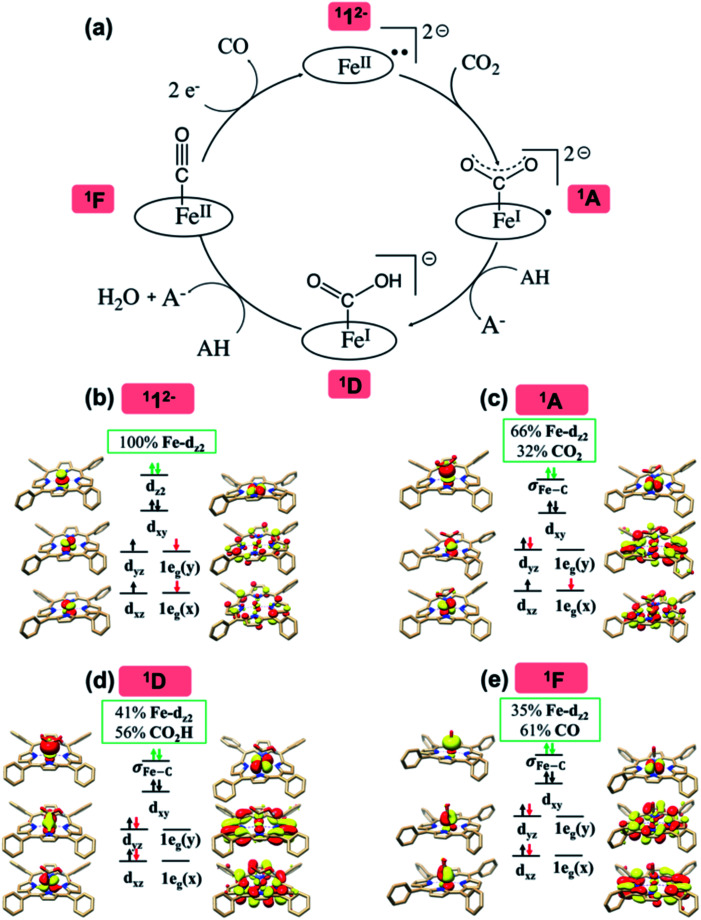
The proposed catalytic cycle of CO_2_-to-CO conversion catalyzed by ^1^1^2−^ (a), and the electronic structures of intermediates ^1^1^2−^ (b), ^1^A (c), ^1^D (d) and ^1^F (e). The electrons involved in the TPP-to-Fe intramolecular electron transfer are shown by red arrows, and those in the Fe-to-CO_2_ electron transfer by green arrows. The weight of Fe and CO_2_/CO_2_H/CO in the σ_Fe–C_ bonding MO is displayed in green boxes. For clarity, all hydrogens are omitted except those in the CO_2_H moiety.

As elaborated in our previous work,^16^ the bonding of ^1^1^2−^ is best described as an intermediate spin Fe^II^ center (*S*_Fe_ = 1) antiferromagnetically coupled with a triplet TPP˙˙^4−^ diradical (*S*_TPP_ = 1), thereby yielding an overall singlet ground state (Fig. 2b). Specifically, the Fe center features an electronic configuration of (d_*xy*_)^2^(d_*z*^2^_)^2^(d_*xz*_)^1^(d_*yz*_)^1^, and there are two electrons occupying the low lying TPP centered π* e_g_ orbitals labelled as 1e_g_(*x*) and 1e_g_(*y*) (in the *D*_4h_ point group representation). The Fe d_*xz*/*yz*_ and TPP 1e_g_ magnetic orbitals form two spin-coupled pairs that represent two antiferromagnetic exchange coupling pathways. It should be noted that the fragment orbitals of Fe d_*xz*/*yz*_ and TPP e_g_ belong to the same representation (e_g_) of the effective *D*_4h_ point group of ^1^1^2−^; therefore, their interactions are symmetry-allowed as indicated by the computed considerable overlap of the two spin coupled pairs (0.33). If both fragment orbital sets transformed as different irreducible representations of the effective *D*_4h_ point group, their exchange interaction would feature ferromagnetic coupling on the grounds of the Goodenough–Kanamori rule^55^ rather than antiferromagnetic coupling as determined experimentally.^16,47^ In line with this reasoning, the corresponding quintet state (^5^1^2−^), which features *ferromagnetic* coupling of these two fragments, was estimated to lie 12.1 kcal mol^−1^ higher in energy than the singlet state.

As depicted in Fig. 2c, the driving force to generate ^1^A largely stems from the σ donation from the doubly occupied Fe d_*z*^2^_ orbital to the vacant CO_2_ in-plane π* 
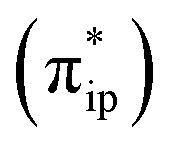
 orbital. The electron acceptor is one of the lowest unoccupied molecular orbitals (LUMOs) of free CO_2_ formed by an out-of-phase combination of the 2p_*z*_ orbitals of the central C atom and the two terminal O atoms and has a larger lobe at the central C atom than those at the two terminal O atoms. Upon CO_2_ binding, it gets considerably bent with an O–C–O angle of 130° compared to 180° for uncoordinated CO_2_ molecules. As elaborated earlier,^39*d*^ such a geometric distortion not only significantly decreases the energy of the CO_2_
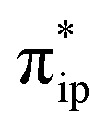
 orbital, but also increases the C-p character in it. Thus, the energy difference between the CO_2_
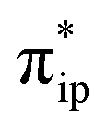
 and Fe d_*z*^2^_ orbitals drops, and their overlap becomes more favored. Consequently, both factors work in synergy to enhance the Fe–CO_2_ interactions. Despite this, the resulting bonding MO, hereafter referred to as σ_Fe–C_, contains only 32% CO_2_ π* parentage; hence, the bonding remains quite weak, consistent with a moderate enthalpy change of −5.4 kcal mol^−1^ estimated for the formation of ^1^A (*vide supra*). Nevertheless, this essentially dative interaction can be viewed as partial electron transfer from the Fe center to the CO_2_ moiety. Interestingly, the MO analyses revealed that the formation of ^1^A is accompanied by an intramolecular β electron transfer from TPP 1e_g_(*y*) to Fe d_*yz*_, whereas the other spin-coupled pair consisting of the Fe d_*xz*_ and TPP 1e_g_(*x*) MOs remains intact. Different from ^1^1^2−^, the Fe d_*xz*_ and d_*yz*_ orbitals in ^1^A are not energetically degenerate anymore, because the former is destabilized by the repulsion with the doubly occupied σ orbitals of the two C–O bonds; while the latter is stabilized by its back-donation to the CO_2_ out-of-plane π* orbital, the other LUMO of free CO_2_. Consequently, the electronic structure of ^1^A is best formulated as having a low spin Fe^I^ center (*S*_Fe_ = 1/2) that is bound to an approximately charge-neutral CO_2_ and is antiferromagnetically coupled with a TPP˙^3−^ radical (*S*_TPP_ = 1/2), thus giving an overall singlet ground state. More importantly, the TPP-to-Fe electron transfer mitigates the depletion of the electron density of the Fe center resulting from the Fe-to-CO_2_ σ donation. Therefore, the CO_2_ association process does not engender substantial variation of the electron density of the Fe center.

Compared to uncoordinated CO_2_, the bent CO_2_ moiety is primed for protonation to afford ^1^D. Besides the influence on its LUMO discussed above, the CO_2_ bending also increases the energy of its highest occupied molecular orbital (HOMO), an antisymmetric combination of the two O lone pairs.^39*d*^ The interaction of the O lone pairs with a proton is therefore favored by the geometric distortion of the CO_2_ moiety. Our theoretical results revealed that the protonation causes an increase of the CO_2_
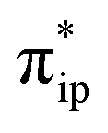
 parentage in the doubly occupied σ_Fe–C_ MO from 32% in ^1^A to 56% in ^1^D (Fig. 2d), thereby indicating substantial covalent character for the Fe–CO_2_H interaction. Consequently, upon going from ^1^A to ^1^D, the calculated Fe–C_CO_2__ bond distance shortens from 2.02 Å to 1.89 Å. This bonding description suggests that, on average, the CO_2_ ligand is reduced by one electron at the stage of ^1^D. As such, the conversion of ^1^A to ^1^D is best described as a concerted proton-electron transfer (CPET) to the CO_2_ ligand.^56^ Moreover, the TPP ligand in ^1^D returns to its usual state of a closed-shell dianion, because the protonation is companied by a β electron transfer from TPP 1e_g_(*x*) to Fe d_*xz*_. Due to the high covalency of the Fe–CO_2_H bond, ^1^D has to be described as a resonance hybrid of a low spin Fe^II^ ion (*S*_Fe_ = 0) bound to a ^−^C(O)OH ligand and a low spin Fe^0^ center (*S*_Fe_ = 0) coordinated by a ^+^C(O)OH ligand. In analogy to the preceding step, the TPP-to-Fe electron transfer balances the Fe-to-CO_2_ electron transfer, and the electron density of the Fe center remains largely unchanged.

The MO diagram of ^1^TS4 (Fig. S8†) suggests that, over the course of the heterolytic C–O bond breaking, the C–O σ bonding orbital evolves into a lone pair of the O atom in the H_2_O product. Accordingly, the resulting C atom formally becomes a carbocation center, and due to its exceedingly high electron-accepting capability, the σ_Fe–C_ MO of ^1^F acquires more C character (61%) as the expense of the weight of the Fe d_*z*^2^_ atomic orbital dropping to 35% (Fig. 2e). Hence, at this stage, the two-electron transfer from the Fe d_*z*^2^_ orbital to the CO_2_
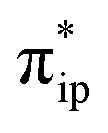
 orbital is largely completed. Of note, ^1^F also features strong back-donation from the doubly-occupied Fe d_*xz*_ and d_*yz*_ orbitals to the C–O π* orbitals. Thus, ^1^F is best described as containing a low spin ferrous center bound to a CO ligand and a closed-shell porphyrin dianion.

### The role of the non-innocent TPP ligand in the reaction

In the present case, the active species ^1^1^2−^ is formed by a TPP centered two-electron reduction of ^3^1 at a mild potential. Because the electron acceptors are the highly delocalized TPP centered 1e_g_ orbitals, the additional electron density is distributed to the twenty-four atoms of the porphyrin ligand. Therefore, the reduction does not significantly escalate the interelectronic repulsion of all delocalized π electrons of the reduced TPP^4−^ ligand. For the same reason, this electron transfer is subjected to a low degree of geometric distortions and hence a reduced reorganization energy. This contrasts with metal centered redox processes for which much more compact and localized d orbitals function as redox active orbitals; consequently, reduction typically causes a much higher gain in interelectronic repulsion. Likewise, a greater reorganization energy is anticipated because of the more significant distortions of the first coordination sphere of the metal center. Typically, two-electron reduction processes of 3d transition metal ions cannot readily occur, because the required reduction potentials are often exceedingly negative. The resulting complexes are likely to be highly reactive and may involve various facile side-reactions or deactivation pathways for electron-richer metal centers, such as H_2_ or HCOOH generation (see the ESI†), instead of participating in the target reaction. Therefore, the non-innocent nature of TPP explains why ^1^1^2−^ can be generated at a mild potential and its catalyzed reaction exhibits high faradaic efficiency for CO production.

Although the reducing equivalents are stored on the TPP ligand, CO_2_ must associate with the metal center for further activation. This is due to the completely delocalized nature of the π-electrons; because none of the C atoms of TPP possesses sufficient electron density allowing for facile CO_2_ binding. In this regard, the Fe center is superior, because its high-lying doubly populated d_*z*^2^_ orbital has an appropriate shape and can efficiently overlap with the CO_2_
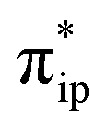
 orbital.^39*d*^ Hence, the Fe center should be the active site for CO_2_ functionalization. However, the required reducing equivalents are stored at the ligand.

The above described situation gives rise to an intriguing question of how the metal center communicates with the ligand in order to efficiently transfer the reducing equivalents to the CO_2_ moiety. As elaborated above, the TPP to CO_2_ two-electron transfer required for conversion of CO_2_ to CO is achieved by two simultaneous electron transfer events. One is the two-electron transfer from Fe d_*z*^2^_ to CO_2_
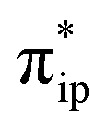
, and the other is the two-electron transfer from TPP 1e_g_ to Fe d_*xz*/*yz*_. Both electron-transfer events are coupled in such a way that the electron density of the Fe center does not vary significantly throughout the reaction. This reduces the energy resulting from the adjustments in the first coordination sphere of the Fe center, while maintaining the electron donating abilities of the Fe center, thereby preventing the formation of highly energetic intermediates or transition states. This analysis underscores the fundamental importance of the non-innocence of the TPP ligand and its cooperativity with the Fe center to the reactivity, which, at least in part, rationalizes why ^1^1^2−^ exhibits the exceedingly high catalytic activity towards CO_2_ reduction.

What happens if the TPP-to-Fe electron transfer cannot take place? As detailed in the ESI,† the triplet state of [Fe(TPP)]^2−^ (^3^1^2−^) was computed to lie 5.7 kcal mol^−1^ above ^1^1^2−^. It also contains an intermediate spin ferrous center but interacts with an open-shell singlet TPP˙˙^4−^ diradical with one α electron and one β electron occupying its 1e_g_(*x*) and 1e_g_(*y*) orbitals, respectively (Fig. S5†). In analogy to the electronic structure changes found for the singlet reaction, CO_2_ binding to ^3^1^2−^ is also driven by a weak σ donation from Fe d_*z*^2^_ to CO_2_
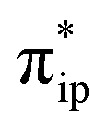
, concomitant with a spin-allowed electron transfer of a β electron from TPP 1e_g_(y) to Fe d_*yz*_. Consequently, the triplet Fe–CO_2_ adduct (^3^A) is best described as having a low spin Fe^I^ center *ferromagnetically* coupled with a TPP˙^3−^ radical, and its formation was estimated to be thermodynamically equally probable (Δ*G* = −0.2 kcal mol^−1^) with that of ^1^A (Δ*G* = +2.5 kcal mol^−1^) within the error range of DFT computations. Subsequently, protonation of ^3^A to yield ^3^D indeed induces further electron transfer from Fe to CO_2_, but unlike on the singlet surface, the electron transfer from TPP 1e_g_(*x*) to Fe d_*xz*_ cannot occur because this process is spin-forbidden. Hence, upon going from ^3^A to ^3^D, the electron density at the Fe center is significantly depleted. Congruent with this reasoning, generation of ^3^D was calculated to be uphill to Δ*G* = +10.0 kcal mol^−1^, whereas that of ^1^D is only Δ*G* = +1.1 kcal mol^−1^. In fact, the cost for the formation of ^3^D is even greater than the highest barrier in the singlet pathway. As such, these findings demonstrate that synchronization of the TPP-to-Fe electron transfer with the Fe-to-CO_2_ electron transfer is fundamentally crucial to the reaction kinetics.

**Fig. 3:**
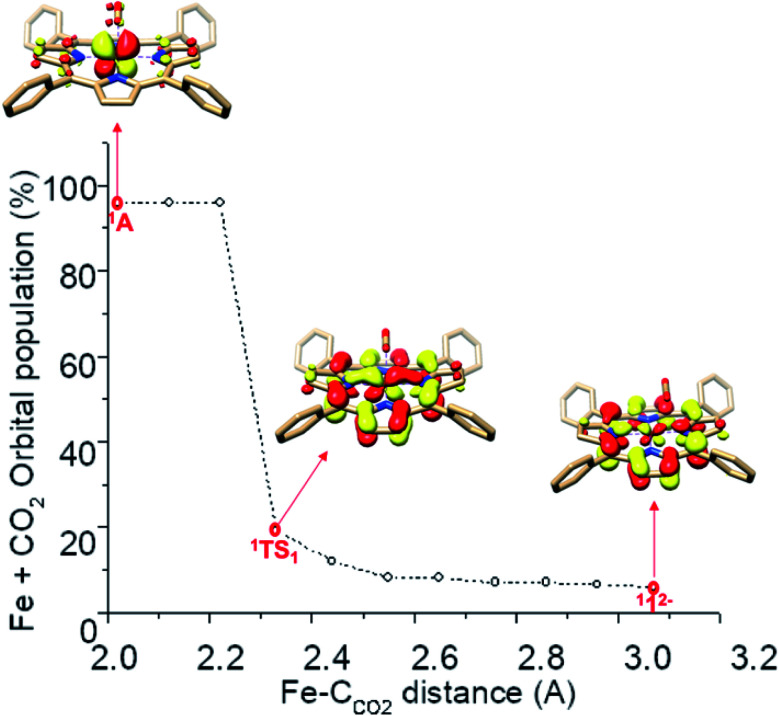
Evolution of the sum of Fe and CO_2_ Löwdin population of the β (spin-down) magnetic orbital 1e_g_(*y*) as a function of the Fe–C_CO_2__ distance during the formation of intermediate ^1^A. The dots correspond to the structures calculated along the relaxed surface scan. The red dot corresponds to ^1^A, ^1^TS1 and ^1^1^2−^ (from left to right).

How does the system synchronize the TPP-to-Fe electron transfer with the Fe-to-CO_2_ electron transfer? First, the TPP e_g_ and Fe d_*xz*,*yz*_ fragment orbitals have comparable energy, otherwise the electron transfer would not be thermodynamically feasible. Most importantly, both sets transform as the same irreducible representation of the effective *D*_4h_ point group of ^1^1^2−^. Consequently, they form two spin-coupled pairs with considerable overlaps. The antiferromagnetic coupling between the ligand and the metal center lowers the energy of the singlet energy surface compared to that of higher spin multiplicities and ensures that the electron transfer does not incur an energetically unfavorable spin crossover.^57^ As seen in Fig. 3, along the reaction coordinates of the formation of ^1^A, the sum of Fe and CO_2_ populations of the β-1e_g_(*x*) magnetic orbital slowly increases from about 6% in ^1^1^2−^ to 20% in ^1^TS1 and then increases drastically and finally reaches 98% in ^1^A. Such a continuous electron transfer minimizes the variation of electron density of the metal center along the reaction coordinates. Consequently, the loss of Fe electron density due to the Fe to CO_2_ transfer is immediately compensated by an increasing TPP to Fe electron delocalization in the magnetic orbitals, which likely lowers the activation barrier by increasing the donating abilities of the metal center, even when the complete ligand-to-metal electron transfer lacks significant thermodynamic driving forces. Hence, the antiferromagnetic coupling is pivotal in synchronizing the two electron-transfer events.

The above analyses correlate the electronic structure of ^1^1^2−^ with its high activity. In fact, non-innocent ligands were found for a range of CO_2_ reduction catalysts. Besides ^1^1^2−^, we have analyzed the electronic-structure evolution in the course of the CO_2_-to-CO reactions mediated by at least ten related catalysts (Scheme 2) that are also supported by non-innocent ligand platforms (for details, see the ESI†). For all these systems, the metal center acts as the active site for CO_2_ functionalization, but the non-innocent ligand acts as the electron reservoir. Like ^1^1^2−^, this requires the metal-to-CO_2_ electron transfer to be coupled with the ligand-to-metal electron transfer. Our results demonstrated that the main differences among them lie in the nature of the chemical steps accomplishing the ligand-to-metal electron transfer, according to which these catalysts are divided into three categories.

Category I catalysts include ^1^1^2−^ (Fig. 2), ^2^2^+^ (Fig. S9†)^12^ and ^3^3^0^ (Fig. S10†).^19^ The active species consists of a metal center in its usual oxidation state that is antiferromagnetically coupled with a one- (^2^2^+^) or two-electron reduced ligand diradical (^1^1^2−^ and ^3^3^0^). This category is distinguished by the ligand-to-metal electron transfer being achieved by two separate one-electron transfer events; one takes place during the CO_2_ adduct formation step, and the other during the first protonation step.

Category II catalysts consist of ^2^4^0^, ^2^5^0^ and ^2^6^0^,^12–14^ which either feature a metal center antiferromagnetically coupled with a two-electron reduced ligand in a triplet state (^2^4^0^, ^2^5^0^), or a metal center coupled with a doubly reduced ligand in a singlet state (^2^6^0^). Irrespective of the electronic structures, the electron transfer pathways are identical for these three catalysts. The characteristics of category II catalysts are that, for two separate ligand-to-metal electron transfer events, one occurs during the CO_2_ adduct formation step, and the other during the final C–O bond cleavage step. Here we take the reaction with ^2^4^0^ as an example to discuss the electronic-structure evolution along the reaction coordinate, and summarize those for ^2^5^0^ and ^2^6^0^ in the ESI (Fig. S11 and S12†).

The reaction mechanism of selective CO_2_ to CO conversion catalyzed by ^2^4^0^ under wet conditions has been subjected to extensive computational and experimental studies.^14^ It was found that ^2^4^0^ first binds CO_2_ to form a η^1^-CO_2_ adduct ^2^G, which then undergoes protonation to yield metallacarboxylic acid ^2^H, and the C–O bond is cleaved concomitant with the second proton transfer, furnishing metal-carbonyl intermediate ^2^I and releasing a water molecule.

**Fig. 4:**
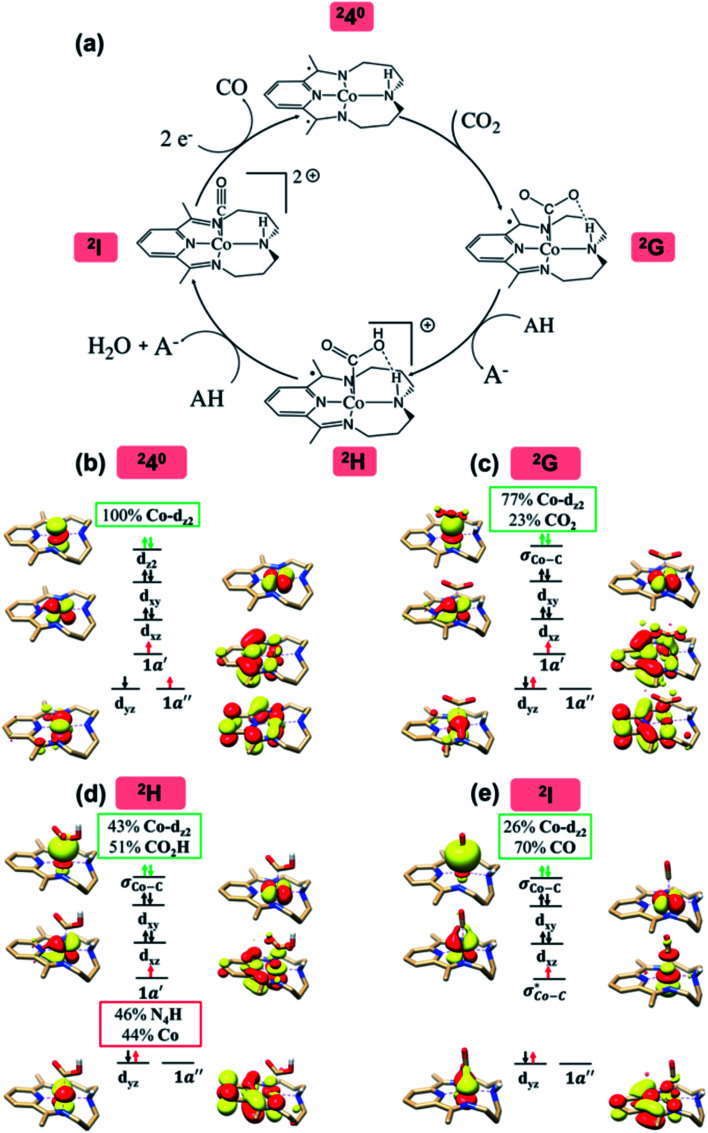
The proposed catalytic cycle of CO_2_-to-CO conversion catalyzed by ^2^4^0^ (a),^14*e*^ and the electronic structures of the intermediates ^2^4^0^ (b), ^2^G (c), ^2^H (d) and ^2^I (e). Dashed straight lines between atoms represent hydrogen bonds. Electrons involved in the ligand-to-metal intramolecular transfer are shown by red arrows. Electrons involved in the metal-to-CO_2_ electron transfer are shown by green arrows. The weight of Co and CO_2_/CO_2_H/CO in the σ_Co–C_ molecular orbital is displayed in green boxes. In ^2^H, the weight of Co and N_4_H in the singly-occupied 1a′ molecular orbital is displayed in red boxes. For clarity, all hydrogens are omitted except the hydrogen of the CO_2_H moiety and that involved in the intramolecular hydrogen bond between the CO_2_H moiety and the amine of the N_4_H ligand.

According to the reported reaction pathway^14^ shown in Fig. 4a, we analyzed the electronic structure changes during the reaction. Our present investigation supports the notion that the pyridine-diimine ligand in ^2^4^0^ is non-innocent, consistent with earlier studies.^14*c*,18,58,59^ As shown in Fig. 4b, ^2^4^0^ was found to be composed of a low spin Co^II^ (*S*_Co_ = 1/2) antiferromagnetically coupled with a N_4_H˙˙^2−^ diradical (*S*_N_4_H_ = 1). This bonding description indicates that, similar to TPP^2−^, the N_4_H^0^ ligand can harbor additional two electrons in its conjugated π system. Our MO analysis (Fig. 4b) suggests that ^2^4^0^ contains a Co^II^ center featuring an electron configuration of (d_*xy*_)^2^(d_*z*^2^_)^2^(d_*xz*_)^2^(d_*yz*_)^1^ and two delocalized unpaired electrons populating the N_4_H π* based 1a′ and 1a′′ MOs, labelled according to their symmetry representation of *Cs* point group. Of note, besides the N_4_H 
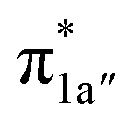
 fragment orbital (71%), the Co d_*yz*_ atomic orbital (29%) makes a sizeable contribution to the 1a′′ MO, because both transform as the A′′ representation of *Cs* point group. Consequently, the magnetic orbitals of N_4_H 
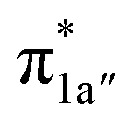
 and Co d_*yz*_ overlap significantly, hence leading to strong antiferromagnetic coupling between the ligand and the metal center.

Upon formation of ^2^G from ^2^4^0^, the doubly-occupied Co d_*z*^2^_ orbital slightly mixes with the unoccupied CO_2_
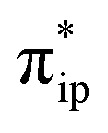
 orbital. The resulting Co–CO_2_ bond is thus essentially dative, as suggested by the estimated dominant Co percentage in the σ_Co–C_ MO (77% Co, 23% CO_2_, Fig. 4c). This step is coupled with an electron transfer from N_4_H 
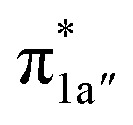
 to Co d_*yz*_ such that the resulting intermediate (^2^G) is best described as a low-spin (*S*_Co_ = 0) Co^I^ center weakly bound to a CO_2_^0^ ligand and to an N_4_H˙^−^ radical (*S*_N4H_ = 1/2). The subsequent conversion of ^2^G into ^2^H substantially increases the covalency of the Co–C_CO_2_H_ interaction, as suggested by σ_Co–C_ containing nearly identical percentages of Co and CO_2_H (43% Co and 51% CO_2_H, Fig. 4d). Hence, this step can be viewed as the first Co-to-CO_2_ electron transfer. Because 
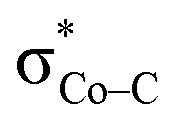
 and N_4_H 
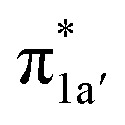
 belong to the A′ representation of *Cs* point group, both fragment orbitals can interact with each other, and the resulting 1a′ MO acquires sizeable character of 
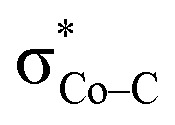
 (Co d_*z*^2^_ 44% and CO_2_H 10%). This orbital mixing can be interpreted as a *partial* electron transfer from the N_4_H ligand to the Co center. The final C–O bond heterolytic cleavage to afford ^2^I and H_2_O further increases the CO character in σ_Co–C_ (26% Co and 70% CO, Fig. 4e). Therefore, it is reasonable to consider this step as the second Co-to-CO_2_ electron transfer, accompanied by completion of the N_4_H 
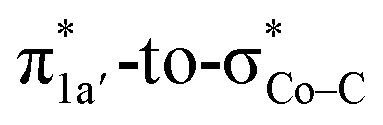
 electron transfer. Consequently, this results in a Co^II^ center bound to a carbonyl ligand in ^2^I.

The reaction entails two intramolecular electron transfer events: a metal-to-CO_2_ transfer that is affected by σ donation from the doubly-occupied Co d_*z*^2^_ to the unoccupied CO_2_
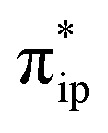
 orbital, and a N_4_H-to-Co electron transfer that makes up for the loss of electron density on the metal center. Both events are coupled efficiently *via* the mixing of the ligand-centered electron donating orbitals (N_4_H 
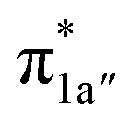
 and 
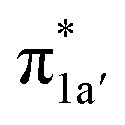
) and the metal-based electron accepting orbitals (Co d_*yz*_ and d_*z*^2^_) of the appropriate symmetry and comparable energies. This orbital mixing gives rise to a spin-coupled orbital pair involving Co d_*yz*_ and N_4_H 
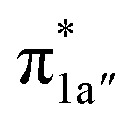
 and a singly-occupied delocalized MO composed of N_4_H 
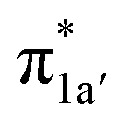
 and 
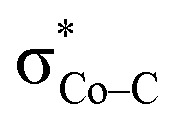
. The first electron transfer route involving the spin-coupled pair is the same as that found for the reaction with ^1^1^2−^. On the other hand, the second pathway chooses 
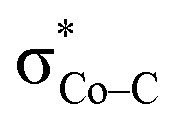
 instead of Co d_*xz*_, because Co d_*xz*_ is doubly populated in ^2^4^0^, and 
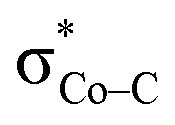
 is the lowest-lying vacant Co d orbital besides Co d_*yz*_. However, due to the high energy of 
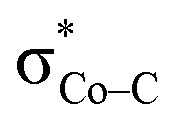
, a significant depletion of the electron density on the metal center is required to give this electron transfer a sufficient thermodynamic driving force. Hence, unlike in ^1^1^2−^, the transfer is not yet complete in ^2^H.

Category III catalysts include ^1^7^−^,^15^^1^8^−^,^26^^1^9^2−^,^27^^1^10^0^,^28^ and ^1^11^0^,^20^ all of which contain metal centers coupled with closed-shell, doubly-reduced ligands. For category III catalysts, the ligand-to-metal transfer is entirely completed during the formation of the metal–CO_2_ adduct. Here we take the reaction with ^1^7^−^ as an example to discuss the electronic structure evolution along the reaction coordinate. The reactions with ^1^9^2−^, ^1^8^−^, ^1^10^0^ and ^1^11^0^ are discussed in the ESI (Fig. S13–S16†).

**Fig. 5:**
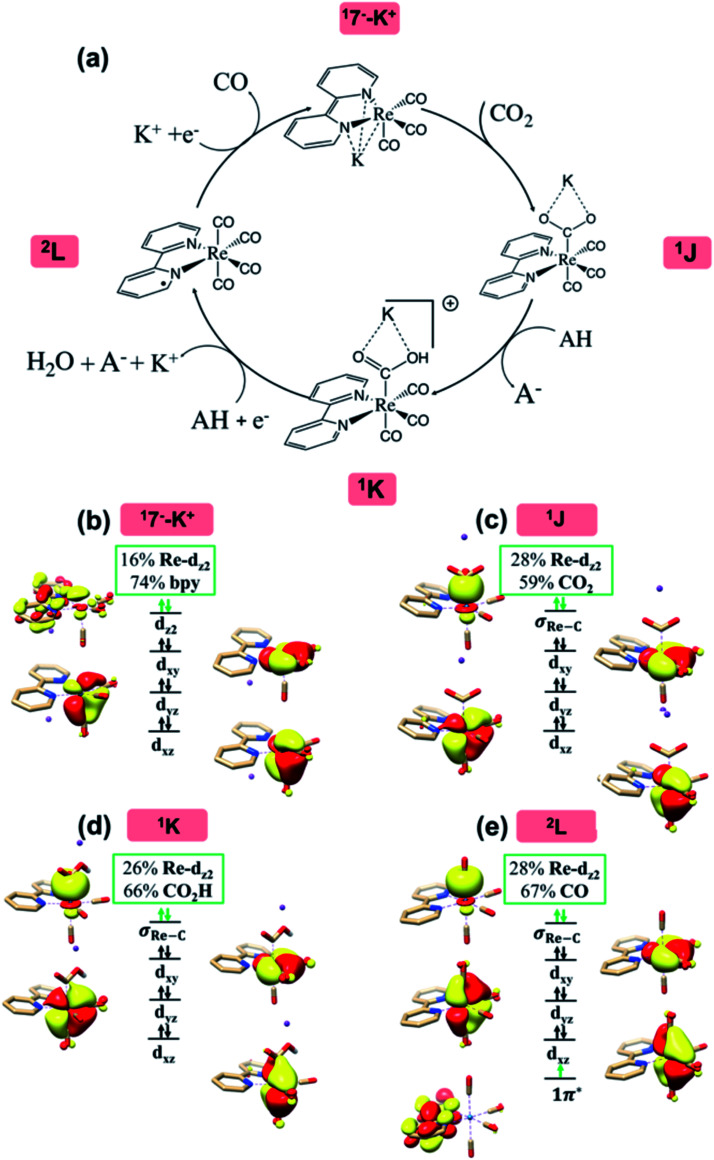
The proposed catalytic cycle of CO_2_-to-CO conversion catalyzed by ^1^7^−^ (a),^15*d*^ and the electronic structure of the intermediates ^1^7^−^–K^+^ (b), ^1^J (c), ^1^K (d) and ^2^L (e). Electrons involved in the ligand-to-metal and metal-to-CO_2_ intramolecular transfer are shown by green arrows. The weight of Re and bpy in the 1π* molecular orbital (in ^1^7^−^–K^+^) and the weight of Re, bpy and CO_2_/CO_2_H/CO in the σ_Re–C_ bonding orbital (in all other intermediates) are displayed in green boxes. For clarity, all hydrogens are omitted except the hydrogen of the CO_2_H moiety.

It was found in the 1980s that ^1^7^−^ and its derivatives can catalyze selective CO_2_-to-CO reduction, and the reaction exhibits one of the highest turnover frequencies reported for homogeneous catalysis (Table S1†).^15*h*^ The mechanism of CO_2_ reduction by ^1^7^−^ has been extensively studied, particularly in the work published by Keith and co-workers.^15*d*^ In their original study, it was shown that the inclusion of a K^+^ counterion in the vicinity of ^1^7^−^ drastically improved the description of the redox potentials associated with the formation of the active species.^15*d*^ Hence, the active species was proposed to be the ion pair ^1^7^−^–K^+^ (Fig. 5a), rather than anionic complex ^1^7^−^.^15*d*^ The reduction of CO_2_ is initiated by binding of CO_2_ to ^1^7^−^–K^+^ to form an η^1^-CO_2_ adduct ^1^J, which is then protonated to afford carboxylic acid ^1^K. Subsequently, ^1^K undergoes a second reduction at the electrode, and the C–O bond breaking is concomitant with a second proton transfer, ultimately yielding ^2^L and releasing a water molecule and the K^+^ counterion.

As the electronic structure changes of the proposed reaction pathway have been elaborated previously,^15*d*^ here we recapitulate only important features in order to compare them with the aforementioned cases. Earlier experimental and computational studies^15*e*,60^ revealed that the diamagnetic ground state of ^1^7^−^ is best formulated as a low spin Re^I^ center (*S*_Re_ = 0) ligated by a singlet bpy^2−^ ligand. Binding of K^+^ to ^1^7^−^ does not discernibly change the electronic structure. Specifically, the Re center of ^1^7^−^–K^+^ features an electronic configuration of (d_*xy*_)^2^(d_*xz*_)^2^(d_*yz*_)^2^, and its HOMO (1π*), albeit with dominant bpy π* parentage (74%), contains nonnegligible Re d_*z*^2^_ character (16%) (Fig. 5b). The mixing of these two fragment orbitals is symmetry-allowed, because both belong to the 1A′ representation of *Cs* point group. Furthermore, the Re center moves out of the bipyridine plane, allowing a stronger overlap between the two fragments.

As CO_2_ approaches the Re center of ^1^7^−^–K^+^ to form ^1^J, two-electron transfer from bpy π* to the formally empty Re d_*z*^2^_ orbital takes place, and simultaneously the latter orbital donates the electron density to the CO_2_
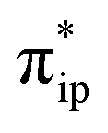
 orbital (Fig. 5c). The resulting bonding σ_Re–C_ MO of ^1^J has a 59% contribution from the CO_2_
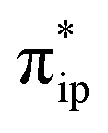
 orbital. Thus, during this elementary step, on average, approximately one electron is transferred from the ligand to CO_2_*via* the metal center. Due to the substantial covalent character of the Re–CO_2_ interaction, the electronic structure of ^1^J is thus best described as a resonance hybrid between a Re^I^ center (*S*_Re_ = 0) bound to a CO_2_^2−^ ligand and a Re^−I^ ion (*S*_Re_ = 0) interacting with a CO_2_^0^ moiety. As shown in Fig. 5d, the subsequent protonation further polarizes the Re–C bond in ^1^K as indicated by the increased –CO_2_H weight in the σ_Re–C_ orbital (Re d_*z*^2^_ 26% and CO_2_H 66%). Finally, an outer-sphere electron transfer from the electrode to the bpy π* orbital occurs, along with the C–O bond cleavage, thereby yielding intermediate ^2^L and releasing a water molecule.

Again, the reaction with ^1^7^−^ also involves two coupled metal-to-CO_2_ and ligand-to-metal electron transfer events, which shift the two electrons stored on bpy directly to the CO_2_ ligand while leaving the Re oxidation state essentially unchanged. The coupling of the two electron transfer events is accomplished by mixing of the bpy π* (electron donor) and Re d_*z*^2^_ (electron acceptor) fragment orbitals, because of their appropriate symmetry and considerable overlap. Different from ^1^1^2−^, in the present case, the orbital mixing leads to a doubly occupied delocalized MO formed by Re d_*z*^2^_ and bpy π* rather than two spin-coupled pairs. Because the Re–CO_2_ interaction lowers the energy of the Re d_*z*^2^_ orbital, CO_2_ association triggers the direct bpy-to-Re two-electron transfer. This contrasts with the two categories discussed above, whose ligand-to-metal electron transfer proceeds *via* two separate events.

### Basic electronic structure requirements for catalysts with non-innocent ligands

All the CO_2_-to-CO catalytic reactions studied above involve two synchronized electron transfer events: (1) a metal-to-CO_2_ two-electron transfer, and (2) a ligand-to-metal one- or two-electron transfer. The former is always effected mainly *via* σ-donation of two electrons from the doubly-occupied metal-centered d_*z*^2^_ orbital to the empty CO_2_ π* orbital. The latter transfer connects highly delocalized occupied ligand π* orbitals with formally unoccupied metal d orbitals. The ligand–metal cooperativity is at the core of the reactivity for all these complexes, thereby ensuring that the electrons stored on the ligand are transferred to CO_2_*via* the metal center.

How does the system ensure the synchronization of the two electron transfer events? All of the investigated catalysts feature strong interactions between the ligand-based electron-donating π* orbital and the metal-based electron-accepting d-orbital, which give rise to molecular orbitals delocalized over the ligand platform and the metal center. In ^1^1^2−^, the strong antiferromagnetic coupling of the TPP e_g_ orbitals with the d_*xz*,*yz*_ atomic orbitals yields the delocalized 1e_g_ MOs. In ^2^4^0^, the N_4_H 
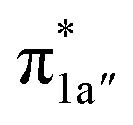
 fragment orbital mixes with the Co d_*yz*_ atomic orbital, and at the later stage, the N_4_H 
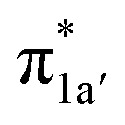
 fragment orbital interacts with the 
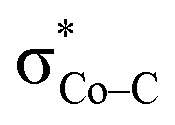
 fragment orbital to give the orbitals 1a′′ and 1a′, respectively. In ^1^7^−^, the bpy π* fragment orbital mixes with the Re d_*z*^2^_ orbital to generate the 1π* molecular orbital. Irrespective of different interaction modes, the coupling of ligand- and metal-based orbitals is pivotal in ensuring that even marginal losses of the electron density on the metal center is compensated by a continuous ligand-to-metal electron delocalization, as found for the case of 1^2−^. This is because the aforementioned orbitals have the same symmetries and similar energies; otherwise, such coupling could not happen.

This work is perfectly in line with the earlier studies on the role of ligand non-innocence in general catalysis.^22–25^ In terms of reactivity, although ligand-centered mechanisms have been occasionally reported,^61,62^ in the vast majority of cases, the reactions completely occur at the metal center. In the latter situations, the ligands serve as an “electron reservoir”, thus enabling the metal center to maintain its common oxidation state as the reaction progresses,^63,64,68^ and the electrons stored on the ligand are transferred to the metal center during the reaction.^22,63–65^ Again, such catalysts also feature significant mixing between the ligand-based redox-active orbitals and unoccupied metal d-orbitals,^66^ in analogy to our present analysis.

Taken together, for all systems under investigation, non-innocent ligands endorse the following properties to CO_2_-to-CO electrocatalysts. First and foremost, ligand-centered reductions enhance the stability of the active species by providing low-lying redox-active orbitals. By contrast, metal-centered reduction would involve the formation of highly reactive species which are likely deactivated by side reactions or initiate unwanted reactions at electron-rich metal centers, like H_2_ or HCOOH production. Furthermore, the ligand–metal cooperativity is essential to the kinetics of the reaction. Owing to the synchronization of both electron transfer events, the metal oxidation state remains unchanged throughout the reaction. In the absence of such synchronization, the reaction would necessarily generate high-energy intermediates as exemplified by the triplet reaction of ^3^1^2−^.

### General molecular structure requirements for catalysts with non-innocent ligands

So, how can one design catalytic systems that take the best advantage of the aforementioned properties? First of all, systems with open coordination sites are preferred, which facilitates the σ-bonding with CO_2_. This step often lacks suitable driving force,^10,15*d*,20^ and, in particular, ligand substitution by CO_2_ can hardly occur. In this regard, four-coordinated planar or five-coordinate square-pyramidal complexes are excellent candidates.

The synchronization of the ligand-to-metal and metal-to-CO_2_ electronic transfer is also dependent on the coupling between the ligand and the metal center, which requires orbitals of similar energies and the same symmetry. Typically, highly conjugated π-systems have a wide range of available MOs, and their varying energies are more likely to match those of the metal d-orbitals. These set orbitals also feature various symmetries, which ensures that such coupling is symmetry allowed. For these two reasons, highly conjugated ligand platforms are clearly excellent candidates. Furthermore, in order to maximize overlap between the lobes of the interacting ligand- and metal-based fragment orbitals, the atoms of the ligand interacting with the metal center should employ their p_*z*_ orbitals in the π-system. For instance, conjugated sp_2_ nitrogen donors or carbenes are excellent candidates.

Lastly, the choice of the metal center influences the metal–ligand cooperativity. The second or third row transition metals have larger, less compact d orbitals than first-row congeners and are likely to delocalize more electron density to the ligand. For instance, the molecular orbital of [Mn(bpy)(CO)_3_]^−^ is much more centered at the metal (24%, Fig. S13†) than its rhenium counterpart, [Re(bpy)(CO)_3_]^−^ (16%, Fig. 5). For the same reason, 4d and 5d metals are also expected to donate more electron density to the CO_2_ ligand, thus facilitating CO_2_ reduction. This, at least in part, explains why rhenium-bipyridine catalysts typically exhibit higher catalytic performances than their manganese counterparts.

In a given row of the periodic table, metals on the left tend to be less electronegative than those on the right. Consequently, the latter are expected to donate less electron density to the CO_2_ molecules than the former. Besides this, the number of valence d-electrons also plays a significant role in the reactivity. The number differs among metals of the same oxidation state; therefore, the available intramolecular electron transfer pathways also change, leading to drastic differences in mechanisms and catalytic performances. For instance, [Fe(qpy)] belongs to category I, while [Co(qpy)] belongs to category II. Because the d_*xz*_ orbital is doubly-occupied in the latter, one of the electron transfer pathways available in the former case is inactivated in the latter, which results in the shift from category I to category II. Furthermore, relative to [Fe(qpy)], [Co(qpy)] exhibits a better catalytic performance (Table S1†). In sharp contrast, [Fe(TPP)] by far outperforms [Co(TPP)], although both Fe compounds feature an analogous electronic configuration and so do the Co compounds.^67^ Clearly, more detailed investigations are required to pinpoint the exact relationship between the type of metal–ligand cooperativity and the catalytic performance.

## Conclusion

Our computational investigation of the CO_2_-to-CO reduction reaction catalyzed by ^1^1^2−^ proposes the following mechanism: (1) Formation of the adduct, (2) protonation of the adduct, (3) cleavage of the C–O bond, and (4) reduction of the metal-carbonyl and release of a CO molecule. We found a satisfying agreement between the predicted mechanism and the available thermodynamic and kinetic data. In particular, we found that the highest barrier was indeed compatible with the observed turnover frequency of this catalytic reaction.

Most importantly, our present investigation provides a profound understanding of the structure–activity relationship of ^1^1^2−^. Indeed, the reaction involves two electron transfer events: (1) a metal-to-CO_2_ transfer and (2) a ligand-to-metal electron transfer. These two electron transfer events are synchronized in such a way that the electron density of the metal center hardly varies along the reaction coordinates. It was shown that the synchronization of the two electron transfer events is fundamental to the reactivity, because it circumvents the formation of intermediates having highly electron-rich or -deficient metal centers. Optimal synchronization is realized by the antiferromagnetic spin coupling between the porphyrin ligand and the metal center, which serves as a conduit between them; consequently, the variation of electron density on the metal center is minimized.

Comparison of the reactivity of 1^2−^ with those of ten related catalysts with non-innocent ligand platforms enables the generalization of the feature of ligand non-innocence in CO_2_ reduction observed for 1^2−^. On the basis of these findings, we propose that ligand non-innocence in CO_2_ reduction plays a central role in ensuring a high selectivity and stability, while maintaining fast kinetics through ligand–metal cooperativity. Finally, fundamental requirements to design catalysts with non-innocent ligands are proposed.

## Author contributions

S. Y. and F. N. conceived and supervised the project; M. T. carried out theoretical computations. All authors wrote the manuscript.

## Conflicts of interest

The authors declare no conflict of interest.

## Supplementary Material

SC-013-D2SC01863B-s001
