# Supplementary material for: Electronic structure analysis of electrochemical CO2 reduction by iron-porphyrins reveals basic requirements for design of catalysts bearing non-innocent ligands
Source: Chem Sci. 2022 Jun 29;13(34):10029–47. doi: 10.1039/d2sc01863b (PMC9430493; doi:10.1039/d2sc01863b)
Supplement: SC-013-D2SC01863B-s001 [file SC-013-D2SC01863B-s001.pdf]

Electronic Supplementary Material (ESI) for Chemical Science.
This journal is © The Royal Society of Chemistry 2022

Supporting Information

Electronic structure analysis of electrochemical CO_2 reduction by iron-porphyrins reveals basic requirements to design catalysts bearing non-innocent ligands

Maxime Tarrago, Shengfa Ye*[,†,§] and Frank Neese*[,†]

[†]Max-Planck-Institut für Kohlenforschung, Kaiser-Wilhelm-Platz 1, D-45470 Mülheim an der Ruhr, Germany

[‡]Max-Planck-Institut für Chemische Energiekonversion, Stiftstr. 34-36, D-45470 Mülheim an der Ruhr, Germany

[§]State Key Laboratory of Catalysis, Dalian Institute of Chemical Physics, Chinese Academy of Sciences, Dalian 116023, China

1) *Catalytic performances and redox potentials of the mentioned catalytic systems*..........7
Table S1..........7
2) *Relaxed surface scans*..........8
Figure S1..........8
Figure S2..........9
3) *Alternative reactivity*..........10
Figure S3..........10
a) **Spin crossovers**..........10
Figure S4..........12
Table S2..........12
Figure S5..........14
b) **HER/HCOOH formation**..........15
Figure S6..........16
Figure S7..........17
4) *Calculation of the turnover frequency for* $^{1}1^{2-}$..........17
5) *Electronic structure of the additonnal intermediates*..........20
a) **The transition states electronic structures**..........20
Figure S8..........20
b) **Intermediates in the reduction of CO_2 catalyzed with $[Fe(qpy)]^{+}$**..........21
Table S3..........21
Figure S9..........23
c) **Intermediates in the reduction of CO2 catalyzed with $[Fe(bpy^{NHEt}PY2Me)]$**..........23
Table S4..........24
Figure S10..........25
d) **Intermediates in the reduction of CO_2 catalyzed with [Co(qpy)]**..........26
Table S5..........26
Figure S11..........28
e) **Intermediates in the reduction of CO_2 catalyzed with [Co(L)]**..........29
Table S6..........29
Figure S12..........31
f) **Intermediates in the reduction of CO_2 catalyzed with $[Mn (bpy)(CO)_3]K$**..........32
Figure S13..........34
g) **Intermediates in the reduction of CO2 catalyzed with $[Cr(bpy)(CO)3]^{2-}$**..........34
Table S7..........34

Figure S14 36

h) Intermediates in the reduction of CO_2 catalyzed with [Ru(bpy)$_2$(CO)] 37

Table S8..37

Figure S15 38

i) Intermediates in the reduction of CO_2 catalyzed with [Ru(bpy)(tpy)] 39

Table S9..39

Figure S16 41

6) Energy of the calculated structures..42

Table S10..42

7) Cartesian coordinates and imaginary normal modes of vibrations for the intermediates and transition states involved in CO_2 reduction and H_2 generation by 1^{2-}............................43

a) ^{3}1 43

b) $^{2}1^{-}$ 46

c) $^{1}1^{2-}$..48

d) $^{3}1^{2-}$..51

e) $^{5}1^{2-}$..53

f) ^{1}TS1 56

Figure S17 56

g) ^{1}A 59

Figure S18 59

h) ^{3}A 62

i) ^{5}A 64

j) ^{1}B 67

k) ^{1}C 70

Figure S19 70

l) ^{1}TS2 74

Figure S20 74

m) ^{1}TS3..77

Figure S21 78

n) ^{1}D 81

o) ^{3}D 83

p) ^{5}D **86**

q) ^{1}TS5 .... **89**

r) ^{1}E **92**

Figure S23 92

s) ^{1}TS4 .... **95**

Figure S24 96

t) ^{1}F **99**

u) ^{3}F **101**

Figure S25 102

v) ^{5}F **104**

w) ^{1}TS6 .... **107**

Figure S26 107

x) ^{1}M **110**

y) ^{3}M **113**

z) ^{5}M **115**

aa) ^{1}N **118**

bb) ^{3}N **120**

cc) ^{5}N **123**

8) Cartesian coordinates of the other compounds investigated in the studies 126

a) $^24^0$ **126**

b) ^{2}G **127**

c) ^{2}H **129**

d) ^{2}I **130**

e) ^{17}-K^+ **131**

f) ^{1}J **132**

g) ^{1}K **133**

h) ^{2}L **134**

i) $^22^+$ **135**

j) $^2[Fe(qpy)(CO_2)]^+$ **137**

k) ^{1}[Fe(qpy)(CO_2)] 138
l) ^{1}[Fe(qpy)(CO_2H)]$^{+}$ 139
m) ^{1}[Fe(qpy)(CO]$^{2+}$ 141
n) ^{3}3^{0} 142
o) ^{3}[Fe(bpy^{NHEt}PY2Me)(CO_2)] 144
p) ^{3}[Fe(bpy^{NHEt}PY2Me)(CO_2H)]$^{+}$ 146
q) ^{3}[Fe(bpy^{NHEt}PY2Me)(CO)]$^{2+}$ 147
r) ^{2}5^{0} 149
s) ^{2}[Co(qpy)(CO_2)] 150
t) ^{2}[Co(qpy)(CO_2H)]$^{+}$ 152
u) ^{2}[Co(qpy)(CO)]$^{2+}$ 153
v) ^{2}6^{0} 155
w) ^{2}[Co(L)(CO_2)] 156
x) ^{2}[Co(L)(CO_2H)]$^{+}$ 158
y) ^{2}[Co(L)(CO)]$^{2+}$ 159
z) ^{1}8^{-} 161
aa) ^{1}[Mn(bpy)(CO)$_3$(CO_2)]K 161
bb) ^{1}[Mn(bpy)(CO)$_3$(CO_2H)]K^{+} 163
cc) ^{2}[Mn(bpy)(CO)$_3$(CO)] 164
dd) ^{1}9^{2-} 165
ee) ^{1}[Cr(bpy)(CO)$_3$(CO_2)]$^{2-}$ 165
ff) ^{1}[Cr(bpy)(CO)$_3$(CO_2H)]$^{-}$ 166
gg) ^{2}[Cr(bpy)(CO)$_4$]$^{-}$ 167
hh) ^{1}10^{0} 168
ii) ^{1}[Ru(bpy)$_2$(CO)(CO_2)] 170
jj) ^{1}[Ru(bpy)$_2$(CO)(CO_2H)]$^{+}$ 171
kk) ^{1}[Ru(bpy)$_2$(CO)$_2$]$^{2+}$ 173
ll) ^{1}11^{0} 174

mm) $^1[Ru(tpy)(bpy)(CO_2)]$ 176

nn) $^1[Ru(tpy)(bpy)(CO)]^{2+}$ 178

9) Bibliography..179

1) Catalytic performances and redox potentials of the mentioned catalytic systems

Table S1: Reported catalytic performances of the catalytic systems in Scheme 1. The electrolysis potential corresponds to the potential at which the Turnover frequency (TOF, calculated according to several different methods, see below) or turnover number (TON, defined as $n_{product}/n_{Catalyst}$) is measured. In cases where the TOF_{max} is obtained from foot-of-the-wave analysis, or in cases where neither TON nor TOF are measured, the potential corresponds to that of the peak of the CV catalytic wave. The turnover frequencies are given in s^{-1} The Faradaic efficiency (FE) is defined as the proportion of coulomb passing through the electrode participating in the CO2-to-CO conversion reaction. Non-disclosed values are signalled by the initials “N.D.”.

Species	Electrolysis potential (V, vs SCE)	TOF (s^{-1})[a)]	TON/CPE time	FE	Reference
[Fe(TPP)]	-1.64 in DMF	>10^4 (FOWA)	ND	~100%	[1]
[Co(N_4H)]	-1.7 in MeCN	N.D.	4.1/0.7h	45%	[2]
[Cr(bpy)$(CO)_3$]	-2.8 vs Fc/Fc^+ in NMP	N. D.	N.D.	N. D.	[3]
[Mn(R-bpy)$(CO)_3$][b)]	-1.8 in MeCN	5000 (CV)	4/1.2h	98%	[4]
[Re(R-bpy)$(CO)_3$][c)]	-1.7 in MeCN	>6206 (CV)	N.D.	96%	[5]
[Co(L)]	-1.5 in DMF	N. D.	N.D.	82%	[6]
[Ru$(bpy)_2$(CO)]	-1.5 in H_2O	N.D.	26.2/N.D.	51%	[7]
[Ru(bpy)(tpy)]	-1.5 vs NHE in MeCN	N.D.	5/5h	76%	[8]
[Fe(bpy^{NHEt}PY2Me)]	-1.5 in MeCN	2067 (FOWA)	N.D.	81%	[9]
[Fe(qpy)]	-1.2 in MeCN	N.D.	12/4h	70%	[10]
[Co(qpy)]	-1.3 in MeCN	3.3 x 10^4	64/8h	87%	[10]

a) The method affording the turnover frequency is displayed between brackets: FOWA=Foot-Of-the-Wave-Analysis,[11] CV= Cyclic Voltammetry where the TOF is determined from the current plateau i_c/i_p[12]

b) R= mesityl

c) R=^{t}Bu

2) Relaxed surface scans

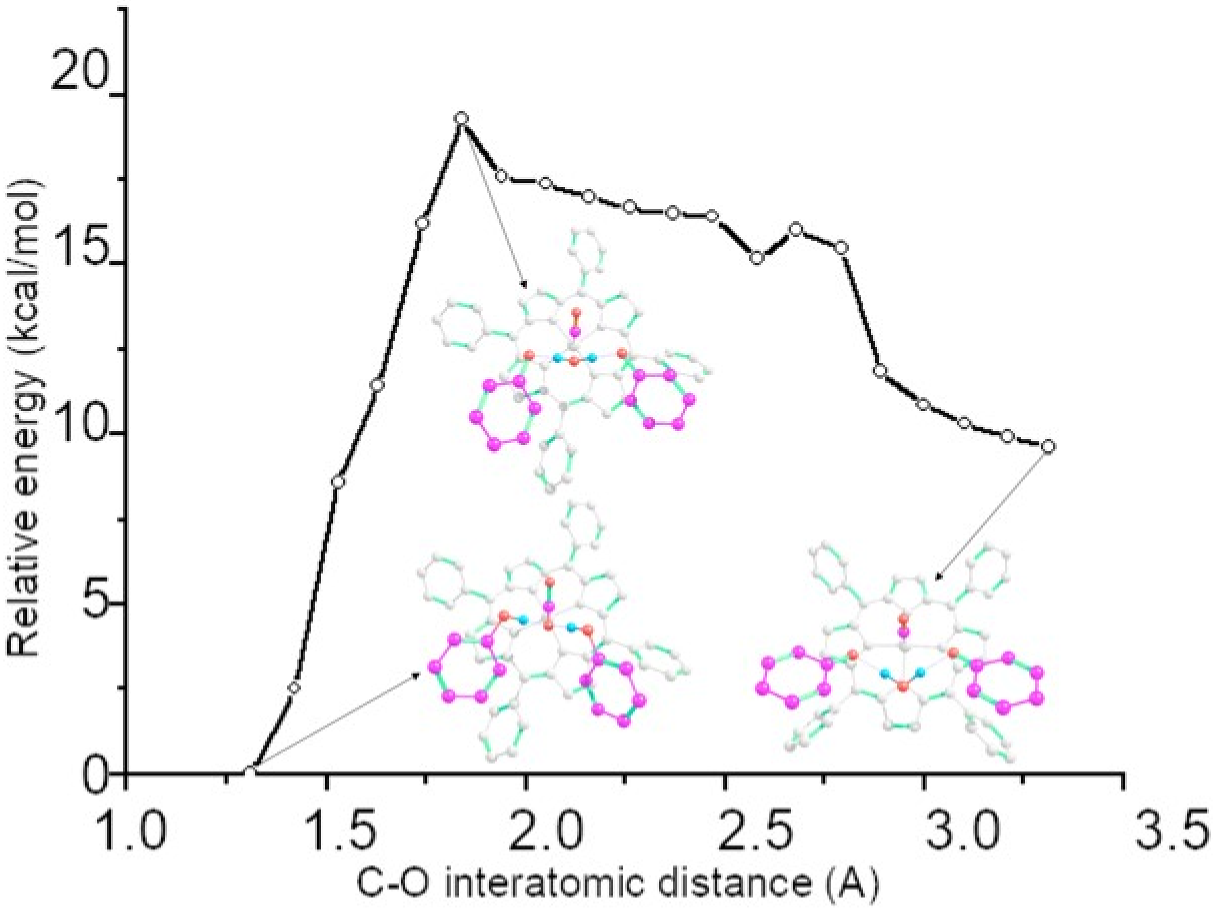

Figure S1:Relaxed surface scan of the C-O interatomic distance of the CO_2 moiety in **^{1}C**. Selected pictures of the structures corresponding to a distance of 1.3 (equilibrium distance in **^{1}C**), 1.8 and 3.3 Å are shown. For clarity, all hydrogens except those belonging to the two transferred protons are hidden, and all atoms except those constituting the two phenols and the CO_2 moiety are greyed out.

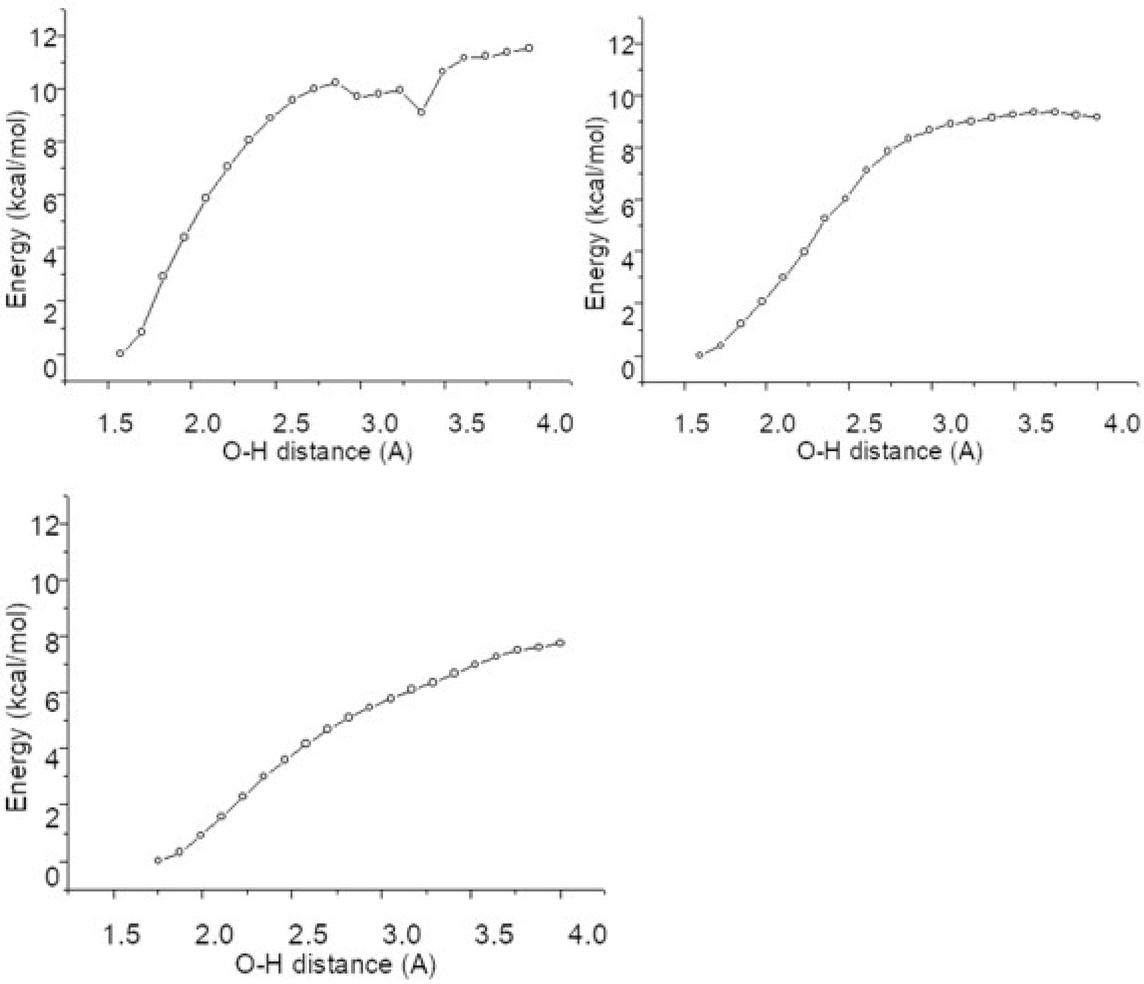

Figure S2: Relaxed electronic energy surface scan of the interatomic distance between the hydrogen of the phenol and one oxygen of the CO_2 moiety in the intermediate ^{1}**B** (Top, left), relaxed electronic energy surface scan of the interatomic distance between the hydrogen of one phenol and the oxygen of the CO_2 moiety in the intermediate ^{1}**C** (Top, right), relaxed electronic energy surface scan of the interatomic distance between the hydrogen of the phenol and the protonated oxygen of the CO_2H moiety in the ^{1}**E** intermediate (bottom). Energies are given relative to the lowest point on the surface for each case.

3) Alternative reactivity

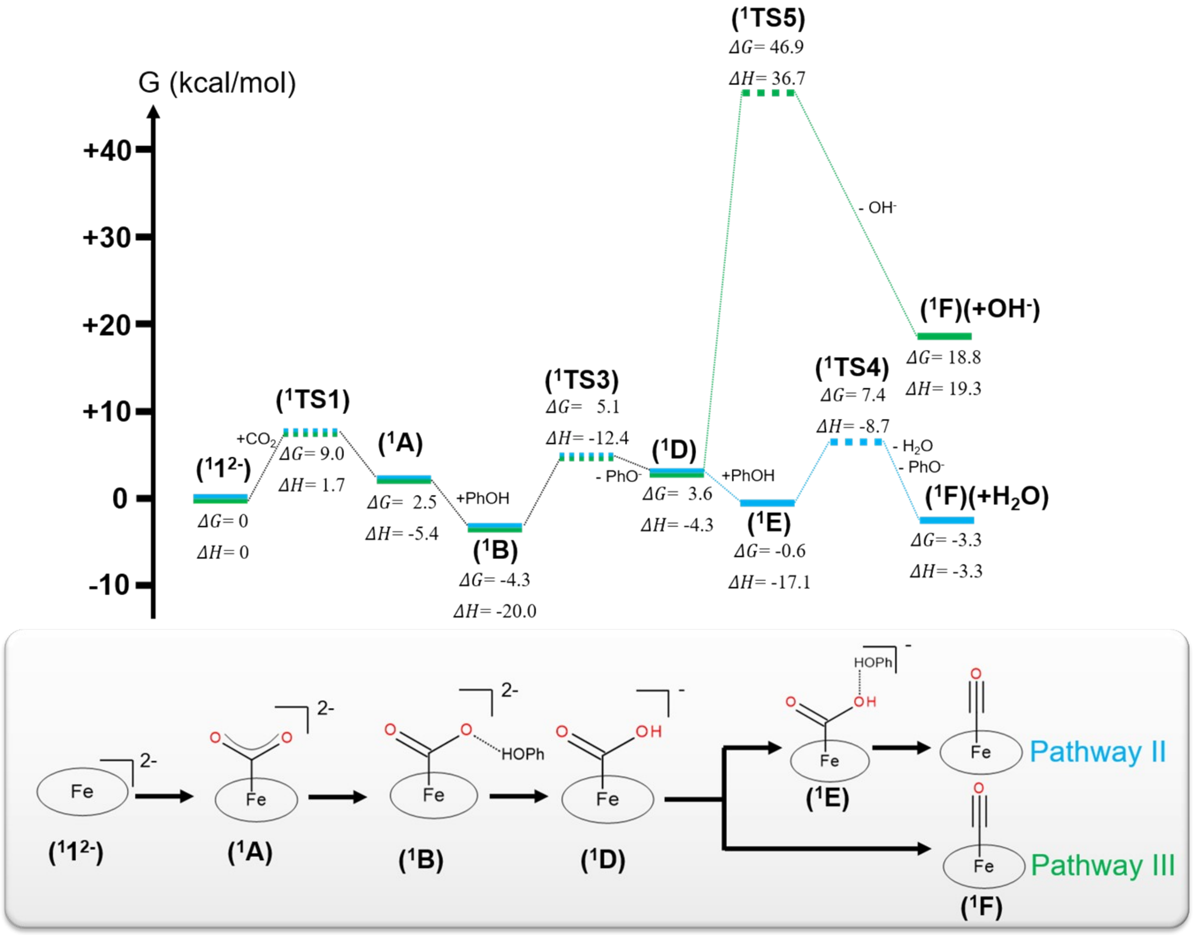

Figure S3: (Top) Energy landscape of the intermediates and transition states involved in the CO_2-to-CO reduction according to pathways II and III. Intermediates are displayed by solid lines and transition states are displayed by dashed lines. Intermediates belonging to pathway II are represented by blue lines and those selectively belonging to pathway III are represented by green lines. The Gibbs free energy changes and enthalpy changes were computed with respect to intermediates $^1\mathbf{1}^{2-}$ and all reactants infinitely separated in DMF. (Bottom) Schematic representation of the intermediates for each pathway, shown by order of formation in pathway II (blue) and pathway III (green).

a) Spin crossovers

Experimental data conclude the molecular catalyst is an overall singlet in its ground state.[13] In the main text, we focus on the singlet state for all intermediates. However, the triplet and

singlet states of [Fe(TPP)] are respectively found 5.7 kcal/mol and 12.1 kcal/mol above the singlet state by our DFT calculations. Such low energy separations suggest that a spin crossover might be possible during the reaction. Because none of the intermediates have been isolated experimentally, the possibility of a spin crossover must be investigated by DFT calculations. However, as seen in Table S2, the free energy of the intermediates in both triplets and quintet pathways are significantly unfavorable compared to the intermediates of the singlet pathway. In the triplet pathway, the formation of the metal-CO_2 adduct is energetically neutral, a behavior undistinguishable from that of the singlet pathway within the typical error range of DFT. On the other hand, the formation of the metallacarboxylic acid, unlike for the singlet pathway, is highly endergonic. In fact, it is about 12.3 kcal/mol higher than the corresponding singlet intermediate (Table S2). The cleavage of the C-O bond step yields a triplet metal-carbonyl adduct that is about 5.1 kcal/mol higher in energy than its singlet counterpart. In the quintet pathway, the formation of the metal-CO_2 adduct was found unstable, and the adduct spontaneously dissociated upon geometry optimization. A relaxed surface scan of the Fe-C bond (Figure S4) confirmed that the adduct is unstable.

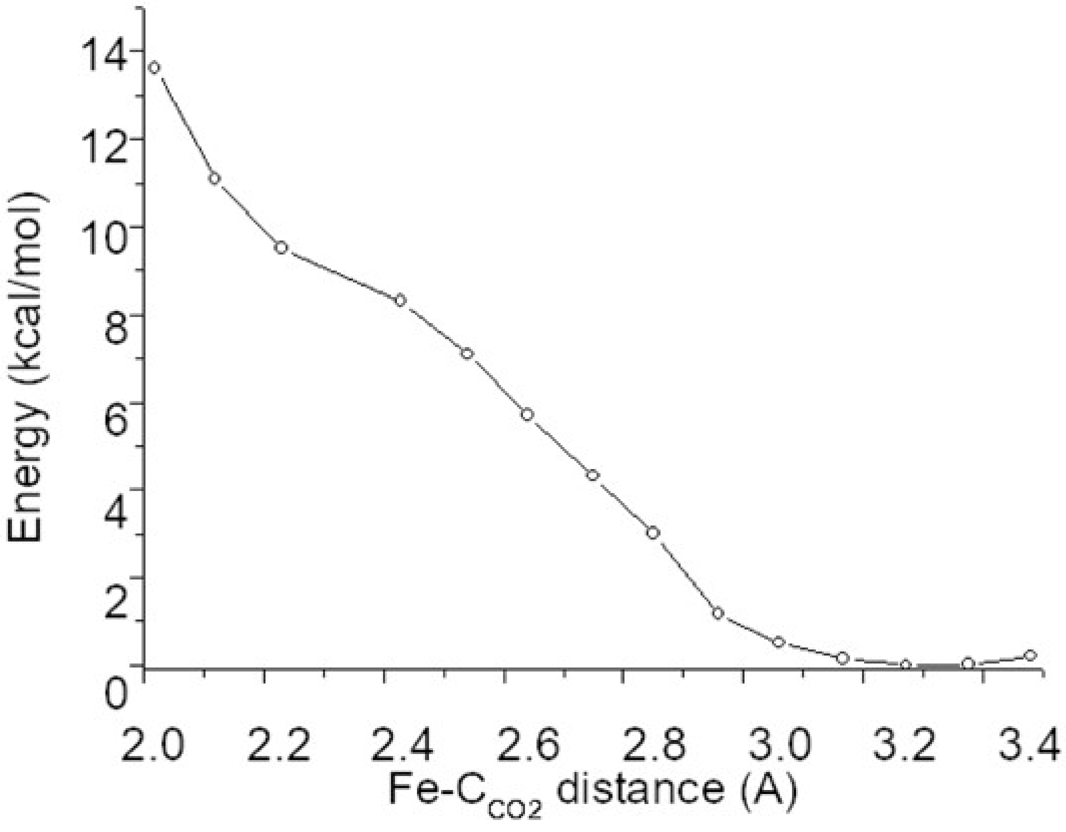

Figure S4: Evolution of the electronic energy of the quintet intermediate $^5[Fe(TPP)(CO_2)]^{2-}$ with the relaxed surface scan of the Fe-C_{CO2} interatomic distance. Relative energies are given with respect to the lowest point on the surface.

Once protonated, the adduct remained stable, but its free energy was found lying 18.1 kcal/mol higher than the singlet metallacarboxylic acid. Finally, after the C-O bond cleavage step, the quintet metal-carbonyl complex lies 7.0 kcal/mol above the corresponding singlet intermediate. Hence, key-intermediates involved in the triplet and quintet pathways are strongly disfavored thermodynamically, rendering spin crossover unlikely, and establishing the singlet pathway as the most favorable. Overall, the significant differences in free energy have an enthalpic origin (Table S10), and are likely to be explained on the basis of differences in electronic structure. In fact, these differences can be attributed to the non-innocence of the ligand, as rationalized in the next paragraphs.

Table S2: Relative energy of the intermediates in the CO_2 to CO conversion reaction (pathway II) in kcal/mol. The chosen reference corresponds to all the reactive infinitely separated in DMF ([Fe(TPP)] in singlet state). The mention “Unstable” accounts for the fact that the adduct dissociates spontaneously into $^{5}\mathbf{1}^{2-}$ and CO_2.

Intermediate	S=0	S=1	S=2
$\mathbf{1}^{2-}$	0	+5.7	+12.1
**A**	+2.5	+5.5	Unstable
**D**	+3.2	+15.5	+21.3
**F**	-3.3	+1.8	+3.7

The differences in energy between the singlet, triplet and quintet intermediates can be rationalized in the basis of the different electronic structures of $^{1}\mathbf{1}^{2-}$, $^{3}\mathbf{1}^{2-}$ and $^{5}\mathbf{1}^{2-}$. The singlet state $^{1}\mathbf{1}^{2-}$ contains an intermediate spin ferrous center interacting *antiferromagnetically* with a porphyrin di-radical tetra-anion. $^{3}\mathbf{1}^{2-}$ also contains an intermediate spin ferrous center but interacting with a singlet diradical TPP tetra-anion with one α electron and one β electron occupying its $1e_g$ orbitals. $^{5}\mathbf{1}^{2-}$ contains an intermediate spin ferrous center too, but interacting *ferromagnetically* with a S=1 diradical TPP tetra-anion with one α electron occupying each of its $1e_g$ orbitals (Figure S5). Consequently, in the triplet and quintet pathways, either one or two electron transfers from the ligand-based $1e_g$ orbitals to the metal-centered $d_{xz,yz}$ orbitals are spin-

forbidden. Consequently, the chemical steps involving such transfers in the singlet pathway cannot involve equivalent transfers on higher spin state surfaces. Instead, either the metal center acquires a higher oxidation state, which hinders its donating ability, or ligand-to-metal electron transfers occur via populating highly antibonding orbitals, which comes at an energetic cost (Figure S5). The electronic structure evolution on the triplet and quintet surfaces are now described in details and compared to the corresponding intermediates on the singlet surface. Such an analysis illustrates the crucial importance of ligand-to-metal electron transfer in the reactivity.

In the triplet pathway, the formation of the adduct ^{3}**A** is almost iso-energetic to the corresponding step in the singlet pathway because the ligand-to-metal electron transfer is spin-allowed in both cases (Figure S5). Hence both ^{1}**A** and ^{3}**A** feature Fe(I) centers with similar donating ability towards CO_2. However, the formation of ^{3}**D** is highly unfavored compared to the formation of ^{1}**D** because for the former, the second ligand-to-metal electron transfer is spin-forbidden. Therefore in ^{3}**D** the metal possesses a higher oxidation state than that of ^{1}**D**. Hence, the ^{3}**D** intermediate is destabilized by as much as 12.7 kcal/mol compared to its singlet counterpart ^{1}**D** because the higher oxidation state of the metal center hinders its donating ability towards CO_2. In details, the iron center in ^{3}**D** is best described as a resonant form between a Fe(III) and a Fe(I) center; while in ^{1}**D** the metal is best described as a resonant form between Fe(II) and Fe(0) (Figure S5). Finally, the C-O bond cleavage affording ^{3}**F** triggers a second ligand-to-metal electron transfer. Like in ^{1}**F**, the intermediate may be described as a Fe(II) center bound to a carbonyl ligand. However, unlike for ^{1}**F**, transfer from the TPP-1e_g(x) to the Fe-d_{xz} orbital is spin-forbidden. Instead, the electron is transferred to the antibonding $\sigma_{Fe\text{-}CO2H}^*$ orbital, which energy is somewhat lowered by the C-O bond cleavage, but remains highly antibonding. Consequently, the energy of ^{3}**F** is higher than that of ^{1}**F**.

In the quintet pathway, the ^{5}**A** adduct spontaneously dissociates upon geometry optimization. This can be easily rationalized on the basis of the ligand-to-metal electron transfers being spin-forbidden. Hence, unlike in the singlet and triplet surface, the oxidation state of the metal remains +II as the CO_2 approaches and its donor ability is not sufficient to bind it. The protonated adduct ^{5}**D** is stable but thermodynamically unfavored compared to its singlet counterpart. In fact, the species is best described as a Fe(II) ferromagnetically-coupled to a porphyrin radical, like the triplet ^{3}**D**. However, ^{5}**D** is higher in energy than ^{3}**D**, because the electronic configuration of iron differs from the latter by a promotion of an electron from the non-bonding Fe-d_{yz} orbital to the antibonding $\sigma_{Fe\text{-}CO2H}^*$, which is energetically unfavorable. The

population of $\sigma_{Fe-CO2H}^*$ is unavoidable in ^{5}**D** because both Fe d_{xz} and Fe d_{yz} are unavailable for a ligand-to-metal electron transfer.

The study of the triplet and quintet surface thus examplifies how intramolecular electron transfers from the ligand to the metal are crucial in enhancing the donor ability of the metal, thus stabilizing key-intermediates.

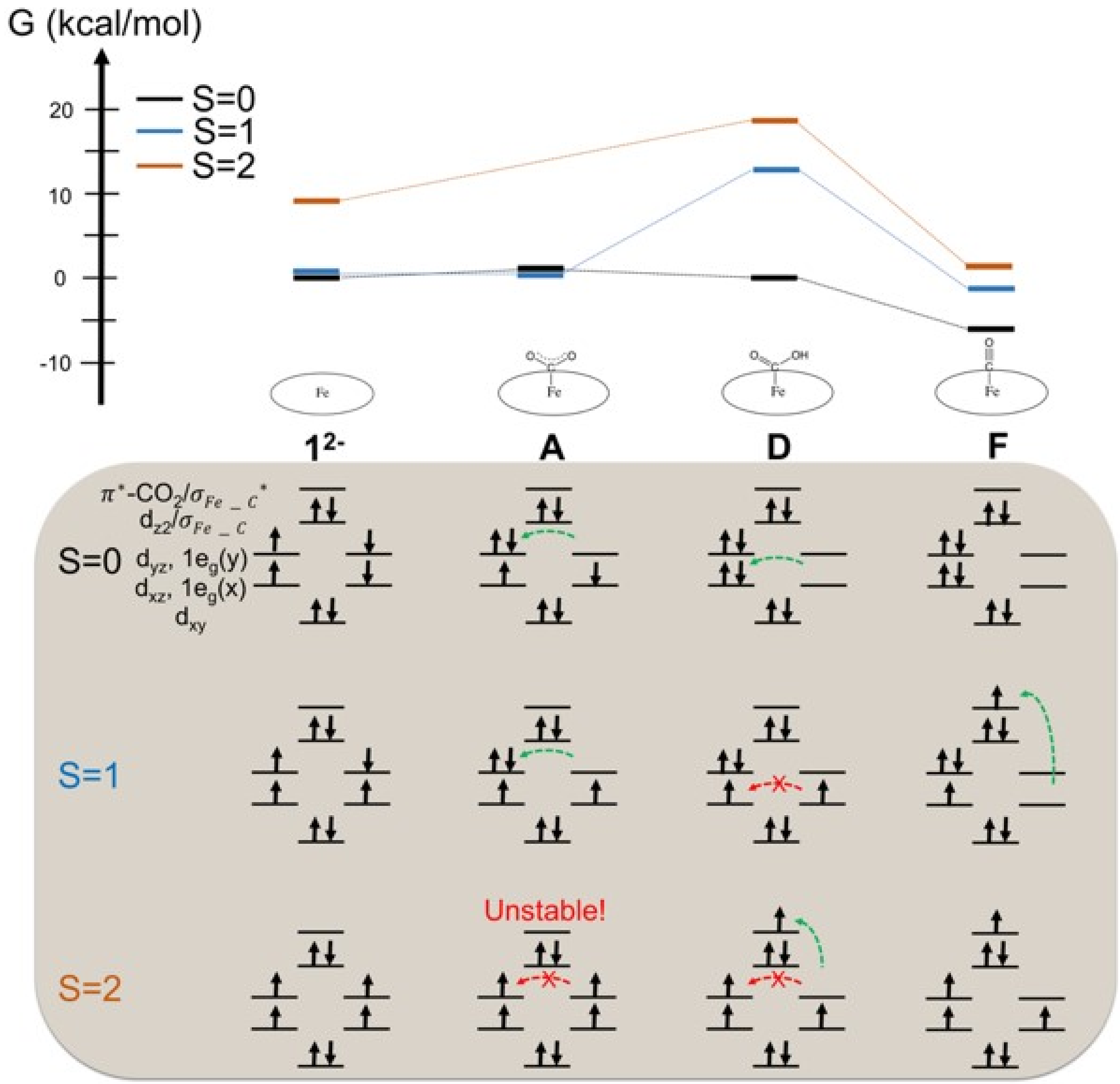

Figure S5: (Top) Energy of key intermediates in the singlet (black), triplet (blue) and quintet (brown) free energy landscape for the pathway II. Each line corresponds to the energy of the intermediate schematized below the diagram, i.e. **1**$^{2-}$, **A**, **D** and **F** from left to right. (Bottom) Schematic representation of the electronic structure of the system (d-shell of iron, redox-active orbitals of the TPP ligand, bonding (σ_{Fe-C}) and antibonding (σ_{Fe-C}^*) combination of the Fe-d_{z2} and CO_2-π^* orbitals) in the intermediates **1**$^{2-}$, **A**, **D** and **F**. Each row corresponds to the overall

spin state of the intermediates, i.e. .S=0 (top row), S=1 (intermediate row) or S=2 (bottom row). Of note, in **1**$^{2-}$, the Fe-d_{z2} and CO_2-π^* orbitals do not overlap, hence σ_{Fe-C} and σ_{Fe-C}^* correspond to Fe d_{z2} and CO_2 π^*, respectively. Green dashed arrows represent spin-allowed electron transfers, and red dashed arrows represent spin-forbidden electron transfers. For the electronic structure of the intermediate ^{5}**A**, the mention "Unstable" accounts for the fact that the adduct dissociates spontaneously into ^{5}**1**$^{2-}$ and CO_2.

b) HER/HCOOH formation

As specified in the main text, the most widely accepted mechanism for both hydrogen evolution and formic acid generation with mononuclear transition metals start with the protonation of the metal center. In the case of ^{1}**1**$^{2-}$, this step (yielding the metal-hydride ^{1}**M**) is significantly endergonic (ΔG = +8.6 kcal/mol), and admits a rather large kinetic barrier (^{1}**TS6**, ($\Delta G^{\neq}$ = +19.1 kcal/mol) (Figure S6). Following this step, the reaction may proceed either towards HER or HCOOH formation. The formation of dihydrogen involves the attack of the metal-hydride ^{1}**M** on a second proton to form ^{3}**1** and release dihydrogen. This step is highly exergonic (ΔG = -18.7 kcal/mol), both because of a favorable enthalpy variation, and a favorable entropic effect due to the release of the H_2 molecule. Alternatively, in the mechanism of formic acid generation a CO_2 molecule inserts into the Fe-H bond of ^{1}**M** to form a Fe-OCHO adduct ^{5}**N**. Our calculation show that this step is extremely favorable (ΔG = -33.1 kcal/mol) despite an unfavorable entropy variation following the association of CO_2 and ^{1}**M**. An alternative pathway for HCOOH generation involves the direct interaction between the reduced metal and CO_2 to form an Fe-OCO adduct, followed by the protonation of the exposed carbon to form ^{5}**N**. However, we could not find a stable geometry for the Fe-OCO adduct, which dissociates upon geometry optimization into $[Fe(TPP)]^{2-}$ and CO_2, showing this alternative pathway is not a viable source.

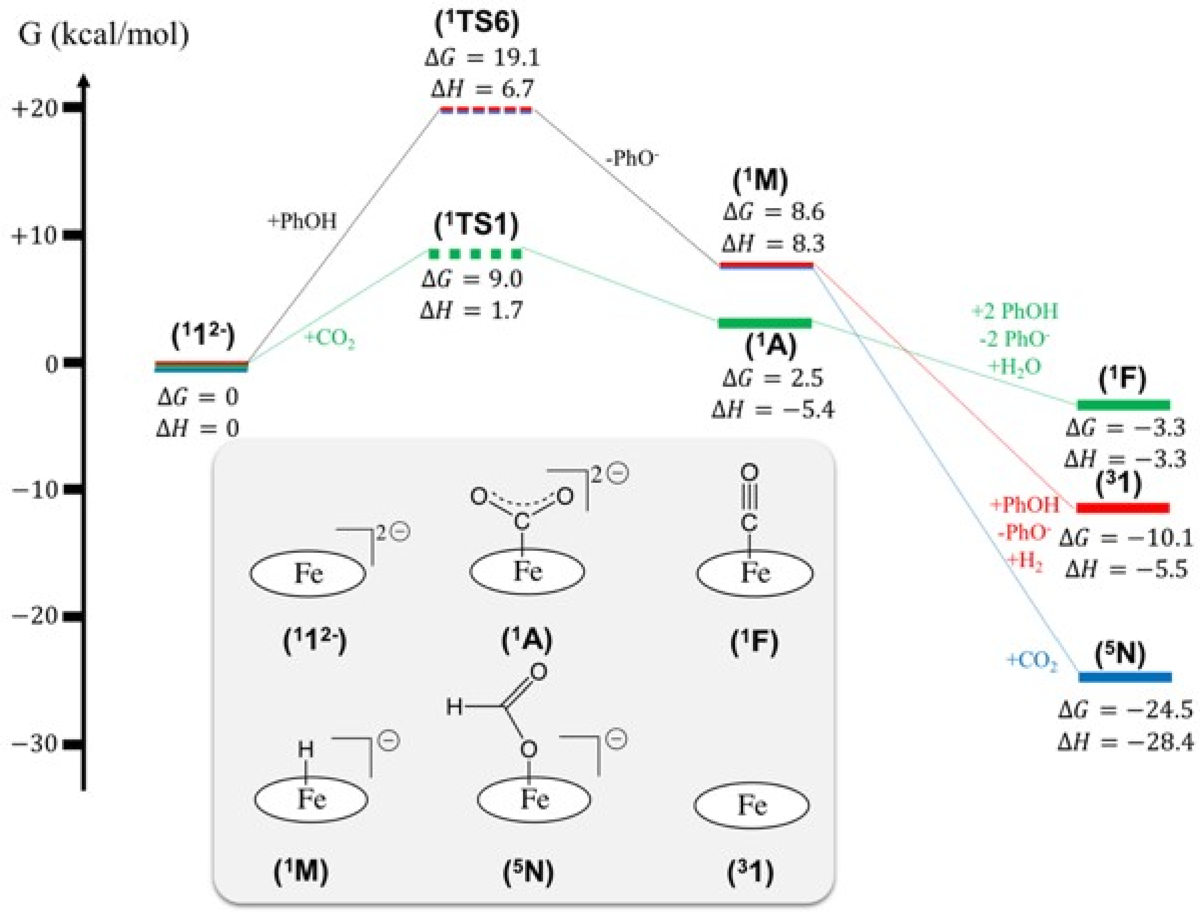

Figure S6: Free energy landscape of key-intermediates and transition states involved in the CO_2-to-CO conversion reaction (pathway II, green lines), hydrogen evolution (red lines) and HCOOH generation (blue lines). Intermediates are displayed in solid lines and transition states are displayed in dashed lines. The Gibbs free energy variation and the gas-phase enthalpy variation both defined in Eq. 1 (Main Text) are displayed below each intermediate. The schematic representation of each intermediate is shown below the free energy surface.

The experimental selectivity of the reaction for CO generation over HCOOH and H_2 generation thus mainly originates from kinetic reasons. In fact, the driving force of the two latter reactions are significantly higher than that of CO generation. However, we found that the formation of the metal-hydride admits an impossibly large barrier ($\Delta G^{\neq}$ = +19.1 kcal/mol), which prevents the formation of this key-intermediate. By comparison, the highest predicted barrier for CO generation is only $\Delta G^{\neq}$ = +9.4 kcal/mol. Hence, our calculations predict the formation of HCOOH and H_2 is prohibited mainly for kinetic reason, due to the impossibly high-lying transition state associated with the formation of the metal-hydride adduct. A similar

result was found by Zhang and al. for a related iron-porphyrin system[14] and by Keith and al. for $[Re(bpy)(CO)_3]$.[15]

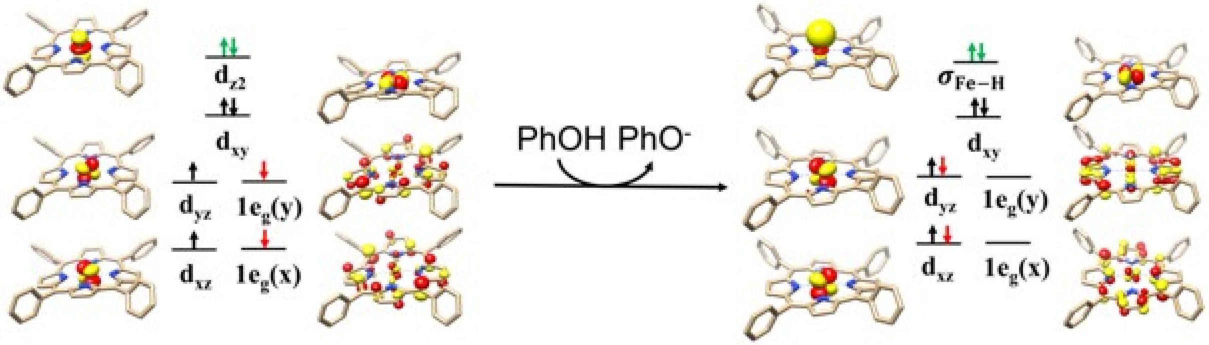

$^{1}\mathbf{1}^{2-}$ $^{1}\mathbf{M}$

Figure S7: Electronic structure of the intermediates $^{1}\mathbf{1}^{2-}$ and $^{1}\mathbf{M}$. Green arrows represent electrons transferred from the metal to the proton during the formation of $^{1}\mathbf{M}$, and red arrows represent electrons transferred from the ligand to the metal.

4) Calculation of the turnover frequency for $^{1}\mathbf{1}^{2-}$

We present here the method used to reproduce the apparent constant experimentally derived by Costentin et. Al.,[11] which can then be used to determine the intrinsic turnover frequency of the system.

In the following treatment, we consider as one elementary step two potential energy surface minima separated by a transition state. This simplifies Pathway I to the following reaction sequence:

$^{2}1^{-} + e^{-} \rightleftharpoons {}^{1}1^{2-}$ k_e, k_{-e} (S1)

$^{1}1^{2-} + CO_2 + PhOH \rightleftharpoons {}^{1}B$ k_1,k_{-1} (S2)

$^{1}B + PhOH \rightarrow {}^{1}F + 2\,PhO^{-} + H_2O$ k_2 (S3)

$^{1}F + {}^{1}1^{2-} \rightarrow 2\,{}^{2}1^{-} + CO$ k_4 (S4)

To fit the situation described by Costentin for electrochemical homogeneous catalysis,[11] it is assumed that reaction (S3) and (S4) are irreversible, and that the reaction cycle is in a steady-state regime, the latter being often assumed in catalysis.[16,17] Under these conditions, the apparent rate constant of the reaction sequence (S2-S3) is directly related to the turnover frequency by the following relationship:[11]

$$TOF = \frac{k_{ap}[CO_2]}{1 + e^{\frac{F}{k_BT}[E - E^0]}}$$ (S5)

Where k_{ap} is the apparent constant of the reaction, F the Faraday constant, k_B the Boltzmann constant and T is the temperature. E is the potential, and E^0 is the standard potential of the couple $^2\mathbf{1}^-/^1\mathbf{1}^{2-}$.

To derive the apparent constant, we make use of the steady-state regime to derive the useful relationship:

$$\frac{d[^1B]}{dt} = 0$$ (S6)

From S6, it follows:

$$k_{ap} = \frac{k_1k_2[PhOH]^2}{k_2[PhOH] + k_{-1}}$$ (S7)

The apparent constant of the reaction can then be calculated at three different phenol concentrations ([PhOH]=0.1 M, 0.75 M and 3 M), using Eyring's law for the calculation of kinetic constants. Following the calculated energy landscape summarized in Figure 1 (Main Text):

$$k_1 = \frac{h}{k_BT} e^{-\frac{9.0\ kcal/mol}{k_BT}}$$ (S8)

$$k_{-1} = \frac{h}{k_BT} e^{-\frac{13.3\ kcal/mol}{k_BT}}$$ (S9)

$$k_2 = \frac{h}{k_BT} e^{-\frac{17.3\ kcal/mol}{k_BT}}$$ (S10)

For three concentrations of phenol ([PhOH]=0.1 M, 0.75 M and 3 M), we obtain k_{ap}= 18.7 $s^{-1}.M^{-1}$, 1.0 x 10^3 $s^{-1}.M^{-1}$, and 1.7 x 10^4 $s^{-1}.M^{-1}$. Maximum turnover frequencies may be obtain from Eq. S6 ($|E| >> |E^0|$) for a CO_2 concentration of 0.23 mol/L,[11] we obtain TOF_{max}=4.3 s^{-1}, 2.4 x 10^2 s^{-1} and 3.9 x 10^3 s^{-1}.

Likewise, the turnover frequencies can be estimated for Pathway II. This time the sequence corresponds to:

$$^21^- + e^- \rightleftharpoons {}^11^{2-}$$ k_e, k_{-e} (S11)

$$^11^{2-} + CO_2 + PhOH \rightleftharpoons {}^1B$$ k_1,k_{-1} (S12)

$$^1B + PhOH \rightleftharpoons {}^1E + PhO^-$$ k_2,k_{-2} (S13)

$$^1E \rightarrow {}^1F + PhO^- + H_2O$$ k_3 (S14)

$$^1F + {}^11^{2-} \rightarrow 2\,{}^21^- + CO$$ k_4 (S15)

To fit the situation described by Costentin for electrochemical homogeneous catalysis, it is assumed that reaction (S14) and (S15) are irreversible, and that the reaction cycle is in a steady-state regime.

To derive the apparent constant, we make use of the steady-state regime of the reaction to derive the following useful relationships:

$$\frac{d[^1E]}{dt} = 0 \tag{S16}$$

$$\frac{d[^1B]}{dt} = 0 \tag{S17}$$

From (S16) and (S17), it follows:

$$[^1B] = \frac{k_1[^11^{2-}][CO_2][PhOH]}{k_2[PhOH] + k_{-1} - \dfrac{k_{-2}k_2[PhOH][PhO^-]}{k_3 + k_{-2}[PhOH]}}$$

(S18)

$$[^1E] = \frac{k_2k_1[^11^{2-}][CO_2][PhOH]^2}{\left(k_2[PhOH] + k_{-1} - \dfrac{k_{-2}k_2[PhOH][PhO^-]}{k_3 + k_{-2}[PhOH]}\right)(k_3 + k_{-2}[PhO^-])}$$

(S19)

Finally, the reaction rate can be written as:

$$v = k_3[^1E] = \frac{k_3k_2k_1[^11^{2-}][CO_2][PhOH]^2}{\left(k_2[PhOH] + k_{-1} - \dfrac{k_{-2}k_2[PhOH][PhO^-]}{k_3 + k_{-2}[PhOH]}\right)(k_3 + k_{-2}[PhO^-])}$$

(S20)

By identification, the apparent constant as defined by Costentin et. al.[1,11] is:

$$k_{ap} = \frac{k_3k_2k_1[PhOH]^2}{\left(k_2[PhOH] + k_{-1} - \dfrac{k_{-2}k_2[PhOH][PhO^-]}{k_3 + k_{-2}[PhOH]}\right)(k_3 + k_{-2}[PhO^-])}$$

(S21)

The apparent constant of the reaction can then be calculated at three different phenol concentrations ([PhOH]=0.1 M, 0.75 M and 3 M), using Eyring's law for the calculation of

kinetic constants and assuming $[PhO^-]\sim 0$. Following the calculated energy landscape summarized in Figure 1 (Main Text):

$$k_1 = \frac{h}{k_B T} e^{-\frac{9.0\ kcal/mol}{k_B T}} \tag{S22}$$

$$k_{-1} = \frac{h}{k_B T} e^{-\frac{13.3\ kcal/mol}{k_B T}} \tag{S23}$$

$$k_2 = \frac{h}{k_B T} e^{-\frac{9.4\ kcal/mol}{k_B T}} \tag{S24}$$

$$k_{-2} = \frac{h}{k_B T} e^{-\frac{5.7\ kcal/mol}{k_B T}} \tag{S25}$$

$$k_3 = \frac{h}{k_B T} e^{-\frac{8.0\ kcal/mol}{k_B T}} \tag{S26}$$

For three concentrations of phenol ([PhOH]=0.1 M, 0.75 M and 3 M), we obtain k_{ap}=1.6x10^5 s^{-1}.M^{-1}, 1.2x10^6 s^{-1}.M^{-1} and 4.8x10^6 s^{-1}.M^{-1}. Maximum turnover frequencies may be obtain from Eq. S6 (|E| >> |E^0|) for a CO_2 concentration of 0.23 mol/L,[11] we obtain TOF_{max}= 3.6x10^4 s^{-1}, 2.76x10^5 s^{-1} and 1.10x10^6 s^{-1}, a remarkable agreement with the experimentally-resolved values of 1.8x10^3 s^{-1}, 1.5x10^4 s^{-1} and 1x10^5 s^{-1}.[11]

5) Electronic structure of the additonnal intermediates

a) The transition states electronic structures

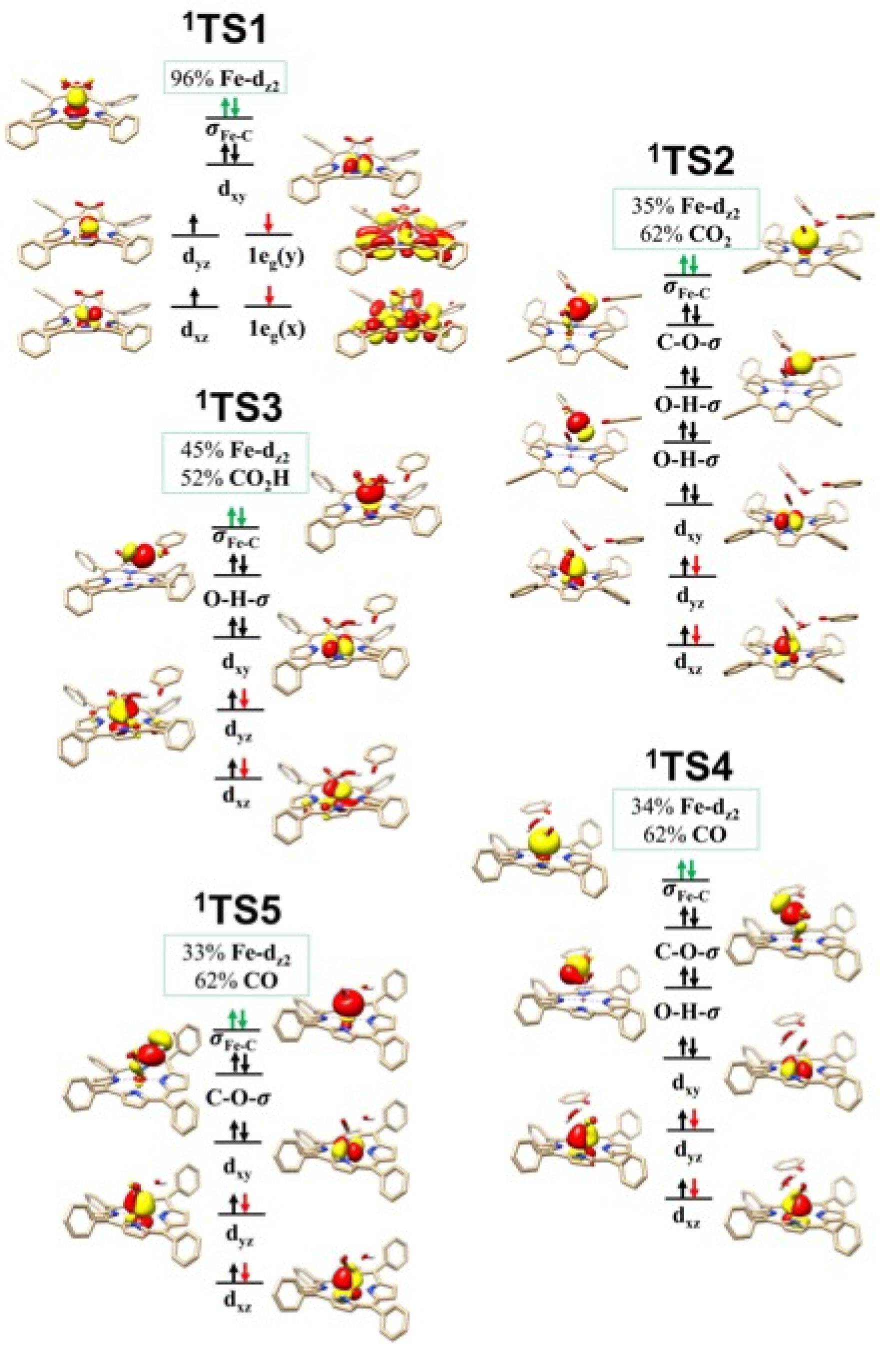

Figure S8: Electronic structure of the transition states **^{1}TS1**, **^{1}TS2**, **^{1}TS3**, **^{1}TS4** and **^{1}TS5** defined in the Main text and in Figure S3. Electrons involved in the TPP-to-Fe electron transfers are represented by red arrows. Electrons involved in the metal-to-CO_2 are shown by green arrows. The Fe and CO_2/CO_2H/CO Löwdin population of the $\sigma_{Fe\text{-}C}$ bonding orbital is displayed circled in green. Carbons are displayed in beige, oxygen in red and iron in orange. For clarity, all hydrogens are hidden except the hydrogen of the CO_2H moiety and the hydrogen of the phenols in the process of being transferred to the reaction catalyst.

b) Intermediates in the reduction of CO_2 catalyzed with $[Fe(qpy)]^+$

Table S3: Electronic energy difference (in kcal/mol) between the spin states of each intermediate involved in the mechanism of CO_2 reduction catalysed with $[Fe(qpy)]^+$. For each line, the energy is given relative to the lowest-lying spin state of the intermediate. Notably, the mention "dissociates" signals that the intermediate $[Fe(qpy)(CO_2)]^+$ spontaneously dissociates into $[Fe(qpy)]^+$ and CO_2 upon geometry optimization.

Intermediate	Low-spin	Intermediate spin	High spin
$[Fe(qpy)]^+$	0 (S=1/2)	19.1(S=3/2)	6.8 (S=5/2)
$[Fe(qpy)(CO_2)]^+$	0 (S=1/2)	Dissociates (S=3/2)	Dissociates (S=5/2)
$[Fe(qpy)(CO_2)]$	0 (S=0)	3.1 (S=1)	4.6 (S=2)
$[Fe(qpy)(CO_2H)]^+$	0 (S=0)	6.2 (S=1)	1.6 (S=2)
$[Fe(qpy)(CO)]^{2+}$	0(S=0)	4.0 (S=1)	<1

The active species, $[Fe(qpy)]^+$, features a distorted square-planar environment. It is best described as a Fe^{II} center (S_{Fe}=1) antiferromagnetically coupled to a $qpy^{\bullet-}$ ligand (S_{qpy}=1/2). In details, the Fe center features a configuration $(d_{xy})^2(d_{xz})^1(d_{yz})^1(d_{z2})^2$, one β-electron populates the primarily ligand-based $1\pi^*$ orbital, which is an admixture of the ligand-centered π_{qpy}^* and Fe d_{yz} atomic orbital. Consequently the orbitals Fe d_{yz} and $1\pi^*$ form a spin-coupled pair. In the next step, the doubly-occupied Fe d_{z2} donates electrons to the unoccupied in-plane CO_2 π_{ip}^* orbital. The resulting bonding orbital ($\sigma_{Fe\text{-}C}$) remains mainly centered around Fe (81% Fe, 16% CO_2). Hence the system is best described as a Fe^I center (S_{Fe}=1/2) bound to a neutral CO_2 ligand. Interestingly, the intramolecular electron transfer connects the π_{qpy}^* to the Fe d_{xz} orbital rather than the Fe d_{yz}, even though in $[Fe(qpy)]^+$, the π_{qpy}^* is mixed with the Fe d_{yz}. In fact, concomitantly with the intramolecular electron transfer from $1\pi^*$ to Fe d_{yz}, a promotion from the Fe d_{yz} to the Fe d_{xz} is triggered because of the interelectronic repulsion between the electrons in the Fe d_{yz} orbital and those of the C-O bond. Hence, in total, the electron is transferred from the π_{qpy}^* to the Fe d_{xz}, even though these orbitals do not mix. In the subsequent step, an electron is transferred from the electrode to the now vacant $1\pi^*$ orbital. The reduction takes place on the ligand, hence the polarity of the Fe-C_{CO2} bond is only marginally affected and the weight of Fe d_{z2} orbital in the $\sigma_{Fe\text{-}C}$ orbital remains predominant (70%). Hence the system is best described as a Fe^I center (S_{Fe}=1/2) antiferromagnetically-coupled with a radical quaterpyridine ligand (S_{qpy}=1/2) and bound to an approximately neutral CO_2 ligand. The subsequent step is the protonation of the CO_2 moiety affording the metallacarboxylic acid intermediate, which triggers

a significant change in the electronic structure. The Fe-C_{CO2} bond becomes more covalent, as indicated by the weight of Fe in the σ_{Fe-C} orbital (40% Fe, 56% CO_2H). Hence this step can be interpreted as a concerted proton electron transfer (CPET) to the CO_2. Meanwhile, a second electron transfer from the π_{qpy}^* orbital to the Fe d_{yz} compensates the loss of electronic density due to the metal-to-CO_2 transfer. The complex has to be described as a resonant form between a singlet Fe^0 bound to a $(CO_2H)^+$ ligand and a singlet Fe^{II} bound to a $(CO_2H)^-$ ligand, bound to a charge neutral quaterpyridine ligand. In total, this step thus corresponds to the transfer of one electron from the metal to the CO_2 ligand, and one electron from the quaterpyridine ligand to the metal center. The next step corresponds to the C-O bond cleavage and yields a metal-carbonyl. The σ_{Fe-CO} bonding orbital is mainly centered around the CO (35% Fe, 61% CO), hence the system is best described as a Fe^{II} center (S_{Fe}=0) bound to a carbonyl ligand (and a neutral quaterpyridine).

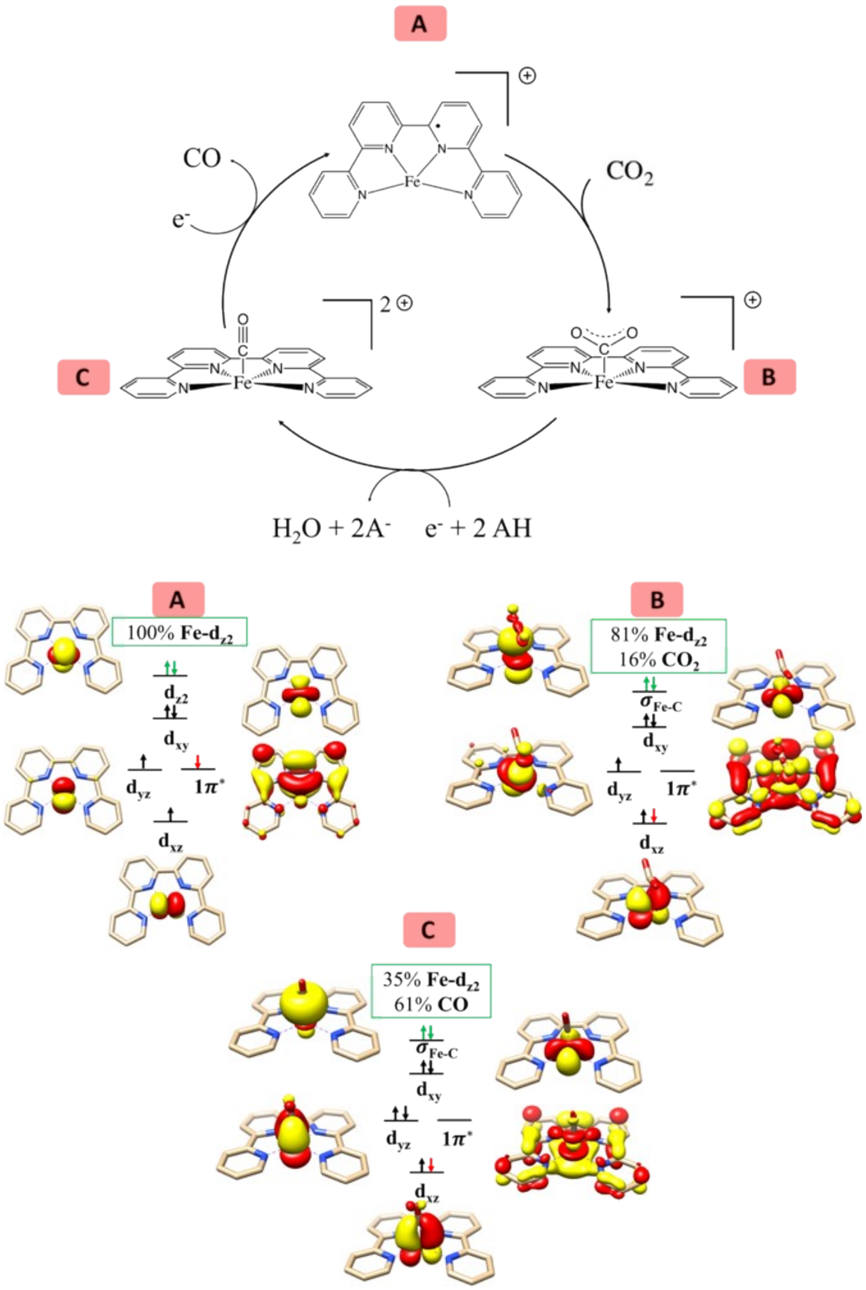

Figure S9: (Top) catalytic cycle of CO_2-to-CO conversion catalysed with $[Fe(qpy)]^{+}$. The mechanism is one compatible with ref. 10. (Bottom) associated electronic structure of the intermediates $[Fe(qpy)]^{+}$ (A), $[Fe(qpy)(CO_2)]^{+}$ (B), $[Fe(qpy)(CO_2)]$ (C), $[Fe(qpy)(CO_2H)]^{+}$ (D) and $[Fe(qpy)(CO)]^{2+}$ (E). Electrons involved in the ligand-to-metal intramolecular transfer are shown by red arrows. Electrons involved in the metal-to-CO_2 intramolecular transfer are shown by green arrows. The Fe and CO_2/CO_2H/CO Löwdin population of the $\sigma_{Fe\text{-}C}$ bonding orbital is displayed circled in green. Carbons are displayed in beige, oxygen in red and iron in orange. For clarity, all hydrogens are hidden.

c) Intermediates in the reduction of CO2 catalyzed with $[Fe(bpy^{NHEt}PY2Me)]$

Table S4: Electronic energy difference (in kcal/mol) between the spin states of each intermediate involved in the mechanism of CO_2 reduction catalysed with [Fe(bpy^{NHEt}PY2Me)]. For each line, the energy is given relative to the lowest-lying spin state of the intermediate.

	S=0	S=1	S=2
[Fe(bpy^{NHEt}PY2Me)]	+23.5	0	+5.8
[Fe(bpy^{NHEt}PY2Me)(CO_2)]	+9.7	0	+6.7
[Fe(bpy^{NHEt}PY2Me)(CO_2H)]$^+$	+5.9	0	+5.9
[Fe(bpy^{NHEt}PY2Me)(CO)]$^{2+}$	+5.1	0	+21.4

The active species [Fe(bpy^{NHEt}PY2Me)] corresponds to a Fe^{II} center (S_{Fe}=2) antiferromagnetically coupled to a diradical bpy^{NHEt}PY2Me$^{\bullet\bullet 2-}$ ligand ($S_{bpyNHEtPY2Me}$=1). In details, the Fe features the electronic configuration $(d_{xy})^1(d_{xz})^2(d_{yz})^1(d_{z2})^1(d_{x2-y2})^1$, and two β-electrons populate the $1\pi^*$ and $2\pi^*$. The former is an admixture of the PY2Me π_{PY2Me}^* and Fe d_{xy} fragment orbitals, while the latter is an admixture of bpy π_{bpy}^* and Fe d_{yz} fragment orbitals. Consequently, $1\pi^*$, $2\pi^*$, Fe d_{xy} and Fe d_{yz} form two spin-coupled pairs. Of note, the Fe d_{z2} orbital is singly-occupied. However, the approach of the CO_2 moleule triggers a substantial electronic reorganization which can be summarized as follow: (1) the approach of CO_2 triggers the π_{PY2Me}^*-to-d_{xy} electron transfer, (2) the energy of the $\sigma_{Fe\text{-}C}$ is lowered due to the mixing of the Fe-d_{z2} atomic orbital with the CO_2 π_{ip}^* orbital, triggering a d_{xy}-to-$\sigma_{Fe\text{-}C}$ electron transfer. The $\sigma_{Fe\text{-}C}$ orbital is therefore doubly-occupied with a strong Fe character (74% Fe, 22% CO_2). Hence, the system is best described as a Fe^{I} center (S_{Fe}=3/2) antiferromagnetically-coupled with a bpy^{NHEt}PY2Me$^{\bullet -}$ ligand ($S_{bpyNHEtPY2Me}$=1/2). In terms of electronic transfers, the step corresponds to an electron transfer from the ligand to the metal. The subsequent protonation of the adduct changes drastically the polarity of the bonding orbital $\sigma_{Fe\text{-}C}$ orbital (35% Fe, 65% CO_2H). Furthermore, an electron is transferred from the ligand π_{bpy}^* to the Fe d_{yz} orbital Hence, this species is best described as a Fe^{II} (S_{Fe}=1) metal center bound to a $(CO_2H)^-$ ligand. This step corresponds to a two-electron transfer from the metal to the CO_2, coupled with a one-electron transfer from the ligand to the metal. Finally, the proton-assisted cleavage of the C-O bond yields a metal-carbonyl adduct in which the bonding $\sigma_{Fe\text{-}C}$ is clearly CO-centered (29% Fe, 67% CO). This species is unambiguously described as a Fe^{II} (S_{Fe}=1) metal center bound to a carbonyl ligand.

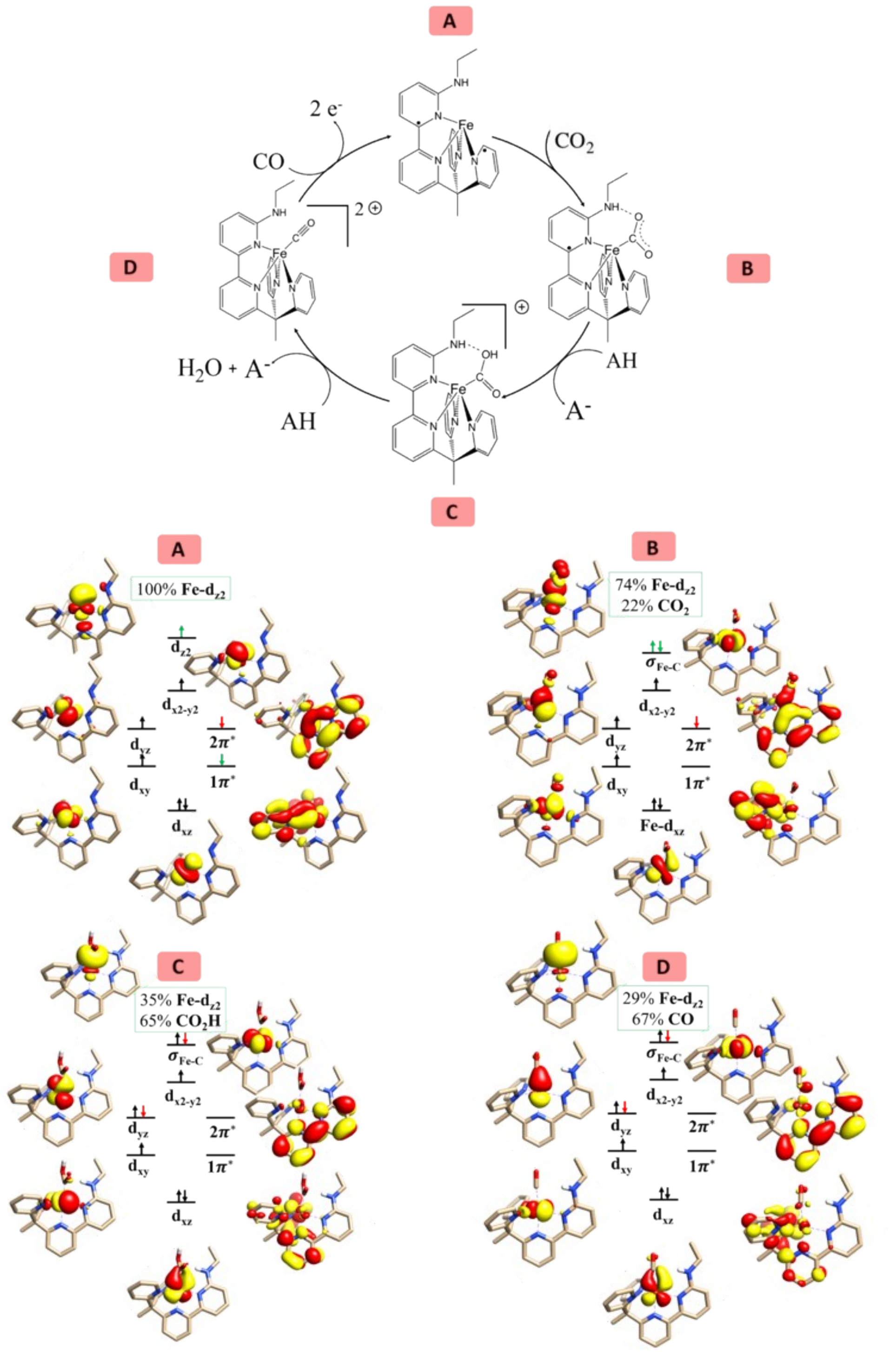

Figure S10: (Top) catalytic cycle of CO_2-to-CO conversion catalysed with [Fe(bpy^{NHEt}PY2Me)]. The mechanism is taken from ref. 18. However, different from the results of the authors, we found that the catalytic system remains in a triplet state for all investigated intermediates (Table S4). (Bottom) associated electronic structure of the intermediates [Fe(bpy^{NHEt}PY2Me)] (A), [Fe(bpy^{NHEt}PY2Me)(CO_2)] (B), [Fe(bpy^{NHEt}PY2Me)(CO_2H)]$^+$ (C),

and [Fe(bpy^{NHEt}PY2Me)(CO)]$^{2+}$ (D). Electrons involved in the ligand-to-metal intramolecular transfer are shown by red arrows. Electrons involved in the metal-to-CO_2 intramolecular transfer are shown by green arrows. The Fe and CO_2/CO_2H/CO Löwdin population of the $\sigma_{Fe\text{-}C}$ bonding orbital is displayed circled in green. Carbons are displayed in beige, oxygen in red and iron in orange. For clarity, all hydrogens are hidden except the hydrogen of the CO_2H motif and the hydrogens participating to the intramolecular hydrogen bond.

d) Intermediates in the reduction of CO_2 catalyzed with [Co(qpy)]

Table S5: Electronic energy difference (in kcal/mol) between the spin states of each intermediate involved in the mechanism of CO_2 reduction catalysed with [Co(qpy)]. For each line, the energy is given relative to the lowest-lying spin state of the intermediate. Notably, the mention “dissociates” signals that the intermediate [Co(qpy)(CO_2)] spontaneously dissociates into [Co(qpy)] and CO_2 upon geometry optimization

	S=1/2	S=3/2	S=5/2
[Co(qpy)]	0	+8.4	+20.9
[Co(qpy)(CO_2)]	Dissociates	0	Dissociates
[Co(qpy)(CO_2H)]$^+$	0	+6.7	+45.7
[Co(qpy)(CO)]$^{2+}$	0	+6.5	+70.5

This mechanism is highly similar to that of [Co(N_4H)]. In the active species [Co(qpy)], the cobalt has a distorted square-planar environment. It is best described as a Co(II) (S_{Co}=1/2) center antiferromagnetically coupled with a qpy$^{\bullet\bullet 2-}$ diradical dianion (S_{qpy}=1). In details, the Co center features a $(d_{xy})^2(d_{xz})^1(d_{yz})^2(d_{z2})^2$ and two electrons populate the primarily ligand-based $1\pi^*$ and $2\pi^*$ orbitals. Of note, the former is purely ligand-centered, while the latter is an admixture of the fragment orbital $\pi_{qpy\text{-}1}{}^*$ and the Co-d_{xz} atomic orbital. Consequently, $2\pi^*$ and d_{xz} form a spin-coupled pair leading to an antiferromagnetic spin coupling between the quaterpyridine and the metal center. In the first step, the Co center binds the CO_2 molecule. However, our calculations showed that the adduct was unstable in a singlet state and spontaneously dissociates into [Co(qpy)] and CO_2. Arguably, this can be attributed to the weak bond between the cobalt center and CO_2 motif, and the unfavorable interelectronic repulsion between the doubly-occupied Co d_{yz} orbital and the electrons of the C-O bond. Indeed, the quartet state, in which one electron of the Co d_{yz} orbital is promoted to the Co $d_{x2\text{-}y2}$ orbital, is

stable, likely due to the lower interelectronic repulsion between the d_{yz} and the electrons of the C-O bond. In the quartet intermediate, the Co d_{z2} donates electrons to the in-plane CO_2 π_{ip}^*. The resulting bonding orbital $\sigma_{Co\text{-}C}$ is strongly Co-centered (82% Co, 18% CO_2). Furthermore, one electron is transferred from the $\pi_{qpy\text{-}1}^*$ to the d_{xz} orbital. Consequently, the Cobalt is best described as a Co^I center (S_{Co}=1) bound to a CO_2 and *ferromagnetically* coupled with a $qpy^{\bullet -}$ ligand (S_{qpy}=1/2). Thus, in terms of electron transfer the step is only accompanied by one electron transfer from the ligand to the metal. The subsequent protonation of the adduct yielding the metallacarboxylic acid intermediate triggers a significant electronic reorganization. Indeed, the $\sigma_{Co\text{-}C}$ bonding orbital is strongly covalent (58% Co, 42% CO_2H). Furthermore, the doublet state is significantly more stable than the quartet, hence the electron populating the $d_{x2\text{-}y2}$ is promoted back to the d_{yz} orbital. Additionnally, the singly populated $1\pi^*$ orbital of this intermediate is a mixture of qpy $\pi_{qpy\text{-}2}^*$ fragment orbital and the $\sigma_{Co\text{-}C}^*$ orbital (53% Co, 39% qpy). In terms of electron transfer, this step corresponds to a one-electron transfer from the cobalt to the CO_2H motif, and a partial electron transfer from the ligand to the cobalt. Finally, the proton-assisted C-O bond cleavage yields a metal-carbonyl adduct. The bonding $\sigma_{Co\text{-}C}$ orbital is mainly CO-centered (26% Co, 71% CO). Futhermore, the electron previously populating the $1\pi^*$ has relocated completely to the antibonding $\sigma_{Co\text{-}CO}^*$. Hence this species is unambiguously assigned as a Co^{II} center bound to a carbonyl ligand. In terms of electronic transfer, the step corresponds to a one-electron transfer from the cobalt to the CO motif, and the completion of the previously partial electron transfer from the ligand to the cobalt.

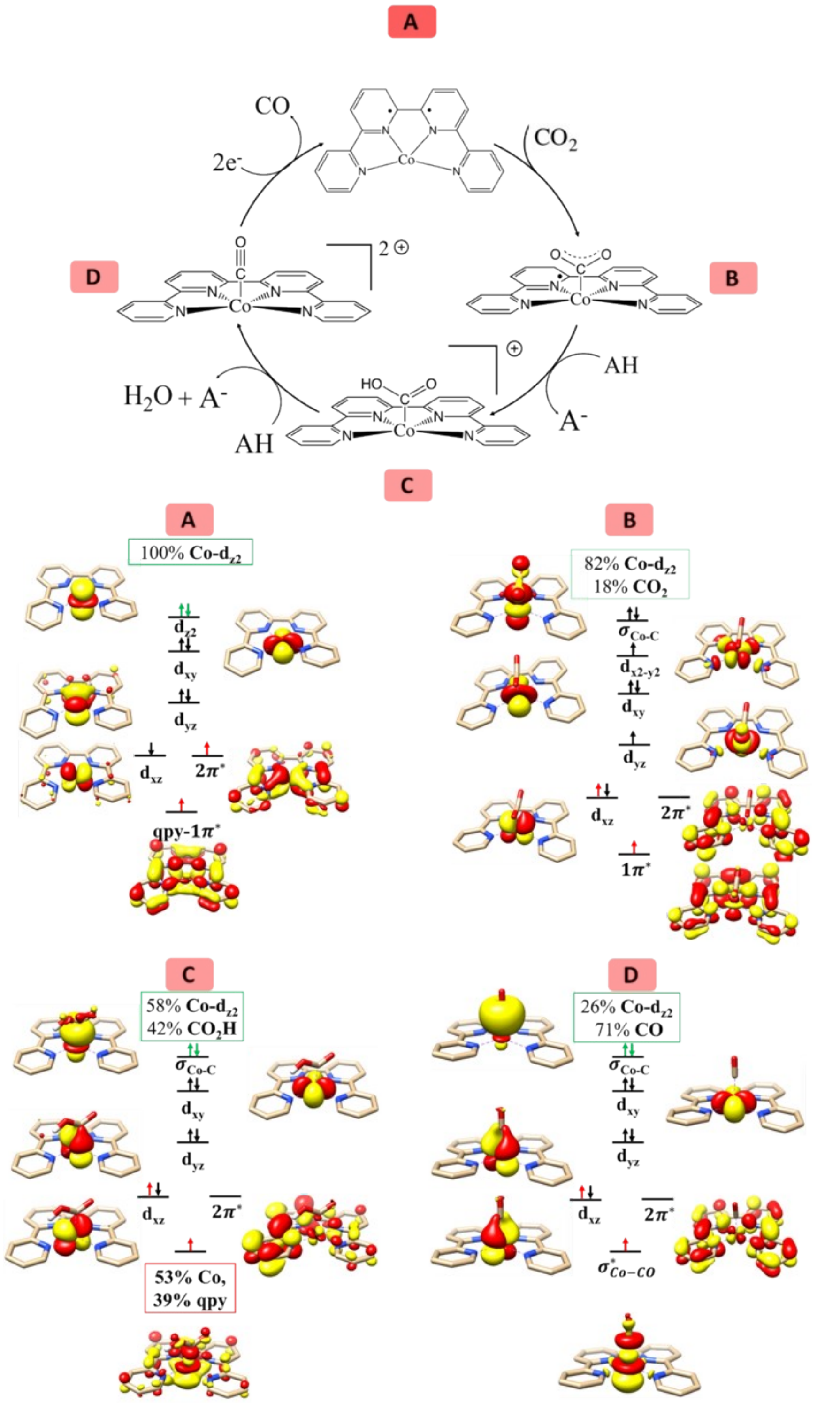

Figure S11: (Top) catalytic cycle of CO_2-to-CO conversion catalysed with [Co(qpy)]. The mechanism is taken from the reference 10. (Bottom) associated electronic structure of the intermediates [Co(qpy)] (A), [Co(qpy)(CO_2)] (B), [Co(qpy)(CO_2H)]$^+$ (C), and [Co(qpy)(CO)]$^{2+}$ (D). Electrons involved in the ligand-to-metal intramolecular transfer are shown by red arrows. Electrons involved in metal-to-CO_2 intramolecular transfer are shown by green arrows. The Co, and CO_2/CO_2H/CO Löwdin population of the $\sigma_{Co\text{-}C}$ bonding orbital is displayed circled in

green. In the intermediate $[Co(qpy)(CO_2H)]^+$, the Co and qpy Löwdin population of the $1\pi^*$ orbital displayed circled in red. Carbons are displayed in beige, oxygen in red and iron in orange. For clarity, all hydrogens are hidden except the hydrogen of the CO_2H motif.

e) Intermediates in the reduction of CO_2 catalyzed with [Co(L)]

Table S6: Electronic energy difference (in kcal/mol) between the spin states of each intermediate involved in the mechanism of CO_2 reduction catalysed with [Co(L)]. For each line, the energy is given relative to the lowest-lying spin state of the intermediate.

	S=1/2	S=3/2	S=5/2
[Co(L)]	0	+5.8	+13.3
$[Co(L)(CO_2)]$	0	+16.9	+27.3
$[Co(L)(CO_2H)]^+$	0	+1.7	+43.0
$[Co(L)(CO)]^{2+}$	+3.4	0	+57.3

The mechanism of CO_2 reduction catalyzed by [Co(L)] is similar to that of $[Co(N_4H)]$. Like for $[Co(N_4H)]$, the ligand environment of [Co(L)] is best described as a square-planar ligand field, despite the presence of a fifth nitrogen donor weakly interacting with the cobalt. However, instead of being described as a Co^{II} antiferromagnetically coupled to a diradical ligand like $[Co(N_4H)]$, it is best described as a low-spin Co^{II} (S_{Co}=1/2) bound to a closed shell L^{2-} ligand (S_L=0). In details, Co features the configuration $(d_{xy})^2(d_{xz})^2(d_{yz})^1(d_{z2})^2$ and two electrons populate the 1a’’ orbital (nomenclature according to the Cs point group). This doubly-populated orbital is an admixture of the ligand-centered fragment orbital $\pi_{L\text{-}a''}^*$ and the singly-occupied Co d_{yz} orbital. During the formation of the $[Co(L)(CO_2)]$ intermediate, the Co d_{z2} donates electrons to the CO_2 π_{ip}^*, forming a bonding orbital $\sigma_{Co\text{-}C}$ mainly centered on Co (61% Co, 35% CO_2). During this step, an electron is transferred from the $\pi_{L\text{-}a''}^*$ to the d_{yz} orbital. Concomitantly, the other electron electron centered on the 1a’’ orbital is promoted to another unoccupied orbital of the ring 1a’. The 1a’ orbital has a different symmetry than that of 1a’’, and in fact predominantly corresponds to the ligand-centered $\pi_{L\text{-}a'}^*$ fragment orbital, slightly mixed with the antibonding $\sigma^*_{Co\text{-}C}$ orbital. Here, the system is best described as a Co^I center (S_{Co}=0) bound to a $L^{\bullet -}$ ligand (S_L=1/2) and a CO_2 motif, and the electron transfer events can be summarized as simply an electron transfer from the ligand to the metal. The subsequent protonation of the adduct affording a metallacarboxylic acid intermediate is characterized by a

significant increase in covalence of the $\sigma_{Co\text{-}C}$ bonding orbital (41% Co, 59% CO_2H). Concomitantly, the mixing of the $\pi_{L\text{-}a'}{}^*$ with the $\sigma^*{}_{Co\text{-}C}$ orbital increases, such as the 1a' orbital is an equal mixture of both fragment (40% Co, 56% L). Hence, this step likely corresponds to the transfer of the first electron to the CO_2 moiety, accompanied by a *partial* electronic transfer from the ligand to the metal. Subsequently, the C-O bond scission occurs coupled with a second proton transfer. After this step is complete the $\sigma_{Co\text{-}C}$ is mainly centered around CO (24% Co, 73% CO). The electron previously populating the $\pi_{L\text{-}a'}{}^*$ orbital is entirely transferred to the antibonding $\sigma^*{}_{Co\text{-}CO}$ orbital. Hence, the oxidation state of this intermediate is clearly identified as a Co^{II} bound to a carbonyl ligand. The step thus corresponds to a second metal-to-CO_2 electron transfer, and the completion of the previously partial electron transfer from the ligand to the metal.

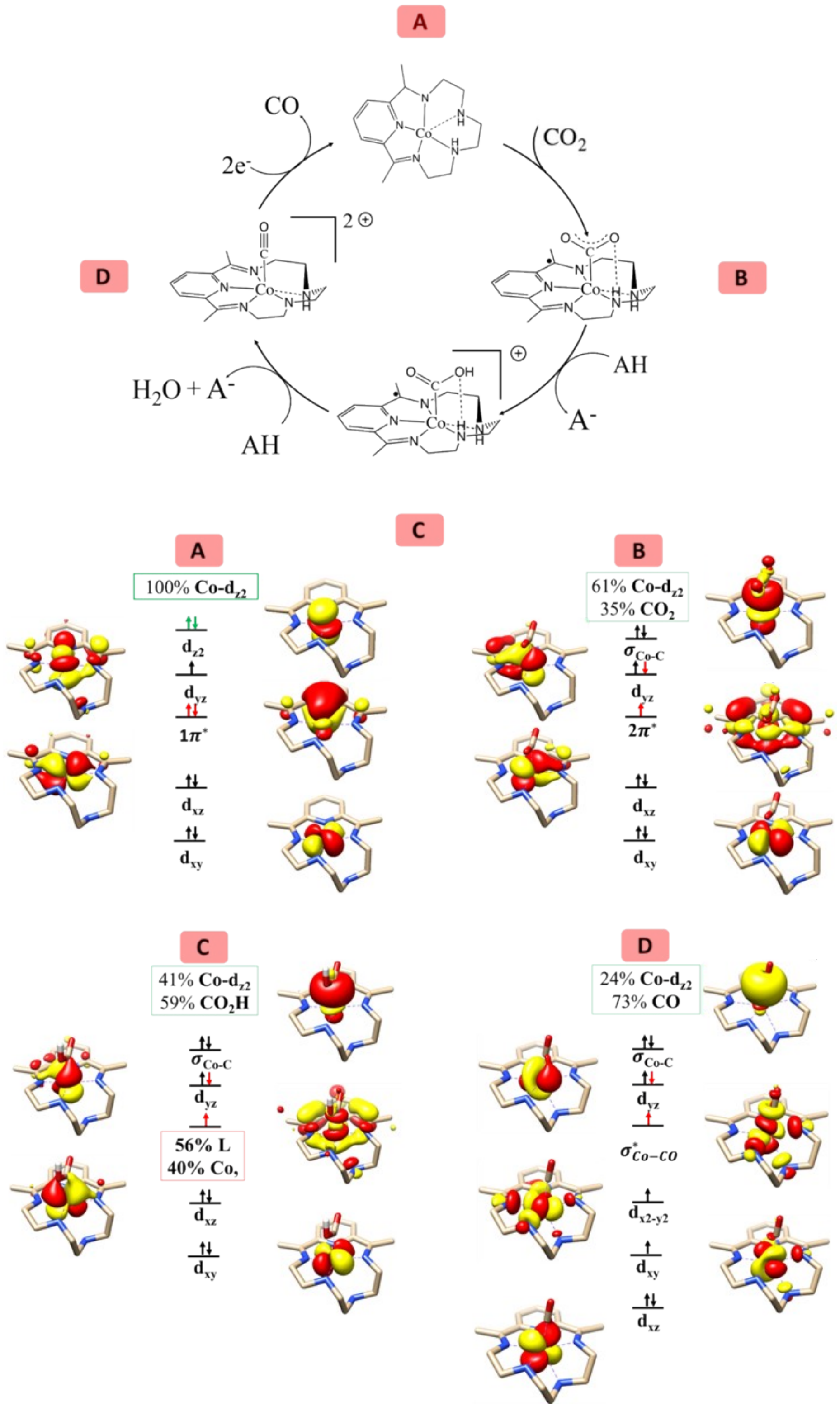

Figure S12: (Top) catalytic cycle of CO_2-to-CO conversion catalysed with [Co(L)]. The mechanism is taken from the reference 6. On the basis of the system's structural and electronic similarity with [Co(N_4H)], it is assumed here that both electrons are transferred from the electrode to the complex after the cleavage of the C-O bond. Dashed bonds between the oxygen of the CO_2 or CO_2H motif and the hydrogen of one amine ligand correspond to hydrogen bonds.

Dashed bond between the nitrogen of the amine ligand and the cobalt center correspond to weak axial interactions. (Bottom) associated electronic structure of the intermediates [Co(L)] (A), [Co(L)(CO_2)] (B), [Co(L)(CO_2H)]$^+$ (C), and [Co(L)(CO)]$^{2+}$ (D). Electrons involved in the ligand-to-metal intramolecular transfer are shown by red arrows. Electrons involved in metal-to-CO_2 intramolecular transfer are shown by green arrows. The Co and CO_2/CO_2H/CO Löwdin population of the $\sigma_{Co\text{-}C}$ bonding orbital is displayed circled in green. In the intermediate [Co(L)(CO_2H)]$^+$, the Co and L Löwdin population of the 1a' orbital is displayed circled in red. Carbons are displayed in beige, oxygen in red and iron in orange. For clarity, all hydrogens are hidden except the hydrogen of the CO_2H motifand the hydrogens participating to the intramolecular hydrogen bonds.

f) Intermediates in the reduction of CO_2 catalyzed with [Mn (bpy)$(CO)_3$]K

The mechanism resembles closely that of [Re(bpy)$(CO)_3$]K. In the first intermediate [Mn (bpy)$(CO)_3$]K, the ligand field is square-pyramidal. The Mn features a configuration $(d_{xy})^2(d_{xz})^2(d_{yz})^2$ and the HOMO $1\pi^*$ is an admixture of Mn and π_{bpy}^* fragment orbitals (24% Mn and 62% bpy). Thus, it is best described as a Mn^I center (S_{Mn}=0) bound to a doubly-reduced bpy^{2-} ligand (S_{bpy}=0). During the formation of the adduct [Mn(bpy)$(CO)_3(CO_2)$]K, a two-electron transfer from the π_{bpy}* orbital to the formally empty Mn d_{z2} orbital takes place, and the latter orbital donates the electron density into the CO_2 π_{ip}* orbital. The subsequently-formed bonding $\sigma_{Mn\text{-}C}$ orbital has a strong covalent character (28% Mn and 55% CO_2). Although the $\sigma_{Mn\text{-}C}$ orbital is partially delocalized to the carbonyl ligands, the system is best described as a resonance form between a Mn^I center (S_{Mn}=0) bound to a $(CO_2)^{2-}$ ligand and a Mn^{-I} center bound to a neutral CO_2 ligand. Hence this step corresponds to a two-electron transfer from the ligand to the metal concomitant with a one-electron transfer from the metal to the CO_2 moiety. The subsequent protonation of the adduct yielding a [Mn(bpy)(CO_2H)$(CO)_3$]K^+ further polarizes the Mn-C_{CO2} bond since the bonding molecular orbital is about 28% Mn and 63% CO_2H. In two distinct subsequent steps, an electron is transferred to the catalyst from the electrode and the C-O bond is cleaved concomitantly with a second proton transfer. As elaborated elsewhere,[19] the electron transfer may be before (reduction-first) or after (protonation-first) the proton-assisted cleavage of the bond. We did not investigate that level of details. However, this two-steps sequence yields a metal-carbonyl adduct In this species, the $\sigma_{Mn\text{-}C}$ orbital is mainly CO centered, and the Löwdin populations remain almost identical to

those of the metallacarboxylic acid (30% Mn, 64% CO). The additional electron transferred from the electrode resides in the 1π^*, here primarily composed of the bpy π^* fragment orbital. Furthermore, this species feature significant backdonation from the Mn d_{xz} and d_{yz} to the empty π^* orbitals of the CO ligand. Therefore, the electronic structure of this species is identified as a Mn^{I} species (S_{Mn}=0) bound to a radical bpy$^{\bullet-}$ (S_{bpy}=1/2) and a CO ligand.

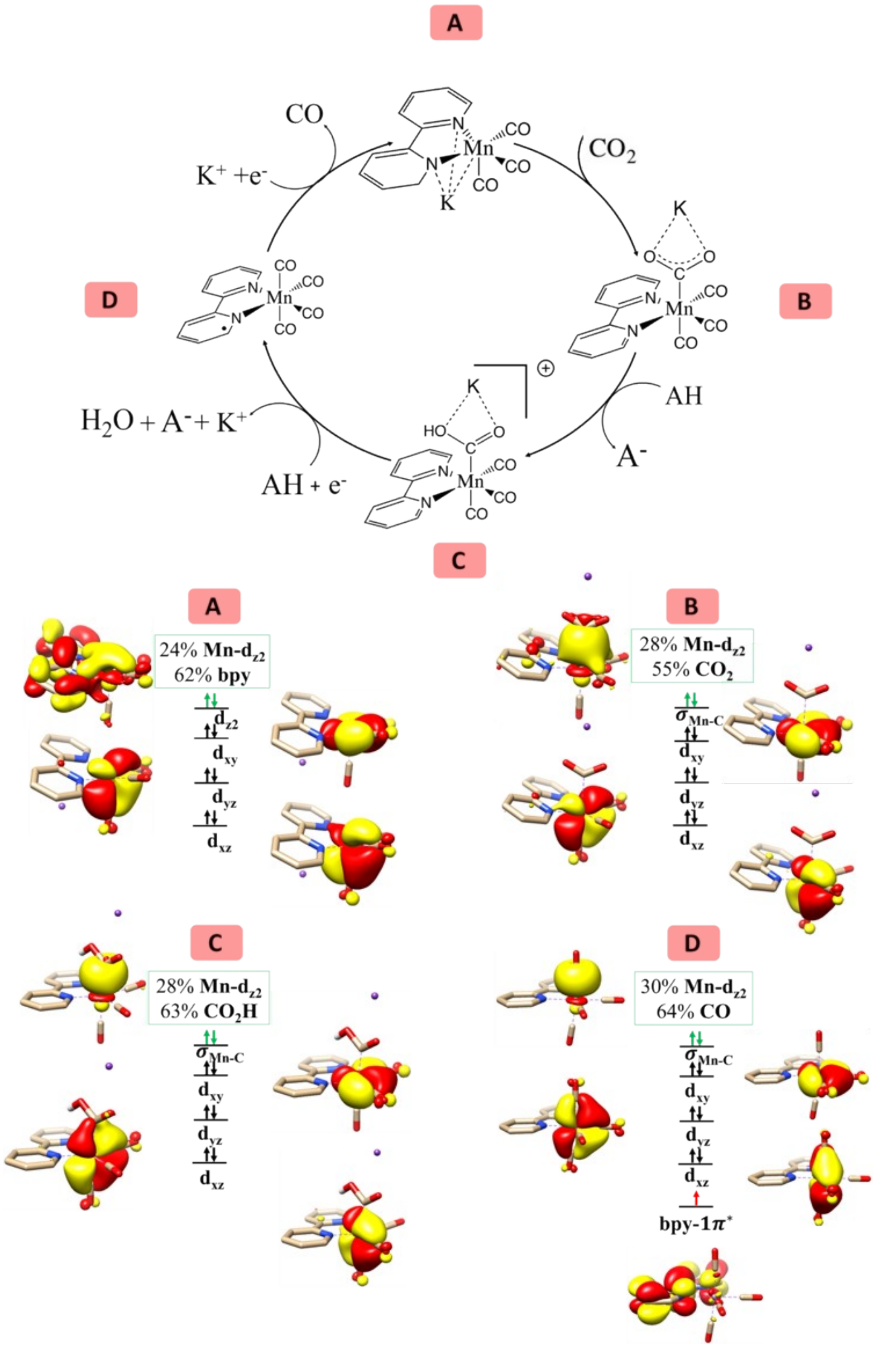

Figure S13: (Top) catalytic cycle of CO_2-to-CO conversion catalysed with [Mn(bpy)(CO)$_3$]K. The mechanism is taken from the reference 19. A K^+ counterion was added to the reaction complex, by analogy to the mechanistic studies on the very similar [Re(bpy)(CO)$_3$]K complex described in reference 15. Dashed bond correspond to weak bonds formed with the K^+ ion. (Bottom) associated electronic structure of the intermediates [Mn(bpy)(CO)$_3$]K (A), [Mn(bpy)(CO$_2$)(CO)$_3$]K (B), [Mn(bpy)(CO$_2$H)(CO)$_3$]K^+ (C), and [Mn(bpy) (CO)$_4$]. The electrons occupying the d-orbitals of the metal and the redox-active orbitals of the ligand are represented by arrows. Electrons involved in the ligand-to-metal and metal-to-CO_2 intramolecular transfer are shown by green arrows. The Mn, bpy Löwdin population of the $1\pi^*$ orbital (for A) and the Mn, bpy and CO_2/CO_2H/CO Löwdin population of the $\sigma_{Mn\text{-}C}$ bonding orbital (for B-D) is displayed circled in green. Carbons are displayed in beige, oxygen in red and iron in orange. For clarity, all hydrogens are hidden except the hydrogen of the CO_2H motif.

g) Intermediates in the reduction of CO2 catalyzed with [Cr(bpy)(CO)3]$^{2-}$

Table S7: Electronic energy difference (in kcal/mol) between the spin states of each intermediate involved in the mechanism of CO_2 reduction catalysed with [Cr(bpy)(CO)$_3$]$^{2-}$. For each line, the energy is given relative to the lowest-lying spin state of the intermediate.

	S=0	S=1	S=2
[Cr(bpy)(CO)$_3$]$^{2-}$	0	18.3	52.5
[Cr(bpy)(CO$_2$)(CO)$_3$]$^{2-}$	0	11.8	50.7
[Cr(bpy)(CO$_2$H)(CO)$_3$]$^-$	0	25.8	62.9
[Cr(bpy) (CO)$_4$]$^-$	0	45.6	74.5

This mechanism is similar to that of [Re(bpy)(CO)$_3$]$^-$. The active species, [Cr(bpy)(CO)$_3$]$^{2-}$ possesses a square-pyramidal ligand field. It is identified as a Cr^0 center (S_{Cr}=0) bound to a doubly-reduced bpy^{2-} ligand (S_{bpy}=0). In details, the Cr center has a $(d_{xy})^2(d_{xz})^2(d_{yz})^2$ configuration, and the HOMO of [Cr(bpy)(CO)$_3$]$^{2-}$ ($1\pi^*$) is an admixture of Cr d_{z2} and bpy π^* fragment orbitals (17% Cr, 75% bpy). During the formation of the adduct [Cr(bpy)(CO)$_3$(CO$_2$)]$^{2-}$, a two-electron transfer from the $1\pi^*$ orbital to the formally empty d_{z2} orbital takes place, and the latter orbital donates the electron density into the CO_2 in-plane π_{ip}^* orbital. The subsequently formed bonding orbital $\sigma_{Cr\text{-}C}$ demonstrates a significant covalent Cr-C_{CO2} bond (31% Cr, 51% CO_2), although the orbital remains partially delocalized over the

carbonyl ligands. This intermediate is best described as a resonant structure between a Cr^0 center (S_{Cr}=0) bound to a $(CO_2)^{2-}$ ligand and a Cr^{-II} center bound to a CO_2 ligand. The proton transfer step affording the metallarboxylic acid intermediate increases the weight of the CO_2H motif in the bonding orbital (28% Cr, 64% CO_2H). Here, the system is best described as a Cr^0 (S_{Cr}=0) bound to a $(CO_2H)^-$ ligand. The two subsequent steps are the proton-assisted C-O bond cleavage and electron transfer from the electrode, in an unknown order. Determining whether this species follows a "protonation-first" or "reduction-first" pathway is beyond the scope of this study. However, this two-step sequence yields a metal-carbonyl adduct. The 1σ bonding orbital remains mainly centered around CO (29% Cr, 65% CO) but does not further polarize. The electron is transferred from the electrode to the $1\pi^*$ orbital, entirely centered around the ligand. Hence the system is best described as a Cr^0 (S_{Cr}=0) bound to a carbonyl and a $bpy^{\bullet -}$ ligand (S_{bpy}=1/2).

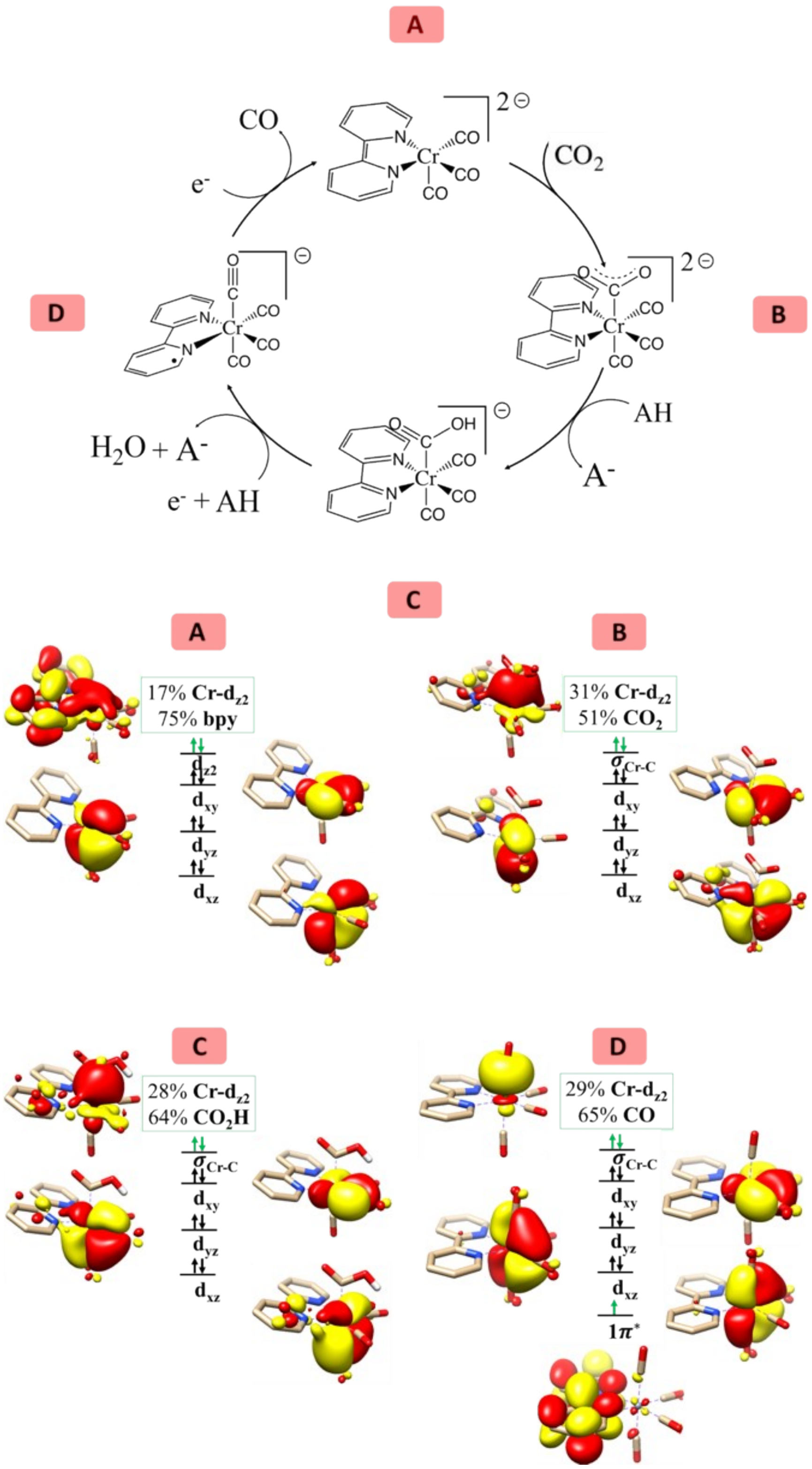

Figure S14: (Top) catalytic cycle of CO_2-to-CO conversion catalysed with $[Cr(bpy)(CO)_3]^{2-}$. The type of mechanism is postulated to be similar to that of $[Re(bpy)(CO)_3]^{-}$ and $[Mn(bpy)(CO)_3]^{-}$. Cyclic voltammetry data suggests the active species is $[Cr(bpy)(CO)_3]^{2-}$.[3] (Bottom) associated electronic structure of the intermediates $[Cr(bpy)(CO)_3]^{2-}$ (A), $[Cr(bpy)(CO)_3(CO_2)]^{2-}$ (B), $[Cr(bpy)(CO)_3(CO_2H)]^{-}$ (C), and $[Cr(bpy)(CO)_4]$ (D). The

electrons occupying the d-orbitals of the metal and the redox-active orbitals of the ligand are represented by arrows. Electrons involved in the ligand-to-metal and metal-to-CO_2 intramolecular transfer are shown by green arrows. The Cr, bpy Löwdin population of the orbital $1\pi^*$ (for A) and the Cr, bpy, CO_2/CO_2H/CO Löwdin population of the σ_{Cr-C} bonding orbital (for B-D) is displayed circled in green. Carbons are displayed in beige, oxygen in red and iron in orange. For clarity, all hydrogens are hidden except the hydrogen of the CO_2H motif.

h) Intermediates in the reduction of CO_2 catalyzed with $[Ru(bpy)_2(CO)]$

Table S8: Electronic energy difference (in kcal/mol) between the spin states of each intermediate involved in the mechanism of CO_2 reduction catalysed with $[Ru(bpy)_2(CO)]$. For each line, the energy is given relative to the lowest-lying spin state of the intermediate.

Intermediate	S=0	S=1	S=2
$[Ru(bpy)_2(CO)]$	0	+8.6	+40.7
$[Ru(bpy)_2(CO)(CO_2)]$	0	+30.3	+63.8
$[Ru(bpy)_2(CO)(CO_2H)]^+$	0	+53.0	+118.5
$[Ru(bpy)_2(CO)(CO)]^{2+}$	0	+64.8	+128.7

The active species, $[Ru(bpy)_2(CO)]$, has a square-pyramidal ligand-field symmetry similar to $[Re(bpy)(CO)_3]^-$. The ruthenium has a $(d_{xy})^2(d_{xz})^2(d_{yz})^2$ electronic configuration. The HOMO of $[Ru(bpy)_2(CO)]$ ($1\pi^*$) is strongly delocalized and mainly corresponds to an admixture of Ru d_{z2} (44%) and bpy π^* (33%) from one bipyridine ligand. Hence the system is best described as a hybrid form between a Ru^{II} (S_{Ru}=0) system bound to a $(bpy)^{2-}$ (S_{bpy}=0), and a Ru^0 bound to a neutral bpy ligand. During the formation of the adduct $[Ru(bpy)_2(CO)(CO_2)]$, two electron transfers from the $1\pi^*$ orbital to the d_{z2} orbital takes place, and the latter orbital donates two electrons into the CO_2 in-plane π^* orbital. The subsequently-formed bonding orbital σ_{Ru-C} is strongly covalent (46% Ru and 49% CO_2), such that the species is best described as a resonant hybride between a Ru^{II} center (S_{Ru}=0) bound to a $(CO_2)^{2-}$ ligand and a Ru^0 center (S_{Ru}=0) bound to a CO_2 ligand. The bpy ligand is neutral. Hence, this step corresponds to an overall electron transfer from the bpy ligand to the CO_2. The subsequent protonation further polarizes σ_{Ru-C} (37% Ru and 59% CO_2H). However, the bond is still strongly covalent and the oxidation state of the ruthenium remains ambiguous. Finally, the proton-assisted cleavage of the C-O bond yields a metal-carbonyl adduct. The bonding orbital σ_{Ru-C} is largely CO-centered

at this point, (32% Ru, 64% CO) and the system unambiguously described as a Ru^{II} center (S_{Ru}=0) bound to a carbonyl ligand.

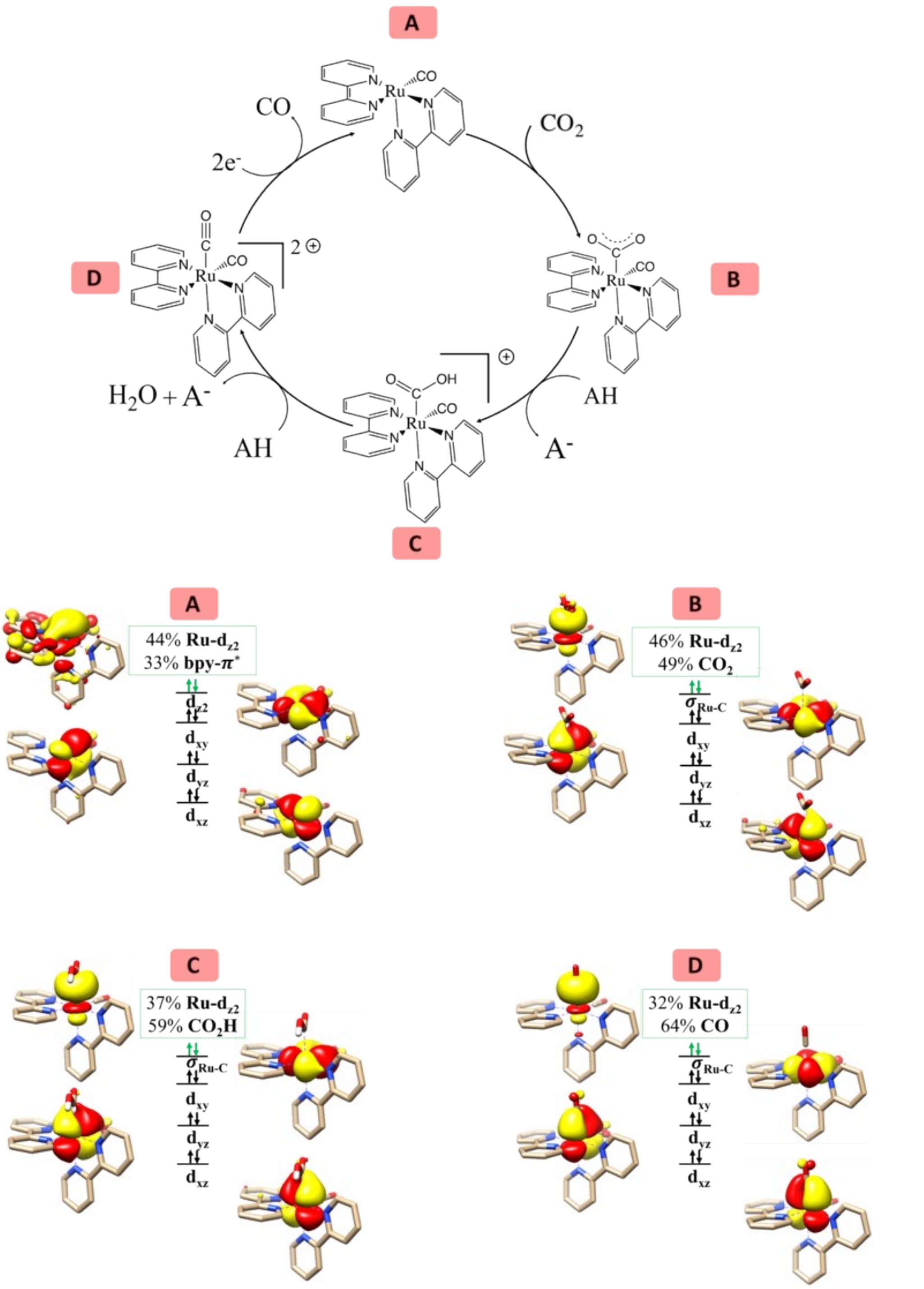

Figure S15: (Top) catalytic cycle of CO_2-to-CO conversion catalysed with $[Ru(bpy)_2(CO)]$. The mechanism is taken from the reference 7. (Bottom) associated electronic structure of the intermediates $[Ru(bpy)_2(CO)]$ (A), $[Ru(bpy)_2(CO)(CO_2)]$ (B), $[Ru(bpy)(tpy)(CO)(CO_2H)]^+$

(C), and $[Ru(bpy)_2(CO)_2]^{2+}$ (D). The electrons occupying the d-orbitals of the metal and the redox-active orbitals of the ligand are represented by arrows. Electrons involved in the ligand-to-metal and metal-to-CO_2 intramolecular transfer are shown by green arrows. The Ru, bpy Löwdin population of the orbital $1\pi^*$ (A) and Ru and CO_2/CO_2H/CO Löwdin population of the $\sigma_{Ru\text{-}C}$ bonding orbital is displayed circled in green. Carbons are displayed in beige, oxygen in red and iron in orange. For clarity, all hydrogens are hidden except the hydrogen of the CO_2H motif.

i) Intermediates in the reduction of CO_2 catalyzed with [Ru(bpy)(tpy)]

Table S9: Electronic energy difference (in kcal/mol) between the spin states of each intermediate involved in the mechanism of CO_2 reduction catalysed with [Ru(bpy)(tpy)]. For each line, the energy is given relative to the lowest-lying spin state of the intermediate.

Intermediate	S=0	S=1	S=2
[Ru(bpy)(tpy)]	0	7.7	34.1
[Ru(bpy)(tpy)(CO_2)]	0	58.7	63.7
[Ru(bpy)(tpy)(CO)]	<0.1	0	50.7

Unlike most investigated catalysts, this catalyst yields CO under aprotic conditions, following the stochiometric equation:

$$2CO_2 + 2\,e^- \rightarrow CO + CO_3^{2-} \quad \text{(S18)}$$

Very similar to $[Re(bpy)(CO)_3]^-$, the active form of the catalyst exhibits a ligand field best described as a deformed square-pyramidal. The ruthenium has a $(d_{xy})^2(d_{xz})^2(d_{yz})^2$ electronic configuration and the HOMO ($1\pi^*$) is a mixture of 27% Ru d_{z2} and 69% tpy π^* orbital. Hence, the species can be described as a Ru^{II} (S_{Ru}=0) bound to a tpy^{2-} ligand. During the formation of the adduct [Ru(bpy)(tpy)(CO_2)], a two-electron transfer from the $1\pi^*$ orbital to the formally empty d_{z2} orbital takes place, and the latter orbital donates the electron density into the CO_2 in-plane π^* orbital. The subsequently-formed bonding orbital is strongly covalent (44% Ru and 49% CO_2), such that the species is best described as a resonant hybride between a Ru^{II} center (S_{Ru}=0) bound to a $(CO_2)^{2-}$ ligand and a Ru^0 center (S_{Ru}=0) bound to a CO_2 ligand. Thus it is safe to consider this step as a first electron transfer to the CO_2 motif. Subsequently, the catalyst accepts two electrons from the electrode. The electron-accepting orbitals $1\pi^*$ and $2\pi^*$ are entirely terpyridine- and bipyridine-based, respectively. The subsequent C-O bond cleavage

yields a metal-carbonyl. In the latter species, the bonding orbital is largely CO-centered (33% Ru, 63% CO). Hence this step can be interpreted as the second electron transfer to CO_2. Meanwhile, the two electrons previously transferred from the electrode remained centered on the ligand. Hence, this species can be interpreted as a Ru^{II} center (S_{Ru}=0) bound to a $bpy^{•-}$ and a $tpy^{•-}$ ligands. Notably, the energy the present triplet state and the singlet state is below the error range of DFT for this intermediate. The latter is a closed-shell system in which a Ru^{II} (S_{Ru}=0) of similar electronic configuration is bound to a tpy^{2-} ligand and a neutral bpy ligand instead of two radical ligands $bpy^{•-}$ and $tpy^{•-}$.

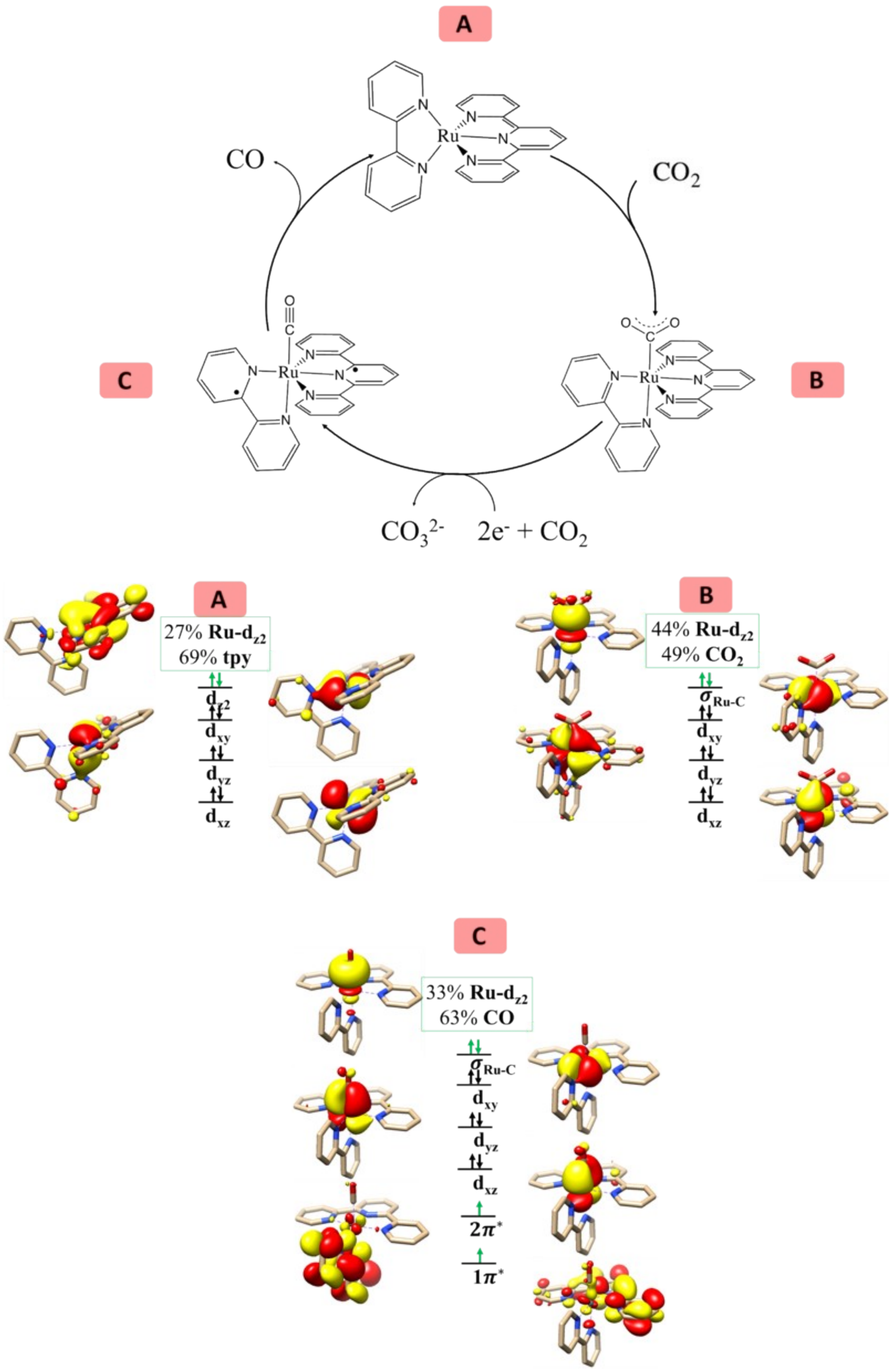

Figure S16: (Top) catalytic cycle of CO_2-to-CO conversion catalysed with [Ru(bpy)(tpy)]. The mechanism is taken from the reference 8. (Bottom) associated electronic structure of the intermediates [Ru(bpy)(tpy)] (A), [Ru(bpy)(tpy)(CO_2)] (B), and [Ru(bpy)(tpy)(CO)] (C). The electrons occupying the d-orbitals of the metal and the redox-active orbitals of the ligand are

represented by arrows. Electrons involved in the ligand-to-metal and metal-to-CO_2 intramolecular transfer are shown by green arrows The Ru, tpy Löwdin population of the orbital $1\pi^*$ (A) and Ru and CO_2/CO_2H/CO Löwdin population of the $\sigma_{Ru\text{-}C}$ bonding orbital (B-D) is displayed circled in green. Carbons are displayed in beige, oxygen in red and iron in orange. For clarity, all hydrogens are hidden except the hydrogen of the CO_2H motif.

6) Energy of the calculated structures

Table S10: Electronic energies, enthalpies and free energy for each of the studied intermediates of the CO_2-to-CO conversion, CO_2-to-HCOOH conversion and hydrogen evolution reaction catalysed by $^{1}\mathbf{1}^{2-}$. The electronic energy consists of the sum of terms $E_{el} + \Delta E_{el}$ in eq. 1a (Main Text). Except mentioned otherwise, the energy is obtained at the B3LYP/Def2-TZVPP level of theory, as explicated in the main text. For **PhOH**, ^{1}**TS2** and ^{1}**TS3**, a single-point using the double-hybrid function w-B2PLYP and the basis set Def2-QZVPP was also calculated. The enthalpy consists of the sum of all terms in eq. 1a (Main Text). The Free energy corresponds to the sum of all terms in eq. 1b (Main Text). All energies are displayed in Hartrees. The energy reference is that of all atoms and electrons infinitely separated in vacuum. All compounds in bold are schematically drawn in Figure 1 (Main Text) and Figure S6.

Compound	El. Energy	Enthalpy	Free energy.
^{3}**1**	-3176.06465	-3175.42925	-3175.51875
$^{2}\mathbf{1}^{-}$	-3176.17597	-3175.54762	-3175.63647
$^{1}\mathbf{1}^{2-}$	-3176.26322	-3175.64346	-3175.73183
$^{3}\mathbf{1}^{2-}$	-3176.25194	-3175.63322	-3175.72281
$^{5}\mathbf{1}^{2-}$	-3176.24213	-3175.62250	-3175.71249
^{1}**TS1**	-3364.85261	-3364.21730	-3364.310805
^{1}**A**	-3364.86420	-3364.22860	-3364.321159
^{3}**A**	-3364.85304	-3364.21837	-3364.31633
^{5}**A**	-3364.84656	-3364.20995	-3364.30901
	(Dissociated)	(Dissociated)	(Dissociated)
^{1}**B**	-3672.33143	-3671.58263	-3671.69025
^{1}**C**	-3979.78454	-3978.92216	-3979.03767
^{1}**TS2**	-3979.76284	-3978.90247	-3979.02106

**^{1}TS2 (w-B2PLYP)**	-3975.90886	-3975.04849	-3975.16708
**^{1}TS3**	-3672.31636	-3671.57053	-3671.67523
**^{1}TS3 (w-B2PLYP)**	-3668.87136	-3668.12553	-3668.23023
**^{1}D** + PhO^-	-3672.32025	-3671.57076	-3671.67558
**^{1}D**	-3365.35250	-3364.69560	-3364.78858
**^{3}D**	-3365.32961	-3364.67396	-3364.76896
**^{5}D**	-3365.31805	-3364.66309	-3364.75968
**^{1}TS5**	-3365.27937	-3364.63020	-3364.719416
**^{1}E**	-3672.81569	-3672.04672	-3672.15331
**^{1}TS4**	-3672.79696	-3672.03337	-3672.14072
**^{1}F**	-3289.39413	-3288.74772	-3288.83968
**^{3}F**	-3289.38116	-3288.73697	-3288.83162
**^{5}F**	-3289.37548	-3288.73186	-3288.82847
**^{1}TS6**	-3483.68772	-3482.96355	-3483.05973
**^{1}M**	-3176.7347	-3176.09891	-3176.18736
**^{3}M**	-3176.72224	-3176.08997	-3176.17939
**^{5}M**	-3176.70962	-3176.07733	-3176.16789
**^{1}N**	-3365.3771	-3364.72071	-3364.81686
**^{3}N**	-3365.38696	-3364.73079	-3364.82850
**^{5}N**	-3365.38898	-3364.73402	-3364.83332
CO2	-188.59192	-188.57654	-188.59326
PhOH	-307.44200	-307.33077	-307.35834
PhOH (w-B2PLYP)	-307.03966	-306.92843	-306.95600
PhO^-	-306.95431	-306.86203	-306.8892
H2O	-76.43994	-76.41509	-76.42897
OH-	-75.91738	-75.91019	-75.92217
$[OH_2...(OPh)_2]^{2-}$	-690.37506	-690.15945	-690.20644
$[OH_2...OPh]^-$	-383.40927	-383.28869	-383.321755
H_2	-1.17383	-1.16043	-1.16751

7) Cartesian coordinates and imaginary normal modes of vibrations for the intermediates and transition states involved in CO_2 reduction and H_2 generation by $\mathbf{1}^{2-}$

a) $^{3}\mathbf{1}$

No Imaginary frequencies.

Fe	-2.09956900610000	7.95205437800771	8.88522516735839
N	-3.55149084089256	8.96821217798403	9.82446494997772
N	-3.24827057786289	7.82752589080681	7.24556272290606
N	-0.64809305562093	6.93507675059479	7.94642524973733
N	-0.95059841987550	8.07711055560886	10.52488607022642
C	-3.51200156394445	9.49026661929519	11.10390087702745
C	-4.77533177951535	9.34798357006235	9.30499564191538
C	-4.51757120510103	8.34741449431300	7.07234200820083
C	-2.93643359264416	7.20111444691082	6.05312645543189
C	-0.68817694964835	6.41158156550892	6.66759000372065
C	0.31944138344332	7.55884832645850	10.69751021331391
C	-1.26222034528808	8.70405697830422	11.71706156366774
C	-4.72539124013780	10.21695648149780	11.38486981265243
H	-4.94147538319853	10.73815174973789	12.31351847012792
C	-5.50802333598863	10.12829767369694	10.27078195694508
H	-6.49064583555603	10.56261731303134	10.10748070295094
C	-5.00783116942454	8.03985948155799	5.75166655808307
H	-5.98044074310450	8.32828918225882	5.36240302791172
C	-4.02451240325159	7.33878636464877	5.11692299519809
H	-4.03239644934755	6.94593812892204	4.10388696704852
C	0.81066085051622	7.86859372412466	12.01733570873627
C	-0.17296661554894	8.56903013296777	12.65229916524135
H	-0.16467465104321	8.96301776052326	13.66489336955794
C	-5.25250109486044	9.06302035907643	8.02214471486367
C	-1.74665370972964	6.52721135868144	5.76141560721127
C	-2.45292511446823	9.37591676494710	12.00950764851656

C	-6.61568455701381	9.54892332748129	7.64599595471707
C	-7.76111933567562	8.96114215056734	8.20725399710913
H	-7.64696621071573	8.14403445611218	8.92388162980980
C	-9.03659258973629	9.40900080938186	7.85117193728658
C	-6.77245610617097	10.59667188645093	6.72367602854450
H	-5.88599143616032	11.06184356920440	6.28567181459316
C	-1.60304670232057	5.88756640933091	4.41784568990932
C	-0.71824315904807	6.41569815365652	3.46363548565868
H	-0.13546719358366	7.30687660429089	3.70903176512773
C	-0.58346751800261	5.81300350986358	2.20947977772309
H	0.10671272939519	6.23673316632569	1.47535448453150
C	-1.33023375614220	4.67281515910606	1.89404306894057
H	-1.22428255441712	4.20110914480658	0.91378126342970
C	-2.21331444980701	4.13895980420402	2.83896499130190
H	-2.79738225133167	3.24596436924856	2.60163601687780
C	-2.34928966769881	4.74300842941947	4.09207189019970
H	-3.03668904705723	4.32419684085937	4.83113044174409
C	-2.59683887806876	10.01514276633004	13.35330046537887
C	-3.47801141259345	9.48346778693206	14.30887876247083
H	-4.05776942470417	8.59009104382171	14.06432496205406
C	-1.85455742677945	11.16258688109138	13.67780687950330
H	-1.17003648090721	11.58407091664410	12.93759346570205
C	-3.61307243178370	10.08556785399247	15.56331109683333
H	-4.30042235289746	9.65913666685451	16.29852595028492
C	-2.87020437650496	11.22863391948547	15.87754681115127
H	-2.97629254544522	11.69982991116109	16.85804220773317
C	-1.99082921981437	11.76602547788312	14.93118682536720
H	-1.40989472829823	12.66129898673865	15.16761684917815
C	-9.18312374072065	10.45264394359792	6.93076214344331
H	-10.18033245417152	10.80322006261325	6.65226161778597
C	-8.04773954793880	11.04602515676561	6.36864169645635
H	-8.15467797915032	11.86437068719001	5.65171995301868
H	3.95769969518147	4.04512577765050	12.12175300066229

H	5.98253103301287	5.10343854368360	11.11655666843500
C	0.57552855346440	6.55491594161683	8.46617504179399
C	0.52423770277826	5.68287239319178	6.38761249158461
H	0.73957078872907	5.15989834234309	5.45979817816850
C	1.30711866436941	5.77226336531627	7.50145942569206
H	2.28923941023094	5.33702748877132	7.66532381513335
H	1.78408850457690	7.58191069808792	12.40585117719906
C	1.05369023927001	6.84190825679031	9.74821782090317
C	2.41719238532643	6.35659077784785	10.12401569401336
C	2.57464821433358	5.31088256542435	11.04856096754460
H	1.68852927824875	4.84698101304626	11.48859811809999
C	3.85019195609483	4.86191096905666	11.40313816917964
C	4.98511458661361	5.45370560877548	10.83840690999427
C	4.83788686884646	6.49534623851400	9.91585126036752
H	5.72004255087900	6.96427630647936	9.47231999974153
C	3.56215004143281	6.94278322285829	9.56016605626412
H	3.44739964360577	7.75829551546503	8.84182036847660
H	-9.91907048809681	8.93879169818988	8.29270637181018

b) $^{2}\mathbf{1}^{-}$

No imaginary frequency.

Fe	-2.13286992952002	7.93760308278297	8.88232143118719
N	-3.58682551764760	8.96483063362922	9.83338322808550
N	-3.28433812417143	7.78871318707596	7.26002348980587
N	-0.68833012363692	6.88294162406422	7.94518835035653
N	-0.97026858433677	8.11014912058203	10.49148330696221
C	-3.51802153182175	9.56003155122638	11.08415535655750
C	-4.84434511282681	9.27997852796560	9.33963858508827
C	-4.58890947240828	8.26112897262302	7.10779798002278
C	-2.95166432298203	7.21642036554033	6.03201051887133
C	-0.71881508478305	6.37844520134263	6.65647565381508

C	0.29293270917041	7.54780202673292	10.67941056648546
C	-1.23632316996047	8.82175867910224	11.66050199365467
C	-4.72707466035464	10.25862160976255	11.36577687190656
H	-4.93188806462720	10.82668016140584	12.26918360349524
C	-5.56024746997135	10.07553601290383	10.27756758645833
H	-6.56165041647285	10.47260250197769	10.13251443988277
C	-5.06906564770274	7.96896397576116	5.77122791938517
H	-6.05800576781037	8.21680977735858	5.39466385218459
C	-4.05773844805060	7.35747794357595	5.10412580085159
H	-4.05683125045587	7.01217289183921	4.07371229811378
C	0.82024609170203	7.93015006007255	11.97336261229685
C	-0.11585556591847	8.70568955235494	12.57640956359629
H	-0.06517742481455	9.15624633894726	13.56363340648576
C	-5.33841170424624	8.93036903641599	8.05881128618127
C	-1.76315460699011	6.57914721031090	5.71821098063773
C	-2.40271198728563	9.50860563468651	11.95849674203041
C	-6.73085574810476	9.35147272363768	7.71517952870346
C	-7.83596623758916	8.74645654048094	8.33871232816178
H	-7.66293585189965	7.96358826038491	9.08144996106045
C	-9.14063837223150	9.13232139444714	8.01542173445038
C	-6.96778882521969	10.36197790151846	6.76751252563148
H	-6.11618909363763	10.84681628159145	6.28438281968741
C	-1.54501128423508	6.05557879081510	4.33740652767687
C	-0.51695537028270	6.59244188141325	3.54097817193839
H	0.09164033902405	7.40717845855422	3.94061281529146
C	-0.26479118371492	6.09266799361828	2.26105001818125
H	0.53794005695307	6.52441126052153	1.65729533444965
C	-1.03929496409246	5.04341217751729	1.75182804162873
H	-0.83358033488270	4.64304532325875	0.75535654761318
C	-2.07245240733697	4.50695587412176	2.52786411173073
H	-2.68346609149024	3.68840065239714	2.13749458453797
C	-2.31955772348745	5.00697582749746	3.81105203247720
H	-3.11458693615175	4.57315574244312	4.42234632704968

C	-2.50024716396100	10.20589964449707	13.27551499780692
C	-3.42834882020238	9.78906214877327	14.24784455084827
H	-4.10207871957429	8.95974809247500	14.02006043973863
C	-1.64205065169030	11.27543556560050	13.58900088723827
H	-0.92488631736597	11.61677356507488	12.83840653631980
C	-3.48688158028490	10.41252063672614	15.49722384450214
H	-4.20818706538805	10.06690080916329	16.24280649214005
C	-2.61282612451207	11.46312101284803	15.80178926854499
H	-2.65022157505207	11.94450437007729	16.78252145689571
C	-1.69199293285827	11.89551017390157	14.84136314393361
H	-1.01049174656253	12.71983125976039	15.06859843534264
C	-9.36320007048250	10.13217737157908	7.06037864352091
H	-10.38118121022480	10.43603868724824	6.80047274889031
C	-8.27131663812108	10.74758184936049	6.43821572866404
H	-8.43631060716721	11.53385961340794	5.69659398022100
H	3.74331620955638	3.92494116007643	12.30492979000332
H	5.81931344139929	4.84724322648474	11.28048612613391
C	0.50145175823874	6.42808346543836	8.49437016694670
C	0.43776604933773	5.58396591436935	6.40714512282275
H	0.65381103769462	5.05690239618496	5.48174130850273
C	1.19798070105228	5.60940770904477	7.56196433280057
H	2.14666826961103	5.11202119396114	7.74671748261664
H	1.78947008454872	7.63092449915237	12.36416711062165
C	0.98121114985295	6.74707342204936	9.78644506977344
C	2.31684209314092	6.21023686420013	10.18794583373437
C	2.42554076449664	5.19903660788082	11.15698713178018
H	1.51569550327768	4.79746439775471	11.61001125425661
C	3.67704466075472	4.71124992826218	11.54810698522658
C	4.84203002597043	5.22801108926964	10.97055431598627
C	4.74646504093536	6.23065455051856	9.99733338974774
H	5.64985225414167	6.64339279830922	9.53968609023465
C	3.49377139541674	6.71494560477016	9.60909568263131
H	3.41791063375687	7.50019854287908	8.85287345610016

H	-9.98570465799283	8.64506586868927	8.50931244826270

c) $^{1}\mathbf{1}^{2-}$

No imaginary frequency.

Fe	-2.13345963117138	7.94622471637990	8.87044966768869
N	-3.59892055754475	8.94076086674785	9.84405001255269
N	-3.29541569706779	7.80453445220167	7.21881915107865
N	-0.68555486261932	6.89592331511760	7.92521192217655
N	-0.95735716026373	8.12127938883634	10.50365711153554
C	-3.52063969214328	9.56170417116744	11.09366062447534
C	-4.88276777958958	9.20361323221776	9.36621514268073
C	-4.60966076487264	8.25343733570756	7.08297334413205
C	-2.93976010106884	7.28094115729122	5.97322401702515
C	-0.70450094899275	6.42347623801177	6.61368950807047
C	0.29409735976861	7.53669372626889	10.69415283056128
C	-1.19969869429958	8.88665575281579	11.64644446956181
C	-4.76038395320810	10.21639770632791	11.38650637659842
H	-4.97139237044744	10.79121294016314	12.28477016105805
C	-5.60745988486758	9.98124271628009	10.32413402515097
H	-6.62562654674851	10.34226258791448	10.19774956579629
C	-5.07072726580599	8.00652871453330	5.75019383619159
H	-6.06276606309963	8.24204199997028	5.37262474239740
C	-4.02725690535892	7.43412289448577	5.05445660753205
H	-4.01947810211479	7.12711237158120	4.01152331342166
C	0.84144621889136	7.95677809653873	11.94696676148160
C	-0.08247277889490	8.79206973227310	12.53827874381557
H	-0.00577034758341	9.27584057102028	13.50845104883639
C	-5.37826440982918	8.86513648569235	8.09150089029640
C	-1.71878893580812	6.65664107364909	5.66703509253762
C	-2.39289888982748	9.57604345768312	11.93397663862681
C	-6.78584155751240	9.24321302455768	7.77158707048493

C	-7.86955756281354	8.62481343683029	8.42416877596359
H	-7.66339593811883	7.85551085866477	9.17294142484753
C	-9.19078921855539	8.97735636552059	8.12914494073317
C	-7.07781145050452	10.23455862636640	6.81469635742189
H	-6.25099770155787	10.73532523907126	6.30561301026044
C	-1.47823455501083	6.17111910568413	4.27814788413529
C	-0.41194744188763	6.69611878488527	3.52192294278242
H	0.20577315400411	7.48309323399084	3.96141399055027
C	-0.12939082768648	6.22097683489183	2.23837222164417
H	0.70472812153813	6.64605384671503	1.67293808881324
C	-0.91413545530716	5.20717453493038	1.67555305861977
H	-0.68556031564555	4.82397228093529	0.67710629261963
C	-1.98801346651299	4.68391411232290	2.40531517265005
H	-2.60785345602828	3.89140831472822	1.97609026534783
C	-2.26392521219631	5.16060868308831	3.69139785409367
H	-3.09120738338546	4.73343546366221	4.26303446213301
C	-2.46917451304265	10.30605968686986	13.23229865097084
C	-3.38605203998037	9.92943531268099	14.23401000572101
H	-4.07460390956207	9.10423014744342	14.03788380778115
C	-1.59079769107016	11.37042878085939	13.51693360509364
H	-0.87854507731215	11.68432395647431	12.74985475362938
C	-3.41692793461700	10.58117846719501	15.47056663439400
H	-4.13178464235043	10.26104586322758	16.23416742710478
C	-2.52137113618297	11.62280634204118	15.74188400453837
H	-2.53312540283970	12.12410205430380	16.71340072611971
C	-1.60956722481008	12.01795093080010	14.75591254085502
H	-0.91151183264982	12.83641435037130	14.95381958796856
C	-9.46154997171694	9.95766736615071	7.16612380049186
H	-10.49211562437712	10.23561867149643	6.92712731723869
C	-8.39649614721044	10.58641517731262	6.51012290239267
H	-8.59561416371807	11.36028804257729	5.76326254310600
H	3.58004665792328	3.95854333478338	12.54941941091483
H	5.69973582546448	4.62786121133084	11.41473782379840

C	0.48829213794867	6.39911532434432	8.49305903506784
C	0.44203727822155	5.59783896838431	6.37743845009772
H	0.66277237531502	5.08009943385125	5.44715567609049
C	1.17151219727156	5.57007154760362	7.54769630734985
H	2.09649883634447	5.03130188156716	7.73901166873244
H	1.81131213805939	7.66067405465194	12.33989279642218
C	0.95831849820931	6.68674375863256	9.78804846137213
C	2.26244315850727	6.10562074952635	10.21662099238833
C	2.33294371056524	5.19396088071452	11.28795683333521
H	1.40844879475988	4.90007308714815	11.79143518350259
C	3.55496583182110	4.66623648369801	11.71581628016181
C	4.74407241101440	5.03912112730116	11.07740097340991
C	4.69373628992992	5.94032987771794	10.00642710969469
H	5.61403358759762	6.24780096537889	9.50095908314790
C	3.46828809451991	6.46433807097445	9.58351121845763
H	3.43606909182290	7.17296402827541	8.75228058281242
H	-10.01227059666773	8.47752842063743	8.65029947491599

d) $^{3}\mathbf{1}^{2-}$

No imaginary frequency.

Fe	-2.13372515891989	7.94597946315546	8.87083475833961
N	-3.60028388477033	8.93943211794929	9.84315479400113
N	-3.29419560212478	7.80838414860672	7.21974366071770
N	-0.68611340155910	6.89480595081494	7.92813216208546
N	-0.95798697905956	8.11979166097219	10.50292859087485
C	-3.52403702216033	9.55691302238826	11.09562961746980
C	-4.88363362490607	9.20647941251328	9.36475093756581
C	-4.60790018932129	8.26057816382963	7.08053459585894
C	-2.93957050290333	7.28078558983013	5.97424268725984
C	-0.70648007256524	6.41712893625592	6.61754810049552
C	0.29654138959254	7.53969891654213	10.69324238370160

C	-1.20090661898652	8.88492957717786	11.64715048018658
C	-4.76229852765657	10.21107023951670	11.38859876386890
H	-4.97421383708503	10.78278347736556	12.28874314849059
C	-5.60868288672761	9.98135484458260	10.32340640138807
H	-6.62604695908513	10.34459508971845	10.19657213208917
C	-5.06755968976070	8.01402591273192	5.74743052320447
H	-6.05827358090122	8.25241850656113	5.36806687295748
C	-4.02506202891967	7.43589743103914	5.05470276370520
H	-4.01684936884539	7.12715847964232	4.01217918187770
C	0.84395171017171	7.96202177777458	11.94525806191817
C	-0.08249015151547	8.79397849392952	12.53705259237627
H	-0.00629560265405	9.27800489275709	13.50722382567379
C	-5.37862291225780	8.87279453049999	8.08735270343494
C	-1.72066717195313	6.65095616888242	5.66912457278743
C	-2.39584827508385	9.57030018514661	11.93772842948256
C	-6.78509606322509	9.25332243741976	7.76572531894778
C	-7.87038526368996	8.63444793625599	8.41508576194492
H	-7.66614768931208	7.86366330598391	9.16284407923195
C	-9.19076273974752	8.98861668220500	8.11830982191116
C	-7.07423881143696	10.24673741396118	6.81032982103098
H	-6.24603607455480	10.74763834557153	6.30362750594530
C	-1.48115571363306	6.16202661716123	4.28127213207244
C	-0.41470851898543	6.68421040002286	3.52351390862625
H	0.20426681972531	7.47127619322372	3.96105032309908
C	-0.13366990956273	6.20610846304351	2.24072072637846
H	0.70051261768502	6.62897165186894	1.67373743648638
C	-0.91986374980649	5.19187813989563	1.68075139860985
H	-0.69241252218642	4.80641623478987	0.68291670332533
C	-1.99375614962200	4.67120743459649	2.41227863557785
H	-2.61470429726962	3.87848436420636	1.98507945467918
C	-2.26833008843120	5.15106047595592	3.69750384430597
H	-3.09576411812327	4.72627696282392	4.27070887287196
C	-2.47384008447639	10.29748659388224	13.23749186825228

C	-3.38834241420597	9.91479580417353	14.23895522578210
H	-4.07370502483369	9.08728903661042	14.04136016640398
C	-1.59987242066910	11.36502085613823	13.5232197893168
H	-0.88975485830904	11.68353686767771	12.75613153618189
C	-3.42086453591876	10.56374829879118	15.47691329568133
H	-4.13379826509709	10.23924589906519	16.24044552672712
C	-2.52946286252559	11.60859215425012	15.74954863871389
H	-2.54255735397305	12.10782236028905	16.72211338166882
C	-1.62030478491923	12.00981343112051	14.76367282683644
H	-0.92555977866152	12.83079727696146	14.96274372046188
C	-9.45883846617508	9.97097346805595	7.15663404397313
H	-10.48871918222076	10.25019513906571	6.91616025998830
C	-8.39211044269967	10.60012686809333	6.50384725934133
H	-8.58929394614821	11.37536925954121	5.75790731656562
H	3.60005639609847	3.96514573829701	12.53924316145819
H	5.71471273028424	4.64900015650778	11.40491955445349
C	0.48839097077644	6.39730752419193	8.49548314029152
C	0.43691253517210	5.58890067956477	6.38333198455775
H	0.65619473841271	5.06822799639082	5.45422838184893
C	1.16918098546714	5.56458842597989	7.55282247427174
H	2.09424991278957	5.02593766337702	7.74454309848363
H	1.81531610487948	7.66936059937344	12.33720662847147
C	0.96251783098424	6.68971365932281	9.78885263928222
C	2.26965346624878	6.11327875590352	10.21517089140360
C	2.34511525301780	5.19848252188789	11.28310617335588
H	1.42249315110100	4.89873429302158	11.78656205628262
C	3.57029267085498	4.67539566974403	11.70799369865273
C	4.75672355274008	5.05663705466410	11.06974679655729
C	4.70115712453897	5.96115194390056	10.00181695513037
H	5.61948321355071	6.27451851541947	9.49643645742468
C	3.47262596592293	6.48021230506183	9.58172518457347
H	3.43545553961529	7.19093319792223	8.75246486770830
H	-10.01362452404776	8.48856674063028	8.63705541088321

e) ^{5}**1**$^{2-}$

No imaginary frequency.

Fe	-2.13394599289031	7.94497618198545	8.86822230068584
N	-3.60327471330404	8.93583276953402	9.84399247424869
N	-3.30503529531795	7.79157399785544	7.22105785993637
N	-0.67707245487981	6.90740880270423	7.91959514186265
N	-0.95445469413761	8.12004317794235	10.50082784196006
C	-3.51087706113340	9.58569612483925	11.07582913121045
C	-4.89526419464329	9.17922284228713	9.37385895703915
C	-4.63232481310563	8.21007744293672	7.09854259627960
C	-2.94128985445618	7.29025083848800	5.97093797003303
C	-0.68662609599107	6.46199802702227	6.60092769837894
C	0.28347634978558	7.50760454785869	10.70324158814927
C	-1.18792982242975	8.90215463072817	11.63111249924125
C	-4.74870856320196	10.24274392816169	11.36454882587208
H	-4.95161472475832	10.84093517824830	12.24935190791593
C	-5.61204135818096	9.97420849532800	10.32075122925415
H	-6.63356748997770	10.32690027117854	10.19894068305318
C	-5.09547642025603	7.96048502336115	5.76883589023138
H	-6.09501250614174	8.17327872173397	5.39801967684873
C	-4.03754874924177	7.42483944693177	5.06197539879695
H	-4.02731563016046	7.13312175351076	4.01469621130148
C	0.82869917394031	7.92135279835043	11.95717041169643
C	-0.08055462947483	8.78818431511048	12.53062319664123
H	0.00044193800522	9.27944249578829	13.49686722825149
C	-5.40225694995970	8.81057368975497	8.11230415415074
C	-1.70244085545561	6.70096125903430	5.65189193211367
C	-2.37346200441696	9.61602337339125	11.90843990787847
C	-6.81797012687970	9.16739192127806	7.80411478960482
C	-7.88689228667347	8.53758029577017	8.46954575654227

H	-7.66398162238664	7.77306168368947	9.21850736785565
C	-9.21530943850929	8.87371870494940	8.18724209115429
C	-7.13144453896100	10.15331048845192	6.84842287740965
H	-6.31579980500766	10.66296344348602	6.33031137954316
C	-1.44930198450434	6.24286586895082	4.25759827420608
C	-0.37078605639500	6.77616221773878	3.52288443862104
H	0.24473336528265	7.55241784750443	3.98395067368331
C	-0.07349288403055	6.32365510225154	2.23473284108618
H	0.76960988445441	6.75612093073616	1.68839002490618
C	-0.85524237825733	5.32448717996822	1.64194979885032
H	-0.61496684571872	4.95816208559543	0.63992001017114
C	-1.94040604804541	4.79283076310702	2.34888247599250
H	-2.55759455543731	4.01033491112263	1.89792562371338
C	-2.23118483665669	5.24667093012550	3.63963839165811
H	-3.06600757641518	4.80957601623016	4.19228399244043
C	-2.43557292987489	10.37125862327601	13.19150842477677
C	-3.36778300392603	10.04186685343330	14.19715624636125
H	-4.07826698909444	9.23229615367409	14.01509459092422
C	-1.52710833598604	11.41477744959474	13.46353724083814
H	-0.80226073517018	11.69439198173474	12.69499420821252
C	-3.38486957879463	10.71687394976140	15.42124245524067
H	-4.11162647654782	10.43054303877298	16.18717264682427
C	-2.46078826792821	11.73715260378127	15.67857929976685
H	-2.46202183702009	12.25626493346461	16.64075387281715
C	-1.53278461858338	12.08583227509659	14.68998782108411
H	-0.81178600941356	12.88707704401773	14.87606906784215
C	-9.50759791695473	9.84874657786446	7.22523853948447
H	-10.54386819752922	10.11386625205365	6.99667428565656
C	-8.45719661044460	10.48900929807234	6.55692941842357
H	-8.67326140288879	11.25915430270105	5.81095795085742
H	3.47276856059898	3.82810156731150	12.55013900854543
H	5.62385313719902	4.50283419741149	11.47862739800418
C	0.48603926233082	6.38900396863178	8.49444716604349

C	0.45899435455572	5.63838284004911	6.36177614935414
H	0.68846258059610	5.13915865592728	5.42341312911022
C	1.17576772149708	5.57790396789250	7.54095675185166
H	2.09650598649701	5.03142054550389	7.73041607974186
H	1.78812190469546	7.60558802665093	12.36011872929751
C	0.94076851640495	6.64989424314834	9.80001523290662
C	2.22930266300959	6.04265320619519	10.24050464239862
C	2.26641327886381	5.10562534196904	11.29083654285851
H	1.32787621018349	4.80995437195346	11.76675338222104
C	3.47347614345209	4.55533631589525	11.73315085852659
C	4.67984530055774	4.93123741548966	11.13005952581320
C	4.66248129469854	5.85776741283394	10.07983859201545
H	5.59675293769093	6.16754491539183	9.60208324455614
C	3.45183643892063	6.40426621524136	9.64266562541103
H	3.44496350680134	7.13297125703046	8.82835971548732
H	-10.02554176703195	8.36556047720033	8.71777372373580

f) ^{1}**TS1**

One imaginary frequency of -227 cm^{-1}.

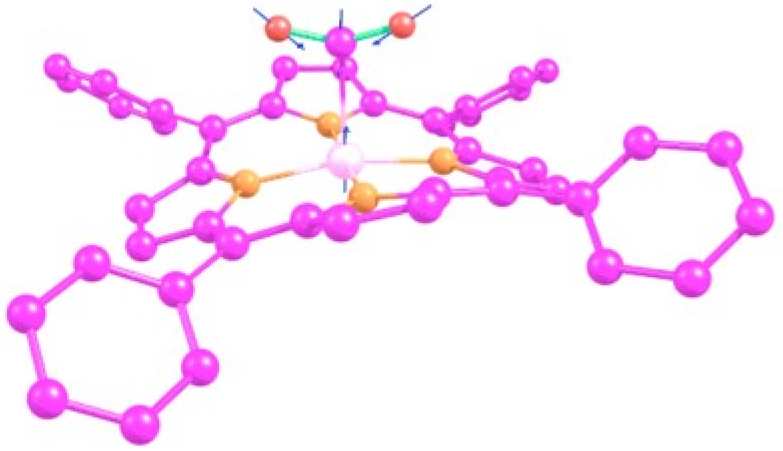

Figure S17: Normal mode of vibration associated with the imaginary frequency of -227 cm^{-1} in ^{1}**TS1**. Blue arrows represent atom displacement vectors. For clarity, all hydrogens are omitted.

Fe	-2.411165000	7.973512000	8.569828000
N	-3.966504000	8.879258000	9.376227000
N	-3.348200000	7.869473000	6.842731000
N	-1.092220000	6.625218000	7.944368000
N	-1.425018000	8.153376000	10.264678000
C	-4.153054000	9.198592000	10.721308000
C	-5.162204000	9.195160000	8.734613000
C	-4.418520000	8.675735000	6.445753000
C	-2.852821000	7.285479000	5.673941000
C	-1.162127000	5.873178000	6.772569000
C	-0.104140000	7.758008000	10.496213000
C	-1.790034000	8.933099000	11.364393000
C	-5.528201000	9.539934000	10.942006000
H	-5.974599000	9.770035000	11.906063000
C	-6.146916000	9.554548000	9.711122000
H	-7.182501000	9.806484000	9.493143000
C	-4.481604000	8.714450000	5.015390000
H	-5.183802000	9.305963000	4.432406000
C	-3.518411000	7.846513000	4.539618000
H	-3.290453000	7.605023000	3.503142000
C	0.403454000	8.445052000	11.645704000
C	-0.642273000	9.161572000	12.189618000
H	-0.623980000	9.774811000	13.087372000
C	-5.340815000	9.247693000	7.338311000
C	-1.917843000	6.234991000	5.642851000
C	-3.110921000	9.321609000	11.660908000
C	-6.552452000	9.927614000	6.805985000
C	-7.422264000	9.294925000	5.894697000
H	-7.202325000	8.274656000	5.573296000
C	-8.559909000	9.943657000	5.405227000
C	-6.874339000	11.241093000	7.208347000
H	-6.208005000	11.758230000	7.902510000

C	-1.727884000	5.471826000	4.379082000
C	-0.452404000	5.303509000	3.802987000
H	0.405688000	5.792161000	4.269322000
C	-0.272115000	4.524662000	2.656315000
H	0.729451000	4.400625000	2.234300000
C	-1.367388000	3.905024000	2.044015000
H	-1.225372000	3.292989000	1.149280000
C	-2.645762000	4.082562000	2.586290000
H	-3.509760000	3.603819000	2.116777000
C	-2.821405000	4.855030000	3.737472000
H	-3.819080000	4.971789000	4.167012000
C	-3.410146000	9.863240000	13.014553000
C	-3.006490000	9.166941000	14.173487000
H	-2.460772000	8.227713000	14.060299000
C	-4.113772000	11.071568000	13.198255000
H	-4.424541000	11.645596000	12.323234000
C	-3.301876000	9.644600000	15.452475000
H	-2.975906000	9.075383000	16.327712000
C	-4.018493000	10.836374000	15.612975000
H	-4.262967000	11.210952000	16.610905000
C	-4.420949000	11.546495000	14.476329000
H	-4.972392000	12.484649000	14.586048000
C	-8.866255000	11.244150000	5.822482000
H	-9.756253000	11.752290000	5.441612000
C	-8.017110000	11.887751000	6.730733000
H	-8.245631000	12.903192000	7.067941000
H	3.414464000	5.013725000	13.090610000
H	5.393407000	5.071751000	11.573197000
C	-0.015640000	6.114441000	8.663427000
C	-0.233177000	4.781485000	6.846911000
H	-0.112482000	4.004826000	6.095219000
C	0.486143000	4.939958000	8.011378000
H	1.292933000	4.312086000	8.382624000

H	1.418914000	8.369570000	12.027967000
C	0.548426000	6.725691000	9.797959000
C	1.872269000	6.247530000	10.284066000
C	2.051436000	5.787289000	11.603887000
H	1.189319000	5.748884000	12.273066000
C	3.304486000	5.372228000	12.063180000
C	4.414511000	5.398125000	11.211356000
C	4.252252000	5.834281000	9.891321000
H	5.110116000	5.861750000	9.213714000
C	2.998443000	6.252878000	9.436676000
H	2.882441000	6.610234000	8.410505000
H	-9.218927000	9.425371000	4.702994000
C	-1.495826000	9.912826000	7.584272000
O	-2.291694000	10.789892000	7.722109000
O	-0.427296000	9.573846000	7.171276000

g) [1]**A**

One imaginary frequency of -16 cm^{-1}.

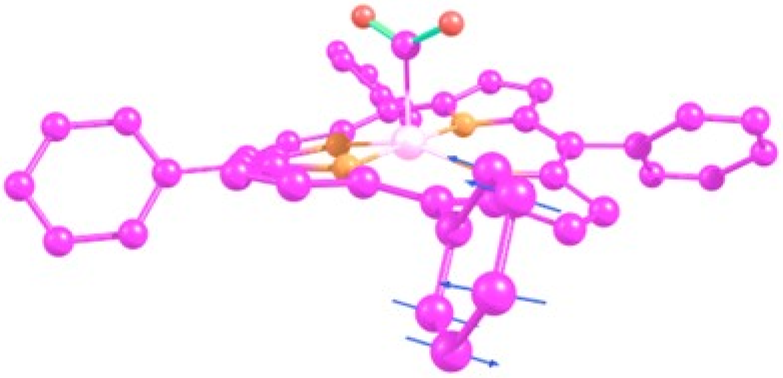

Figure S18: Normal mode of vibration associated with the imaginary frequency of -16 cm-1 in [1]**A**. Blue arrows represent atom displacement vectors. For clarity, hydrogens are omitted.

Fe	-2.32653192316301	8.05268331501034	8.55516160410301
N	-3.85625451076375	9.02662595955405	9.36591926387377
N	-3.27293793825177	8.04322098623113	6.78958967296727

N	-1.18890352228204	6.52935630296807	7.93302969663149
N	-1.43177830679122	7.96919914875901	10.34476099316225
C	-3.99323327300529	9.41329393155318	10.69491811723284
C	-4.97579531734173	9.51775765651599	8.70586446811783
C	-4.30025746487031	8.88939901198373	6.41147487294073
C	-2.83317018408663	7.43377209165763	5.62690285716463
C	-1.23656883308747	5.88899214980528	6.70262432240157
C	-0.22758279531647	7.35199946244787	10.63576111907266
C	-1.76331932699288	8.69316585668283	11.47464462706585
C	-5.29164468051422	10.02507175341145	10.89242156896297
H	-5.68039089881501	10.37529479626999	11.84626802095160
C	-5.89496555446826	10.08942505154947	9.67335862615965
H	-6.87493222061627	10.49899350265372	9.44110679457853
C	-4.42108293412981	8.90601497233009	4.98483480938201
H	-5.12085592370228	9.51279441499852	4.41545797434500
C	-3.50361856346105	7.99366733193816	4.49549046085435
H	-3.31627257879936	7.72173533635314	3.45878735348993
C	0.25599231913544	7.77944822684850	11.91229130392246
C	-0.70641936556603	8.61906647342322	12.43899324751487
H	-0.69681112280108	9.11708339122336	13.40560464092275
C	-5.16814318437059	9.54379667966290	7.32520351629249
C	-1.93285026004709	6.33871540010821	5.58490983044476
C	-3.01462225520574	9.32531590845887	11.67837017317570
C	-6.35987992374478	10.25946820052721	6.78291597136265
C	-7.30412493712480	9.59519059840957	5.97515025780826
H	-7.15091585552591	8.54035617063345	5.73688910310693
C	-8.43128066627342	10.25875343601086	5.48294072946684
C	-6.58623594921588	11.61908996017475	7.07316750999538
H	-5.85900209748607	12.15937666519162	7.68348347468829
C	-1.73993401719660	5.61242612131791	4.29658499285320
C	-0.46413305352085	5.50259224160899	3.71177312154789
H	0.37966711878847	6.00931576803896	4.18557893882689
C	-0.26427342901175	4.74859110921193	2.55144615486244

H	0.73841846410496	4.66454154932502	2.12290047407285
C	-1.34203398486656	4.10061376920270	1.93900343812399
H	-1.18519254138937	3.50708220908021	1.03439103417899
C	-2.62199444556449	4.22194170049026	2.49309579136923
H	-3.47101327555645	3.71838561122762	2.02276837898089
C	-2.81745831236350	4.97070065406357	3.65688749623436
H	-3.81505560457457	5.04423479873574	4.09634673738083
C	-3.26688780955303	9.92030934011067	13.02505538545541
C	-3.32627376052389	9.10694018109865	14.17160734631073
H	-3.23104631588245	8.02445230384475	14.05726040647392
C	-3.39934906075389	11.31013448533583	13.19122513284513
H	-3.34952006249530	11.95640367309889	12.31153148348209
C	-3.49371746277759	9.66244307725811	15.44307544466479
H	-3.53343605659435	9.01000509670176	16.31952086500482
C	-3.61016699021460	11.04918579590233	15.59411607798004
H	-3.73531779636931	11.48884955999780	16.58739494135752
C	-3.56932686055468	11.86974780797047	14.46209152845478
H	-3.66173181277705	12.95387310021989	14.57053044888062
C	-8.64677957864303	11.60714105805339	5.79201915420535
H	-9.52893540440435	12.12772160275619	5.40980019927779
C	-7.71977746369907	12.28259795356498	6.59283040188772
H	-7.87905066458206	13.33458983999276	6.84630358916885
H	2.56963095809072	3.26763628726567	12.62817027554726
H	4.81243744569354	3.94030921794344	11.76443062129853
C	-0.17879930930234	5.89794792962872	8.64697771453700
C	-0.34856503373935	4.73854870196420	6.71371676042910
H	-0.23880997598346	4.02324425350445	5.90245278947488
C	0.31529018891403	4.75700365232214	7.90262321968876
H	1.07470952770979	4.06347708180390	8.25771110745024
H	1.18694248231800	7.45603105157346	12.37307715064637
C	0.34981143387709	6.32628401988857	9.85536487305026
C	1.57713796090024	5.65404738938211	10.38058438746919
C	1.49757436216050	4.66028173722982	11.37009248005760

H	0.51650405929478	4.36673848049288	11.75320477160005
C	2.65272986152639	4.04395481505023	11.86258749983831
C	3.91012104954974	4.42008692269841	11.37552844710330
C	4.00286968058276	5.40956472405858	10.38952375294331
H	4.97905567875275	5.71291801458530	10.00004798441593
C	2.84408129781765	6.01783383272789	9.89504811512882
H	2.91470422957419	6.79028014759537	9.12472453386705
H	-9.15144865957452	9.71688200232848	4.86368568214172
C	-1.23734634860451	9.64709812829621	7.96546363555629
O	-1.73409102853138	10.75015703806171	8.21037792857527
O	-0.17622320236627	9.34110649704202	7.41055753713941

h) ^{3}**A**

No imaginary frequency.

Fe	-2.28283766202117	8.06088628131781	8.56363448904804
N	-3.90952542329784	9.07914109577848	9.42049616141754
N	-3.39970935189131	7.93877426625570	6.77662393859393
N	-1.08772757420510	6.50318365296516	7.88679993537160
N	-1.49713169743520	7.78009442601406	10.48676986496510
C	-3.94021965521566	9.57721664480448	10.71785755816325
C	-4.96232796375830	9.64622993450869	8.73907788856973
C	-4.54860506457422	8.62768388611622	6.47569912583006
C	-3.04584397435414	7.21856394875011	5.64735272168393
C	-1.05724051545035	6.00513894295883	6.59089441927298
C	-0.37172906235277	7.06005307282653	10.79794766048451
C	-1.88415996326748	8.45620732434368	11.63194958057668
C	-5.05555345444666	10.49521603440714	10.84333461840478
H	-5.31890040028252	11.04692551489830	11.74292126976566
C	-5.68638500082952	10.53767676580813	9.63253232174972
H	-6.55515053309256	11.13246820291806	9.35980906690250
C	-4.93090403819273	8.35335562674508	5.11504633163506

H	-5.80693369768595	8.75267859844153	4.60914562761230
C	-3.98744195509799	7.48464662336404	4.59976277709070
H	-3.96071737529980	7.06064843389025	3.59824880038963
C	-0.01735596067799	7.29465757622080	12.17405613707103
C	-0.96662024353443	8.15532813806825	12.69203183217516
H	-1.02466314216524	8.53702568789306	13.70922286312918
C	-5.27817812906403	9.45311271562519	7.38283568333043
C	-1.93434556452222	6.33965570429429	5.55464430592432
C	-3.02316037002741	9.29675944232574	11.73724081525309
C	-6.49063872449461	10.13931832601754	6.84643638032500
C	-7.77803171830505	9.78601591014847	7.29071071822185
H	-7.88148087096831	8.99962321993982	8.04261778488686
C	-8.91697581194407	10.41916792098203	6.78280266117118
C	-6.38226198386694	11.15135128479959	5.87490661831094
H	-5.38970517444862	11.44238805003933	5.52114647968657
C	-1.66501353037411	5.72224658421012	4.21844960747372
C	-0.55736564043117	6.14096579975988	3.46163088353463
H	0.08298459247655	6.93429534584877	3.85490924829416
C	-0.26729806357600	5.55028801022089	2.22787642058742
H	0.59876907023850	5.89010658075720	1.65335706199215
C	-1.08405626137706	4.52872382190627	1.73020937574739
H	-0.85127947417800	4.05903640930729	0.77063692633857
C	-2.19836725775845	4.11183997531197	2.46763045023833
H	-2.84331185548416	3.31647473495115	2.08393573335582
C	-2.48630507317607	4.70661521812704	3.70097381951282
H	-3.35072889331276	4.37386911607967	4.28101653953063
C	-3.26812638808095	9.94310155807065	13.06393512475374
C	-4.36403805050152	9.55869789132866	13.85528255838217
H	-5.02305391425649	8.76355261903920	13.49841406724223
C	-2.42392394810376	10.96241213132524	13.53775387786493
H	-1.56893506171570	11.26914767463268	12.92992382578802
C	-4.62038132194920	10.18628128153924	15.07872961380971
H	-5.47775480126295	9.87295923087302	15.68067772826458

C	-3.78106107293751	11.21010763669751	15.53198581509922
H	-3.98695577533508	11.70710101992363	16.48387225610609
C	-2.67818698370108	11.59386881214319	14.75980122112593
H	-2.01646869374277	12.39189239994840	15.10790649089278
C	-8.79152555184837	11.42167566907867	5.81356590135460
H	-9.67869444046615	11.91837217679026	5.41142891601860
C	-7.51733590554466	11.78659901731453	5.36198144780807
H	-7.40496897465461	12.57350081853478	4.61082288483078
H	2.77147659785460	3.22153694284143	12.57252815005504
H	4.95794111500694	4.04005223122803	11.69320993250682
C	-0.02856356001082	5.95036913907704	8.56650699445265
C	0.04183115451417	5.06553463021800	6.47540559399982
H	0.29382057358559	4.49904404039647	5.58158422003212
C	0.67550807262518	5.03081469903882	7.68537473836380
H	1.53772245807964	4.42925657102237	7.96497697927621
H	0.83292587667303	6.85944115583791	12.69401542096807
C	0.33876513670611	6.21525393567901	9.89589601490585
C	1.60395740832998	5.59856042465274	10.39583810909485
C	1.60112112293020	4.57598688959611	11.36024862834056
H	0.64511627311265	4.21468321665268	11.74801497198641
C	2.79734220705127	4.01892993080427	11.82459429816013
C	4.02382876273413	4.47640355690928	11.32855815816600
C	4.04316479353956	5.49343304399295	10.36614986500749
H	4.99412200159236	5.86300097480770	9.97119952363242
C	2.84441976585440	6.04853765426418	9.90806661431095
H	2.85713615841142	6.84915217061597	9.16384057225244
H	-9.90506408633229	10.12055542791805	7.14560588090782
C	-0.96117535029501	9.62441452939699	8.07184058758226
O	-1.60003515289890	10.64292316172751	7.79044261636553
O	0.23352107276010	9.33582453013427	8.13866007167016

i) **^{5}A**

No imaginary frequency.

Fe	-2.44925297143453	7.83354334753761	8.65107863134254
N	-3.75601249699205	9.18450877620540	9.38962923413613
N	-3.31399476577234	8.01239288313910	6.83889653313988
N	-1.15828406420659	6.46299112081167	7.92115556173082
N	-1.55932408673422	7.68586381911249	10.45478043621121
C	-3.78946357931223	9.66597248798274	10.69794589782506
C	-4.91987835351378	9.65253905356847	8.77315582895033
C	-4.39594289990766	8.82397110633395	6.49500252439220
C	-2.81048294637433	7.51107774424803	5.63684519324768
C	-1.08706045053076	6.01447579392868	6.60417151099591
C	-0.48602544247544	6.86228499072501	10.79451405325207
C	-1.73062094936232	8.55718993379818	11.53235353438617
C	-5.00567479993364	10.37841740858195	10.91673547014375
H	-5.30698448246972	10.83574650564380	11.85681907883705
C	-5.71539530539100	10.35361621212709	9.72948310113296
H	-6.69240062470595	10.79126808624168	9.54075752308374
C	-4.52508865805650	8.87216512757525	5.07230759719725
H	-5.26165050736854	9.45808012994222	4.52803328026876
C	-3.53461251075876	8.06698733628500	4.54024360553268
H	-3.32774721149496	7.87112166962983	3.49018212973793
C	0.05109392390350	7.26174034110957	12.05428280715533
C	-0.70622754364849	8.32919372611141	12.50273588917394
H	-0.56805877012019	8.89677912692811	13.41988433708031
C	-5.23034046599794	9.51787700589589	7.40016325137137
C	-1.78340574426197	6.55925511372433	5.51038504177230
C	-2.79270943884122	9.47208368868658	11.67881005460285
C	-6.45617579077443	10.16804503791243	6.86494522273737
C	-7.39220071611919	9.43427894635614	6.10235047441274
H	-7.20470381295949	8.37454621773506	5.91499110371410
C	-8.54530699441612	10.03070478751100	5.58611372497992
C	-6.73711852021958	11.53447947034198	7.08263711933503

H	-6.02298724404998	12.13861342833484	7.64598930993702
C	-1.44938748755939	6.02807179970898	4.15861526956043
C	-0.18173434977656	6.25737666272087	3.59099482895662
H	0.53623774336240	6.87303344299271	4.13848606316929
C	0.16854821150974	5.70947311523040	2.35345789305470
H	1.15754339442108	5.91038731478031	1.93137528893145
C	-0.74331159477174	4.91024252676062	1.65439228705366
H	-0.46582932555829	4.46801805851123	0.69352704298602
C	-2.01292438082240	4.68086809951230	2.19837157082294
H	-2.73316509484173	4.05513810003985	1.66386051687638
C	-2.36080943695879	5.23801233971678	3.43313326335867
H	-3.34790465189350	5.04294603674685	3.86048435329404
C	-2.89911909118766	10.23458207968605	12.95191354288353
C	-2.99950454163308	9.57849492227339	14.19576373184902
H	-3.03431138420583	8.48678275336904	14.21329447423350
C	-2.88811213211725	11.64424085615941	12.95955406204203
H	-2.81340052981628	12.17649514646045	12.00780158026043
C	-3.05223984021875	10.29408443689132	15.39503694976989
H	-3.12255685618890	9.75459309816707	16.34400085656614
C	-3.02631274143621	11.69366122362321	15.38243740564123
H	-3.06612712726468	12.25757053521791	16.31818471915178
C	-2.95341175548340	12.36438728534840	14.15560573687299
H	-2.93309719958009	13.45777087775283	14.13074732915683
C	-8.81003520430338	11.38456166160048	5.82602374589042
H	-9.71431858131163	11.85277749733807	5.42772352920249
C	-7.89822191820113	12.13077218381533	6.58188223548694
H	-8.09067797659165	13.18943433573265	6.77972327435906
H	1.16975098142312	2.59391889488668	13.20098594028175
H	3.46499886241159	2.27117644919636	12.25448101510680
C	-0.30297982638833	5.63596386658092	8.65425507372292
C	-0.22418566362493	4.87830792144683	6.52395620402439
H	-0.00749061335268	4.31844608474994	5.61716299841515
C	0.24210165865713	4.62714047606794	7.80011082810356

H	0.91312341301497	3.83129715646023	8.11352035875225
H	0.91429582397404	6.80873554527238	12.53738185196210
C	0.03256720462925	5.81214114806900	10.00947654790778
C	1.00036106739915	4.87164649235960	10.63679624495790
C	0.62456221763283	4.10724679105267	11.76064582853745
H	-0.37921212743334	4.23722668591873	12.17248696686582
C	1.49745352751104	3.18216689132994	12.33900984320233
C	2.78055473366955	2.99789556353534	11.80916497189913
C	3.17916750546431	3.75969461020996	10.70439486274231
H	4.18151512720859	3.63085831704261	10.28517253346690
C	2.30355763192266	4.68731159500674	10.13151663740425
H	2.62804609412771	5.28222633671217	9.27435443948070
H	-9.25027489138463	9.43007256316867	5.00398042150742
C	-0.45192061771453	10.34922762074372	7.75083464727425
O	-0.61229524642011	10.80688329541709	8.80487237480845
O	-0.26718078599707	9.92345888054801	6.68716079632670

j) **[1]B**

No imaginary frequency.

Fe	-2.68871830954791	8.48319916510937	8.62129059912383
N	-4.31069766549141	9.31482875675880	9.46317997739052
N	-3.64574995627189	8.44438552844061	6.85535593483150
N	-1.29666231397990	7.24606058129588	7.86684484991340
N	-1.86141714662810	8.28095751620214	10.43771086002186
C	-4.54177864757531	9.53717364463882	10.82273424571945
C	-5.43599125001002	9.77397586450916	8.81218371509945
C	-4.75477858138556	9.18888975546481	6.51024388279032
C	-3.19451327558352	7.86610960576885	5.66685982267825
C	-1.27129565922286	6.65418142256171	6.60234554290251
C	-0.58267839963410	7.83842928669910	10.70261920384725
C	-2.33160614508907	8.81621564516910	11.63744523054515

C	-5.85991383835029	10.06764711632441	11.01728164136986
H	-6.30521142107284	10.30598616678486	11.98058546735455
C	-6.41746805225303	10.21880140774397	9.77450631222453
H	-7.40731517801070	10.59637552373490	9.53132361695105
C	-4.94047088811699	9.16578828315773	5.07760528287843
H	-5.70852240007057	9.70650095141174	4.53171860042321
C	-3.98386713367791	8.32722777641457	4.56413749067988
H	-3.82075969832683	8.05620308329974	3.52349912771911
C	-0.21510062482643	8.15785545584712	12.06121401340549
C	-1.30696535293672	8.74910812046721	12.64111934471422
H	-1.40215174291710	9.12124645563837	13.65851893740268
C	-5.63120073948638	9.81523841032348	7.41675738411021
C	-2.13296961116463	6.96609436360084	5.55115792126294
C	-3.60645207656072	9.34964015934726	11.84366837173367
C	-6.85293558560437	10.49077618148518	6.89451162945023
C	-7.79196410523831	9.80542394326378	6.09713564770819
H	-7.61844069326552	8.75435462273485	5.85562795362274
C	-8.93850941199658	10.44285999398722	5.61449513447320
C	-7.11735023770918	11.84130718154292	7.19805006554881
H	-6.40085486256064	12.39542171333924	7.80934885715013
C	-1.88368528418676	6.32161114129512	4.22681846441595
C	-0.71453178345578	6.60855087495829	3.50270448072209
H	-0.00733789068401	7.33390190473279	3.90241317864939
C	-0.45927002573629	5.97987327547674	2.28072702592989
H	0.45221747024217	6.22494547101054	1.72861152759345
C	-1.36559561397366	5.04566315872075	1.76636766456325
H	-1.15895629242935	4.54513630920819	0.81632963123655
C	-2.53832948997381	4.75789630622439	2.47438531858610
H	-3.25345065624294	4.03110748030332	2.07926920060207
C	-2.79674641929641	5.39636055977290	3.69254298150963
H	-3.70978995879069	5.16647708304599	4.24759960127934
C	-3.96509604599501	9.76189470182506	13.23417804241995
C	-4.08113603909415	8.80244870230057	14.25465759913330

H	-3.93931612644853	7.74705311294365	14.00897680512755
C	-4.15766517417061	11.11473010144777	13.56268167718341
H	-4.06465946022010	11.87029090201162	12.77894190722995
C	-4.36895186515949	9.18444960235953	15.56822408700581
H	-4.45689403102140	8.42317694668007	16.34784569934397
C	-4.54529857896699	10.53574939874026	15.88509812699666
H	-4.76691920867250	10.83798240999074	16.91221906828450
C	-4.44190291163623	11.50001887323399	14.87665541455308
H	-4.57852716057162	12.55804698724905	15.11688347881052
C	-9.18554567163924	11.78389498997499	5.92966354113225
H	-10.08267563255333	12.28377430242077	5.55461745973945
C	-8.27086532775001	12.47776412786377	6.73030161812729
H	-8.45634259791109	13.52356623282415	6.99160040019927
H	3.18429023482196	4.61760877726880	12.46278049395778
H	5.18760654768340	5.55481032434228	11.31604349432254
C	-0.17931109391081	6.77469870560689	8.51665072781196
C	-0.16484758976585	5.74312093885626	6.50586887675706
H	0.06608092880089	5.12588823996039	5.64105620765305
C	0.52468010094628	5.82585688966923	7.68590254317762
H	1.41933652660576	5.28173272671823	7.97828283529782
H	0.74837688973327	7.94614715787306	12.51780720023964
C	0.23490452522137	7.12836557806031	9.81089605219322
C	1.59224139393691	6.69507910346062	10.24715312782304
C	1.77844855563251	5.75954130446575	11.28082420070796
H	0.90223476351019	5.34240684988638	11.78301992089622
C	3.06098969202202	5.35222521323365	11.66210291868769
C	4.18638817490733	5.87676030877124	11.01554426087206
C	4.01696409662119	6.80878982459936	9.98361648362641
H	4.88777375496881	7.22767649198691	9.47138498285856
C	2.73319983759529	7.21088977746625	9.60311978199627
H	2.59670211187431	7.93307964895706	8.79403248934307
H	-9.64955108703228	9.88484277815291	4.99874202226525

C	-1.72338814526970	10.18810020842181	8.28120292267196
O	-2.40883359529141	11.14015762082270	7.90490448639397
O	-0.48197122951529	10.15649924581993	8.46948486982720
H	0.46302989219112	9.52454034031638	7.37593472760431
C	0.82184227620164	9.49025594213616	5.45033820438269
C	1.76160579586073	9.22108824891604	4.43528605616680
C	-0.44077884480851	10.00228003131425	5.08644098628178
C	1.45131171546237	9.47059820338219	3.09632418011467
C	-0.73954008731879	10.23930718292102	3.74308064886874
C	0.19866583576981	9.98314678265252	2.73675017881696
H	2.73296027776607	8.81035323225610	4.72328641780910
H	-1.18867717205164	10.20637207676220	5.85238136687194
H	2.19636624466368	9.25371787054053	2.32569490739789
H	-1.72833821128247	10.62722414273514	3.48219506186228
H	-0.04737046363347	10.17553972547726	1.68937313116281
O	1.17832712798866	9.24950886649932	6.72280256785072

k) **^{1}C**

One imaginary frequency of -2 cm^{-1}.

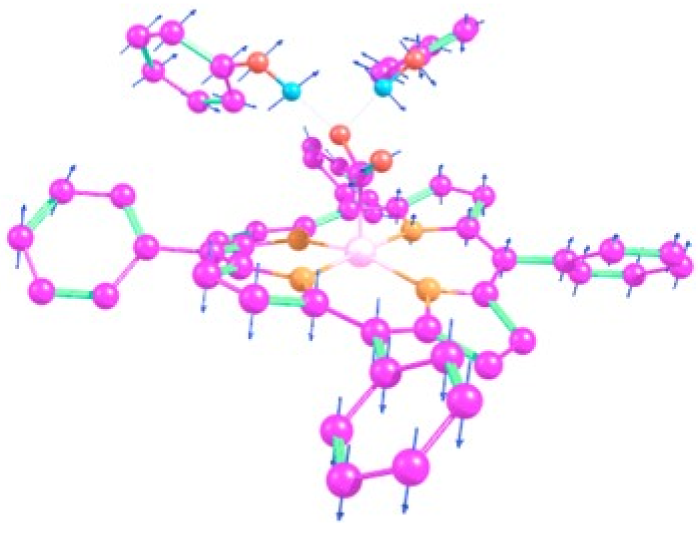

Figure S19: Normal mode of vibration associated with the imaginary frequency of -2 cm^{-1} in **^{1}C**. Blue arrows represent atom displacement vectors. For clarity, all hydrogens are omitted, except the hydrogens involved in the formation of the H_2O molecule.

Fe	-3.48266755486526	8.41060416211967	8.07347373079929
N	-4.99811024170932	8.93143934068084	9.24654787667734
N	-4.71999278466925	8.30538782327604	6.51950594317539
N	-2.14318632388221	7.34930196745166	7.07962524863282
N	-2.28067092904750	8.44886549393715	9.65990938667089
C	-4.97182727813796	9.11281255396282	10.61681802056488
C	-6.33350400219227	8.99525995867636	8.88565875721828
C	-5.99164642524148	8.83356323597336	6.45236445953513
C	-4.34969780599206	8.03423835181693	5.21617469258872
C	-2.24447008811799	6.89108805494794	5.78106543841913
C	-0.94469996899288	8.10467812751134	9.68129127614556
C	-2.52650213259182	9.08097982218490	10.86586539919830
C	-6.32098279028490	9.13567785432028	11.14113981627816
H	-6.59076795212514	9.19081371094927	12.19296418068861
C	-7.16324168061825	9.06434539729750	10.06976391532436
H	-8.25034313014399	9.04840678730899	10.08157590896858
C	-6.38330638024938	9.00554733306432	5.06924461211739
H	-7.31622126873690	9.44500493987523	4.72551881800477
C	-5.36381072348246	8.51391986633101	4.30336805658413
H	-5.30957833365872	8.47017247556525	3.21813635530317
C	-0.30507618791954	8.66982157436180	10.84989865044468
C	-1.28180900201819	9.28552839612380	11.57797591544307
H	-1.16816265054497	9.82083635922974	12.51709793619549
C	-6.80831614608678	9.08177439260502	7.56806813677531
C	-3.20768668868504	7.30907692364442	4.85127054140287
C	-3.80957835953444	9.32657684706214	11.37716569600182
C	-8.23510660149716	9.45695474835250	7.35399886742403
C	-9.08613242220365	8.67605567113882	6.54826825607681
H	-8.69604555201960	7.76803823032689	6.08389848912171
C	-10.41911321182260	9.04218856226372	6.34325555730737
C	-8.77344380258759	10.60944460451996	7.96050412676782
H	-8.12886326311726	11.23098065068439	8.58560641979171

C	-3.00680926301604	6.93108383674373	3.42200597670776
C	-1.84784092456949	7.34270634482234	2.73702795656636
H	-1.11755183677607	7.96481357199993	3.25549645555985
C	-1.62524876595068	6.95761287696111	1.41229240885876
H	-0.72000825135298	7.29320806414889	0.89909647106683
C	-2.55413532137496	6.14809909419030	0.74858365796831
H	-2.37113714505939	5.83134995575465	-0.28187534759537
C	-3.71558637277300	5.73959882111437	1.41489216000762
H	-4.44632685147778	5.10531635170877	0.90571237404300
C	-3.94083712676065	6.13185700405659	2.73821289866063
H	-4.84094914858340	5.79913736515881	3.26015852822746
C	-3.96491843325632	9.78197621302314	12.78854665162460
C	-3.35369774695095	9.09323934798246	13.85431373629015
H	-2.74845553066140	8.21014160984831	13.64039364073414
C	-4.75540048832584	10.90629661383847	13.09858905409184
H	-5.23085399860376	11.46268939317475	12.28854885154413
C	-3.52589262265130	9.51092487563162	15.17707265640954
H	-3.03965237758850	8.95681878345654	15.98442516960338
C	-4.32546208241001	10.62203042197813	15.46768505055368
H	-4.47018250762504	10.94753029310439	16.50158960231106
C	-4.93986456068306	11.31793351202862	14.42090889209740
H	-5.56143916260575	12.19144531968313	14.63528340170329
C	-10.93619409001529	10.19267706625245	6.94904155604123
H	-11.97645990972478	10.48474652284004	6.78128672359783
C	-10.10842848954747	10.97214039627075	7.76505826103733
H	-10.50240711940625	11.87132550692298	8.24684007117666
H	2.82914149917104	5.13863908517378	11.48819099259326
H	4.67986823880767	5.52476159574272	9.86325392044142
C	-0.95163406387593	6.84115541629144	7.56627220891649
C	-1.16475612514848	5.97153081314407	5.49038731525719
H	-1.02825851858184	5.43452534601910	4.55516966706962
C	-0.37410294873547	5.92701127183119	6.60069511477398
H	0.52867478442209	5.34043676893126	6.74742784756876

H	0.75664517331135	8.61969584603926	11.07535721815898
C	-0.32680577433140	7.25450148796072	8.75246144002437
C	1.05176160122462	6.76913278163223	9.04979946165800
C	1.33643536891901	6.09947662642837	10.25545896087582
H	0.52824290874634	5.91773709940941	10.96705301055798
C	2.63055856536875	5.65889912860908	10.54725436549175
C	3.66863906727290	5.87189713747351	9.63407966722678
C	3.39904669399188	6.52490072681420	8.42624364953002
H	4.20286971037534	6.69898309058705	7.70626612894010
C	2.10549876019068	6.97096133017454	8.14026302834371
H	1.90593734522546	7.50569705187832	7.21045283743372
H	-11.06023511661115	8.41947674273528	5.71373785130083
C	-2.91619448804926	10.13801642946407	7.46219417836195
O	-3.69693346274276	11.09948884787793	7.41223376624911
O	-1.66323130854476	10.26655439966143	7.10250686744522
C	0.53218922644061	11.74546311733680	9.01362357969743
C	1.07846381560955	12.46982295241847	10.09287306855097
C	1.25202677729497	10.64557996775473	8.50324928656455
C	2.29966479404172	12.09201977186249	10.65660951691739
C	2.47352215540973	10.28221748578335	9.07176672933388
C	3.00670796436821	10.99156655354178	10.15442597380173
H	0.51424442069802	13.31815448548075	10.48960223580681
H	0.82598561234158	10.06611495653718	7.68395842591279
H	2.70126913603841	12.66325488599028	11.49891007613535
H	3.00803829704679	9.41962347806468	8.66986454000567
H	3.95588301312867	10.68551477581041	10.60218811154734
O	-0.65148732988227	12.12993020989221	8.51001479330782
H	-1.03517077239683	11.41337515721833	7.88873697315316
C	-0.00014373675654	10.36535165025483	4.30204625912777
C	0.49642757898483	10.65811301513455	3.01574788196703
C	0.46038949437252	9.20367781442689	4.95731734792081
C	1.43149949254504	9.81752027679863	2.40847765410684
C	1.39214229418321	8.36865514246744	4.33714836492310

C	1.89077574922242	8.66588770115710	3.06264974581087
H	0.12373771338334	11.55093569099079	2.50672029554766
H	0.04858149889947	8.95134783845555	5.93478934518463
H	1.80804611620432	10.06876812003232	1.41223923479290
H	1.71511247941400	7.46299070343996	4.85733107132735
H	2.61758248531975	8.00697770738610	2.58159389252452
O	-0.89496892512723	11.20513160689046	4.84678683246132
H	-1.22581445066277	10.85714653073198	5.74542092081308

l) **[1]TS2**

One imaginary frequency of -138 cm^{-1}.

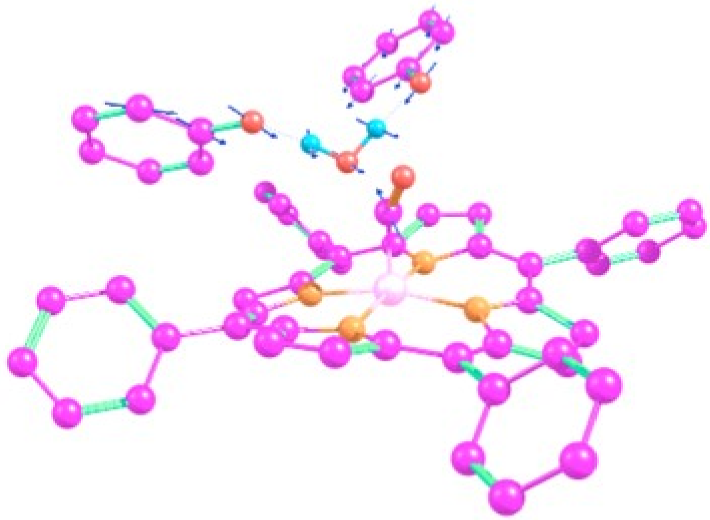

Figure S20: Normal mode of vibration associated with the imaginary frequency of -138 cm^{-1} in **[1]TS2**. Blue arrows represent atom displacement vectors. For clarity, all hydrogens are omitted, except the hydrogens involved in the formation of the H_2O molecule.

Fe	-3.431643000	8.663192000	8.320104000
N	-4.949746000	9.212397000	9.488142000
N	-4.692029000	8.553366000	6.760344000
N	-2.100388000	7.582327000	7.301470000
N	-2.242888000	8.592154000	9.934121000
C	-4.912147000	9.417215000	10.852122000

C	-6.279175000	9.325801000	9.123160000
C	-5.950858000	9.110767000	6.693229000
C	-4.330272000	8.265779000	5.460205000
C	-2.219329000	7.123248000	6.006009000
C	-0.914031000	8.237185000	9.943120000
C	-2.475155000	9.233651000	11.133911000
C	-6.256544000	9.519698000	11.372675000
H	-6.521086000	9.612564000	12.422709000
C	-7.101964000	9.459370000	10.303685000
H	-8.188112000	9.492005000	10.314958000
C	-6.346641000	9.270233000	5.311441000
H	-7.272093000	9.723994000	4.967450000
C	-5.342161000	8.747422000	4.548200000
H	-5.293369000	8.688575000	3.463838000
C	-0.268116000	8.760488000	11.127652000
C	-1.231915000	9.388222000	11.859186000
H	-1.108864000	9.913971000	12.801798000
C	-6.757623000	9.396863000	7.805871000
C	-3.199071000	7.526723000	5.088052000
C	-3.745415000	9.568578000	11.621384000
C	-8.179241000	9.788061000	7.585468000
C	-9.037242000	8.992493000	6.801989000
H	-8.657128000	8.067802000	6.362949000
C	-10.366555000	9.365812000	6.588850000
C	-8.702504000	10.962255000	8.161053000
H	-8.051321000	11.595632000	8.767096000
C	-3.042407000	7.107127000	3.664925000
C	-1.898735000	7.479209000	2.934455000
H	-1.141796000	8.107682000	3.405152000
C	-1.728612000	7.049282000	1.615396000
H	-0.835235000	7.353091000	1.063907000
C	-2.691793000	6.236896000	1.007217000
H	-2.548377000	5.887250000	-0.018873000

C	-3.837587000	5.867554000	1.721704000
H	-4.594161000	5.230284000	1.255967000
C	-4.012409000	6.302851000	3.038756000
H	-4.899134000	5.999713000	3.600185000
C	-3.887019000	10.077209000	13.015095000
C	-3.338664000	9.383222000	14.110150000
H	-2.797107000	8.451553000	13.935140000
C	-4.595997000	11.266975000	13.271414000
H	-5.020502000	11.826840000	12.435702000
C	-3.494164000	9.860140000	15.414496000
H	-3.058045000	9.302540000	16.247370000
C	-4.211293000	11.037492000	15.653638000
H	-4.342554000	11.409713000	16.673326000
C	-4.761843000	11.738657000	14.575549000
H	-5.318871000	12.662875000	14.750513000
C	-10.870429000	10.537602000	7.163720000
H	-11.908101000	10.834955000	6.989729000
C	-10.034314000	11.331676000	7.956484000
H	-10.419199000	12.247505000	8.413170000
H	3.042625000	5.327794000	11.506066000
H	4.827501000	5.945475000	9.880410000
C	-0.914394000	7.064893000	7.779599000
C	-1.146359000	6.200780000	5.705847000
H	-1.021367000	5.664009000	4.769591000
C	-0.344890000	6.156387000	6.807056000
H	0.559993000	5.571534000	6.947498000
H	0.790097000	8.681239000	11.354219000
C	-0.289832000	7.436970000	8.977917000
C	1.115275000	7.000470000	9.218826000
C	1.464345000	6.272218000	10.370890000
H	0.684146000	5.994207000	11.082845000
C	2.789883000	5.896839000	10.607533000
C	3.791436000	6.240533000	9.693271000

C	3.456297000	6.957394000	8.538994000
H	4.231988000	7.233560000	7.820505000
C	2.131566000	7.335238000	8.304911000
H	1.879272000	7.919030000	7.417998000
H	-11.014538000	8.732330000	5.977587000
C	-2.993520000	10.349868000	7.840359000
O	-3.368672000	11.412865000	7.548444000
O	-1.068173000	10.447116000	7.761733000
C	0.837493000	11.910570000	9.949095000
C	1.425631000	12.653220000	11.024171000
C	1.648040000	10.854135000	9.416161000
C	2.688586000	12.345656000	11.533165000
C	2.905650000	10.557104000	9.934743000
C	3.447512000	11.290356000	11.003022000
H	0.835335000	13.470031000	11.453576000
H	1.233347000	10.254246000	8.604859000
H	3.090799000	12.941274000	12.360424000
H	3.470065000	9.726074000	9.499051000
H	4.430601000	11.042039000	11.413059000
O	-0.347247000	12.183448000	9.499966000
H	-0.787575000	11.194458000	8.411908000
C	-0.056557000	10.392366000	4.594788000
C	0.215485000	10.700985000	3.222084000
C	0.587629000	9.215725000	5.100315000
C	1.047905000	9.903666000	2.436217000
C	1.412089000	8.424903000	4.302347000
C	1.659926000	8.753991000	2.960662000
H	-0.268442000	11.588163000	2.800074000
H	0.381782000	8.926018000	6.132128000
H	1.230498000	10.187832000	1.393519000
H	1.857689000	7.522698000	4.734282000
H	2.303888000	8.129064000	2.337025000
O	-0.833528000	11.134231000	5.318161000

H	-0.931212000	10.763383000	6.793564000

m) [1]**TS3**

One imaginary frequency of -1106 cm^{-1}.

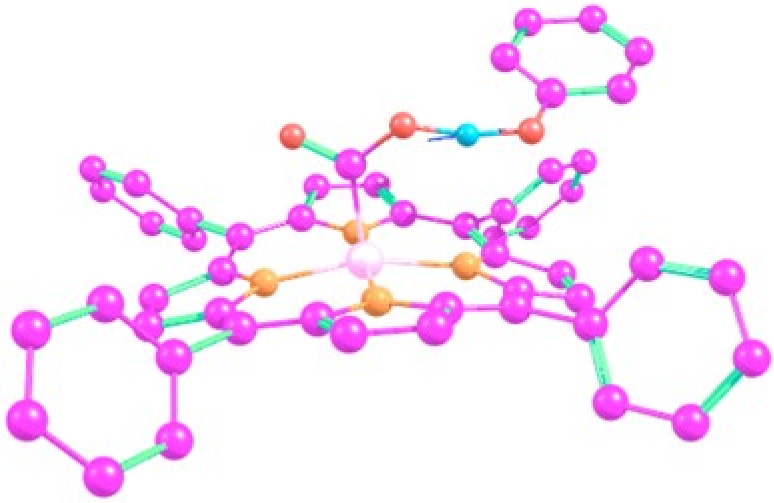

Figure S21: Normal mode of vibration associated with the imaginary frequency of -138 cm^{-1} in [1]**TS3**. Blue arrows represent atom displacement vectors. For clarity, all hydrogens are omitted, except the hydrogens involved in the formation of the H_2O molecule.

Fe	-2.826221000	8.491416000	8.551360000
N	-4.449022000	9.159271000	9.491323000
N	-3.836445000	8.435640000	6.829986000
N	-1.431366000	7.308836000	7.747203000
N	-1.871240000	8.397518000	10.305290000
C	-4.587071000	9.440566000	10.836787000
C	-5.665513000	9.469968000	8.918953000
C	-5.029268000	9.078809000	6.566392000
C	-3.367929000	7.995412000	5.606430000
C	-1.415729000	6.777233000	6.471866000
C	-0.586640000	7.939039000	10.523010000
C	-2.254720000	8.998729000	11.488942000
C	-5.951340000	9.822123000	11.133402000

H	-6.337626000	10.056552000	12.122988000
C	-6.621500000	9.835880000	9.945189000
H	-7.667313000	10.077781000	9.773007000
C	-5.257971000	9.138021000	5.137450000
H	-6.097916000	9.626992000	4.651143000
C	-4.231908000	8.459623000	4.543433000
H	-4.073721000	8.281027000	3.481728000
C	-0.116495000	8.354165000	11.825456000
C	-1.151142000	9.011985000	12.426010000
H	-1.172145000	9.457001000	13.417727000
C	-5.927064000	9.532993000	7.542781000
C	-2.260745000	7.158551000	5.424334000
C	-3.547178000	9.453535000	11.773942000
C	-7.219545000	10.135595000	7.102513000
C	-8.136541000	9.414146000	6.316697000
H	-7.902831000	8.385163000	6.034401000
C	-9.337247000	9.994260000	5.896991000
C	-7.546993000	11.456220000	7.463268000
H	-6.840116000	12.031748000	8.065621000
C	-1.965895000	6.616431000	4.065623000
C	-0.749929000	6.925242000	3.429031000
H	-0.049648000	7.604112000	3.916619000
C	-0.435133000	6.368449000	2.186572000
H	0.512700000	6.627481000	1.706866000
C	-1.325626000	5.487692000	1.561859000
H	-1.070109000	5.038172000	0.598277000
C	-2.544056000	5.183161000	2.180186000
H	-3.246611000	4.496154000	1.700621000
C	-2.863088000	5.748772000	3.419030000
H	-3.808641000	5.499238000	3.906820000
C	-3.816473000	9.988017000	13.143055000
C	-3.749342000	9.156596000	14.274066000
H	-3.533441000	8.093907000	14.139574000

C	-4.102289000	11.351293000	13.331726000
H	-4.158996000	12.007764000	12.460010000
C	-3.946669000	9.673617000	15.558152000
H	-3.888129000	9.009932000	16.424796000
C	-4.212005000	11.035889000	15.733776000
H	-4.358930000	11.445953000	16.736644000
C	-4.291592000	11.872298000	14.615108000
H	-4.501793000	12.937533000	14.743375000
C	-9.649600000	11.308106000	6.263500000
H	-10.587021000	11.763594000	5.933465000
C	-8.751465000	12.034952000	7.053241000
H	-8.989815000	13.059728000	7.351610000
H	2.928600000	4.459716000	12.320405000
H	4.974891000	5.257360000	11.146800000
C	-0.345910000	6.745945000	8.389526000
C	-0.362149000	5.791521000	6.347162000
H	-0.162118000	5.193880000	5.461533000
C	0.301736000	5.773133000	7.537281000
H	1.151756000	5.157747000	7.822049000
H	0.871788000	8.151359000	12.231421000
C	0.123708000	7.106971000	9.653211000
C	1.457341000	6.589767000	10.078284000
C	1.585577000	5.665689000	11.128275000
H	0.687772000	5.317723000	11.645105000
C	2.843598000	5.185892000	11.507329000
C	3.992957000	5.633758000	10.845691000
C	3.875883000	6.559390000	9.800669000
H	4.768973000	6.917882000	9.281321000
C	2.616833000	7.030417000	9.415683000
H	2.503437000	7.753547000	8.603099000
H	-10.035789000	9.414611000	5.287566000
C	-2.178513000	10.271344000	8.174593000
O	-2.963081000	11.228286000	8.147168000

O	-0.898737000	10.509796000	7.995326000
H	-0.069412000	9.751879000	7.589438000
C	1.099892000	9.360897000	5.843718000
C	2.165333000	8.690724000	5.180731000
C	0.276933000	10.207707000	5.047520000
C	2.403002000	8.874297000	3.818263000
C	0.529815000	10.384347000	3.686873000
C	1.594537000	9.727422000	3.053321000
H	2.798780000	8.020997000	5.770149000
H	-0.550927000	10.725599000	5.534513000
H	3.230033000	8.337655000	3.341861000
H	-0.121987000	11.045281000	3.106564000
H	1.793687000	9.877985000	1.989260000
O	0.913994000	9.192424000	7.130764000

n) ^{1}**D**

No imaginary frequency.

Fe	-3.06442949786278	8.53489442645424	8.46065594569298
N	-4.61984809260560	9.17478727211168	9.50071621039930
N	-4.20796201910413	8.41897406852064	6.83199766030469
N	-1.67999239537901	7.40032599335457	7.56068989445008
N	-1.94706847710126	8.58239860930701	10.10703178973918
C	-4.68137431486350	9.37115494888704	10.86775262745493
C	-5.90133224118593	9.37341413763527	9.03326855959072
C	-5.40964329390229	9.07333065916655	6.64144838832011
C	-3.81257354376631	7.97889470054096	5.58604937538307
C	-1.78005910101694	6.79141701823063	6.32048794919220
C	-0.59791255162897	8.29313756429045	10.17551917632905
C	-2.25875821725470	9.23284935338436	11.28202219040503
C	-6.05996479104228	9.53395227834438	11.28011513470283
H	-6.40623799402513	9.62698432010892	12.30621364479912

C	-6.81343848758821	9.54618533374062	10.14289106987087
H	-7.89238867288319	9.65007386179576	10.06005035414451
C	-5.70982329141196	9.15192296474005	5.22713449889041
H	-6.56502911818295	9.65903637783765	4.78880377091625
C	-4.72912596676407	8.46134150043044	4.57545961708404
H	-4.63196180818013	8.29258986849534	3.50581712091570
C	-0.02538141686350	8.89470996583827	11.36160384919537
C	-1.05383649309369	9.47465656521993	12.04692157944948
H	-0.99933074118838	10.01543164720794	12.98806839760811
C	-6.26023697420877	9.48094967604698	7.67929830528000
C	-2.72177972972789	7.13428817220961	5.33941324243136
C	-3.56569385634658	9.52457346678940	11.70611917123549
C	-7.59970025535439	10.05093183683108	7.35880624556146
C	-8.51637790263269	9.37843866039171	6.52945965617939
H	-8.24987813823145	8.40373010669881	6.11603134749542
C	-9.76478495520472	9.93637464447895	6.23986674196180
C	-7.98038834306744	11.29498043040348	7.89961147043932
H	-7.27846269032769	11.83343503900426	8.54021795733529
C	-2.53614855098708	6.59060763202952	3.96331751633231
C	-1.36937147617801	6.88312990005834	3.23445405356314
H	-0.60930902257995	7.52636278430500	3.68350519295705
C	-1.18330139583099	6.37121045303730	1.94758448075329
H	-0.27465036943006	6.61785111396348	1.39215042868117
C	-2.15990012421735	5.55215641865418	1.37006858953910
H	-2.01025946296651	5.14315731510541	0.36725055018208
C	-3.32814995863934	5.25805522447657	2.08241059773404
H	-4.09547542472006	4.61858240640854	1.63789577379962
C	-3.51589404797185	5.77664860819044	3.36730361605914
H	-4.42493094489734	5.54034673122610	3.92539785757128
C	-3.78316734074889	10.01611383489777	13.09670726838860
C	-3.26290994879604	9.31947929765728	14.20413513934917
H	-2.69221432210491	8.40359135564348	14.03718560040375
C	-4.52496821244718	11.18833667735719	13.34134293010795

H	-4.92533699422950	11.75420460374221	12.49794097397015
C	-3.48139930198456	9.77253086620166	15.50801474061088
H	-3.06694259084686	9.21132825167489	16.34959806594531
C	-4.23264019676199	10.93100369199036	15.73474182651474
H	-4.41378281108582	11.28539642024672	16.75321578369459
C	-4.75291016893519	11.63675716421109	14.64454650334556
H	-5.33455854656076	12.54759960493269	14.80974977512188
C	-10.12741176021986	11.17376511015535	6.78278772767735
H	-11.10271325958580	11.61089800490820	6.55224747071318
C	-9.23212223023235	11.84863863331254	7.61987115690617
H	-9.50763011056755	12.81379712980837	8.05390625057640
H	3.35813173117250	5.55765836332475	12.05260446776530
H	5.18414703815956	6.05820617621271	10.43233821010825
C	-0.49393917379223	6.94782961054398	8.11154418087224
C	-0.69955804349804	5.84886388441820	6.14568939243185
H	-0.56353305657149	5.20093406249101	5.28328540071114
C	0.09679153458676	5.94732050895771	7.25210611239825
H	1.00429093270143	5.38942340803457	7.46807812587922
H	1.02506604855812	8.87263743104111	11.64111468377243
C	0.08718200121891	7.45215758087611	9.28546660907824
C	1.49046777272800	7.06067277375691	9.60419416441077
C	1.81050433169145	6.41957166913716	10.81533011456756
H	1.01253363930234	6.19075293695881	11.52494860852261
C	3.12911838392134	6.06220049703180	11.11018672283320
C	4.15363909480905	6.33924904508816	10.19864727287787
C	3.84839522392513	6.97026752594374	8.98738099013596
H	4.64180933286319	7.19497111618326	8.26977186027847
C	2.52936721522736	7.32638500820378	8.69331014942650
H	2.29511708853592	7.83089996143420	7.75292914489785
H	-10.46239542784505	9.39701799778152	5.59386759967576
C	-2.38341139861022	10.20982012200459	7.91025493432923
O	-2.68285983050712	11.32769743480753	8.29192436825606
O	-1.40970021680470	10.16174204557156	6.93180973989135

H	-1.24379527624972	9.21591413750487	6.77299333362415

o) ^{3}**D**

No imaginary frequency.

Fe	-3.06722272424901	8.52767144604051	8.47813065062273
N	-4.63615107655702	9.13619078411162	9.51211827978467
N	-4.23808226864408	8.36783435138262	6.85152220745873
N	-1.69851016052430	7.36402299399897	7.58610790826871
N	-1.96181286002955	8.55512410524133	10.11707548908044
C	-4.70177473936229	9.29847024309797	10.88674300533784
C	-5.93299530385524	9.30440820843209	9.04411715938175
C	-5.43237278956456	9.05831990346810	6.65512972003719
C	-3.82821058000056	7.93998171750228	5.60017972356126
C	-1.81951862210881	6.72510478902724	6.35794953280692
C	-0.59193392598985	8.29181271047573	10.17485528119342
C	-2.27754785788128	9.22523222607219	11.28851994490070
C	-6.06374576238031	9.40507145686490	11.29269986512718
H	-6.41341019744508	9.48506989580092	12.31864770227833
C	-6.82993785575771	9.42002829566548	10.13857201822887
H	-7.91040577319325	9.51180385121531	10.05963536244014
C	-5.69487978918454	9.17144833801514	5.23523636630083
H	-6.52985291202836	9.70675829123908	4.79098969533996
C	-4.72310571728669	8.46923663985776	4.58901576107311
H	-4.61178989463129	8.31932099787302	3.51784228727660
C	-0.02786189697825	8.94899470206581	11.33544484693385
C	-1.05950473296579	9.51884891500066	12.01835582791104
H	-1.00436946727976	10.08904014121862	12.94213315209137
C	-6.28227459385547	9.45918443236861	7.67461042312462
C	-2.75446940894750	7.09480399226178	5.34967448382136
C	-3.57077804690065	9.49299015853944	11.72885781067778
C	-7.59965249717426	10.07834972267617	7.36510243589905

C	-8.52355003542147	9.47025959312970	6.49433976659640
H	-8.27934870511547	8.50599696971136	6.04428016676958
C	-9.75202264174760	10.07546076062345	6.21167904632995
C	-7.95774306114256	11.30636906884869	7.95884089134077
H	-7.25338147052245	11.79393489039637	8.63629278230342
C	-2.53653329993673	6.57697324502322	3.97152913250674
C	-1.33037720637711	6.83517501581865	3.29181362071796
H	-0.56186774065433	7.43446054382524	3.78488961154605
C	-1.11521447778530	6.34876618602606	2.00060179945330
H	-0.17645031934868	6.57271042331176	1.48676996545148
C	-2.10069291743161	5.58543005973225	1.36279779082007
H	-1.92817260651203	5.19482864874753	0.35627963304649
C	-3.30588663800743	5.32341254459294	2.02346053234262
H	-4.08089546026051	4.72643144816489	1.53515349539624
C	-3.52106525293113	5.81526478808917	3.31508621403144
H	-4.45852988547818	5.59780953190176	3.83261394724454
C	-3.79241250586736	9.99766261403867	13.11086253635672
C	-3.21795330373529	9.34975026569319	14.22297713546019
H	-2.60320662570845	8.46176257216880	14.06158493284600
C	-4.59707081642942	11.12997586225996	13.35521506879225
H	-5.04360920160450	11.65963059968536	12.51164190683592
C	-3.43853559823600	9.81138851227365	15.52392497099495
H	-2.97955922699853	9.28626994440338	16.36596415660930
C	-4.25215796711959	10.92724807941645	15.74776211391279
H	-4.43825090907033	11.28621099754817	16.76378217070548
C	-4.82887254327758	11.58520077820877	14.65469873731244
H	-5.45714681075264	12.46538292195572	14.81618649399641
C	-10.08936132844536	11.29646244736291	6.80442980423607
H	-11.04939658237778	11.76969829357020	6.58159738751232
C	-9.18814749341090	11.90676349472887	7.68533768897788
H	-9.44368130162695	12.85952034271308	8.15761600128850
H	3.46886960807287	5.77757888729901	12.10084499691587
H	5.23634934723416	6.17797973370514	10.38909666557989

C	-0.52295349712974	6.88267522942948	8.15845046428847
C	-0.78721211620242	5.75898566293555	6.21677028473647
H	-0.66096819354857	5.09378903548591	5.36615461992749
C	0.02341615196228	5.86154702736528	7.33989798065281
H	0.92139489822683	5.29044327710189	7.56109491248499
H	1.02641354338189	8.96490677548506	11.60105423083245
C	0.08708232724066	7.43863921265882	9.31782414804838
C	1.50132450742129	7.07706406627771	9.61147709705530
C	1.87073319796682	6.52581833933148	10.85332853090410
H	1.09918855721658	6.33428234890324	11.60208493140141
C	3.20363495098507	6.20911166049201	11.13190523603726
C	4.19487920149855	6.42869212025948	10.17024743282797
C	3.84042204853027	6.96171054604041	8.92487116739985
H	4.60719676165275	7.13957654231179	8.16619631349335
C	2.50901725324467	7.28059872131946	8.64868283865216
H	2.23918158304575	7.71029028648367	7.68122125365894
H	-10.45419219792161	9.58435072740857	5.53282664734660
C	-2.37381208128540	10.14763261887109	7.73043438425893
O	-2.68846511543996	11.23852448445125	8.13790363426997
O	-1.48555202179069	10.04446379926143	6.72514866745595
H	-1.29818832625307	9.09391214363808	6.59985611907797

p) ^{5}**D**

No imaginary frequency.

Fe	-3.06844651788818	8.55715684389281	8.46530915966463
N	-4.66581274877489	9.14414082014925	9.53313365173805
N	-4.23586087664590	8.34466183575105	6.87698120244619
N	-1.67496592392203	7.36462695396343	7.56354671997797
N	-1.99909572118364	8.50383926998834	10.12957560771054
C	-4.74494704188946	9.25814045833221	10.91240439142869
C	-5.95364687364894	9.29607608100024	9.05617235878150

C	-5.41770710621779	9.05877116961692	6.67244352193187
C	-3.81471853128909	7.91868187876566	5.61803965429823
C	-1.81862900799502	6.68610943264199	6.36095430850026
C	-0.62201346306471	8.27787425759510	10.17127384322492
C	-2.32110517482323	9.18108962499445	11.30545201079663
C	-6.10774700169643	9.34717416443952	11.30712905810373
H	-6.46888706706996	9.40209155105299	12.33099088522538
C	-6.86363283645380	9.38072476489893	10.14333005227324
H	-7.94388588219776	9.46806151519278	10.05480309516181
C	-5.65084504525677	9.19532748616953	5.25165593855478
H	-6.46266758197458	9.75926429085978	4.80020512139725
C	-4.68393850831479	8.48017384182172	4.60951656540520
H	-4.55765063426564	8.34798077124375	3.53760376605842
C	-0.06656820522720	8.95343927622727	11.32315289229189
C	-1.10524444956583	9.49956376424531	12.01792439357763
H	-1.05152427489810	10.07466334240965	12.93871465577571
C	-6.28387533773353	9.46288480594538	7.67934837965563
C	-2.76054077566288	7.05326614448908	5.36105951604087
C	-3.61163059201740	9.43822034960678	11.75493368891763
C	-7.58820435566057	10.09614488966386	7.35138543474384
C	-8.49041251523939	9.51965928119051	6.43623079316908
H	-8.24186421777924	8.56419355140806	5.97022659545063
C	-9.70284003600275	10.14407531755797	6.12903856351101
C	-7.95653287855610	11.31106228245735	7.96666336402473
H	-7.26977461390106	11.77599837922584	8.67731928864719
C	-2.56954409433562	6.51861946011368	3.98556708180909
C	-1.35701990479000	6.72492604128687	3.29954606679080
H	-0.56407453382772	7.29835928834249	3.78426911691132
C	-1.16747669060989	6.22126707325991	2.01096773450202
H	-0.22294303401503	6.40307634377268	1.49131538898375
C	-2.18585313386870	5.49418137489747	1.38288705224452
H	-2.03419519823185	5.09124371332025	0.37787999969949
C	-3.39710304162423	5.28323796824718	2.05068506896971

H	-4.19661548759393	4.71336272332542	1.56963951409971
C	-3.58690008478486	5.79112404457327	3.33986853900331
H	-4.52880822461788	5.61193530227906	3.86383185718627
C	-3.82815496484817	9.93818857544308	13.13860372130127
C	-3.23650827575505	9.29572466267952	14.24449427223023
H	-2.61414119600889	8.41423921878890	14.07728913779364
C	-4.64355494847957	11.06145866808721	13.38771965786521
H	-5.10126575518718	11.58645351961108	12.54739464571346
C	-3.45108562695335	9.75569276261720	15.54684559498397
H	-2.97960645491520	9.23612009923113	16.38525655496903
C	-4.27415284096094	10.86344079216805	15.77646837625764
H	-4.45428804612523	11.22148751176847	16.79382528477293
C	-4.86754620355012	11.51541287869575	14.68885161676669
H	-5.50241480264112	12.38973268139753	14.85561128835583
C	-10.04839422985506	11.35312941013567	6.74160422720488
H	-10.99550792196781	11.84210679934224	6.49860520295734
C	-9.17181564941635	11.93010908656614	7.66873894507216
H	-9.43384296534727	12.87317816358581	8.15661613883358
H	3.53940106518441	5.88366847133709	12.05955293872968
H	5.27048326495260	6.28732397940864	10.31157699977118
C	-0.51273723476837	6.88342398597419	8.14536085927235
C	-0.79655816251592	5.70905308621547	6.23554657351727
H	-0.68092213655380	5.02001548017088	5.40220310658388
C	0.02362999036172	5.83531269796245	7.35088564170206
H	0.91636496473873	5.26045325363435	7.58445286037627
H	0.99011082128486	9.00242668144853	11.57480654383881
C	0.08560169427446	7.45055563971433	9.30675318448434
C	1.50852014970234	7.12329516798998	9.59014451172221
C	1.90869276558473	6.59334825495846	10.83217966349963
H	1.15349351278691	6.39657340720711	11.59613136435133
C	3.25080332058941	6.30008118938764	11.09068372738015
C	4.22191057589054	6.52119154389657	10.10881878017115
C	3.83769673933195	7.03359129727258	8.86361553527768

H	4.58804478331253	7.21239040818787	8.08885221446578
C	2.49742643826023	7.32986158152021	8.60755807585664
H	2.20470715900777	7.74424061982794	7.64003371797118
H	-10.38669578100990	9.67706556970252	5.41531200217642
C	-2.21055096744628	10.39694938631367	7.74468568764308
O	-2.52560640060024	11.53819034470705	8.00464878427707
O	-1.20107723609181	10.20891725100048	6.84305631316259
H	-1.07021521914894	9.24303214182775	6.77888935000566

q) ^{1}TS5

One imaginary frequency of -168 cm^{-1}.

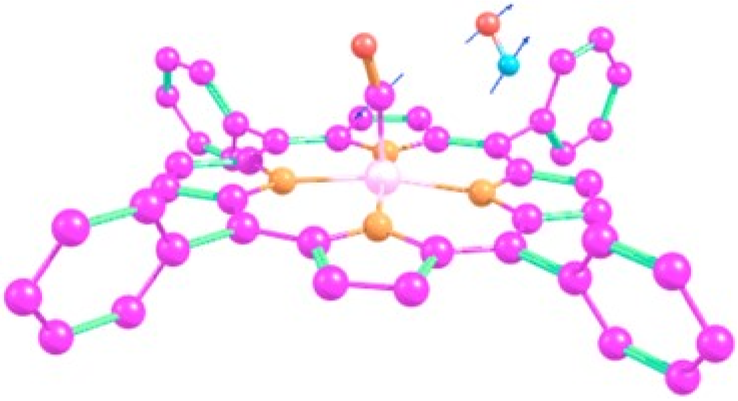

Figure S22: Normal mode of vibration associated with the imaginary frequency of -168 cm^{-1} in **^{1}TS5**. Blue arrows represent atom displacement vectors. For clarity, all hydrogens are omitted, except the hydrogen of the forming hydroxide ion.

Fe	-1.869156000	6.850350000	8.819857000
N	-1.974005000	6.860383000	10.831771000
N	-3.744099000	7.591639000	8.733141000
N	-1.967252000	6.465872000	6.844583000
N	-0.197350000	5.732472000	8.943967000
C	-1.030476000	6.385301000	11.723205000
C	-2.961979000	7.435170000	11.607005000
C	-4.488123000	8.084946000	9.787526000

C	-4.492595000	7.844762000	7.599275000
C	-2.949899000	6.857294000	5.957341000
C	0.575541000	5.307805000	7.884642000
C	0.525695000	5.423351000	10.078258000
C	-1.450743000	6.643374000	13.081649000
H	-0.904947000	6.344438000	13.972455000
C	-2.644927000	7.295498000	13.009702000
H	-3.271806000	7.636846000	13.829219000
C	-5.723338000	8.658674000	9.305818000
H	-6.487967000	9.112716000	9.930403000
C	-5.731757000	8.498510000	7.952560000
H	-6.507110000	8.790411000	7.249435000
C	1.801900000	4.713098000	8.359393000
C	1.769802000	4.779219000	9.721174000
H	2.519945000	4.432426000	10.426711000
C	-4.134920000	8.032170000	11.138162000
C	-4.141197000	7.508745000	6.289198000
C	0.153605000	5.720723000	11.392951000
C	-5.083259000	8.603861000	12.144075000
C	-6.292385000	7.950479000	12.433896000
H	-6.535997000	7.021285000	11.912842000
C	-7.175959000	8.474795000	13.381673000
C	-4.776689000	9.793907000	12.823154000
H	-3.837945000	10.308635000	12.603661000
C	-5.087130000	7.865925000	5.187563000
C	-5.286976000	9.207442000	4.822146000
H	-4.738486000	9.992178000	5.348862000
C	-6.174191000	9.541099000	3.794628000
H	-6.316525000	10.589667000	3.520214000
C	-6.874802000	8.536824000	3.117813000
H	-7.569046000	8.797053000	2.314598000
C	-6.682329000	7.197584000	3.474070000
H	-7.228096000	6.407185000	2.952039000

C	-5.794210000	6.864925000	4.501143000
H	-5.646636000	5.818865000	4.780671000
C	1.069866000	5.310690000	12.500802000
C	1.257858000	3.953291000	12.810198000
H	0.722280000	3.194467000	12.234520000
C	1.760056000	6.277243000	13.251249000
H	1.622244000	7.335337000	13.015666000
C	2.115851000	3.570788000	13.845437000
H	2.248527000	2.510442000	14.075882000
C	2.799607000	4.541154000	14.585986000
H	3.470981000	4.242632000	15.395301000
C	2.619248000	5.895739000	14.285767000
H	3.151743000	6.660079000	14.857876000
C	-6.862352000	9.661958000	14.052480000
H	-7.552756000	10.072489000	14.793886000
C	-5.660541000	10.320226000	13.770346000
H	-5.409337000	11.249098000	14.289182000
H	2.602175000	2.684343000	3.532243000
H	4.163261000	4.533885000	2.930433000
C	-0.956727000	5.942986000	6.069885000
C	-2.552907000	6.552377000	4.600631000
H	-3.148242000	6.753450000	3.714020000
C	-1.312465000	5.990968000	4.671627000
H	-0.687325000	5.634221000	3.856823000
H	2.579518000	4.297078000	7.723572000
C	0.260077000	5.446544000	6.533873000
C	1.325809000	5.143390000	5.529577000
C	1.471740000	3.885412000	4.931271000
H	0.785821000	3.076875000	5.197664000
C	2.491559000	3.667756000	3.997126000
C	3.367073000	4.705715000	3.659988000
C	3.221551000	5.963568000	4.259357000
H	3.907263000	6.774507000	3.996502000

C	2.204926000	6.187567000	5.192217000
H	2.046169000	7.151697000	5.701217000
H	-8.111332000	7.952136000	13.598612000
C	-1.175750000	8.472831000	8.807549000
O	-1.000719000	9.578838000	9.057212000
O	1.071064000	8.384624000	7.201655000
H	1.173255000	7.586323000	7.730492000

r) **¹E**

One imaginary frequency of -7 cm^{-1}.

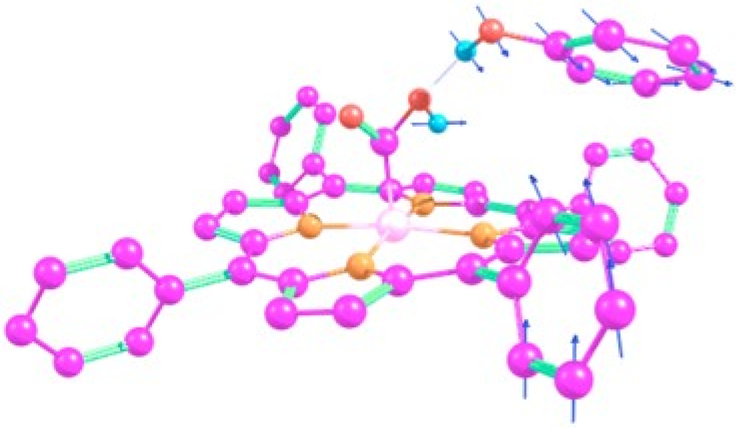

Figure S23: Normal mode of vibration associated with the imaginary frequency of -7 cm^{-1} in **¹E**. Blue arrows represent atom displacement vectors. For clarity, all hydrogens are omitted, except the hydrogens involved in the formation of the H_2O molecule.

Fe	-3.01860696228867	8.41004880654802	8.40684577252160
N	-4.40065044840683	9.50488696858514	9.35411889578651
N	-4.36194886955551	7.89906077198793	7.00874511715454
N	-1.73653071535960	7.03611140966917	7.64960354891859
N	-1.74922931019404	8.71021545745836	9.92556583914809
C	-4.20160225616572	10.32253736405972	10.44584183743680
C	-5.70445047159523	9.72135505768523	8.95871784654249

C	-5.65078140659003	8.37617225594899	6.89047162936177
C	-4.12220046234228	7.17736460182539	5.85361583607968
C	-1.89011907939615	6.32859358712086	6.47215039614296
C	-0.52360338298786	8.10733769537314	10.14420353213381
C	-1.87454687743338	9.66446886004840	10.91789056107505
C	-5.41460441292421	11.04888991347469	10.76055619109182
H	-5.53267170699418	11.75747299857872	11.57561816885933
C	-6.35391047772366	10.65348438623729	9.85667345067563
H	-7.38899146365341	10.97783471419833	9.78890653742728
C	-6.22810872152025	7.96024233473241	5.63071673360316
H	-7.22653048145956	8.21423306104833	5.28353459572666
C	-5.27609243121045	7.23441005988074	4.97998163130594
H	-5.34754893725544	6.77492398254412	3.99791146135223
C	0.13012215100324	8.70143783532509	11.29086396119992
C	-0.70019711782137	9.67001878592671	11.76450055936712
H	-0.54121734049404	10.32620774310983	12.61606374346805
C	-6.30915388221836	9.20188656828130	7.80899509266728
C	-2.95662548736998	6.45520475878245	5.57235028555803
C	-3.00101850046385	10.45852563005669	11.15249560282809
C	-7.72281170595256	9.59403554782635	7.51676345134560
C	-8.75997259581616	8.65094774999613	7.60422633489233
H	-8.52128562132163	7.62144691371621	7.88288717672894
C	-10.08453281406868	9.02151124513210	7.35104367839056
C	-8.04196932966386	10.91434486515741	7.15658591362361
H	-7.24026672800306	11.65180338989565	7.07208730342245
C	-2.82758246144679	5.76522139244752	4.25391143177916
C	-1.82008285394835	6.17094247270599	3.35982500280406
H	-1.15286211795032	6.98724143271723	3.64719222137256
C	-1.67067839097788	5.54597320927548	2.11896117963842
H	-0.88630193734924	5.87824034455943	1.43370294345616
C	-2.52874358366939	4.50307037899012	1.75153269966962
H	-2.41227163938304	4.01284810550493	0.78143313463928
C	-3.53260621784615	4.08772675114694	2.63364410261467

H	-4.20163042217356	3.26938183327689	2.35423724652556
C	-3.68008019761936	4.71372517274362	3.87589030067189
H	-4.45627525787466	4.37952920581069	4.56843673242149
C	-2.92042959750380	11.48968742519992	12.23164547638511
C	-3.65291169493804	11.36473928653414	13.42394675868128
H	-4.28847642411185	10.48857710133345	13.57390731716425
C	-2.09632284999348	12.61562011838507	12.06027986275876
H	-1.52441266487193	12.72233437247578	11.13541884588124
C	-3.56947562488162	12.34510651080213	14.41778451225959
H	-4.14330535090994	12.22762291061228	15.34097573122278
C	-2.74822745598512	13.46431714870205	14.23621193533631
H	-2.68283723469717	14.23020443237298	15.01356455521200
C	-2.00931004648870	13.59503954465151	13.05416251704247
H	-1.36623282542213	14.46557169029889	12.89884509837976
C	-10.39270568465753	10.34213326663923	7.00762017937911
H	-11.42787797443267	10.63531652684827	6.81294193662303
C	-9.36595890573768	11.28724950185528	6.90738826690486
H	-9.59790615606005	12.31828358131431	6.62705453731943
H	2.91578455309907	4.80385150833230	12.27650693229193
H	4.89600919234512	5.19137655113254	10.81480759445278
C	-0.55316265319449	6.57995143092006	8.20785456920783
C	-0.80803181575696	5.38497469664762	6.30927507408448
H	-0.70456278632760	4.68544073255806	5.48354490851912
C	0.01081428531643	5.52893485985839	7.39183824432348
H	0.91454123974802	4.97089005544701	7.62126794247991
H	1.10806274955994	8.42162440610274	11.66889955848133
C	0.04394715277605	7.09102702981691	9.36712201729677
C	1.38054238871764	6.55589788148515	9.76626370266816
C	1.54768157102531	5.84497790384757	10.96702410363261
H	0.68153168328781	5.66990621133682	11.60941350141277
C	2.80387023581268	5.35771742527984	11.34070322711548
C	3.91511741334498	5.57308271317030	10.51870179952216
C	3.76022620707944	6.27533735746773	9.31814729751476

H	4.62218027126283	6.45215978135145	8.66941948480966
C	2.50325415038529	6.76075231246811	8.94563299773966
H	2.38491397663022	7.31515790875039	8.01186156601004
H	-10.88002535209091	8.27600147881140	7.43001583509068
C	-2.41411784480127	9.88777081550287	7.41555858699641
O	-2.94745701896964	10.94776607971614	7.16098124189019
O	-1.11403514221865	9.75776724454861	6.88460554997165
C	0.97006522925480	11.73140655598781	8.81236151299589
C	1.24884176542643	12.70291980762476	9.79144003678454
C	1.67082151712116	10.51276120542751	8.83894352365883
C	2.20946204149220	12.45647468735414	10.77543956259842
C	2.62533707986556	10.27609862477313	9.83140739044827
C	2.90704440520459	11.24175714551436	10.80312301622494
H	0.69635839479938	13.64548909070218	9.76622545068612
H	1.45258154587289	9.74545702260986	8.09299730029294
H	2.41214387636918	13.22266876108017	11.52917257518805
H	3.14305772559414	9.31434101546905	9.85087272050597
H	3.65411781885409	11.04530210537976	11.57582749234370
O	0.03553878971909	12.01982413454303	7.87560721267735
H	-0.81814636249135	8.85528014125906	7.09747288076007
H	-0.24753393993540	11.20004849730469	7.40598601234103

s) **[1]TS4**

One imaginary frequency of -930 cm^{-1}.

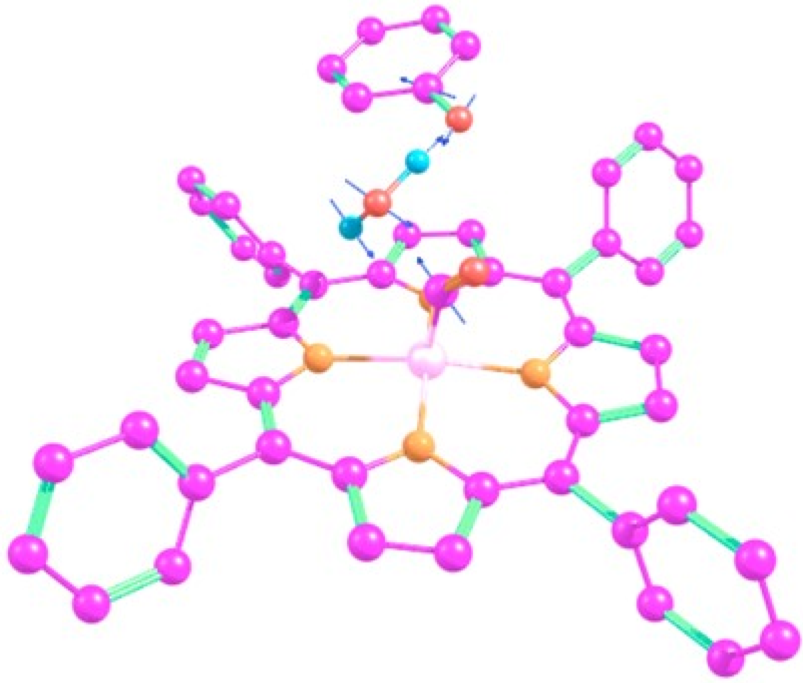

Figure S24: Normal mode of vibration associated with the imaginary frequency of -930 cm^{-1} in ^{1}**TS4**. Blue arrows represent atom displacement vectors. For clarity, all hydrogens are omitted, except the hydrogens involved in the formation of the H_2O molecule.

Fe	-3.100323000	8.442489000	8.330562000
N	-4.601216000	9.203658000	9.428352000
N	-4.351595000	8.213105000	6.775385000
N	-1.698189000	7.403645000	7.331813000
N	-1.983460000	8.305293000	9.998696000
C	-4.499409000	9.744938000	10.694275000
C	-5.884821000	9.478222000	9.002892000
C	-5.675366000	8.595617000	6.709905000
C	-4.011906000	7.816548000	5.496577000
C	-1.700745000	7.115258000	5.982370000
C	-0.756386000	7.686860000	10.146505000
C	-2.231551000	8.931044000	11.201079000
C	-5.747239000	10.372545000	11.068803000
H	-5.940470000	10.878483000	12.010242000
C	-6.614255000	10.178861000	10.035891000
H	-7.650998000	10.497691000	9.968333000

C	-6.183870000	8.415593000	5.368083000
H	-7.196237000	8.645620000	5.045474000
C	-5.148242000	7.952985000	4.612683000
H	-5.150714000	7.716912000	3.551820000
C	-0.243560000	7.904710000	11.481031000
C	-1.153274000	8.678387000	12.131331000
H	-1.100955000	9.049434000	13.150747000
C	-6.415519000	9.175289000	7.745449000
C	-2.758859000	7.341288000	5.094571000
C	-3.372486000	9.675181000	11.521576000
C	-7.844619000	9.526268000	7.478810000
C	-8.817248000	8.517081000	7.396782000
H	-8.515862000	7.473621000	7.519844000
C	-10.158480000	8.839362000	7.170401000
C	-8.239964000	10.863722000	7.313056000
H	-7.486483000	11.653555000	7.365479000
C	-2.531384000	7.008418000	3.655558000
C	-1.620448000	7.764565000	2.897939000
H	-1.111952000	8.611054000	3.365970000
C	-1.351435000	7.432461000	1.567263000
H	-0.636945000	8.028144000	0.993115000
C	-1.993653000	6.340296000	0.972762000
H	-1.768364000	6.067220000	-0.061704000
C	-2.913834000	5.589830000	1.713453000
H	-3.421161000	4.736930000	1.254813000
C	-3.182539000	5.923603000	3.044361000
H	-3.890546000	5.328016000	3.625469000
C	-3.358590000	10.418457000	12.816528000
C	-4.241161000	10.114711000	13.866318000
H	-4.977469000	9.317899000	13.737910000
C	-2.402981000	11.435688000	12.998716000
H	-1.726120000	11.668543000	12.172216000
C	-4.166631000	10.810905000	15.076875000

H	-4.852545000	10.557002000	15.889677000
C	-3.206582000	11.814523000	15.253874000
H	-3.139830000	12.349370000	16.204980000
C	-2.326301000	12.126921000	14.210986000
H	-1.575016000	12.910226000	14.344320000
C	-10.544889000	10.175327000	7.019140000
H	-11.593870000	10.429020000	6.844449000
C	-9.581023000	11.187081000	7.086252000
H	-9.876204000	12.232289000	6.960006000
H	2.841899000	4.146613000	11.432908000
H	4.872206000	5.217383000	10.463465000
C	-0.495274000	6.928656000	7.815397000
C	-0.474395000	6.444879000	5.611620000
H	-0.228081000	6.089662000	4.614588000
C	0.263020000	6.313033000	6.749955000
H	1.231453000	5.834247000	6.864599000
H	0.704024000	7.532985000	11.858530000
C	-0.041043000	7.035277000	9.136300000
C	1.313334000	6.500975000	9.473487000
C	1.466020000	5.434318000	10.374263000
H	0.577455000	4.968842000	10.807190000
C	2.738797000	4.975059000	10.727289000
C	3.878459000	5.573977000	10.178856000
C	3.737077000	6.628441000	9.269041000
H	4.619505000	7.104665000	8.833554000
C	2.463937000	7.088011000	8.920540000
H	2.354190000	7.922578000	8.224325000
H	-10.905288000	8.043271000	7.114200000
C	-2.652127000	10.099829000	7.756502000
O	-2.972914000	11.091470000	7.246837000
O	-0.740163000	10.353110000	8.139833000
C	0.760434000	11.395207000	10.760810000
C	1.130776000	12.098958000	11.939186000

C	1.674388000	10.412649000	10.288570000
C	2.325917000	11.819538000	12.605512000
C	2.860412000	10.136747000	10.966865000
C	3.201977000	10.830667000	12.136180000
H	0.454157000	12.870379000	12.318740000
H	1.428075000	9.860527000	9.381324000
H	2.577120000	12.382952000	13.510249000
H	3.522885000	9.358124000	10.576459000
H	4.130410000	10.606051000	12.667135000
O	-0.384704000	11.650289000	10.161247000
H	-0.520376000	9.451314000	8.406455000
H	-0.571176000	10.985735000	9.130360000

t) **[1]F**

No imaginary frequency.

Fe	-1.98478275902634	8.14531378342664	8.81133948694087
N	-3.48347599870905	9.04895716581951	9.81078816238159
N	-3.24029471124623	7.81723465359203	7.27857752935062
N	-0.64284870518440	6.92148899247836	7.95798552107993
N	-0.89137641602479	8.14651373091202	10.49964243183098
C	-3.46270007632128	9.51522273589688	11.10968166762606
C	-4.68765105530295	9.47503567680114	9.28397147031694
C	-4.48728488009028	8.38320548255670	7.09477701343016
C	-2.98061683826687	7.10948312245554	6.12005518515838
C	-0.72884132319908	6.31880959937399	6.71776011860075
C	0.40112636942713	7.67793493495451	10.64401837796590
C	-1.21720465844246	8.71807560523538	11.71500500751491
C	-4.66891909775462	10.25787491008375	11.39547700333223
H	-4.89947310726762	10.73817795879000	12.34201425205581
C	-5.42080796941942	10.24385798213115	10.25997275865289
H	-6.39015544576599	10.70517156849612	10.09225602509789

C	-5.02693312473601	8.00662453908992	5.81118014145096
H	-5.99970017038106	8.30488745929888	5.42987206646299
C	-4.09250644550000	7.22411301514586	5.20431577341521
H	-4.14784605599066	6.76572046841403	4.22150565455984
C	0.89586037204926	7.97182659893883	11.96811104401144
C	-0.11535199140377	8.58918009938544	12.63933056569646
H	-0.11729673769721	8.93462609032126	13.66925005674173
C	-5.17115908778501	9.18731135928769	8.00761172088376
C	-1.81066038086993	6.39880473141177	5.83630055602305
C	-2.42614134470064	9.34305736488725	12.03101985800282
C	-6.51176156909208	9.71921253336024	7.61271092496296
C	-7.68248013553775	9.17212771423376	8.16220596585775
H	-7.60167335945250	8.36068243923113	8.88959905679377
C	-8.93788666936623	9.65309517935951	7.77842117584745
C	-6.62003463124910	10.75893767141339	6.67594968756198
H	-5.71180564503274	11.18786182282295	6.24566918369704
C	-1.70984433536328	5.71617608068460	4.51112283216997
C	-0.78001476718204	6.15553258731644	3.55287086455258
H	-0.11203637014347	6.98516905347080	3.79610906092394
C	-0.71603941806777	5.54974414697733	2.29506057138093
H	0.00596829481655	5.90787886411185	1.55662513611947
C	-1.58410579108408	4.49943832465386	1.97600605093729
H	-1.54100485607835	4.03127924956698	0.98928205188528
C	-2.50703175324241	4.04834674969123	2.92614935699083
H	-3.18336982513939	3.22429846304597	2.68441742512500
C	-2.56730388988181	4.65132931542681	4.18570647752580
H	-3.28720115388976	4.29917733348112	4.92840577089293
C	-2.64602060050274	9.83899210977132	13.42305829213980
C	-3.62960506351874	9.23456052669095	14.22470028602770
H	-4.20319967830919	8.39585811251502	13.82286693982032
C	-1.90925954459852	10.91382604276062	13.94710927143792
H	-1.14640954005073	11.39309497762493	13.32908943389360
C	-3.88128506955553	9.70263609223345	15.51676443613444

H	-4.65078067240804	9.22366292786884	16.12759901430146
C	-3.14999571261303	10.78131124753326	16.02634159974280
H	-3.35734123634807	11.15727148461256	17.03180183646586
C	-2.15910989427562	11.38129166967460	15.24124416208953
H	-1.58242266665366	12.22257807054240	15.63461244329244
C	-9.03710831220278	10.68771378271224	6.84058082225560
H	-10.01670937551043	11.06526208573980	6.53453226284113
C	-7.87510008988640	11.24133925553908	6.29144404913958
H	-7.94671822937145	12.05224207825563	5.56194829325246
H	4.23532574752557	4.37618671967515	12.03653832097252
H	6.17223821695833	5.46892455133158	10.91552297050298
C	0.60770581378667	6.59708279206701	8.44569047723480
C	0.47311068088722	5.56591611288275	6.44592864665306
H	0.64824381840944	4.96870578481828	5.55545016130221
C	1.30435208903372	5.74721168660267	7.51013834664036
H	2.29318724018476	5.32515744727419	7.66722563951298
H	1.88427781419294	7.71706737516597	12.33983877703367
C	1.13067137862851	6.97148662995478	9.68564725914194
C	2.52528969310843	6.55013320457566	10.02089088743169
C	2.76065256744856	5.54598279111973	10.97428734810378
H	1.91107997984633	5.06663793096810	11.46607712669195
C	4.06564353389770	5.15973480963023	11.29351988634714
C	5.15314882888518	5.77286127596647	10.66090663568528
C	4.92757130461271	6.76889267285937	9.70349788693592
H	5.77008323768784	7.25235310877973	9.20226193294499
C	3.62158651175873	7.15464139978315	9.38488264419305
H	3.44304010070432	7.93387236719269	8.63969621396861
H	-9.83946641622851	9.21331273390256	8.21323605786059
C	-1.26839661012434	9.55800382104857	8.11714089245265
O	-0.77401733080362	10.47038116229374	7.63275570374584

u) ^{3}**F**

One imaginary frequency of -14 cm^{-1}.

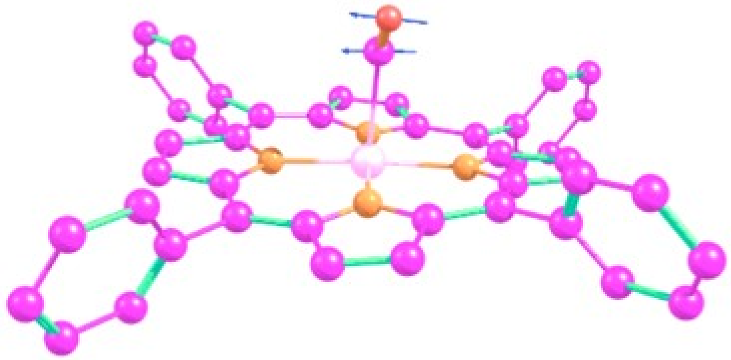

Figure S25: Normal mode of vibration associated with the imaginary frequency of -14 cm^{-1} in ^{3}**F**. Blue arrows represent atom displacement vectors. For clarity, all hydrogens are omitted.

Fe	-2.12347609872180	8.00968679397708	8.87064681543870
N	-3.59181812124668	8.97379029438367	9.84394046235982
N	-3.32373436456126	7.79898400076820	7.26960313081894
N	-0.71917341606248	6.89359565655513	7.96042870157468
N	-0.96944460954224	8.11769065410746	10.50853209104862
C	-3.50836396155304	9.57386264840861	11.08432808708500
C	-4.86058842661822	9.25291642639906	9.37266710030223
C	-4.62894149680158	8.23471904373626	7.14113887360870
C	-2.98293318572054	7.25900591336304	6.04558701029962
C	-0.74323959122248	6.43312625602573	6.66200054088010
C	0.26626724702346	7.52887804600836	10.69827432547296
C	-1.22825322369210	8.83133697519262	11.65998331988024
C	-4.74300621168799	10.26045318946745	11.38523769387531
H	-4.93777282275813	10.83412725212581	12.28700453568479
C	-5.58461001304187	10.04881842593554	10.33362696127824
H	-6.59798505354246	10.41891346470961	10.20437329545024
C	-5.12084313630681	7.94623151105127	5.81521365523330
H	-6.12119389417915	8.16591008520537	5.45306747637492
C	-4.09257402523306	7.37237473373643	5.12848452644502
H	-4.09065835633960	7.03809190653546	4.09475728902558
C	0.79741125873995	7.88861433121267	11.98948038564291

C	-0.12736443392143	8.69310613041588	12.58606480503089
H	-0.07582325526647	9.14091715639538	13.57419647086985
C	-5.37505703848204	8.89739354067576	8.12086639168411
C	-1.76476459140750	6.64702130015496	5.72779229195198
C	-2.40281972770832	9.54113637429569	11.94366456599093
C	-6.78253378390376	9.28563938292345	7.79798729170882
C	-7.86152720588275	8.65313951445188	8.43731481138592
H	-7.66139690449877	7.87440805165665	9.17748946975060
C	-9.17820519620301	9.01073584379868	8.12989603551218
C	-7.04928265040270	10.28797716826491	6.85041524478842
H	-6.21494908870214	10.79035201922974	6.35568430733904
C	-1.52928543685657	6.16650052054583	4.33360065310699
C	-0.51251578583518	6.75638844126256	3.56287517752459
H	0.06652844596581	7.57997568251088	3.98756346536766
C	-0.23569409949103	6.29275992168522	2.27423336537386
H	0.55916265157595	6.76073110930747	1.68783191242469
C	-0.97358773841109	5.23204664524240	1.73712206396657
H	-0.74576043848838	4.85801196968630	0.73536355060461
C	-1.99699440717262	4.64627493282882	2.49081013009307
H	-2.57821921750048	3.81802508864572	2.07687753642636
C	-2.27277563329813	5.11014624345974	3.78074680195145
H	-3.05883539831824	4.63877290397064	4.37522821971483
C	-2.48454481163139	10.24701141967958	13.25772293608187
C	-3.39703367288172	9.83150260893406	14.24361859335389
H	-4.07454225937966	9.00133745910551	14.03095910900804
C	-1.61241872479196	11.31033941096893	13.54934024576646
H	-0.90509790649319	11.64392383994198	12.78621704610388
C	-3.42934526978427	10.45817362039760	15.49237891538492
H	-4.13778146895581	10.11669448967484	16.25170570606996
C	-2.54570217315694	11.50551964041616	15.77690566504045
H	-2.56194811484566	11.98818759488484	16.75742915189334
C	-1.64030448210706	11.93392339871271	14.79982558242479
H	-0.95216018202047	12.75584327448127	15.01380654103334

C	-9.43383594257831	10.00497270332250	7.17817374973514
H	-10.46146372730509	10.28631221701334	6.93197152697079
C	-8.36488621211049	10.64465606801941	6.54048389986654
H	-8.55730233009762	11.42656967220324	5.80127361120607
H	3.61194461439406	3.77144700233486	12.25886607525878
H	5.71843681711070	4.69823107240383	11.30337383711138
C	0.46083098286890	6.42578051327357	8.50691811638717
C	0.43067802892090	5.63298624770766	6.39805095890901
H	0.64868710407343	5.13054166120113	5.45976244932216
C	1.16833440927743	5.61694097108523	7.54520834402730
H	2.10894694331507	5.10506589046727	7.72954114530376
H	1.75412832773062	7.56112336761530	12.38707724874715
C	0.94107208560746	6.71156348063278	9.78689951889385
C	2.26276208936690	6.14384775044646	10.19451350274933
C	2.33360687430807	5.10206659577546	11.13292048588225
H	1.40986419016070	4.69951733653590	11.55579001916281
C	3.57065308390797	4.58362716060593	11.52838419034033
C	4.75263647867631	5.10308508529845	10.98865010662741
C	4.69109898558537	6.13892583083288	10.04891420382692
H	5.60856250223581	6.55220472732297	9.62126026975115
C	3.45334408380913	6.65517914171254	9.65369459700942
H	3.40213401105759	7.46738910200518	8.92448756974366
H	-10.00596260160860	8.50606811042300	8.63482267377131
C	-0.92170212697131	9.85724085217506	7.66410702430402
O	-0.15546216840962	10.16459713207398	6.89590254258262

v) **^{5}F**

No imaginary frequency.

Fe	-1.96602827172305	8.26040214397503	8.77324985979341
N	-3.54685985391773	9.06438584060231	9.84454138102130
N	-3.30341374515900	7.82694279915033	7.20425226324610

N	-0.61589251302727	6.93536074644817	7.91773761583902
N	-0.85708701062620	8.17232389893393	10.55960208159209
C	-3.47941397504341	9.57507097636342	11.12150427234255
C	-4.76295196842351	9.43822115141045	9.31448993238942
C	-4.57678949904120	8.32280026017972	7.08143713623933
C	-3.01554514753982	7.14201089053324	6.05125892476754
C	-0.70879558301455	6.37486091168257	6.66247729442642
C	0.39106044911337	7.62253059046736	10.70922020674119
C	-1.19795309955953	8.75260905146811	11.75514164684444
C	-4.68561011051472	10.32345942094697	11.39540379309373
H	-4.90316753419146	10.85562051562549	12.31740111651907
C	-5.47463357816025	10.24126911676997	10.28006785213285
H	-6.45197112958040	10.69164884989717	10.12753292540049
C	-5.12550235070226	7.92667139787887	5.79797751753891
H	-6.12239760839559	8.16512506044714	5.43599690920250
C	-4.15824781035994	7.21026996622786	5.15582063619026
H	-4.21742175515011	6.76573849484801	4.16625500819559
C	0.87275638351163	7.88387354619041	12.05360345945887
C	-0.11478123395552	8.56419932105189	12.70440685150662
H	-0.11090727214055	8.90439311394984	13.73655821138471
C	-5.25763217896013	9.09648499078595	8.03927855905333
C	-1.80026808644528	6.48524747095548	5.77301450736630
C	-2.40976428045189	9.41635355983390	12.03024933849518
C	-6.62825385659408	9.57668676464730	7.67998661056433
C	-7.76271164095990	9.03619655101050	8.30777276550245
H	-7.63251828865427	8.26481770865700	9.07080744308662
C	-9.04448489821695	9.47129406210202	7.95717477515830
C	-6.80308814538133	10.56656292511397	6.69894066924319
H	-5.92460627168173	10.99298394097238	6.20889462831679
C	-1.65034308365548	5.85232930695979	4.42767025984258
C	-0.68247588821335	6.33097026216670	3.52706062393346
H	-0.04171182556688	7.16412827546827	3.82535142122016
C	-0.54096443805948	5.75644751646273	2.26112241278953

H	0.21268163665780	6.14327547402864	1.57037049293531
C	-1.36845754206499	4.69605725862706	1.87508089050503
H	-1.25826373822837	4.24678256160346	0.88470001693391
C	-2.33485685086320	4.21108746958544	2.76325854693163
H	-2.98202963235456	3.38064010201632	2.46882795229830
C	-2.47216528031538	4.78291976145549	4.03165996869910
H	-3.21907891805579	4.39603364627257	4.72893119385681
C	-2.60230050085134	9.96980475390824	13.40468044681375
C	-3.61504174728957	9.45527498032299	14.23322037442150
H	-4.24409588136260	8.64194434240821	13.86395839511647
C	-1.79149590434631	11.00898777521436	13.89253487171393
H	-1.00562993811014	11.41886117830449	13.25367243088538
C	-3.81943393462769	9.97329672340867	15.51460644854469
H	-4.60934461157099	9.56078496553756	16.14751903139005
C	-3.01246634429998	11.01432295776971	15.98705270119685
H	-3.17914900974824	11.42619579360169	16.98592214354020
C	-1.99435366486754	11.52707695031048	15.17547071797467
H	-1.35923890328171	12.33897539858898	15.53962822119068
C	-9.20858923515932	10.45619572924188	6.97586990412209
H	-10.20861194341887	10.79986668950227	6.69704936260204
C	-8.08390761912825	11.00531786134383	6.34957472201556
H	-8.20569327920753	11.77885233764990	5.58685070256139
H	4.06882353818967	4.17238041438179	12.10792121752288
H	6.05935449506871	5.19870827627228	11.01854066927224
C	0.60005038214942	6.56342731106806	8.44929206452533
C	0.48531696514987	5.60367379569821	6.40216597831114
H	0.67949723478399	5.02699524795049	5.50175976878521
C	1.29200175078464	5.72199147724012	7.50188927743252
H	2.26255590645475	5.25972988788962	7.66220584477687
H	1.83397228717933	7.57139386226642	12.45306283289706
C	1.09231732255928	6.89648550615691	9.72843241629308
C	2.46469449925363	6.41436246487701	10.07867535000840
C	2.65164675080888	5.39884171384932	11.03087490296947

H	1.77909731035951	4.94827452400215	11.50957581391590
C	3.93768443823261	4.96453938933444	11.36614805997749
C	5.05541484361521	5.53991128919904	10.75105387304602
C	4.87901105521627	6.54651126481054	9.79430056904542
H	5.74485870873144	7.00146457155020	9.30602085060486
C	3.59219445787447	6.97997305846308	9.46042137092321
H	3.45207732131580	7.76906511156490	8.71747233574495
H	-9.91607608982264	9.03496871051484	8.45225386105077
C	-0.98334908003489	9.89055756479614	7.80899765893208
O	-0.34964915493325	10.57888244722688	7.17819283727599

w) [1]**TS6**

Three imaginary frequencies of -309, -14 and -5 cm^{-1}.

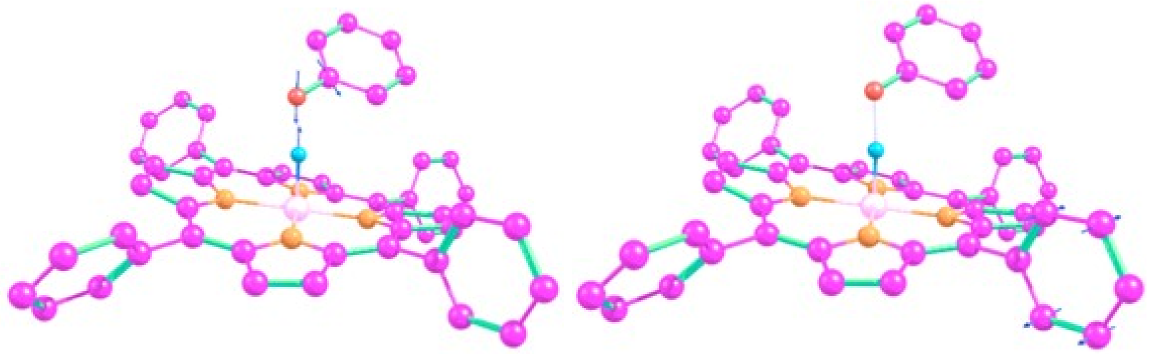

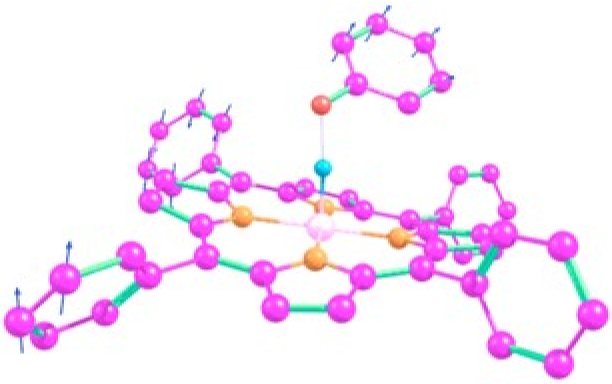

Figure S26: Normal mode of vibration associated with the imaginary frequency of -309 cm-1 (top, left), -14 cm-1 (top, right) and -5 cm-1 (bottom) in [1]**TS6**. Blue arrows represent atom displacement vectors. For clarity, all hydrogens are omitted, except the transferred proton.

Fe	-1.697466000	7.779170000	8.568521000
N	-2.865192000	9.247850000	9.217542000
N	-2.493307000	7.951814000	6.737148000
N	-0.697983000	6.164766000	7.981330000
N	-0.926355000	7.580883000	10.412268000
C	-2.859542000	9.834460000	10.480285000
C	-3.874803000	9.900520000	8.507159000
C	-3.450800000	8.860181000	6.316799000
C	-2.063953000	7.314056000	5.583442000
C	-0.629228000	5.647704000	6.690643000
C	0.004541000	6.642786000	10.832063000
C	-1.032490000	8.488682000	11.454560000
C	-3.926469000	10.811284000	10.575090000
H	-4.165234000	11.389586000	11.464238000
C	-4.555595000	10.842733000	9.367564000
H	-5.403083000	11.455988000	9.069091000
C	-3.593809000	8.819552000	4.892212000
H	-4.267363000	9.442816000	4.309264000
C	-2.711387000	7.864967000	4.431247000
H	-2.536091000	7.551789000	3.404350000
C	0.535432000	7.002937000	12.111048000
C	-0.103197000	8.163441000	12.495592000
H	0.048819000	8.732291000	13.409360000
C	-4.162836000	9.738706000	7.161525000
C	-1.186265000	6.211255000	5.551572000
C	-1.977765000	9.541281000	11.515054000
C	-5.208981000	10.589810000	6.516395000
C	-6.414920000	10.026321000	6.064498000
H	-6.611991000	8.968307000	6.256668000
C	-7.352692000	10.797660000	5.370879000
C	-4.973835000	11.953325000	6.269022000
H	-4.037031000	12.401128000	6.609027000
C	-0.869703000	5.591386000	4.228808000

C	0.376762000	5.820894000	3.621600000
H	1.097506000	6.472557000	4.121772000
C	0.699450000	5.228774000	2.396336000
H	1.672174000	5.426246000	1.937325000
C	-0.220023000	4.388938000	1.759077000
H	0.034789000	3.917377000	0.806004000
C	-1.467870000	4.156697000	2.349605000
H	-2.193192000	3.501952000	1.858962000
C	-1.789566000	4.756832000	3.571385000
H	-2.762325000	4.568451000	4.033395000
C	-2.066630000	10.324592000	12.781550000
C	-2.383712000	9.689025000	13.997084000
H	-2.588167000	8.615881000	13.994679000
C	-1.825400000	11.710259000	12.804066000
H	-1.569463000	12.219821000	11.871590000
C	-2.442540000	10.405762000	15.194354000
H	-2.691497000	9.886218000	16.123897000
C	-2.194720000	11.783774000	15.201723000
H	-2.236774000	12.347883000	16.137122000
C	-1.893801000	12.432897000	13.999727000
H	-1.692954000	13.507800000	13.994051000
C	-7.097603000	12.149708000	5.113657000
H	-7.824607000	12.750655000	4.560862000
C	-5.906280000	12.726121000	5.569294000
H	-5.691506000	13.778642000	5.363406000
H	1.039942000	2.299750000	13.451224000
H	3.290963000	1.649452000	12.578927000
C	0.046092000	5.287952000	8.773447000
C	0.101324000	4.399368000	6.693334000
H	0.274992000	3.779054000	5.816860000
C	0.488480000	4.160816000	7.977233000
H	1.043928000	3.305909000	8.353701000
H	1.303704000	6.453322000	12.649921000

C	0.393535000	5.493416000	10.102772000
C	1.222922000	4.467182000	10.799745000
C	0.722895000	3.810042000	11.940262000
H	-0.262497000	4.089543000	12.320736000
C	1.457798000	2.806560000	12.577035000
C	2.716579000	2.438718000	12.086904000
C	3.236245000	3.095063000	10.966033000
H	4.219035000	2.816559000	10.574720000
C	2.500469000	4.103871000	10.335661000
H	2.916945000	4.619203000	9.467111000
H	-8.282343000	10.339035000	5.021849000
H	-0.517260000	8.735830000	8.289864000
C	0.113067000	10.880155000	7.632581000
C	0.233853000	12.154749000	8.269257000
C	-0.604426000	10.866043000	6.396501000
C	-0.310651000	13.312160000	7.709987000
C	-1.157203000	12.025178000	5.855565000
C	-1.020407000	13.265474000	6.500108000
H	0.774247000	12.194638000	9.220606000
H	-0.732580000	9.907281000	5.890372000
H	-0.181041000	14.267794000	8.230175000
H	-1.712225000	11.960087000	4.913338000
H	-1.452601000	14.171159000	6.066116000
O	0.613576000	9.802201000	8.164334000

x) ^{1}**M**

No imaginary frequency.

Fe	-2.39866047041247	7.86485910863807	8.66026353570341
N	-3.74381221795533	9.13147153017439	9.42272253986664
N	-3.37504024774438	7.89237903078384	6.91629674586569
N	-1.18663045040802	6.43803518102667	7.95422828214372

N	-1.55994125140243	7.67449352955412	10.46855186147007
C	-3.87159725400564	9.53551523136820	10.73878798489102
C	-4.68072775319498	9.86532530458731	8.71992003633106
C	-4.36359091974293	8.78053428159444	6.53266024580926
C	-3.12016662021137	7.12124507809578	5.79675580565828
C	-1.22529044417277	5.83703704359836	6.70991721197933
C	-0.40574168511992	6.97870043947871	10.78016285452967
C	-1.95025510094777	8.28498367085992	11.64669588989753
C	-4.92186611393715	10.52494067486523	10.86377471175090
H	-5.22888696891979	10.99845362483475	11.79297421912690
C	-5.41180174394854	10.74195761844922	9.60932888874598
H	-6.20635456357798	11.41922905063101	9.30484856319747
C	-4.72694923728986	8.56695148131068	5.14805935986567
H	-5.46846343974623	9.13823464456103	4.59649296881929
C	-3.97182364315612	7.52488073726204	4.69905929449184
H	-3.97484083056969	7.07660515297998	3.70858963216065
C	-0.05765996865638	7.17106997906026	12.17141876611556
C	-1.03082471343854	7.95541931850034	12.71513348120204
H	-1.11570963810730	8.29408472323352	13.74462964862208
C	-4.94843946194288	9.75057928561513	7.35317268950208
C	-2.12564834000324	6.14467749953894	5.68627217988792
C	-3.05081429259229	9.13426539849358	11.79892237552688
C	-5.91346496858617	10.70602133221090	6.72780048534763
C	-7.18129175905989	10.28827052046191	6.29222521715866
H	-7.49165312249716	9.25484207284543	6.46539462988880
C	-8.03911295886582	11.17610699746917	5.63629574742920
C	-5.53440714298803	12.04239503515473	6.51516356459440
H	-4.55194159902874	12.37901614636704	6.85610506771476
C	-1.97146881968083	5.43975814201311	4.37592144755201
C	-0.89868270902763	5.75589293856492	3.52496455116163
H	-0.17980580142608	6.51836320157326	3.83493327258068
C	-0.74823864089171	5.10902441560991	2.29490078545405
H	0.08828970840118	5.37164712912482	1.64180330222085

C	-1.67037267437671	4.13388365696325	1.89748187370879
H	-1.55215734159885	3.62684501470127	0.93631880186969
C	-2.74304014824597	3.81152388140531	2.73604833906723
H	-3.46730985106500	3.05132182792290	2.43111157515703
C	-2.89329797442438	4.46287426946635	3.96533912923448
H	-3.73110430515692	4.21075152871168	4.62048518860498
C	-3.35300627622928	9.67577226145088	13.16004907889150
C	-4.48957053189941	9.23490594665253	13.85814979680271
H	-5.13344739187156	8.47876709988383	13.40244096384512
C	-2.52874388655472	10.64435312609137	13.75709431789534
H	-1.64230828329435	10.99381778242712	13.22217604260457
C	-4.80087285025508	9.75575621879229	15.11784955132201
H	-5.68639438024749	9.39710976772769	15.64939147270195
C	-3.98003026757218	10.72972812445407	15.69726373687071
H	-4.23022938104187	11.14603364835735	16.67687854116283
C	-2.84016643683571	11.17013656167046	15.01516206163819
H	-2.19147034841288	11.92827756884240	15.46228745624879
C	-7.64272047130564	12.49903637995776	5.41072512920173
H	-8.30882856389389	13.19099345087665	4.88916989362263
C	-6.38992826035282	12.93187591816709	5.85892356438795
H	-6.06857776485089	13.96239660655436	5.68334995703829
H	2.79128881401262	3.11867669891548	12.47272358498503
H	4.96193509941463	3.98022589355559	11.59867856499722
C	-0.10026314259460	5.87700426527972	8.59837447932650
C	-0.16917768308930	4.85365400694219	6.58841163062880
H	0.00348035549981	4.22366423704111	5.71926656274222
C	0.53807399733652	4.89022753122994	7.75315498673702
H	1.40140800735626	4.29164397230821	8.03344743253362
H	0.80818835009002	6.74055223924178	12.66791168148039
C	0.31599695618004	6.16491829798030	9.90182071056747
C	1.59161317881138	5.54768214767718	10.37931620726292
C	1.59879197658646	4.49799397261515	11.31192220585970
H	0.64885674648725	4.11942421363615	11.69736069595628

C	2.80382586897914	3.93702707659602	11.74788457194821
C	4.02140111109776	4.41897290302907	11.25420895167950
C	4.02600420166249	5.46140526070129	10.31973627910880
H	4.97054672111328	5.84664853258295	9.92521382097395
C	2.81970850930133	6.01921241748321	9.88561021651351
H	2.82213054734290	6.83449040440639	9.15793963448882
H	-9.01821913580551	10.83049601792704	5.29340318794536
H	-1.45167277144145	8.97882653128152	8.25457908712204

y) ^{3}**M**

No imaginary frequency.

Fe	-2.37906595663253	7.86456181804942	8.62637879078843
N	-3.78257660207515	9.05047304639842	9.39925959454097
N	-3.37759736873811	7.83756465259298	6.87998672867007
N	-1.19908573301061	6.40121802047964	7.94468829530191
N	-1.60463409733052	7.62486165304709	10.47529493285308
C	-4.02240810764987	9.33311055126243	10.72829228349731
C	-4.66753424915122	9.81503154979880	8.67395098140348
C	-4.21491984080061	8.84456652021315	6.44570168865931
C	-3.12784969438790	7.03580971437176	5.79084847875106
C	-1.32539583798476	5.67180515142229	6.77907807212831
C	-0.37192431357155	7.07464227476031	10.74452446036797
C	-2.05377918587191	8.17305813191308	11.65529472940734
C	-5.12396277073440	10.26573082380709	10.84333926370958
H	-5.53170329999744	10.64221874492820	11.77808857159411
C	-5.50680386844233	10.58285966076168	9.57252393152820
H	-6.30021009002672	11.25954161865186	9.26530726569606
C	-4.44960108503833	8.69967907568762	5.02851446547401
H	-5.02890723398726	9.37709702219587	4.40780141125314
C	-3.81048935595808	7.55678788741640	4.63150225052804
H	-3.76853276185740	7.12846317430033	3.63323487930057

C	-0.02412462030956	7.30393021136326	12.12710872028471
C	-1.08671230864773	7.94430948128095	12.70344340049580
H	-1.19280898702312	8.25911073310636	13.73846619347638
C	-4.79629496923818	9.82391867883444	7.27368343665629
C	-2.24134951317342	5.94262890622373	5.75393361449824
C	-3.23428576577626	8.91641182257572	11.80837459608956
C	-5.60193350336712	10.92276278913270	6.66795877194378
C	-6.70543298281201	10.69014137598841	5.82686890871104
H	-7.01995547340761	9.66609805037625	5.61589871735039
C	-7.41594950397575	11.75658650596029	5.26762392351758
C	-5.24695689214186	12.25761060019116	6.94662010302110
H	-4.39286367826322	12.45495860473221	7.59850890689353
C	-2.21924103925717	5.10404249618404	4.51808490893453
C	-1.07373691560982	5.03096073697437	3.70496080545968
H	-0.19126979025723	5.61536633812711	3.97503782482049
C	-1.06105142631630	4.23495048337047	2.55562468397794
H	-0.16093718106159	4.18703941705090	1.93696491142727
C	-2.19827429938153	3.50439788879217	2.19347611769340
H	-2.18966412248039	2.88486046196503	1.29303080324582
C	-3.34884861695028	3.57865518171266	2.98609125286442
H	-4.24199794641718	3.01357981399394	2.70594995116750
C	-3.35883487661152	4.37091033474018	4.13840308514476
H	-4.25608646116457	4.42011368990414	4.75998061897939
C	-3.62322233157663	9.37374993299048	13.17819391014652
C	-4.73018250421395	8.80248123709759	13.82750147546229
H	-5.28932475943675	8.00767368828766	13.32771125977542
C	-2.90947486469840	10.39334189077118	13.83063198176642
H	-2.04944917959481	10.84669977082287	13.33200150473506
C	-5.12089342106449	9.24636668769566	15.09468011464028
H	-5.98259056548628	8.78874421978882	15.58788571918433
C	-4.40903743096466	10.26906506761634	15.73144132499627
H	-4.72280118482082	10.62626815408945	16.71607649171751
C	-3.30024673157667	10.84005733177243	15.09639411260238

H	-2.73731761106824	11.63856874370103	15.58718377479728
C	-7.04650303092432	13.07683127448888	5.54750308951491
H	-7.60607256712295	13.90724726435199	5.10951831774425
C	-5.96162837458949	13.32382561463018	6.39646395567451
H	-5.66222754923327	14.34925357916827	6.63101701886135
H	3.47101208245274	3.83755533792839	12.37203193150353
H	5.41690605176626	4.86746835599950	11.20930668837658
C	-0.07449757798706	5.92626117480964	8.57842982413854
C	-0.28471412245341	4.66295802056730	6.71566336661723
H	-0.17177239971060	3.92151815111372	5.92924020563844
C	0.51413276697942	4.85252575812487	7.80543682515632
H	1.40120353009319	4.29188097853527	8.09066300190757
H	0.90236862994563	6.99513340089542	12.60437463579616
C	0.39729385688166	6.32891626636503	9.83636381482533
C	1.77223627247020	5.89931259683017	10.23130426437507
C	2.00254294179141	4.95068685524752	11.24096922461916
H	1.15078644745826	4.49702008377676	11.75241785843295
C	3.30624519125744	4.58058845505040	11.58741469607711
C	4.39973232741718	5.15751451045254	10.93138755066790
C	4.18152191769964	6.10128383829043	9.92070592487785
H	5.02730143396104	6.56011216606815	9.40156498207877
C	2.87777788109935	6.46473585999950	9.57097988994435
H	2.70432134417037	7.20280562504518	8.78369252921247
H	-8.27074377951254	11.55203249097309	4.61751669634418
H	-1.17006522851753	9.29326373401262	8.14043551468222

z) **^{5}M**

No imaginary frequency.

Fe	-2.35725879163164	7.88550640479806	8.61651247269013
N	-3.79843284926460	9.01654767300425	9.39857171134044
N	-3.40097175582004	7.79981629486618	6.91866674220755

N	-1.22842079087695	6.36902719686054	7.97079957032403
N	-1.60607506103081	7.63398207547571	10.44985461907787
C	-4.07695341125894	9.26127601968640	10.73567338823083
C	-4.67041154192802	9.80254382672828	8.66803451823174
C	-4.18132996300477	8.85497376886512	6.45164454027918
C	-3.16141520711991	6.96978029555267	5.83011882702375
C	-1.39832614266605	5.58041219556843	6.83913249917190
C	-0.33454770580341	7.13932557470922	10.70743423167037
C	-2.07429616314467	8.16512228306072	11.64749583448411
C	-5.16826619352307	10.16513682108217	10.84112938699101
H	-5.59543402560701	10.52676580646475	11.77327080312630
C	-5.52664455300000	10.52052365525543	9.54787098280574
H	-6.31601159955137	11.20135681200530	9.23861899357261
C	-4.37430174822490	8.70595658675591	5.02527946545279
H	-4.89792390466763	9.40957413154921	4.38438580199196
C	-3.79889251189946	7.52596197399912	4.65813133804425
H	-3.75763051253375	7.08779804375019	3.66386939218826
C	0.01297347630473	7.40285847219038	12.08789631501222
C	-1.07580244581953	7.97278663876516	12.67683584794039
H	-1.18767668803470	8.27325786436376	13.71579133102392
C	-4.74497321056894	9.84822727537840	7.24472099041461
C	-2.32856410891969	5.85726515404042	5.80311093134680
C	-3.26894301045985	8.84300305342004	11.82485275504083
C	-5.49254849602229	10.98375364758168	6.63993522470502
C	-6.57169961948022	10.80663548324415	5.75363814397445
H	-6.90482934778167	9.79744634663006	5.50297516521670
C	-7.23745807674643	11.90596957982551	5.20095940909445
C	-5.12155832226317	12.30309933753264	6.97341853612662
H	-4.29204398391209	12.46159730655407	7.66650785900370
C	-2.31688355259187	5.01101567244018	4.57492378058642
C	-1.15945443208175	4.88445190785432	3.78366289539725
H	-0.25623323459106	5.42561386136380	4.07395675414713
C	-1.16191953933538	4.09694805490021	2.62928485716784

H	-0.25304925256559	4.01077791148740	2.02754209165736
C	-2.32606540331676	3.42530240751586	2.23728734709234
H	-2.32912340526327	2.81264822681193	1.33206715233595
C	-3.48655135650449	3.54963030799178	3.00793233755970
H	-4.40023151181745	3.03055412557150	2.70554559921666
C	-3.48064121187110	4.33275343036399	4.16696616317541
H	-4.38616442076470	4.42119120527354	4.77197703944202
C	-3.67873735480434	9.27921043049644	13.19338409428640
C	-4.78460064201922	8.68476351410514	13.82530988366214
H	-5.32292263732777	7.88355622752112	13.31294843582140
C	-2.99719217884652	10.31073790477955	13.86227154258822
H	-2.14191684515405	10.78692548244496	13.37682891955631
C	-5.20040419573372	9.11179399620725	15.09003592843970
H	-6.05923665022679	8.63384026816167	15.56896909000597
C	-4.51673628685695	10.14350804666175	15.74395369657292
H	-4.85008973360254	10.48777963894989	16.72681210372760
C	-3.41201494127492	10.74049923012217	15.12663142135264
H	-2.87124237244484	11.54672909044255	15.62974070815738
C	-6.84955021690501	13.20786824858015	5.53411644304820
H	-7.37462018141038	14.06443958787803	5.10345690774453
C	-5.79008052332231	13.40148340709738	6.42991803749824
H	-5.47589649462505	14.41207140955648	6.70689465422045
H	3.62911683839552	4.01313640456835	12.30477382912811
H	5.52591612310511	5.09381287130779	11.10617760266106
C	-0.08059775951715	5.91645849794138	8.59208616641238
C	-0.38842990684110	4.57847643091417	6.79993868181612
H	-0.29666698912798	3.80409953935358	6.04280878499913
C	0.45946290894104	4.81481839075386	7.87318655334137
H	1.35076621963961	4.25999822485979	8.15674381412905
H	0.95966570914321	7.14116037481762	12.55398681068048
C	0.43982528929065	6.40564885990194	9.82171087439379
C	1.83443828602564	6.02132670474306	10.18721244219103
C	2.11147119355169	5.07998256871955	11.19258731833419

H	1.28133678464092	4.60360718261198	11.71901385223692
C	3.43086170414953	4.74788791394035	11.51992338732330
C	4.49617614839005	5.35294520986067	10.84433306188264
C	4.23315241752778	6.28716459996308	9.83445348207663
H	5.05734270865153	6.76870249196374	9.30121026840428
C	2.91410690644145	6.61303708754087	9.50620326225783
H	2.70807776647557	7.34383531977471	8.71966199269177
H	-8.07327552843115	11.74026286833832	4.51581154728637
H	-1.27749484493256	9.19643107597966	8.12242956448613

aa) [1]**N**

No imaginary frequency.

Fe	-1.46001808198874	9.10877332030085	7.68892273446824
N	-2.13286906682889	10.95954467109840	8.14135022976683
N	-2.26248995817463	9.29102867262209	5.83356791316675
N	-1.04904286953834	7.16862121878271	7.31507406252911
N	-0.94960601405195	8.82476159968460	9.62962756193166
C	-1.99548558262413	11.61277058031744	9.34654397472972
C	-2.79915014968520	11.83487594772032	7.31028253224115
C	-2.84578424045244	10.41335672862977	5.28819140979149
C	-2.15786849765334	8.37930956926300	4.80596337165019
C	-1.17240701929273	6.51988639800690	6.10585997089600
C	-0.40177035750549	7.68958568028025	10.18633182639975
C	-0.90099870211816	9.79056728006604	10.61113101618397
C	-2.61465925722050	12.92069262758754	9.28085017649463
H	-2.67064650906903	13.63015169632315	10.10259555981152
C	-3.11837768144628	13.05570827834884	8.02186347287736
H	-3.66701657603288	13.89816635312288	7.60908408819275
C	-3.09980384660733	10.20947970058514	3.87841335214425
H	-3.53917262469013	10.94168277196907	3.20604861768673
C	-2.66544859378821	8.95166659243881	3.57788295253813

H	-2.68122356227564	8.45458361914211	2.61143865758233
C	0.02120690246603	7.95286600601800	11.54421989040541
C	-0.28229849394997	9.25701349627357	11.80516036740585
H	-0.10122090624854	9.81228152568371	12.72171181072736
C	-3.12553301865210	11.60757750668856	5.96724703808481
C	-1.64246598625870	7.07982874658403	4.91060048840570
C	-1.38249298565558	11.10218889393873	10.49830891102210
C	-3.79775049043360	12.70724654361691	5.20970512800598
C	-5.12567183882924	12.57076387608201	4.77080617137861
H	-5.66897209629921	11.64882051719545	4.99221928432469
C	-5.75333245778763	13.60121662065866	4.06425124743910
C	-3.11186541608316	13.89937098604326	4.92105722546057
H	-2.07702589189535	14.01311675037791	5.25279972045085
C	-1.60879680743362	6.22682422598558	3.68301356471652
C	-0.37929485263971	5.86105625792061	3.10889022665052
H	0.54829046498628	6.21664788536026	3.56435514493770
C	-0.33576242007422	5.05578348792138	1.96703521919992
H	0.62935383293191	4.78400824288953	1.53120113252158
C	-1.52294056919224	4.60141898354654	1.38251432159164
H	-1.48976106083750	3.97074710990554	0.49025558109628
C	-2.75273954370308	4.95896808521599	1.94601166363283
H	-3.68479635417155	4.60494464949349	1.49758373185652
C	-2.79469717599724	5.76551966429796	3.08730277393921
H	-3.75598344248989	6.03736967638232	3.53003800231017
C	-1.26114169661147	12.00383115122765	11.68498787567265
C	-2.00160924666343	11.76251499446524	12.85461312165341
H	-2.67709631675619	10.90436034515861	12.89177737555453
C	-0.40195198132661	13.11513438357965	11.64884065191629
H	0.17924035855193	13.31074101239652	10.74434069521987
C	-1.88514472805868	12.60972420014602	13.96053860776778
H	-2.47188018050883	12.40919057983382	14.86098563134268
C	-1.02396903444637	13.71153760890134	13.91453624623019
H	-0.93116141899143	14.37323012005447	14.77979804726831

C	-0.28183232261466	13.96141375439954	12.75505843121355
H	0.39581370087032	14.81828719046670	12.71146054927464
C	-5.06069244070814	14.78380305980362	3.78281174247388
H	-5.55045286288930	15.58951454011755	3.22975037121222
C	-3.73731755747197	14.92968557634101	4.21323418170421
H	-3.18721754351845	15.84871296254598	3.99420663167462
H	-0.36781888924954	3.23931159957206	12.93694909471805
H	1.91802380950620	2.39531013860034	12.38833559455123
C	-0.56011781754045	6.22995039725726	8.19941160747702
C	-0.77681335879978	5.13257612840977	6.23931533012339
H	-0.80323291771721	4.39355274555662	5.44257810801313
C	-0.40581805542075	4.95100534598579	7.53799490729995
H	-0.06661297837697	4.03488802409245	8.01441381114727
H	0.49861746863856	7.23370260277317	12.20450188632636
C	-0.23174147782139	6.45708525529150	9.54129715554608
C	0.35836550669301	5.32977637087450	10.32572685524834
C	-0.35385556473632	4.73956848770524	11.38321543535006
H	-1.35421101864211	5.10734383285919	11.62421414791179
C	0.20225860889839	3.68961699724946	12.11996843956719
C	1.48195754241748	3.21524773391974	11.81165024295015
C	2.20037502607110	3.79598242719075	10.76059355082295
H	3.20212716334569	3.43376645578943	10.51487945152024
C	1.64237602424438	4.84480974543041	10.02407824180306
H	2.20651034534740	5.30102331631918	9.20693057212770
H	-6.78864311307663	13.48106290962387	3.73455921653555
H	1.34472152708363	8.35715788568019	8.06578882362232
C	1.46055247990823	9.25146001096776	7.41588608877923
O	0.36209327878496	9.82361373022292	7.08020572843204
O	2.59601591087745	9.60599100482021	7.08651500730111

bb) ^{3}**N**

No imaginary frequency.

121

Fe	-1.37705219519280	9.16757674848067	7.67199062943863
N	-2.05972711644821	11.02016250741394	8.12045602960334
N	-2.14938933400743	9.35970938336756	5.81421021560422
N	-0.96445226528822	7.22352126869930	7.29967994971968
N	-0.87892474261654	8.87409175980068	9.60794687449820
C	-1.94686194925520	11.66187366448813	9.33511543266509
C	-2.74181193152682	11.88875978466692	7.29393214425917
C	-2.74958194640730	10.47526130671126	5.27017361612596
C	-2.05871594467486	8.44051161256133	4.79027077125583
C	-1.10602300845905	6.57107493365517	6.09338006801110
C	-0.33276875653255	7.73834033887587	10.16797554292518
C	-0.83479225172722	9.84144356004697	10.58894119115647
C	-2.59154491196748	12.95581329940681	9.27596320208064
H	-2.66971176468522	13.65407557620706	10.10551319862631
C	-3.08773370434011	13.09537709396187	8.01289258014498
H	-3.64928657095520	13.93007461066255	7.60160693238439
C	-3.00724323255535	10.26641622929488	3.86284288486611
H	-3.45142336965227	10.99392987457905	3.18944605582278
C	-2.57401022008414	9.00760763720465	3.56580266010870
H	-2.59727958408530	8.50424532966765	2.60280925332393
C	0.10295637072718	8.00869485889744	11.51871044136806
C	-0.20276073866694	9.31274580194600	11.77742990967809
H	-0.00931376157622	9.87167975102673	12.68894199274391
C	-3.06457281439493	11.65938296893710	5.95146420902180
C	-1.57512744727314	7.13113675469315	4.89891767196856
C	-1.34043681378307	11.14565306726177	10.48786130869052
C	-3.79568902987606	12.71647563593493	5.19001157249579
C	-5.10384235987435	12.47697165209878	4.73497771265237
H	-5.58773664088676	11.52944175447073	4.98464619424313
C	-5.77980051903077	13.43000974916849	3.96880927064826
C	-3.18756529911361	13.94428355427341	4.87885377049389
H	-2.17100415988793	14.14123448175548	5.22817035828098

C	-1.61000842257027	6.25669107253124	3.68592723101382
C	-0.41818572037801	5.85360289725673	3.06120954874034
H	0.53600977010326	6.20884700419069	3.45925623985872
C	-0.44870059222993	5.01176861770180	1.94449597179054
H	0.48586337663219	4.70308806989443	1.46811023369062
C	-1.67195578044660	4.56082511793259	1.43654198410380
H	-1.69641370318346	3.90131898357673	0.56512265744225
C	-2.86515509393523	4.96078737169528	2.04902041039401
H	-3.82341029356165	4.60835743146190	1.65717620628475
C	-2.83349692539760	5.80200047975210	3.16568522348860
H	-3.76363471678770	6.10706221652186	3.65222073240924
C	-1.27123481124009	12.01053456132471	11.70476059666871
C	-2.00472785414106	11.67483595218675	12.85640932373087
H	-2.64478744690127	10.78943703695847	12.84133007453721
C	-0.46047835026500	13.15787236566525	11.73193608109465
H	0.11078082031043	13.43085810592270	10.84133979206695
C	-1.91316395820352	12.45281105485641	14.01345245801813
H	-2.48684090404952	12.17027361001351	14.90037733939787
C	-1.08718445257456	13.58297023256278	14.03584758341230
H	-1.00204651082228	14.18586123668771	14.94367005543504
C	-0.36604787107714	13.93634551397114	12.88977412889381
H	0.27840491328609	14.81922878554423	12.89857446523993
C	-5.15952740651682	14.64406049228592	3.65226291837257
H	-5.68368332357749	15.38838400508187	3.04738526715495
C	-3.86315980777335	14.89964782235641	4.11329459496432
H	-3.37000863122317	15.84323685849102	3.86451885520687
H	-0.42532338583023	3.34561080003058	12.99127790685098
H	1.74890805004429	2.30216320412512	12.34302627800676
C	-0.51195880892661	6.27304135692486	8.19205492879362
C	-0.75109676335634	5.17632266823493	6.23497481754198
H	-0.79787347261071	4.43532662349734	5.44114246301728
C	-0.39398621702190	4.98822425668559	7.53680112695411
H	-0.08381029925510	4.06406831198797	8.01653143773390

H	0.59002071457198	7.29338794733972	12.17555574340487
C	-0.18798737879319	6.49607792518503	9.53611588018044
C	0.35280558361327	5.35154561331135	10.32800992714356
C	-0.35557869098496	4.83264846144626	11.42552404134675
H	-1.31044265057530	5.28385871732859	11.70690337541402
C	0.14368641766872	3.74356958678113	12.14687730945161
C	1.35984298349048	3.15562705985122	11.78157079717576
C	2.07540093956421	3.66714666216846	10.69318444974181
H	3.02523662518802	3.21365040914281	10.39720640878576
C	1.57797272530465	4.75903800116538	9.97715598108995
H	2.14116383201228	5.16396506264369	9.13314110818207
H	-6.79218709082435	13.22091198547955	3.61260529574796
H	1.50125161934636	8.46724847442986	8.15138313284821
C	1.68232770931910	9.34785641909664	7.49109435467556
O	0.62396968071280	9.92591003218813	7.06829436694500
O	2.85400601796420	9.66079464431469	7.24338683465059

cc) ^{5}**N**

No imaginary frequency.

Fe	-1.05904940552515	9.27888889094935	7.57227304316516
N	-2.05443331612089	11.08345908787547	8.11469856879254
N	-2.17865093702856	9.35216387883749	5.76337231803465
N	-0.94713385389887	7.17140884208566	7.27766358914170
N	-0.92121085853294	8.86248719813173	9.65909340155610
C	-1.99656660701915	11.69065981723116	9.34341391362237
C	-2.71435336344941	11.93666850665329	7.26764160193868
C	-2.74874775712307	10.47867410640553	5.22502599147757
C	-2.12695435459297	8.40720568222275	4.76790285551179
C	-1.14083529865052	6.51653073166423	6.08815535795522
C	-0.33155962496750	7.74204488646319	10.19018653630813
C	-0.90079953733409	9.83255212333797	10.63055344576469

C	-2.65410978653969	12.98311321360566	9.27493056010396
H	-2.78476951892974	13.67166802903443	10.10642585119316
C	-3.09820931743036	13.13567694673398	7.99033123958524
H	-3.65510512849337	13.97281407256343	7.57625714634739
C	-3.03887991558569	10.24974609330260	3.82106959484044
H	-3.47118164432620	10.97325566142990	3.13491058943558
C	-2.65357075177550	8.96913525929442	3.53911365878866
H	-2.71826296265759	8.45503483603862	2.58332998363215
C	0.10644200802990	8.02116498329703	11.54459963078970
C	-0.24403078533308	9.31391749485496	11.81700583089531
H	-0.05522345617124	9.86705032373252	12.73334448872343
C	-3.01820588874166	11.68343029079058	5.91263635714756
C	-1.64963794901129	7.08666502630976	4.90212824706817
C	-1.42379389819816	11.14033853906388	10.51121458330222
C	-3.71555429218535	12.75830588454310	5.14315438690735
C	-5.01817550005074	12.55007392786153	4.65659748572902
H	-5.52387508477586	11.60753082683938	4.87974315047059
C	-5.66419759036404	13.52979496178573	3.89829947499977
C	-3.08402966131806	13.98389802290987	4.86894941552916
H	-2.07178242803217	14.15798595948758	5.24224548877172
C	-1.71237820942247	6.20100733169264	3.69770524476295
C	-0.53201222637069	5.77559028201126	3.06494340162990
H	0.43053269864305	6.12042763675102	3.45200373024928
C	-0.58374572332469	4.92837629017864	1.95330969914505
H	0.34240289628475	4.60427007343744	1.47073036365114
C	-1.81778990595147	4.49310494399966	1.45774472450857
H	-1.85953419719089	3.82945054234264	0.59013946410314
C	-2.99964115734621	4.91461907416873	2.07771106503010
H	-3.96612350390015	4.57431298084388	1.69537484526745
C	-2.94647591982280	5.76183459235251	3.18920009088428
H	-3.86808657530441	6.08369418323737	3.68112511539291
C	-1.38367765534405	11.99323365504369	11.73751097100181
C	-2.07938469438492	11.60682701464233	12.89751250289905

H	-2.68089201948210	10.69506504845773	12.87876887928323
C	-0.62711212021951	13.17771197643741	11.77195695145063
H	-0.08318080663894	13.49105690808284	10.87763925083069
C	-2.00008968693327	12.36736848989188	14.06684582806558
H	-2.54455279707008	12.04441807854053	14.95840187032779
C	-1.22446383794890	13.53225099376630	14.09555639084508
H	-1.14727948666254	14.12156839004036	15.01294309690770
C	-0.54495892482956	13.93895278377565	12.94173436877599
H	0.05992466485110	14.84942114077237	12.95453537684881
C	-5.02047446872918	14.74104254983101	3.62026624322854
H	-5.52196327558664	15.50643287997210	3.02244573751823
C	-3.72952211222131	14.96608304480680	4.11147195153539
H	-3.21745107924398	15.90718033343859	3.89342851416081
H	-0.22914543846958	3.38088499002675	13.01404135460237
H	1.93344899852753	2.35792309520419	12.29830347961951
C	-0.47955247553224	6.24996152495127	8.17895196918912
C	-0.78827596101086	5.11719350282701	6.24188469021666
H	-0.86808836539518	4.35623754662738	5.46911915335165
C	-0.38508695883832	4.95043371565645	7.53724681921557
H	-0.06845424382554	4.02760321679801	8.01682946825542
H	0.62923913784775	7.32715414465230	12.19764290417295
C	-0.15024652083225	6.50751052297681	9.52818022932345
C	0.43285724553458	5.37828597907572	10.31209843137728
C	-0.22965754264477	4.85871490197095	11.43833335704411
H	-1.17965778561749	5.30037310711944	11.74937437461723
C	0.30633670535605	3.78006339891619	12.14847436421895
C	1.51538712111487	3.20273238325841	11.74471222297281
C	2.18702441127430	3.71507355718599	10.62906568236372
H	3.13157446029724	3.27058113193764	10.30388203514955
C	1.65151928600804	4.79522846228843	9.92269816748579
H	2.18046842202800	5.20029605385815	9.05685285508080
H	-6.67326437457999	13.34483050400976	3.51999196163924
H	1.67988239908852	8.47238401699784	8.23136860199617

C	1.87116959292012	9.33081041013133	7.54940075881672
O	0.80730186284720	9.90355726290527	7.08999086053117
O	3.02992504418496	9.64979891677190	7.29754437692593

8) Cartesian coordinates of the other compounds investigated in the studies

a) $^{2}\mathbf{4}^{0}$

C	14.80882517284594	9.90434790250516	-5.60356514427484
C	13.57873445034695	10.52213018911017	-5.82830248432907
C	12.41640514803837	9.73830413916790	-5.97008485720541
C	13.71850668102655	7.70914996693548	-5.70258102395760
C	12.48844006224268	8.33020541495615	-5.91096932808322
H	13.51569207287615	11.61156595351947	-5.88910035820508
H	11.45371094981788	10.22623248040428	-6.13842045350007
H	11.58431087767072	7.72963480588755	-6.03423066581781
C	14.07053239963198	6.32861389809331	-5.62963696950314
C	16.10608360311518	10.45173390814024	-5.40096167198676
N	14.85061649783622	8.50980585779683	-5.53081264798847
N	15.38340225253426	6.15085213676871	-5.40441542428921
N	17.04635982750624	9.50781068910583	-5.18052642129650
C	16.00629901698806	4.83510846654312	-5.41654986520346
C	19.39984424100911	8.75419702542797	-4.87953213011657
C	17.40858326832212	4.93490457851744	-6.03095259496960
C	18.47339215238545	5.60823438958029	-5.15748231594634
C	19.04450460196241	7.65887724569232	-3.87865319944050
N	17.96432865889053	6.77281596779573	-4.38002706040199
H	15.41393520222814	4.11723550623557	-6.00371855528503
H	16.06778430745071	4.42547584854368	-4.38941775426077
H	17.77244316967329	3.92441919786038	-6.27840320505218
H	17.31217076785346	5.48148244160544	-6.98370526582346

H	19.29163164159478	5.96442631701881	-5.79855620350611
H	18.90529110085327	4.88200521380146	-4.44775964213607
H	17.49297895696751	6.38875405794465	-3.56438073696667
H	19.93376338934972	7.05339376176518	-3.63588408337479
H	18.68869590560737	8.11091809503751	-2.94054519877843
H	20.39104665383099	9.15733388589010	-4.61638648482907
H	19.49574503089461	8.31122815347101	-5.88568973696237
Co	16.41164767511214	7.71947458905384	-5.19325342695437
C	16.41687406361178	11.91768254051968	-5.43751333301420
H	16.83655086302681	12.27836313336258	-4.48145601157421
H	17.16703403823897	12.15573299760528	-6.21173050966578
H	15.51755627396503	12.50825766944207	-5.65879670529371
C	13.10388390321580	5.19716281018232	-5.80865244794074
H	13.35790902513513	4.57519200288616	-6.68554543149879
H	13.09757768589054	4.52102772201995	-4.93622901955558
H	12.08190693380991	5.57317773454966	-5.95434360264448
C	18.41607431036462	9.92368020387381	-4.91159394587392
H	18.46409603161316	10.45250965450334	-3.93871743161843
H	18.75913969966409	10.64798783087891	-5.67101366587477

b) **^{2}G**

C	14.77640396460733	9.84754243154049	-5.57090231794701
C	13.57342650026656	10.48064635521618	-5.89407531633891
C	12.42671641186073	9.69749740515818	-6.10195289594898
C	13.70926668148171	7.69634661310489	-5.69285304410848
C	12.48808369553664	8.29516860653483	-6.00412556475650
H	13.52152217354643	11.56682942248103	-5.98580299672373
H	11.48119773353664	10.18286882886966	-6.35218630006854
H	11.59696115238465	7.69008284870227	-6.17673358776297
C	14.06432077332189	6.28772734131449	-5.57064224312461
C	16.09062287234002	10.40702453555267	-5.30863905432227
N	14.79919830999404	8.48838550389554	-5.48547912311496

N	15.32416268340964	6.10873962294936	-5.22830498685491
N	17.00884925736015	9.49937173687322	-5.02345913417241
C	15.97913217286173	4.80963159118730	-5.22291073755382
C	19.37986544724194	8.76385841800121	-4.83539690505344
C	17.29347583713024	4.91375037464434	-6.01387369249872
C	18.45076935506867	5.63619743481228	-5.30438873874053
C	19.11747066951085	7.57403604419628	-3.91811198869681
N	18.00949743586294	6.71004185515832	-4.38545185025297
H	15.34401899739136	4.03642305847626	-5.67621132048050
H	16.17321155430989	4.50941667144185	-4.17841448242991
H	17.63581050836133	3.89968252304770	-6.27318615761856
H	17.06049329900172	5.41989755605646	-6.96467085060152
H	19.11908051757760	6.07688521022477	-6.05773628921316
H	19.04793933623548	4.91241621512099	-4.72233183313261
H	17.60889907579796	6.28076020370321	-3.54829351763263
H	20.03382046600505	6.96873303284594	-3.81516627014639
H	18.84615895896848	7.93187967562996	-2.91340267420815
H	20.37104703746807	9.17560867104546	-4.58636248294008
H	19.43365144649490	8.41969821073968	-5.88240582790718
Co	16.34086383986200	7.69850436327149	-4.89297133283849
C	16.36966836465592	11.87693343661802	-5.36789917713061
H	16.73828106697019	12.25109478464000	-4.39792882991327
H	17.15054645349723	12.10308262842568	-6.11251244086332
H	15.47257805414352	12.44676358850266	-5.64114008445037
C	13.10399295334138	5.17786236349360	-5.87242321483992
H	13.41261409116335	4.62016458456006	-6.77313781954172
H	13.05284850516843	4.45471288021046	-5.04327988839071
H	12.09350439085814	5.56696526225311	-6.05119412164893
C	15.86789219204088	7.81367127762356	-2.95662757514726
O	15.24465947632295	8.81067915601845	-2.60613660563900
O	16.27363695641001	6.83211081748269	-2.30575048499261
C	18.37230845377555	9.90666772161366	-4.71353179683686
H	18.39749603342947	10.30572921903884	-3.68142408155625

H	18.68759740842516	10.72834330172224	-5.37564837685905

c) ^{2}**H**

C	14.78379664354337	9.81368730406841	-5.58388356155805
C	13.58440657349270	10.44832855653723	-5.92449701086740
C	12.44335096010981	9.66467006371355	-6.13351579935595
C	13.72297626845090	7.67825876664903	-5.68048913801168
C	12.50327806837383	8.26656396942336	-6.01432391488721
H	13.53780886445030	11.53226819613037	-6.03432662071773
H	11.50193215342299	10.14562654321278	-6.40522380451905
H	11.61876805292828	7.65451956352397	-6.19336992011953
C	14.07587866232352	6.25733265061612	-5.55276586704290
C	16.10775695439017	10.38209316172847	-5.32432691288961
N	14.80291327997728	8.46757681306778	-5.47265460324045
N	15.32741324564615	6.07570807593957	-5.23206298253369
N	17.02473653969212	9.48275067662875	-5.06724726320214
C	16.00000354560708	4.78193942595948	-5.26607148884227
C	19.38428438219115	8.73395463016824	-4.82151609861178
C	17.29800392848393	4.94054860305610	-6.07275783187043
C	18.44883946679762	5.66002892668366	-5.35247721521980
C	19.10386284438484	7.55530041887581	-3.89628406588997
N	17.99720026539010	6.68662813015630	-4.37400018943574
H	15.36780970658880	4.01809659848487	-5.73598150554284
H	16.20970696659511	4.45452639808358	-4.23418214158481
H	17.65562672635033	3.94314107691992	-6.37024555128413
H	17.04233409588691	5.47219257566253	-7.00327535100711
H	19.09232736802853	6.15314171977891	-6.09291974805248
H	19.07041797456008	4.93174125838251	-4.80583307236328
H	17.66400426568594	6.18777596627827	-3.55196018841683
H	20.01223201397065	6.94260745149636	-3.78091263470517
H	18.82149882372226	7.91696182144488	-2.89692435928095
H	20.37206871417727	9.14188328703322	-4.55652508766256
H	19.45791898399932	8.38275039327469	-5.86405019499960

Co	16.34211066145204	7.67995226332421	-4.86378894069062
C	16.36843506928130	11.85339417142831	-5.34349550218445
H	16.71745867793157	12.19770547317900	-4.35605786455842
H	17.16141694968851	12.09939827607098	-6.06713737151296
H	15.47072112741877	12.42106887229066	-5.61581925899119
C	13.08824045801183	5.17047881363255	-5.83498793793425
H	13.16252552909794	4.84288700290199	-6.88676015266865
H	13.26040164721495	4.29388455052689	-5.19736463084343
H	12.06335232798108	5.52806121628282	-5.66831611216687
C	15.88455221817757	7.96875188761670	-2.97395921667376
O	15.31123420381865	8.91359508846409	-2.48029515372133
O	16.31794782846178	6.95299239379581	-2.17169194599849
C	18.38526932997796	9.88456541884051	-4.72911769449103
H	18.38221136948545	10.29420382525157	-3.70235120885104
H	18.71055858628672	10.69796266893616	-5.39505076865197
H	16.07802878149123	7.15215583947848	-1.24443252834607

d) **^{2}I**

C	16.15768977396836	8.55191236213550	-6.24137843895117
C	15.37910295179596	8.93548310649339	-7.33633960708828
C	14.34558479865224	8.08478259213219	-7.74582923574572
C	14.93729943876409	6.53998314934891	-6.00703751178063
C	14.12293049229435	6.86500956853340	-7.09369167951479
H	15.57165096779477	9.86923423463329	-7.86334587822650
H	13.71886981593564	8.36766077878998	-8.59303806615231
H	13.34004521652072	6.18783306272041	-7.43375963946475
C	14.96302939670193	5.29369374438770	-5.19616704179299
C	17.34699819265315	9.22439386151606	-5.65746848996407
N	15.88171941392374	7.40467830458530	-5.61230361386226
N	15.86810787397325	5.30884374894555	-4.27630150295481
N	17.89254590090817	8.57215454306293	-4.68190530554515
C	16.15998198063807	4.20956533080420	-3.37486363395858

C	19.92705382907829	7.88560452995725	-3.45649559984199
C	17.64205057357684	3.80032975072931	-3.45081555954883
C	18.66469505208091	4.94961208554392	-3.50413388704476
C	19.25378405525194	7.09317201825135	-2.34369150163760
N	18.17708166735219	6.18075691471597	-2.81340921421262
H	15.53077501043674	3.33354164708971	-3.57100884270209
H	15.91768818692620	4.56855320516507	-2.36123159777438
H	17.83859439904299	3.17350181601019	-2.56801555838518
H	17.80223934129559	3.16633452649350	-4.33524180302740
H	18.89962434903394	5.22233179948852	-4.54348399257627
H	19.60550977325117	4.62100273779852	-3.03655290431224
H	17.68087635237446	5.88625655714718	-1.97596257785827
H	20.00451665806414	6.49017135438912	-1.80872937371734
H	18.79566864721105	7.77675478270204	-1.61366691816537
H	20.84775103679252	8.31925936717701	-3.03749193743409
H	20.24325602938433	7.21793319009116	-4.27404178385387
Co	16.88899798085874	6.96784891016257	-4.09807481879419
C	17.83785770828784	10.53226312126543	-6.18059453940025
H	17.76034866018838	11.29686260593888	-5.39052203369400
H	18.89798607228161	10.46397180038935	-6.47039927857401
H	17.26179986266780	10.86900163065479	-7.04903659423605
C	14.05064119775573	4.14827114911023	-5.48682093519299
H	14.61382559558704	3.35017605557761	-5.99918253336849
H	13.63518717688594	3.72658706171865	-4.56076108528671
H	13.22147160334706	4.45075589603998	-6.13642528974033
C	15.75792984867945	7.91091477999495	-2.80013329325317
O	15.10321794724778	8.52706414422695	-2.11940666888320
C	19.10199690794598	9.04206281529866	-4.01681468805247
H	18.79847270161629	9.72037437816508	-3.20051765048834
H	19.71697494697138	9.62667021161773	-4.71489640894113

e) **^{1}7-K^{+}**

Re	-0.58909687858616	8.99299155143199	7.60957658255615
C	-1.99098467952718	11.38816961077302	11.02318413303491
C	-2.60250276981300	11.16382476186038	6.88503273833761
C	-3.35754051262054	12.04005908417505	6.03975487455051
C	-3.35071504746323	11.88015185903895	4.67960722573395
C	-2.58894671624784	10.80556035175336	4.11481497555831
C	-1.88205406044772	9.98275687718285	4.94758357266769
N	-1.84852670402018	10.12272567160425	6.31311791547261
H	-3.93562565325995	12.84315821321170	6.49951732369419
H	-3.92351498579454	12.55138563011576	4.03658150422347
H	-2.57213314366528	10.62533664671193	3.03895320749467
H	-1.28701720162516	9.15811738436495	4.55349660698619
C	-2.48047585849731	11.27807458974346	8.27939135193089
C	-3.13991074688795	12.25468859488693	9.09138420349099
C	-2.90619940649256	12.33009354391697	10.43713317296840
C	-1.39445257137079	10.44818379608180	10.23734907786170
N	-1.58942480552393	10.34367197412088	8.86967501652898
H	-3.84367921113265	12.93851976061294	8.61393418839371
H	-3.41849731907088	13.06701678734049	11.05798112629320
H	-1.77838808374110	11.39957308866487	12.09357167006408
H	-0.71210334550936	9.71367682473129	10.66426246579399
C	0.27307805761913	8.02409904060664	9.05909089756116
C	1.05631284416345	9.60667719966450	6.97543518365068
O	0.77879758086369	7.48616529655645	9.94859234904032
O	2.01718849847860	10.16715224253869	6.58914117167067
C	-0.45024496944696	7.40080703477530	6.49526166071806
O	-0.39123206126800	6.46002779713535	5.82830506664385
K	0.09501294488757	12.33623128939846	7.31448877807845

f) **[1]J**

Mn	-0.11694382018828	9.66839919664885	7.59168135684987
C	-2.19473571812539	11.28095789113674	11.07905556685099

C	-2.22509783415723	11.57898154322267	6.88004695441306
C	-3.08026343128695	12.37986040883773	6.10515073077455
C	-3.05922432718150	12.27008009721702	4.72184950936217
C	-2.17739727043761	11.34996943430886	4.13774167844074
C	-1.35499568263576	10.59627119480604	4.96202782700328
N	-1.36562311257217	10.70244911847339	6.30124574193817
H	-3.76625216164658	13.07273522063631	6.59134656895613
H	-3.72455434153785	12.87794381632751	4.10529533785775
H	-2.13204600335081	11.20956508118964	3.05640997588796
H	-0.65651769248502	9.87011559913341	4.54623752053477
C	-2.22889867494391	11.56332155970759	8.34227239209953
C	-3.06312656626513	12.37350383753952	9.13037164884058
C	-3.04726996851604	12.23840213807725	10.51140163603613
C	-1.39123150430009	10.52050288794545	10.24234881406784
N	-1.39298752823843	10.65390736180825	8.90500086072406
H	-3.72395664969987	13.09871795472489	8.65659287659703
H	-3.69252879135791	12.85688820853359	11.13876810701714
H	-2.15547591317899	11.11927372740220	12.15779770804646
H	-0.71284076720919	9.77029519535305	10.64822408374314
C	1.01170903442066	9.11553548196249	8.88711094327023
C	1.09145690540423	9.20023772589445	6.33415170858504
O	1.72541423700704	8.78554245942954	9.72840072960144
O	1.86281038636777	8.91148670040281	5.52952091929332
C	-1.02903001977615	8.10985729840078	7.48003714308931
O	-1.58664061550521	7.10524123711590	7.38487151254391
C	1.08966810676142	11.69817973698194	7.66164346095219
O	0.38283270808583	12.73537197747462	7.65813508424674
O	2.33898923359082	11.65547808311839	7.68619761233168
K	2.46843492795836	14.16270856518879	7.69019826804467

g) **[1]K**

Re	-0.55745862163107	11.05836131883922	8.07544106346498

C	-2.41998456623367	14.91283471649423	9.37241804516596
C	-1.61127259198875	12.81922656273738	5.79756408530763
C	-1.89927986624212	13.24681962498213	4.46730869520113
C	-1.59129743885759	12.44724111848934	3.39177460772099
C	-0.97509598844345	11.18874389773034	3.61825669004346
C	-0.70921532908530	10.82087693720884	4.92659404100516
N	-1.00361937912020	11.58302415301967	5.99189664273064
H	-2.37779712484178	14.21373702648562	4.30874675690489
H	-1.82706491367658	12.78065078433188	2.37766721605266
H	-0.71353781868774	10.51834166698339	2.79931550623393
H	-0.23436812929033	9.86449350142398	5.15195064685425
C	-1.91383526314921	13.57489366615654	6.96998843699745
C	-2.57754265215256	14.83797603903198	6.96599614940393
C	-2.83233628133757	15.49675658749684	8.14547976200969
C	-1.78654012583140	13.68333235620236	9.32664970438376
N	-1.53973125582497	13.01322576914946	8.18904456496142
H	-2.89384190631214	15.27543074444989	6.01858830155130
H	-3.35087710048107	16.45858834620784	8.13536461612164
H	-2.60526616938685	15.40060806536648	10.32977545154713
H	-1.45158576751978	13.19102484244521	10.24111187993312
C	-0.12927806936549	10.87716883190706	9.94780909713048
C	0.35579898853598	9.38932769852676	7.73710666642551
O	0.14784060669167	10.81566483797334	11.06896822150837
O	0.90344594588892	8.39666938090220	7.50847130595425
C	-2.28222509949641	10.11794257109302	8.31351817824472
O	-3.27866705131195	9.56613974849797	8.47621987174804
C	1.31146550346209	12.17925764355053	7.81377418207992
O	1.26392255548462	13.41635794107297	7.11439588725329
O	2.44463585949241	11.92226874229008	8.19801266933563
H	0.35394291066557	13.58985135139727	6.81850058325425
K	3.78704763704678	13.82868188755547	7.24848812847007

h) **^{2}L**

135

Re	-0.58304113981426	10.67768202854153	8.80066334965387
C	-2.12464590017066	14.51104229410539	10.46668527728893
C	-1.96390875992692	12.48042679805341	6.77299949482462
C	-2.51328367357444	12.90568214127994	5.53022981057590
C	-2.35856028693818	12.14560948827313	4.39509612013798
C	-1.63720366721717	10.92311654654211	4.47245842584705
C	-1.13058213546118	10.54356469669214	5.69816218839422
N	-1.27803595073867	11.27094358042758	6.82438680902398
H	-3.05897625893970	13.84819601226967	5.48405352843587
H	-2.78656388636956	12.48251850494028	3.44810155464619
H	-1.47939436906491	10.28874674403229	3.59996908997836
H	-0.57851076242789	9.60888762537142	5.80676549578696
C	-2.05762260715752	13.21320926918336	7.99008004519949
C	-2.73222264931779	14.45863918316244	8.13328038445113
C	-2.77504373131102	15.09953092880942	9.34843970636970
C	-1.47778656231724	13.30656380986020	10.28410370959050
N	-1.43815272178783	12.65415154199794	9.10435834038505
H	-3.22296537052987	14.90121786808863	7.26636558055789
H	-3.30199064376099	16.05059854837059	9.45145362336434
H	-2.12869495092406	14.98606058828586	11.44774314393508
H	-0.96045229320255	12.82050213502268	11.11250854723924
C	-0.05361392862148	10.36353865902004	10.65407524183307
C	0.08734567734006	8.91379911188901	8.30498110778960
O	0.25252387251591	10.20493955246550	11.75122852032079
O	0.48157314515257	7.88028586853893	7.99078289294959
C	-2.41747141170722	9.90542089477265	9.16834234230138
O	-3.44280057290237	9.45918080917744	9.38325886151679
C	1.23560923614039	11.45702376896927	8.40635897983616
O	2.28066318103458	11.85665350085681	8.19056469676602

i) **^{2}2^{+}**

C	-8.76481474819572	16.33298327228164	11.10803041454820
C	-9.38504787729820	15.43633897017013	7.01519328802895
C	-10.35097668768986	15.91119264264387	6.12330514293621
C	-10.38621051923286	15.41145053347064	4.82341060394066
C	-9.44797265000199	14.44638942985877	4.44148550572934
C	-8.50842834889684	14.02185557271130	5.37582991647084
N	-8.46373484996564	14.49570977877911	6.62908443505906
H	-11.07297191007072	16.65872935049261	6.45246121771321
H	-11.13839944749477	15.76786337254046	4.11701767385600
H	-9.43979724837486	14.02007363993797	3.43756889631230
H	-7.76565130676681	13.26951584181486	5.11249962236924
C	-9.27666700199458	15.85441098151986	8.41683107572718
C	-10.04769961573443	16.79292180539875	9.09092263322916
C	-9.77770384622599	17.03885862792054	10.45307812549512
C	-8.01944773354112	15.38675642969552	10.39709610730881
N	-8.28885538027825	15.18267622191560	9.06412837128850
H	-10.84136004545143	17.33512612995849	8.57624852581865
H	-10.36559153718061	17.77977699599547	10.99725925106485
H	-8.54572276020502	16.52148086665903	12.16067706591054
C	-4.39160793244825	11.23777036691644	6.25639467091738
C	-5.43516642262164	12.87437937588191	10.00515648345800
C	-4.89444513083198	12.67692129891371	11.26928622344927
C	-5.39746162265627	13.43541890016904	12.34691358594236
C	-6.41594719689307	14.36951291172935	12.13734759027477
C	-6.94406099568880	14.52762792978341	10.85231613057226
N	-6.44058409135518	13.77007073155524	9.82017728535653
H	-4.09338451436930	11.95476168284270	11.42933212161784
H	-4.98275123811821	13.29159212923286	13.34625644412607
H	-6.80514999026387	14.96628756976561	12.96424607755656
C	-5.04371391407008	12.22956434007029	8.74715509390376
C	-4.00852421385175	11.29708438533649	8.62979416401996
C	-3.67740716708073	10.79363336251192	7.37404895581020
C	-5.42406159328823	12.15219890402591	6.44430067973103

N	-5.75957135039959	12.63638486933199	7.64932796003603
H	-3.46207790464969	10.97800937497093	9.51760077602992
H	-2.86685556419051	10.07025579471873	7.26547061350011
H	-4.15621957923461	10.87978631779808	5.25308503897552
H	-6.00250365469497	12.51559959786269	5.59533357455762
Fe	-7.21531891269331	13.96675975081761	8.13173347135768

j) $^{2}[Fe(qpy)(CO_2)]^{+}$

C	-9.14685443583626	15.86675157751225	11.15324794530189
C	-9.80087052919364	15.01284270441841	7.04759165385114
C	-10.73947842187526	15.54876316094100	6.16918618011994
C	-10.79948718855394	15.06167927819643	4.86047901540644
C	-9.91353431948296	14.05731069110828	4.47212622585926
C	-8.99750195846897	13.56968895727210	5.40594290980637
N	-8.93503461834787	14.02950054312540	6.65771971918444
H	-11.42274952760341	16.32941089487334	6.50366709794473
H	-11.53255196996505	15.46264928751403	4.15820812321747
H	-9.92720764527940	13.64154798010956	3.46372347258552
H	-8.29723323973755	12.77608570825033	5.14451375730694
C	-9.67085045597862	15.42348076168785	8.46338338052028
C	-10.40229718742076	16.40610956376399	9.13957317535244
C	-10.12495020566281	16.62300834611582	10.49239026787934
C	-8.46029374283157	14.89569456710988	10.42594616987092
N	-8.74346987130109	14.71695584422478	9.11972171711058
H	-11.16347270430109	16.99248208287171	8.62554430286635
H	-10.67746895031179	17.38787213788483	11.04113969983146
H	-8.92191449389557	16.04173480578637	12.20580001084607
C	-4.66613807167525	10.86670288323576	6.29637832197244
C	-5.84397827477620	12.40622041777069	10.04953548253137
C	-5.27876002658181	12.23827485447649	11.31879070812457
C	-5.80341449295811	12.97439452720175	12.38399961081983
C	-6.84944342001979	13.88421585858569	12.17211694237495

C	-7.37939899547303	13.99399982526749	10.88940996246710
N	-6.88673594805539	13.23264112556974	9.88493018711465
H	-4.44238591840804	11.55721552282489	11.47513041863147
H	-5.38062902468373	12.85344356181228	13.38304188444124
H	-7.24075609304891	14.48954205297443	12.99044533022770
C	-5.40223004246436	11.79079669889162	8.78070605841901
C	-4.34351657168757	10.88886117246857	8.67989877604658
C	-3.97079181045715	10.41738289737913	7.41913880275696
C	-5.72578280377827	11.75771431265745	6.47636632176954
N	-6.09310437523281	12.20404070335686	7.67944896536518
H	-3.81350267861972	10.56020304212466	9.57392283764782
H	-3.14329138445403	9.71276752757950	7.31668739845818
H	-4.39970136960295	10.53133660051697	5.29286077717709
H	-6.30068267996899	12.12323625451651	5.62454171557007
Fe	-7.76351647885427	13.35131701493529	8.19100801446654
C	-9.10089165874536	11.94183422513858	8.90651632949568
O	-9.75855080305406	12.06507062462418	9.90931656766602
O	-8.87452107135185	11.15980258432391	7.99581183259363

k) $^{1}[Fe(qpy)(CO_2)]$

C	-9.16983206928820	15.84894283604545	11.16990843889976
C	-9.78876861030715	15.06073759986905	7.05962311229672
C	-10.73228512948203	15.58268517170425	6.16475138907616
C	-10.81025558860101	15.06670489057332	4.87541558886401
C	-9.93353047850888	14.03548834301179	4.50313683450533
C	-9.01480504749095	13.56824917793297	5.43461302768346
N	-8.92499276248360	14.06127662125797	6.68083431745313
H	-11.40924159134357	16.37319202578780	6.49068580540386
H	-11.54752950714492	15.45371454143775	4.16925033297799
H	-9.96588875928407	13.59228767211515	3.50659822187808
H	-8.32737635493065	12.75790359284274	5.19003612931793
C	-9.65516391801820	15.45918971777055	8.45825835250899

C	-10.37181128372302	16.43908823956374	9.14122019864818
C	-10.12265071320940	16.63856228044908	10.50965082476524
C	-8.47306704767828	14.87687447306860	10.45407223956155
N	-8.72299243923295	14.70646291012758	9.11144645655501
H	-11.11470182931301	17.04305681385861	8.61871878165974
H	-10.67245191783753	17.40479105547363	11.05873329617422
H	-8.96091646412174	16.00281862082516	12.23033417075863
C	-4.68233948893696	10.82927508276093	6.31606158978284
C	-5.82661666527933	12.43456559471983	10.05186708416427
C	-5.25027246691556	12.28319517846919	11.31054116341638
C	-5.78873159516948	13.00119099096764	12.39304138393192
C	-6.85780200244362	13.87829647049377	12.18853174117653
C	-7.40901362989140	13.99285221350481	10.90953318614168
N	-6.90432160606580	13.23857776730677	9.87982142458205
H	-4.39183310342211	11.62686117036475	11.45563960582595
H	-5.35374107164222	12.88805355848769	13.38783039330967
H	-7.25938484173610	14.47095844071211	13.01242343341762
C	-5.38281768060275	11.81482554892803	8.79439850453498
C	-4.32156470867531	10.90725072286982	8.69393180908347
C	-3.96642760462426	10.40852595370993	7.44286246468217
C	-5.73526175904918	11.72356469179962	6.49227522272782
N	-6.08384935099787	12.20591924928790	7.69060974920171
H	-3.78274860223336	10.59390227345476	9.58855523301875
H	-3.14038620584513	9.70114773496422	7.34498181345510
H	-4.43088153247879	10.46733042835389	5.31774251617541
H	-6.32529311969798	12.07089017325963	5.64281408953124
Fe	-7.80567792538402	13.34714183744753	8.21844038021699
C	-9.06558518104011	11.91815181458437	8.74156832376731
O	-9.69603467967207	11.88946360857747	9.78592145215165
O	-8.96009912619639	11.18061609225845	7.75322798771536

l) $^{1}[Fe(qpy)(CO_2H)]^{+}$

C	-11.05502504125215	16.20362424904290	9.07253371145129
C	-9.94739137610126	14.17171512925731	5.52451320807832
C	-10.44724065483002	14.33907501325430	4.23405172089210
C	-9.84433080905723	13.65630258102076	3.17508981655054
C	-8.76802796482472	12.81183362745162	3.44491738201710
C	-8.32075414972589	12.69751078130814	4.76211385732169
N	-8.87839187194965	13.35993616610105	5.78157220998857
H	-11.28931581406254	15.00770847760762	4.05391066504556
H	-10.21550171809429	13.78898734979086	2.15660814506895
H	-8.26547386726640	12.25318417997248	2.65442637384483
H	-7.47247986960189	12.05622893628476	5.00242392054531
C	-10.47317062104604	14.89753730131461	6.69984872388239
C	-11.56887328977203	15.76699617639244	6.74193279286042
C	-11.86574581271531	16.40641482288577	7.94897857272072
C	-9.97206103397493	15.33153252074867	8.96291848278669
N	-9.74030266834192	14.68180563923847	7.80041667045030
H	-12.17475145655878	15.95118629037859	5.85466159374002
H	-12.72034764850525	17.08252725295869	8.01063834042788
H	-11.26032367917074	16.72576293714960	10.00750635344647
C	-4.57868667399243	11.14030937512018	7.93320208663140
C	-6.90461808805375	13.84813695092663	10.19526443341406
C	-6.71537082183848	14.34985855081109	11.48738778103364
C	-7.67403571652536	15.22455375255735	12.00712540552331
C	-8.79442358116786	15.58475310659239	11.24718575482089
C	-8.92538242845765	15.04733335147336	9.96756577890546
N	-7.98549246087602	14.20268340028008	9.48968793928260
H	-5.84477241001875	14.06508918239354	12.07805790410781
H	-7.54541741970469	15.63443654610278	13.01038993517485
H	-9.54254894709635	16.27269488027278	11.64249586772869
C	-6.03174648708202	12.91328150810301	9.45400912498244
C	-4.86865180433221	12.35807831564871	9.98388879648639
C	-4.13229971673154	11.45185791629825	9.21674864006285
C	-5.74226869739704	11.74969003171746	7.46319644728839

N	-6.46196861808794	12.60662023077885	8.19407087259363
H	-4.54177699720896	12.63057635775564	10.98774973435972
H	-3.22735472675366	10.99526430324680	9.62209466183714
H	-4.04010555060686	10.44068541392132	7.29292287817494
H	-6.11225761936992	11.53046118829126	6.46150049886630
Fe	-8.25322063230628	13.49884794161776	7.75031788950537
C	-9.22096342186477	12.02699418505640	8.46501768940629
O	-9.95004215676895	12.01435472596690	9.43404970152452
O	-9.03282368285709	10.81437185814531	7.83990514747868
H	-8.46358090904929	10.93165468976153	7.06486344269031

m) $^{1}[Fe(qpy)(CO]^{2+}$

C	-8.79695176631375	16.25718654836620	11.13126533402769
C	-9.40765068297931	15.37350981144009	7.02544059103194
C	-10.31944097559044	15.91603462963317	6.12565976836944
C	-10.31750582843133	15.46647810333879	4.80148816791960
C	-9.39822472076012	14.49153807458361	4.42014074839285
C	-8.51521264204449	13.98805372153415	5.37631891419835
N	-8.51219338233613	14.41316146882229	6.64162950098394
H	-11.02675441500805	16.67736710650366	6.45389583884791
H	-11.02924747226305	15.87525245181571	4.08233282583407
H	-9.35835440588020	14.10551254635179	3.40086941504680
H	-7.79698478327693	13.21325538422526	5.11450559759698
C	-9.32095039712135	15.76999390092674	8.44715566049823
C	-10.06446896045511	16.74998140108358	9.11268444410451
C	-9.79376611872357	16.98309095885606	10.46366591523885
C	-8.09728048645952	15.28811202968691	10.41665865357760
N	-8.39430653423939	15.07638833213285	9.11717701535086
H	-10.82910495184727	17.32399932326761	8.59024336899864
H	-10.36021107325842	17.74308275552664	11.00440260613060
H	-8.56564315150615	16.45541402010842	12.17816512959211
C	-4.42884106999422	11.17880961664796	6.27947489289031

C	-5.43773647962807	12.84995699735744	10.02534297353626
C	-4.81797579926163	12.73656051636839	11.27348415280971
C	-5.32082470591485	13.49610522095350	12.33325138871795
C	-6.39880495360480	14.37216215087607	12.14129046266203
C	-6.97996788767299	14.43119784513897	10.87754405547302
N	-6.49514734775125	13.65584194553920	9.88448519876211
H	-3.96251549415996	12.07764464440912	11.41915502210008
H	-4.85770545358494	13.41577041165423	13.31825733367592
H	-6.77318815622628	14.98919715262749	12.95853654732814
C	-5.05074480840228	12.19571266170437	8.75778936914800
C	-3.94312893459122	11.36391465122431	8.62585250707598
C	-3.62493554255294	10.84607425814342	7.36655688676627
C	-5.53326225285156	12.00718993907933	6.48764776484690
N	-5.84374991969332	12.50476431983074	7.68740916527022
H	-3.32845770605163	11.12739791335242	9.49432794781190
H	-2.75649956744317	10.19688134726107	7.24120321862533
H	-4.21419825927301	10.80538985054676	5.27734638986279
H	-6.18333064475216	12.27986616317875	5.65846786367488
Fe	-7.38662971440310	13.74142662542925	8.19657411300884
C	-8.54054224717812	12.45414027459661	8.55487346856206
O	-9.30696665151340	11.64991823887600	8.78812093564972

n) ^{3}**3**^{0}

C	-2.31187353756197	-0.99838600616794	-3.53295220675675
C	-4.13332608935709	-1.46237891085961	-4.93859106581516
C	-1.43881640857606	-1.30273273102407	-4.58227770989195
C	-3.31829812088540	-1.76374511831574	-6.01073066015176
C	-1.92672436119481	-1.67694079929810	-5.83525528365322
H	-5.22156635087997	-1.50315269062665	-5.03279899821313
H	-0.36300782065135	-1.23708435734324	-4.43553848513916
H	-3.76156938420978	-2.05571281777807	-6.96491318200681
H	-1.23941652150728	-1.89162026843727	-6.65544659250018

N	-3.66377558351255	-1.07574204291555	-3.72743166563480
C	-1.80579659552237	-0.54435947269235	-2.13864189138583
C	-0.27195978272192	-0.59074264580019	-2.09721612152339
H	0.09697388669357	-0.26638082160027	-1.11617275697749
H	0.10452410407755	-1.60302578401711	-2.28939268650185
H	0.15742648670095	0.08053142077213	-2.84963053074056
C	-2.26813771698980	0.92113529237516	-1.89688368583923
C	-1.41115095799520	1.99725286460886	-1.72523657705483
C	-1.96955063344851	3.29713074731033	-1.58249748969731
C	-4.17237121176762	2.34434363301323	-1.78527607887699
C	-3.33987741681933	3.47195162231576	-1.61699933548637
H	-0.33066563935110	1.87562190788112	-1.70325125919273
H	-1.30881625794666	4.15692981985507	-1.45124135594550
H	-3.77360796159999	4.46825792788949	-1.51781858168958
C	-2.38628640376825	-1.50753041207764	-1.07152462935890
C	-1.56674189309324	-2.26446474849938	-0.22748788262685
C	-2.11137470935792	-3.15257476943183	0.70224278476604
C	-4.27333385323120	-2.50280554062493	-0.08920163429243
C	-3.50974401047975	-3.27710489487456	0.76134657470530
H	-0.48496754229634	-2.16673170335219	-0.28098393235739
H	-1.46114446981031	-3.72843670014629	1.36290344635366
H	-5.36462631405111	-2.55726082499019	-0.06471114853582
H	-3.99842234018123	-3.95617763889003	1.46262005174808
N	-3.59733606174616	1.10462348078536	-1.91058911070783
N	-3.74790009113420	-1.62920025408370	-0.98614824285970
C	-5.60626672419920	2.33062005321083	-1.87242881804011
C	-6.42690706034616	3.47673056742158	-1.78851033562022
C	-7.51038490802956	0.96131278435674	-2.18975797173208
C	-7.79735166443475	3.33954914704952	-1.90519410848793
C	-8.36495826158539	2.06455464035209	-2.10893454966751
H	-5.97472748806812	4.45625738655693	-1.63208686979247
H	-8.44554689955906	4.21662248848026	-1.83979451047600
H	-9.44290483199776	1.93612945798590	-2.19458690006271

N	-6.16264717860812	1.07715144833444	-2.07672454320125
N	-7.97590438527640	-0.31866167516294	-2.38886692628338
H	-7.23560717439536	-0.98307718729179	-2.60435063696239
C	-9.28767042769353	-0.61420822270684	-2.92897946049734
H	-9.52665341643153	0.06921428865332	-3.76841924308027
H	-10.06327665359687	-0.43798489657652	-2.16119943892604
C	-9.34840904960105	-2.06122531458055	-3.40376080138835
H	-10.33754453616460	-2.29851124893981	-3.82322929062934
H	-8.59788971080210	-2.24903618904917	-4.18964502089832
H	-9.14772454012335	-2.75596613989138	-2.57232614767737
Fe	-4.80543034491060	-0.44249613716217	-2.20064235573651

o) $^{3}[Fe(bpy^{NHEt}PY2Me)(CO_2)]$

C	-2.21041411728392	-1.03023180821797	-3.50218591379562
C	-3.97080925486603	-1.23722904903945	-5.02867472038062
C	-1.29156653415545	-1.29557339734177	-4.52541364798917
C	-3.10617797564870	-1.45364104434175	-6.09296063620429
C	-1.73721872813512	-1.49634770635378	-5.83141113092382
H	-5.05329514585884	-1.28251568029270	-5.14618351368783
H	-0.22538224783953	-1.33799936677914	-4.31947601617756
H	-3.50914783778107	-1.60978438304915	-7.09512905252284
H	-1.01817945448513	-1.68961318940686	-6.63068929708927
N	-3.53204618644349	-1.02437307877709	-3.77657105037099
C	-1.75444906989226	-0.64318016148600	-2.07099054857815
C	-0.22020447778570	-0.64157244246765	-2.00353829476425
H	0.12554018338246	-0.29328659501524	-1.02269352090657
H	0.18549162489514	-1.64713245624701	-2.17395825088505
H	0.20529797966954	0.02818575761674	-2.75835821489048
C	-2.29515711346345	0.78851083468219	-1.83680717176510
C	-1.46199366985176	1.88936602803605	-1.71389507951464
C	-2.02898751821395	3.17951267715955	-1.63703204839112
C	-4.21027648921823	2.16677291225569	-1.82816527893503

C	-3.40668640163112	3.31115366552650	-1.71315134671496
H	-0.38026079045539	1.78180077706280	-1.68920819551371
H	-1.38851171732084	4.05808776679996	-1.54199556215401
H	-3.86127978653952	4.30080345186360	-1.69096355797884
C	-2.31082376631368	-1.60213108101764	-0.99058597532585
C	-1.47122808488349	-2.25478908466792	-0.07578922215789
C	-2.01717852018533	-3.07406773655208	0.91207888596293
C	-4.17457681956254	-2.57535790904095	0.01913005231143
C	-3.40011312940910	-3.23927480807395	0.96452706913672
H	-0.39250664674474	-2.13177222154494	-0.11746320780959
H	-1.36109599162770	-3.57842680027491	1.62539497824865
H	-5.25881732926566	-2.68222611919744	-0.03723043290856
H	-3.87799526933815	-3.87232444350183	1.71397800831901
N	-3.64319295885097	0.91941930669321	-1.85465083116932
N	-3.64192926538106	-1.78016909149275	-0.92151573246353
C	-5.65640416097444	2.19279628549538	-1.97186577582771
C	-6.37283099718902	3.40015547708160	-2.03551157804814
C	-7.63517735275236	0.94141930339545	-2.20524177402739
C	-7.74901138307026	3.35977735424075	-2.21593771739786
C	-8.39354599766509	2.12983059854143	-2.29885006508312
H	-5.85414562024570	4.35301422418095	-1.95509320324287
H	-8.32646659181655	4.28554304720538	-2.27521382387214
H	-9.47594742709426	2.07021376846997	-2.39680886909776
N	-6.26970581162327	0.97081120169014	-2.08819429213283
N	-8.23873680683391	-0.26547207628131	-2.23280033636101
H	-7.69537487821607	-1.07308734354022	-1.91300373884469
C	-9.60033001691797	-0.50610255517091	-2.65578982952813
H	-9.81920928315355	0.11821798408490	-3.54130536773679
H	-10.33273639458309	-0.20507876973877	-1.87878160413056
C	-9.78268137057308	-1.98053963288499	-3.00779555104213
H	-10.78827761596079	-2.16029795267008	-3.41845808460590
H	-9.02972955412440	-2.29548389755656	-3.74619438764159
H	-9.65034307310567	-2.61716531919943	-2.11865689478135

Fe	-4.86944776751684	-0.64915319984776	-2.25698469629976
C	-6.05631487726101	-2.15288156823311	-2.78971076288263
O	-6.54406195839962	-2.35156697744184	-1.64283663934806
O	-6.29257886743822	-2.59002653933729	-3.90586257607763

p) $^{3}[Fe(bpy^{NHEt}PY2Me)(CO_2H)]^{+}$

C	-2.44855499126808	-1.02905696617379	-3.45530494978651
C	-4.43204860921377	-1.51186992082029	-4.57207073693402
C	-1.72612152781222	-1.16540729717853	-4.64499667908695
C	-3.78703930081357	-1.62937221635685	-5.79879217720976
C	-2.40421175733992	-1.45084998635752	-5.83146307865950
H	-5.50571729868733	-1.67645467969000	-4.47950270273898
H	-0.64749755171890	-1.03034293398740	-4.66384710249800
H	-4.35960516716838	-1.86242587208968	-6.69751153990706
H	-1.84973027121565	-1.53546997313751	-6.76864952346337
N	-3.78098375476403	-1.20122354168689	-3.44441665871578
C	-1.77895991473465	-0.60675996337981	-2.12291450089438
C	-0.25189108880824	-0.59151038352659	-2.27444469032523
H	0.23129834424329	-0.31927436011304	-1.32803949810733
H	0.12516416548007	-1.57335290693296	-2.58409978848948
H	0.05495882851395	0.13906161526882	-3.03164782417552
C	-2.28844173852326	0.81531737058379	-1.81245323742626
C	-1.45335316231306	1.92312904993777	-1.64722973802595
C	-2.02754980789668	3.18243005950027	-1.45606236829350
C	-4.19352560794931	2.17144759263769	-1.62905597596671
C	-3.41373859576096	3.31839413665444	-1.46060503049490
H	-0.37132020222656	1.82192587687979	-1.67728758290657
H	-1.39008554984018	4.05872331413609	-1.32446039308099
H	-3.87658890172621	4.29840113158041	-1.35258681341205
C	-2.19171225507144	-1.55367630028433	-0.97455666547754
C	-1.26592943776426	-2.40923574192333	-0.36694461989025
C	-1.65059121863114	-3.19317505057599	0.72056592559432
C	-3.83422242575499	-2.25038158103764	0.52603743777276

C	-2.95640694811419	-3.09321822440976	1.19669287244687
H	-0.24411948776498	-2.47231788065207	-0.73113470884090
H	-0.92762453332561	-3.86167346000721	1.19247059812927
H	-4.87559771159310	-2.15851501116161	0.82948484242984
H	-3.30293350972015	-3.66524885389671	2.05860453725701
N	-3.61769122953893	0.95993190321514	-1.76248867010311
N	-3.46902318333093	-1.52111260456275	-0.54147084709436
C	-5.65738817893298	2.16147506925030	-1.79528359088765
C	-6.42812067845984	3.31216779718269	-1.71426557407153
C	-7.47137755800577	0.87753593328452	-2.55141065688163
C	-7.79052653376009	3.22064980329740	-2.03731875927884
C	-8.31367273939438	2.02027111884026	-2.47458606778430
H	-5.98417576711930	4.26088674473172	-1.41789891209155
H	-8.42979031234383	4.10336181746336	-1.97049430156923
H	-9.35655597927317	1.94254918126372	-2.77535487827552
N	-6.18108064842002	0.94302531850415	-2.12905720164159
N	-7.92233776234184	-0.27890148003135	-3.07346669809836
H	-7.30073148255411	-1.07169658320908	-3.07320540384384
C	-9.28869505823599	-0.51468437293360	-3.52203407017514
H	-9.54838755782914	0.21600853651434	-4.30647510187887
H	-9.99362832888453	-0.35540350926994	-2.68754910680541
C	-9.41986192986926	-1.93218912860832	-4.06123964581671
H	-10.43126630284555	-2.09349379489073	-4.46169840214656
H	-8.69965536424255	-2.11385950289204	-4.87475180244763
H	-9.23885655679550	-2.67634844524826	-3.27055447305174
Fe	-4.86448656775940	-0.57630173379068	-1.74100126467183
C	-6.17130975608960	-1.98555542561050	-1.38862415582203
O	-6.77348035324357	-2.12134811548129	-0.33226446352081
O	-6.43933019740803	-2.89550461338279	-2.38414177922487
H	-7.10464387003776	-3.53194441243582	-2.05164998363922

q) $^{3}[Fe(bpy^{NHEt}PY2Me)(CO)]^{2+}$

C	-2.31540840500000	-1.02788782200000	-3.45260564300000
C	-4.19284040900000	-1.35747612700000	-4.79915370800000
C	-1.48569055100000	-1.17026474900000	-4.57164158400000
C	-3.42900094700000	-1.46034454700000	-5.95601630400000
C	-2.04401364800000	-1.37460188200000	-5.83440723600000
H	-5.27578640300000	-1.46869001000000	-4.81876167000000
H	-0.40551898700000	-1.10376162100000	-4.47464461400000
H	-3.91742936400000	-1.61954276800000	-6.91871915400000
H	-1.39696594800000	-1.46383657000000	-6.70995163000000
N	-3.64963518700000	-1.13996343200000	-3.59419603500000
C	-1.75528187700000	-0.64013061500000	-2.06064354100000
C	-0.22122375800000	-0.61452126200000	-2.10964713400000
H	0.19164343100000	-0.29410508700000	-1.14610823100000
H	0.18923221500000	-1.60388523700000	-2.35096760300000
H	0.12931327200000	0.09792160700000	-2.86361790500000
C	-2.29422620000000	0.79398311000000	-1.77457687200000
C	-1.45502462200000	1.88369022700000	-1.60649374000000
C	-2.04977838200000	3.17401726600000	-1.48145953300000
C	-4.23498005100000	2.16298632700000	-1.74575808500000
C	-3.41559200400000	3.30861117400000	-1.56647227200000
H	-0.37337034700000	1.78614026700000	-1.58538801600000
H	-1.41603467300000	4.05255623400000	-1.34262349400000
H	-3.86920247300000	4.29749338900000	-1.50357857600000
C	-2.21684676900000	-1.62280733200000	-0.95718697000000
C	-1.29506538400000	-2.28991224800000	-0.13649307500000
C	-1.74125883000000	-3.13982660800000	0.87504152100000
C	-3.97094605800000	-2.63494299300000	0.19815758300000
C	-3.11182277300000	-3.31590961900000	1.05250731100000
H	-0.22624625200000	-2.15346315000000	-0.27323547000000
H	-1.01959357800000	-3.65442400400000	1.51324755800000
H	-5.05430525500000	-2.74273034400000	0.26538540100000
H	-3.51745130400000	-3.96738258900000	1.82846878500000
N	-3.62752769200000	0.92130280100000	-1.79633653200000

N	-3.53534744300000	-1.81874440100000	-0.77379288700000
C	-5.65550702400000	2.19593046600000	-1.94890549800000
C	-6.37035214100000	3.41250656600000	-2.06995325500000
C	-7.63885334000000	0.97088901800000	-2.36708264800000
C	-7.72277441800000	3.38465438900000	-2.35150541200000
C	-8.38265349000000	2.15691684900000	-2.50732438500000
H	-5.85049789300000	4.36323457900000	-1.96531879700000
H	-8.28162163900000	4.31871066800000	-2.45275508500000
H	-9.45102858200000	2.11469597900000	-2.70799819300000
N	-6.30732720100000	0.98004823800000	-2.09080672900000
N	-8.22406213400000	-0.25373190400000	-2.52305122000000
H	-7.78941898300000	-1.01591302000000	-2.00774218300000
C	-9.58467445000000	-0.45548220100000	-2.96494205900000
H	-9.76764511500000	0.17511533500000	-3.85271347600000
H	-10.32660163100000	-0.13508035100000	-2.20358017100000
C	-9.80132919400000	-1.92356859900000	-3.32032090400000
H	-10.80451948800000	-2.08159652400000	-3.74566343400000
H	-9.05066301500000	-2.25826736000000	-4.05268256700000
H	-9.70069971600000	-2.56464038900000	-2.42942867700000
Fe	-4.93338023100000	-0.67249924200000	-1.96482695600000
C	-5.96072509900000	-2.33903317500000	-2.38797331000000
O	-6.66182196000000	-3.26728800200000	-2.70247122400000

r) ^{2}**5**^{0}

C	-8.928494	16.150535	11.176160
C	-9.354396	15.358949	7.055383
C	-10.249208	15.840115	6.093878
C	-10.073069	15.530415	4.752390
C	-8.958723	14.747481	4.392545
C	-8.111040	14.281601	5.381440
N	-8.285629	14.522617	6.707676
H	-11.077723	16.472001	6.414886

H	-10.765917	15.901532	3.997915
H	-8.748298	14.505436	3.351360
H	-7.230869	13.697652	5.120010
C	-9.374169	15.691738	8.459888
C	-10.242785	16.525018	9.167205
C	-10.020011	16.753877	10.529956
C	-8.086497	15.315649	10.449956
N	-8.324083	15.091175	9.108642
H	-11.081962	16.999072	8.658313
H	-10.690716	17.405770	11.088576
H	-8.739493	16.326982	12.234823
C	-4.901936	10.937674	6.341991
C	-5.314414	13.040243	9.949863
C	-4.653700	12.976353	11.178070
C	-5.139896	13.718273	12.260774
C	-6.285161	14.517282	12.109271
C	-6.916041	14.571690	10.871378
N	-6.419915	13.841788	9.809730
H	-3.771504	12.346663	11.292638
H	-4.632709	13.671117	13.223789
H	-6.678370	15.093651	12.946413
C	-5.050345	12.328218	8.722246
C	-4.030567	11.390109	8.530559
C	-3.932932	10.692563	7.334556
C	-5.881707	11.886679	6.572241
N	-5.972965	12.623961	7.710419
H	-3.323505	11.207884	9.340261
H	-3.141343	9.960519	7.178028
H	-4.898385	10.391868	5.399117
H	-6.653911	12.070480	5.827940
Co	-7.361481	13.903101	8.087118

s) ^{2}[Co(qpy)(CO_2)]

C	-8.72900401932919	16.25660481682916	11.07590858142024
C	-9.46558744835759	15.51415560537824	6.97658424147580
C	-10.40652844347246	16.12615452959250	6.13278036647608
C	-10.53334539337892	15.69515236974473	4.81811531457657
C	-9.71075524374746	14.65038440212353	4.36592447975407
C	-8.79277440049496	14.10134925351642	5.25333371985531
N	-8.65678947842548	14.51683110721673	6.51779564253605
H	-11.03934173132979	16.92761546597912	6.51512688666132
H	-11.26503460156437	16.15753244523876	4.15215910646519
H	-9.78922688144792	14.26202086194840	3.34892621927365
H	-8.14250530817474	13.27739722283632	4.95180682594590
C	-9.29146370481471	15.85964733445626	8.39380772910620
C	-10.01380555814757	16.82402306180557	9.09848663787466
C	-9.72652281341276	17.02282887380323	10.45737571137878
C	-8.03993050125311	15.30601952662775	10.32097125015872
N	-8.33920739804183	15.12947882320485	9.01031420291549
H	-10.78487109959883	17.41742422815535	8.60589682150712
H	-10.27725361043784	17.77168358997229	11.02955947820031
H	-8.48336056096901	16.41530675961894	12.12745764720492
C	-4.19100343797139	10.99257112293650	6.40091052143452
C	-5.36938580299418	12.86264369429725	10.00886831903906
C	-4.85545897268660	12.69390620592213	11.29503477809322
C	-5.42556773714039	13.42563110644922	12.34858647655216
C	-6.47293104557723	14.31926284494896	12.08967521092294
C	-6.94879776119822	14.44089590940210	10.78279662856923
N	-6.39862009615928	13.70393727242736	9.78764402120342
H	-4.03536625560258	12.00126387513072	11.48802039666907
H	-5.04961401618027	13.29586422397417	13.36554825652224
H	-6.92021390400680	14.89834007341133	12.89918661531035
C	-4.90363879735201	12.17891934702810	8.79268299042590
C	-3.78788952976506	11.32761347595129	8.74818520803158
C	-3.42275185169617	10.73248829914306	7.54653550821456

C	-5.28705886491210	11.83947748510661	6.52162489458230
N	-5.63593428460352	12.42881281983959	7.67077129893270
H	-3.21192489225616	11.13607595181049	9.65440155768294
H	-2.55421961773731	10.07201765652757	7.49732415940389
H	-3.94372677290796	10.54153286964376	5.43829932202613
H	-5.92683883310547	12.06000413028478	5.66405008296432
Co	-7.38310005821181	13.62199462109340	7.99062384790499
C	-8.59811063831455	12.10984707007696	8.63004729156722
O	-9.02816849069260	11.93123859143754	9.72586952130957
O	-8.51615486052983	11.72245897010897	7.47761340285129

t) $^{2}[Co(qpy)(CO_2H)]^{+}$

C	-8.928494	16.150535	11.176160
C	-9.354396	15.358949	7.055383
C	-10.249208	15.840115	6.093878
C	-10.073069	15.530415	4.752390
C	-8.958723	14.747481	4.392545
C	-8.111040	14.281601	5.381440
N	-8.285629	14.522617	6.707676
H	-11.077723	16.472001	6.414886
H	-10.765917	15.901532	3.997915
H	-8.748298	14.505436	3.351360
H	-7.230869	13.697652	5.120010
C	-9.374169	15.691738	8.459888
C	-10.242785	16.525018	9.167205
C	-10.020011	16.753877	10.529956
C	-8.086497	15.315649	10.449956
N	-8.324083	15.091175	9.108642
H	-11.081962	16.999072	8.658313
H	-10.690716	17.405770	11.088576
H	-8.739493	16.326982	12.234823
C	-4.901936	10.937674	6.341991

C	-5.314414	13.040243	9.949863
C	-4.653700	12.976353	11.178070
C	-5.139896	13.718273	12.260774
C	-6.285161	14.517282	12.109271
C	-6.916041	14.571690	10.871378
N	-6.419915	13.841788	9.809730
H	-3.771504	12.346663	11.292638
H	-4.632709	13.671117	13.223789
H	-6.678370	15.093651	12.946413
C	-5.050345	12.328218	8.722246
C	-4.030567	11.390109	8.530559
C	-3.932932	10.692563	7.334556
C	-5.881707	11.886679	6.572241
N	-5.972965	12.623961	7.710419
H	-3.323505	11.207884	9.340261
H	-3.141343	9.960519	7.178028
H	-4.898385	10.391868	5.399117
H	-6.653911	12.070480	5.827940
Co	-7.361481	13.903101	8.087118
C	-8.512203	12.203230	8.655605
O	-8.561470	11.627118	9.974418
O	-8.976245	11.450330	7.519204
H	-8.383692	11.597045	6.778289

u) $^{2}[Co(qpy)(CO)]^{2+}$

C	-8.75545101787745	16.31563886572415	11.11392821457803
C	-9.37770206346013	15.38091041896234	7.02034721725670
C	-10.30441372442691	15.89453543041005	6.11581487786434
C	-10.33511214960757	15.39569660493187	4.80995396923895
C	-9.43306555250353	14.39508445242060	4.45136918079555
C	-8.53463570511923	13.92469569677936	5.41028825199331
N	-8.49384052523048	14.40053208081335	6.65779563804704

H	-11.00387527661094	16.67155474783000	6.42551172936952
H	-11.05759848544226	15.78227105260632	4.08812727717717
H	-9.42199836242857	13.96832502093903	3.44702169694889
H	-7.83518222297490	13.12666928513293	5.16497437946335
C	-9.27491017165064	15.80786451140954	8.43048567047462
C	-10.00626664036063	16.80604030353421	9.08618922388637
C	-9.73715692502477	17.05278484495158	10.43542562846274
C	-8.06536188991238	15.32948246763794	10.41105107394846
N	-8.35702049246365	15.10985919422486	9.11051591002293
H	-10.76263270884393	17.38681285979593	8.55791100138569
H	-10.29287429704902	17.82953205771458	10.96516882627758
H	-8.52777448509134	16.52241375979991	12.16075387926188
C	-4.37789232937742	11.30752482658390	6.23695768576473
C	-5.44915922422155	12.85110191372710	10.01865897140873
C	-4.86724858112696	12.68622683020192	11.28139160158058
C	-5.37598355503939	13.42821549874229	12.35131922591432
C	-6.42693075916727	14.33678215770139	12.15673569585511
C	-6.97233868710794	14.45006113331895	10.87934241572775
N	-6.48140558277556	13.69075440390927	9.87552376810595
H	-4.03400800241540	11.99986290074102	11.43369587919368
H	-4.94029094285432	13.30682356650362	13.34562959626674
H	-6.80940313472751	14.93420577611978	12.98556595401747
C	-5.05111486847537	12.22544858936618	8.74193487320592
C	-3.97053495828938	11.35745673747977	8.60387086954811
C	-3.62827903977319	10.88508513095119	7.33299728058178
C	-5.46080976747150	12.16262494703691	6.45284705017740
N	-5.80677269638232	12.60436183495305	7.66538333207356
H	-3.39021242916807	11.05716956069365	9.47693596530332
H	-2.78138306604149	10.20779300237408	7.20329407208028
H	-4.13392935553275	10.98127019229992	5.22427373419067
H	-6.06409790666891	12.50690190236312	5.61401289277186
Co	-7.38261434038315	13.75638842672460	8.21337450786285
C	-8.67684471733807	12.24006803231013	8.64328071462097

O	-9.43127970458415	11.46650429327957	8.95103142129511

v) **$^{2}6^{0}$**

C	2.15365353187102	0.13266554217152	-10.89618516702987
C	3.64325205643890	-0.44425553126592	-9.05132438312780
C	1.04834779083810	0.04854544094256	-10.04372411318399
C	2.53619883450402	-0.55130105082629	-8.19569807758481
C	1.24856026474416	-0.27892406653370	-8.68683646947429
H	0.04119965986739	0.21589378420781	-10.43152348618357
H	2.67529194204418	-0.86206894000072	-7.15783757131477
H	0.38781775039264	-0.35914648259880	-8.01842492111722
N	3.42643078939473	-0.03515750830975	-10.36330130191805
C	2.20766232903847	0.31297651932171	-12.31091188191358
N	3.47282673568731	0.24458018126055	-12.77016187034496
C	1.01968479352759	0.49322812901111	-13.20555189353072
H	1.07076441453338	1.43819490851020	-13.77697715687454
H	0.93718452168861	-0.31742345180053	-13.95149654558385
H	0.08680850272610	0.50476398453293	-12.62482184299177
C	3.80624626819149	0.17591524347948	-14.17879843776787
H	3.55570783681832	-0.82365295795094	-14.58475228993906
H	3.25732482218292	0.91236005565713	-14.79108062255326
C	5.30345358658336	0.43468987209607	-14.29672858909414
H	5.69058228634230	0.13181266305725	-15.28482731413508
H	5.48801600302215	1.51020561548943	-14.17648211969509
N	6.03368947639939	-0.26462179637052	-13.20754428427235
C	7.43565811519266	0.17794807014120	-13.10692141009890
H	7.97434600231103	-0.56378703273450	-12.50490276035994
H	7.91881983566874	0.20341859071183	-14.10025377138846
C	7.52486828283607	1.56311042769323	-12.46281807694824
H	8.59186203422913	1.79686434617604	-12.28255748442053
H	7.15056085746616	2.32186791520610	-13.16459828814422
N	6.72839738201008	1.65678400435105	-11.23848632407480

C	7.39162574667906	1.20410150162868	-10.00916757198224
H	8.46803845662913	1.46632039286163	-10.00950983953208
H	6.91651334819794	1.72796448019146	-9.16629758173497
C	7.20217952704060	-0.30614225097479	-9.79325216305629
H	7.76412403952588	-0.86627750059954	-10.55497636887266
H	7.63061459142783	-0.58836958146212	-8.81869303551166
N	5.78991814540744	-0.58940506586426	-9.91175159842610
C	5.02041039215421	-0.68085299045493	-8.79886044036598
C	5.57526238450961	-0.96640099575183	-7.43047432405140
H	6.12820618142529	-0.10745421468069	-7.00697790715870
H	4.77021890484148	-1.21497239014327	-6.72522243478166
H	6.27672715637105	-1.81850301496597	-7.44275439217567
Co	4.84646038897216	-0.00826884961023	-11.51381168885213
H	6.40272841732268	2.60537000881805	-11.11917884544228
H	6.03012841994524	-1.25965727161774	-13.41158377699045

w) $^2[Co(L)(CO_2)]$

C	2.11518013313697	0.05628818806633	-10.91617638437305
C	3.59737232953440	-0.42397144698942	-9.08918840308243
C	1.01137205000969	-0.01505518078432	-10.06187411729975
C	2.52044671682845	-0.50564183327898	-8.20965182561066
C	1.22044783724479	-0.28020650537841	-8.70103597288439
H	0.00137535819945	0.12000560051238	-10.45252602440065
H	2.67849622347070	-0.75415724854362	-7.15914555969356
H	0.36735273093505	-0.33966815731998	-8.02286920120688
N	3.37043848118886	-0.08782317646668	-10.38883965181084
C	2.16883740840346	0.22092273641239	-12.35414297755627
N	3.40393534884575	0.14324673328004	-12.81352318525884
C	0.97262721731935	0.42263156631875	-13.22954586568542
H	1.04689242618209	1.36910785351526	-13.79183136965969
H	0.88657219232424	-0.38663667909303	-13.97357652489605
H	0.04577682294253	0.44731393533223	-12.64223247487689

C	3.78990648509512	0.17102902973352	-14.21071604132969
H	3.66105148214114	-0.83845470932142	-14.63860290418391
H	3.18230778917248	0.86921362762540	-14.80648805404367
C	5.26063016678276	0.57694884635437	-14.25345509252256
H	5.69249193985650	0.38012647464309	-15.24994835758549
H	5.34253558129843	1.65392103010262	-14.05023608868028
N	6.01553203485912	-0.15115890497377	-13.20640813380107
C	7.41699202222649	0.27762895169905	-13.10600528306768
H	7.95734903880109	-0.49138015092696	-12.53935629960791
H	7.88187698813771	0.31278078001703	-14.10817545107659
C	7.58282340724860	1.64652312656786	-12.44586004142824
H	8.67181773307712	1.84976410794621	-12.37088263588470
H	7.16394647682074	2.42354639965582	-13.10226759518319
N	6.90893785265589	1.75443140834621	-11.15688344320892
C	7.56129744356631	1.10064298978943	-10.02395927054411
H	8.66632327500180	1.18521923918890	-10.07430792206676
H	7.23118345147228	1.62069923274484	-9.11185169530916
C	7.19221664830952	-0.38963623680270	-9.86956836022549
H	7.63165287201743	-0.98614185901283	-10.67860069598006
H	7.61978926020592	-0.76274187552170	-8.92743153245932
N	5.75258481365571	-0.51969045176440	-9.92897420781164
C	5.01738642883680	-0.62434119960889	-8.84320254204752
C	5.56497966376731	-0.86742410200108	-7.46629998457148
H	6.07844788951219	0.02585127659403	-7.07171532367337
H	4.76386460886295	-1.13065312856969	-6.76431004762179
H	6.29536273479102	-1.69098028946214	-7.46805435394581
Co	4.75818643921412	-0.24852678835329	-11.57449005882923
H	6.75562669294459	2.72865780726812	-10.93821331437297
H	5.97625160809998	-1.15376143073721	-13.41653109736437
C	4.47467986027289	-2.20315782533259	-11.85248097955971
O	3.77110385220198	-2.79334152467131	-11.03629218499238
O	5.05617637052823	-2.64678949479943	-12.86573209472555

x) $^{2}[Co(L)(CO_2H)]^{+}$

C	2.19676835553207	0.10736794557138	-10.91167253534185
C	3.64564495866592	-0.47598427441305	-9.10591531881276
C	1.08214462251837	0.03023993698709	-10.07355062067048
C	2.56014787692072	-0.56945711837540	-8.23663983676895
C	1.27223103925464	-0.29622536335367	-8.72454072582817
H	0.08192488135603	0.20833431402672	-10.47063768716386
H	2.70868413001385	-0.85953173274028	-7.19588550742224
H	0.41313012897743	-0.36400734281834	-8.05537046408567
N	3.44061594366822	-0.09266286597878	-10.39186344919438
C	2.25167220288604	0.30338227946339	-12.35254568075190
N	3.46833674902889	0.16381704229584	-12.81911215942199
C	1.04902195245645	0.55976192180840	-13.20128122191031
H	1.27807650293408	1.27789196409411	-14.00205860549320
H	0.70837712354794	-0.37223570806810	-13.68524465114896
H	0.21972575976844	0.95797201479186	-12.60212452762627
C	3.83408789248474	0.12830877404536	-14.22387914879261
H	3.65790060050968	-0.89260675939815	-14.60304430778197
H	3.23312055912481	0.81938478602940	-14.83229861853125
C	5.30926916838116	0.49193899879918	-14.30481248913114
H	5.72272516516986	0.24866867873891	-15.29677888930142
H	5.41575382166152	1.57192888137283	-14.14365857558586
N	6.08090263205645	-0.20774425408408	-13.23961257584229
C	7.44569508306699	0.33909463950068	-13.11002901499598
H	8.03823448037573	-0.38462348455049	-12.53707998990763
H	7.91771400688453	0.42326947477660	-14.10433427177679
C	7.45541628863774	1.70551222039272	-12.43196777827345
H	8.51161569676125	2.01499605763851	-12.30522879811953
H	6.98542527652409	2.45163909433403	-13.08791215593886
N	6.72647935424285	1.70283675400960	-11.16883368212029
C	7.44502407946681	1.15497574329371	-10.01787084549795
H	8.53020531824411	1.36597464340608	-10.07124698573618

H	7.05130427554966	1.64459750333433	-9.11515337421686
C	7.23150819319645	-0.36344218411859	-9.85570932962610
H	7.71667659521943	-0.92283947907606	-10.66408851789771
H	7.67659544574675	-0.69836916591979	-8.90854127334610
N	5.80292477394974	-0.58054951453550	-9.92092698601709
C	5.06537333530292	-0.69521384923042	-8.84782619099240
C	5.59567329258189	-0.93739608302028	-7.46622505389677
H	5.99928249549567	-0.00766034122088	-7.03019645726857
H	4.80300223860068	-1.30186291100329	-6.80062952922742
H	6.40561602235655	-1.68026121050601	-7.47450317282045
Co	4.82074851343295	-0.30045336631845	-11.58589713281771
H	6.39214670877735	2.63220599443521	-10.95836537436915
H	6.16051857524536	-1.18548745802141	-13.50688173839074
C	4.46831335374184	-2.20385254922174	-11.79330596994855
O	3.80142651379235	-2.90787946141621	-11.06585370249326
O	5.05447040007995	-2.74310440280312	-12.90217260798500
H	4.82146296280906	-3.69218198495385	-12.94628839171186

y) $^{2}[Co(L)(CO)]^{2+}$

C	2.18601823668700	-0.08002664622206	-10.90826447569065
C	3.58457763978672	-0.72582314252579	-9.12035428382910
C	1.06823689163406	-0.04835148115633	-10.07170099804191
C	2.50913955274064	-0.71107501235160	-8.23060807899220
C	1.24294990088293	-0.36990707296630	-8.72146206255193
H	0.08700566170951	0.21644708927882	-10.46561444212598
H	2.65304639535434	-0.96377446241701	-7.18033430916749
H	0.38586791123557	-0.35808763885518	-8.04650743835541
N	3.38697641828983	-0.40905167986209	-10.40637157768789
C	2.24290518432711	0.13336201170076	-12.37498743632512
N	3.42613722864531	-0.05526588722775	-12.85225745435623
C	1.01945442881326	0.43304077977381	-13.16875686744833
H	1.26258990587057	0.93268454659067	-14.11486573402071

H	0.49612799714625	-0.51034981236137	-13.40400573945951
H	0.32843916531719	1.06241360138278	-12.59237115817879
C	3.78335511061529	-0.12691077440851	-14.26052670919853
H	3.57765029059867	-1.15666567288684	-14.60017425287121
H	3.18649708283274	0.55416236674687	-14.88277131191101
C	5.26214889960097	0.20192178478457	-14.36444600637282
H	5.65758776988243	-0.06169333879592	-15.35662756585232
H	5.39204148423328	1.28072996519710	-14.21995657438782
N	6.04139325765208	-0.49808556172242	-13.29751059322932
C	7.37194838508291	0.12760150930789	-13.11558146268102
H	7.99320151137476	-0.57399871379403	-12.54516505932763
H	7.86000539932178	0.27852616383573	-14.09257652408438
C	7.24606667375312	1.45985159511217	-12.38935640555217
H	8.26099354793271	1.85814101906749	-12.20775704964988
H	6.72803063016470	2.19332283832730	-13.02094942419014
N	6.48592105223087	1.31594479539210	-11.14683399596208
C	7.28215144924072	0.85497817891266	-9.99660945152788
H	8.33998284897705	1.15866535010171	-10.08553377530752
H	6.87265951860576	1.32468486933388	-9.09110793537653
C	7.18843629670892	-0.67114643087439	-9.82200925138359
H	7.69075605750826	-1.20574367997695	-10.63634581965786
H	7.64859628889067	-0.97694182973945	-8.87351103965194
N	5.76526352152730	-0.93676687763114	-9.88834413034823
C	5.02210336972508	-0.96633886891518	-8.83467807265969
C	5.50781100154738	-1.08832828806466	-7.42753080509084
H	5.71760512735801	-0.08694355193974	-7.01316290295400
H	4.74367317027570	-1.55740096736250	-6.79408884507067
H	6.42843832681819	-1.68158794463389	-7.36989061454953
Co	4.81017848820398	-0.58479400711242	-11.62689169706380
H	6.04114670568948	2.19479456026199	-10.91833892409910
H	6.20072057850867	-1.45316130077660	-13.60545619565188
C	4.45816357578048	-2.58011604445010	-11.83779013086199
O	4.17778006591774	-3.66487433707807	-11.71449823824335

z) [1]**8**$^-$

Mn	-0.59895777328590	9.11698249572691	7.59589084454266
C	-1.95615584797304	11.32859926156019	11.00847175247277
C	-2.57691979402394	11.11841647891011	6.88119734719822
C	-3.35095833393588	11.96696246161547	6.03395503903745
C	-3.29848828604625	11.81957428958679	4.66910816684499
C	-2.46640349889175	10.79683214945034	4.12202639731368
C	-1.74175344800858	10.00157559976896	4.97342551323587
N	-1.76253557030803	10.13008396867604	6.33227761136069
H	-3.97985142516865	12.73563372394177	6.48606464515572
H	-3.88598121866423	12.46629556668176	4.01451050952062
H	-2.40203524709879	10.63064588314196	3.04550063938416
H	-1.08902246327150	9.21635952666072	4.59041808803298
C	-2.47207874486825	11.21621482091214	8.28684821184000
C	-3.15285751400308	12.16858788453246	9.10054190806422
C	-2.90376139168011	12.24582375982726	10.44770591199443
C	-1.34262357063435	10.40859060793036	10.20354159682654
N	-1.54896408986882	10.30687298070468	8.84317555172629
H	-3.87647103791622	12.83732308541491	8.63058393326483
H	-3.42477830932838	12.96729617212248	11.07929241282143
H	-1.72574241001734	11.33957591735541	12.07559533857330
H	-0.63253000867319	9.69659507847401	10.62307873131581
C	0.20935418110532	8.18008346446480	8.91190415181258
C	0.91587260010006	9.69933702025560	6.96915449360190
O	0.75224414863039	7.58088238707174	9.73420710710995
O	1.88376145307338	10.24313989898231	6.58496522127445
C	-0.70076414373044	7.59077204725943	6.62345139872880
O	-0.75880314427141	6.62605138717828	5.99616605814212
K	0.07432808275916	12.37378858479210	7.43315945980286

aa) [1][Mn(bpy)$(CO)_3(CO_2)$]K

Mn	-0.11694382018828	9.66839919664885	7.59168135684987
C	-2.19473571812539	11.28095789113674	11.07905556685099
C	-2.22509783415723	11.57898154322267	6.88004695441306
C	-3.08026343128695	12.37986040883773	6.10515073077455
C	-3.05922432718150	12.27008009721702	4.72184950936217
C	-2.17739727043761	11.34996943430886	4.13774167844074
C	-1.35499568263576	10.59627119480604	4.96202782700328
N	-1.36562311257217	10.70244911847339	6.30124574193817
H	-3.76625216164658	13.07273522063631	6.59134656895613
H	-3.72455434153785	12.87794381632751	4.10529533785775
H	-2.13204600335081	11.20956508118964	3.05640997588796
H	-0.65651769248502	9.87011559913341	4.54623752053477
C	-2.22889867494391	11.56332155970759	8.34227239209953
C	-3.06312656626513	12.37350383753952	9.13037164884058
C	-3.04726996851604	12.23840213807725	10.51140163603613
C	-1.39123150430009	10.52050288794545	10.24234881406784
N	-1.39298752823843	10.65390736180825	8.90500086072406
H	-3.72395664969987	13.09871795472489	8.65659287659703
H	-3.69252879135791	12.85688820853359	11.13876810701714
H	-2.15547591317899	11.11927372740220	12.15779770804646
H	-0.71284076720919	9.77029519535305	10.64822408374314
C	1.01170903442066	9.11553548196249	8.88711094327023
C	1.09145690540423	9.20023772589445	6.33415170858504
O	1.72541423700704	8.78554245942954	9.72840072960144
O	1.86281038636777	8.91148670040281	5.52952091929332
C	-1.02903001977615	8.10985729840078	7.48003714308931
O	-1.58664061550521	7.10524123711590	7.38487151254391
C	1.08966810676142	11.69817973698194	7.66164346095219
O	0.38283270808583	12.73537197747462	7.65813508424674
O	2.33898923359082	11.65547808311839	7.68619761233168
K	2.46843492795836	14.16270856518879	7.69019826804467

bb) ^{1}[Mn(bpy)(CO)$_3$(CO$_2$H)]K$^+$

Mn	-0.55293063428358	11.22630233186263	7.99305529826711
C	-2.40168354484023	14.90987169617508	9.38952765551526
C	-1.68246216548926	12.81377466790651	5.80196706872301
C	-2.13586698849176	13.16081800805517	4.52269094187193
C	-1.80747634714932	12.35420763577221	3.43612797350518
C	-1.02337387409440	11.21850261196451	3.65564812284714
C	-0.61028751226942	10.93206353299654	4.95243932504466
N	-0.92904553196458	11.70343985207527	6.00525832884951
H	-2.74947222357343	14.04928853826008	4.37707245013909
H	-2.16276433316109	12.60942973076450	2.43544465118497
H	-0.73771647934377	10.55269892572494	2.83976045121331
H	-0.00610678969004	10.05000166044447	5.16378943004729
C	-1.96894480213869	13.59467508262158	7.01694333149495
C	-2.67099289631000	14.80489396140682	7.01037163690642
C	-2.90045216410193	15.46953768640266	8.21226914560072
C	-1.70100868613485	13.70888565575088	9.31928519468658
N	-1.49489092345543	13.05583683104562	8.16676391225229
H	-3.04012438892298	15.22516183879099	6.07538977093091
H	-3.45483826123142	16.40980489060956	8.22739332416523
H	-2.55495592651711	15.39236706613916	10.35614129619417
H	-1.28973482204669	13.24912334704053	10.21765836515349
C	-0.30126641456353	10.98884854636822	9.76779635750463
C	0.42749404910687	9.76379047574768	7.62348877591258
O	-0.14224456472570	10.85875112768803	10.89175049917207
O	1.11124939003591	8.88343044655796	7.35384734615857
C	-2.14452982838394	10.28745191842844	8.08830711431781
O	-3.10589874759648	9.67878467853801	8.18096746297589
C	1.24777750431287	12.19298449206218	7.99928267304746
O	1.59112352864203	13.03883143484314	6.92729705613739
O	2.13912996495546	12.12199079781680	8.82873000794138
H	0.87352197706465	13.02308655927278	6.27874723215809

K 4.18515393336193 13.15688233086604 7.73098545508036

cc) ^{2}[Mn(bpy)(CO)$_3$(CO)]

Mn	-0.62447234670752	10.71899442596471	8.76773840142581
C	-2.07178125653398	14.44180946353124	10.49788658002227
C	-1.92713795533123	12.43316347544064	6.79913828528436
C	-2.46865836618486	12.85364921211598	5.54991150083222
C	-2.31206926391259	12.08216367635299	4.42282883417385
C	-1.59812706617272	10.85677742950955	4.51568421817229
C	-1.09956538915425	10.48789829895135	5.75150375343368
N	-1.24807223791009	11.22368051664797	6.86547935448386
H	-3.00886715169399	13.79916387782213	5.49488828663615
H	-2.73312534505882	12.41257212639805	3.47022150073775
H	-1.43830775107535	10.21271390318816	3.65062868017622
H	-0.55189488375478	9.55115104193920	5.86349185176851
C	-2.01970907320330	13.16426084421910	8.01324719095044
C	-2.68659076919395	14.41396843121723	8.16582527107986
C	-2.72124882248600	15.04438459171029	9.38662508010529
C	-1.43586260105731	13.23086560293480	10.30062327847561
N	-1.40432743836172	12.59159819412977	9.11973744515802
H	-3.17672170302142	14.86504469803604	7.30258170378248
H	-3.24182242804087	15.99787756014808	9.49994636575048
H	-2.06749607934219	14.90780879956149	11.48325818655962
H	-0.92190296382037	12.73867880040487	11.12757354001001
C	-0.14142553686428	10.41852870502612	10.50169767589042
C	-0.01114240542454	9.07113952978932	8.28796368465364
O	0.16720043343810	10.22132147702313	11.58618248253285
O	0.37430948905223	8.03441353664060	7.99348027565182
C	-2.37450587308409	10.08537149010652	9.07159553627880
O	-3.44360318366942	9.74700089628582	9.25088670914100
C	1.02123628490877	11.54293870327668	8.38846397904285
O	2.00188256166062	12.06879319062773	8.15840721678962

dd) **^{1}9^{2-}**

Cr	-0.54202718384952	9.12902128639803	7.58616775093086
C	-2.02338452515632	11.32979385308651	11.03035467191908
C	-2.54248855031244	11.21875902660498	6.87870146933466
C	-3.36663179379173	12.03524577721002	6.03919995907713
C	-3.41388692463396	11.83295034230055	4.68339900966748
C	-2.61209124009286	10.77949941132785	4.12184187151205
C	-1.82966287852596	10.02492755763732	4.96299415293500
N	-1.75593240985568	10.18893307270400	6.31496575247987
H	-3.95762353440466	12.83178526749429	6.49807836353697
H	-4.04024630053944	12.46038239085554	4.04381264223449
H	-2.61886843299695	10.56955303932105	3.04960755601071
H	-1.20133674142348	9.22447018268913	4.56474858372432
C	-2.42419815391688	11.32613889886835	8.26901321759146
C	-3.13494226566676	12.24915992200658	9.09899992134844
C	-2.95541392535557	12.26945010808552	10.45821252962446
C	-1.36791172552306	10.45543561608477	10.20540011149248
N	-1.51880649222669	10.39157274260454	8.84213806687344
H	-3.83283445464912	12.94491357189890	8.62630763741605
H	-3.50211319772270	12.97232791011232	11.09142935886954
H	-1.83091539633695	11.30220981449722	12.10560061173584
H	-0.66247977992503	9.73490134464829	10.62287091958791
C	0.19378589096586	8.18657602925729	8.94084977499185
C	1.10116032697292	9.50908580306522	6.98781661926519
O	0.69523090379873	7.55163836224371	9.81215893465995
O	2.20734277508026	9.71600345652875	6.59812322359632
C	-0.51461161495553	7.62102093812372	6.59177724236094
O	-0.45700212595644	6.61690949034532	5.95715931222326

ee) $^1[Cr(bpy)(CO)_3(CO_2)]^{2-}$

Cr	-0.62938215537878	9.02642833605819	7.65630050254483
C	-1.73381617355080	11.58959664933111	11.00393920829769
C	-2.56678554460865	11.10404951403240	6.91657151630996
C	-3.46521057985723	11.86214729924221	6.13693058950274
C	-3.57442002449419	11.62520686554943	4.77713425253290
C	-2.75726062676822	10.62331329960964	4.20927063084314
C	-1.88212486808316	9.92976100689818	5.02445895984979
N	-1.76995279555009	10.14118120861364	6.35648774796793
H	-4.07112208058769	12.63459484597119	6.61185785963429
H	-4.26587774116022	12.20352941277090	4.16089173612131
H	-2.80369053353428	10.38726733382757	3.14395386053885
H	-1.23317233150856	9.15589795413171	4.61423129204419
C	-2.36434944396079	11.29908046252835	8.33532026775567
C	-3.04109990462385	12.26656905588930	9.10803711496312
C	-2.73379051398350	12.41711494423987	10.44996087356821
C	-1.11501483691252	10.65472007863373	10.19203404817842
N	-1.41214128751111	10.48862767814668	8.88592080408926
H	-3.80174656288977	12.89476534834284	8.64329553106081
H	-3.25032820100690	13.15970473894025	11.06182655124898
H	-1.44052994440532	11.67544721607242	12.05234679032469
H	-0.34609470072407	9.98960729046655	10.58671069478611
C	-0.08886441916468	7.96491507533677	9.02060131894003
C	1.05893858562687	9.66986791003476	7.51638403831097
O	0.26061758619931	7.26635914896388	9.90636474381596
O	2.20889153761749	9.96323153189500	7.46570515988721
C	-0.20179545182115	7.75295182103508	6.43700820516691
O	0.10177269738670	6.92645146386543	5.64876084768245
C	-2.56878175420737	7.87688875041688	7.97033626810795
O	-3.55909329665552	8.61468734389538	8.14872135239427
O	-2.59342678688183	6.62413476726012	7.93915218653103

ff) $^{1}[Cr(bpy)(CO)_3(CO_2H)]^-$

Cr	-0.49627559762669	8.89684899133991	8.81925497473563
C	-1.32645680614893	9.60186429505423	13.06361507387571
C	-2.16048018813157	11.30358769319404	9.29832996590068
C	-2.98516870631270	12.41101208119361	9.05034454127313
C	-3.12830237755300	12.88062977152416	7.74873342835004
C	-2.42163092429172	12.23698643943327	6.72592836336446
C	-1.61195777341722	11.15359725281406	7.05072409691052
N	-1.48646585139726	10.68398437748700	8.30166520384733
H	-3.51301841209596	12.89675168364278	9.87055419152733
H	-3.77176296714133	13.73622365931933	7.53345642928253
H	-2.49338985722901	12.57214440884796	5.68951077929972
H	-1.04540347995218	10.62629388436848	6.28333961572842
C	-1.93621275391245	10.74230867613139	10.63696711724164
C	-2.47211025181383	11.29624497378270	11.80933062484026
C	-2.16698090141529	10.72210197131796	13.03954019719817
C	-0.83881454258650	9.10511998866474	11.86060589478496
N	-1.13346491614002	9.65267010820254	10.66990249549367
H	-3.11356500869318	12.17559291394004	11.75732848039404
H	-2.57370305541192	11.14385826133384	13.96121870435550
H	-1.04655399385682	9.11305524521055	13.99886453276746
H	-0.19040823194476	8.22974027831025	11.83762439315938
C	0.19989951853386	7.36304146614767	9.50059284901899
C	1.19069023008707	9.60230886804794	8.95906074006719
O	0.70461727868855	6.40053738759169	9.95907102866382
O	2.31044317501203	9.95728096347192	9.03164413298859
C	-0.10934113952873	8.38449843007018	7.11462368674694
O	0.17257255525326	8.06918899145065	6.01453274208941
C	-2.47258308298162	8.09987965200609	8.67754896664776
O	-3.52636173762433	8.63575511565844	8.98151599722661
O	-2.63651187343846	6.81785242363201	8.16483630782684
H	-1.74354996192918	6.50019906780994	7.96081677039274

gg) $^{2}[Cr(bpy)(CO)_4]^{-}$

Cr	-0.58413102878378	8.09876190562792	8.30119528567082
C	-0.06020689281358	9.82456392654444	12.34131539756681
C	-1.03924971020876	11.05870144712152	8.42845032263220
C	-1.34702017410004	12.38358880483461	7.98995011290202
C	-1.64710483694915	12.63158861616981	6.66897935164528
C	-1.65233080173233	11.55093674747545	5.74640107279528
C	-1.35143068296877	10.28277117091819	6.23091941919299
N	-1.05005132775439	10.01828942127513	7.50667366227522
H	-1.34042601804101	13.20209644895994	8.71133718270629
H	-1.88550347649548	13.64547294964774	6.33585317079480
H	-1.89668897493973	11.69543127072076	4.69299162874500
H	-1.35219003431898	9.42275934430014	5.55801314012968
C	-0.71530333698011	10.70826958275158	9.76890954594501
C	-0.69381117885825	11.62544771126398	10.86400101721672
C	-0.37660341660502	11.19408158305937	12.13320350425347
C	-0.09451338806915	8.97973236759822	11.23827272741305
N	-0.41068728090665	9.37260771388924	9.99939842517843
H	-0.94699678436634	12.67253065990922	10.68990874147700
H	-0.37175159016408	11.89943103728709	12.96823713855249
H	0.21013670470659	9.43238509903294	13.32296389970975
H	0.14474118607370	7.92050015661450	11.35187825764035
C	-0.16264128232353	6.55382716334033	9.18969607839600
C	1.23256406687718	8.37404636973592	7.82985660913517
O	0.08337534118424	5.55549653520927	9.75220442713967
O	2.33762928475392	8.49737602876867	7.50714817940896
C	-0.83295390127993	7.15158588496621	6.75214738513496
O	-0.98974976018794	6.51426350725005	5.78135319833175
C	-2.39137538408862	7.76957812407026	8.75464888105269
O	-3.47227630465998	7.47620971465710	9.03114345795777

hh) ^{1}**10**^{0}

Ru	-0.305535	0.486769	-1.002611
C	-0.469505	2.654643	-5.394689
C	-0.646905	4.479424	0.872097
C	-0.590637	3.114699	0.644701
N	-0.537477	2.633091	-0.574384
C	-0.536615	3.395523	-1.709763
C	-0.588276	4.809340	-1.526056
C	-0.641199	5.343674	-0.258833
H	-0.588060	2.400448	1.470466
H	-0.584009	5.462929	-2.399183
H	-0.680080	6.426933	-0.121948
H	-0.695379	4.867435	1.890160
C	-0.490404	2.730375	-2.962921
N	-0.430969	1.334122	-2.921802
C	-0.417329	0.640684	-4.077800
C	-0.434662	1.232549	-5.326348
C	-0.500521	3.384759	-4.229300
H	-0.421145	0.613050	-6.223610
H	-0.477008	3.163588	-6.361578
H	-0.537343	4.474202	-4.266129
H	-0.407998	-0.443828	-3.975382
C	-0.352983	-1.384700	3.581314
C	0.861366	-0.516646	1.690757
N	-0.341282	-0.326013	1.038061
C	-1.540844	-0.556678	1.641199
C	-1.551717	-1.118851	2.934402
C	0.883780	-1.075555	2.958333
H	-2.501405	-1.341954	3.423620
H	-0.358394	-1.825755	4.579549
H	1.825678	-1.266384	3.471868
C	-2.654909	-0.200753	0.819533
N	-2.324695	0.389552	-0.398510

C	-3.291831	0.808094	-1.231467
C	-4.637476	0.660190	-0.946777
C	-5.005390	0.041579	0.277761
C	-4.020801	-0.380555	1.147885
H	-2.947814	1.273208	-2.156667
H	-5.384648	1.022328	-1.653660
H	-6.057799	-0.100181	0.534855
H	-4.283158	-0.858223	2.093209
C	-0.206550	-1.474907	-1.752915
O	-0.291112	-2.576330	-2.259382
H	1.789068	-0.263636	1.199070

ii) $^{1}[Ru(bpy)_2(CO)(CO_2)]$

Ru	-0.159556	0.493225	-1.020999
C	-0.323526	2.661099	-5.413078
C	-0.500926	4.485880	0.853709
C	-0.444658	3.121155	0.626313
N	-0.391498	2.639547	-0.592773
C	-0.390636	3.401980	-1.728151
C	-0.442297	4.815796	-1.544445
C	-0.495220	5.350131	-0.277221
H	-0.442081	2.406905	1.452078
H	-0.438030	5.469385	-2.417571
H	-0.534101	6.433390	-0.140336
H	-0.549400	4.873892	1.871772
C	-0.344425	2.736831	-2.981309
N	-0.284989	1.340579	-2.940190
C	-0.271350	0.647140	-4.096188
C	-0.288683	1.239006	-5.344737
C	-0.354542	3.391216	-4.247689
H	-0.275166	0.619506	-6.241998
H	-0.331029	3.170045	-6.379966

H	-0.391364	4.480659	-4.284517
H	-0.262019	-0.437371	-3.993770
C	-0.207004	-1.378244	3.562926
C	1.007345	-0.510189	1.672369
N	-0.195303	-0.319556	1.019673
C	-1.394865	-0.550222	1.622811
C	-1.405738	-1.112395	2.916014
C	1.029759	-1.069099	2.939945
H	-2.355426	-1.335498	3.405231
H	-0.212415	-1.819298	4.561160
H	1.971657	-1.259927	3.453479
C	-2.508930	-0.194297	0.801145
N	-2.178716	0.396009	-0.416898
C	-3.145852	0.814550	-1.249855
C	-4.491497	0.666647	-0.965165
C	-4.859411	0.048036	0.259373
C	-3.874822	-0.374099	1.129497
H	-2.801835	1.279664	-2.175055
H	-5.238669	1.028785	-1.672048
H	-5.911820	-0.093725	0.516466
H	-4.137179	-0.851766	2.074820
C	-0.060571	-1.468451	-1.771303
O	-0.145133	-2.569874	-2.277770
H	1.935047	-0.257180	1.180682
C	1.823530	0.439865	-1.275006
O	2.547224	0.785033	-0.201355
O	2.354738	0.339230	-2.501094

jj) $^{1}[Ru(bpy)_2(CO)(CO_2H)]^{+}$

Ru	-0.13659623131226	0.38996296844432	-1.10344027358489
C	-0.43792786594914	2.68606056972834	-5.38131802664696
C	-0.38648201348228	4.32040700824230	0.92119152372274

C	-0.30745716974781	2.95541125115191	0.66183869905279
N	-0.28908670531862	2.46315176105446	-0.58297795792878
C	-0.37714606497464	3.30092162920709	-1.64368185720308
C	-0.45508728362008	4.68397410480164	-1.45537444916031
C	-0.45314272356039	5.19993153859338	-0.16017546432019
H	-0.24964895174348	2.23267673531195	1.47547248473480
H	-0.51697487900250	5.35399221219901	-2.31147200804122
H	-0.50844818437421	6.27790167368895	0.00189699151887
H	-0.39143857948910	4.67801870754315	1.95157663606777
C	-0.38458290655678	2.65412224316971	-2.96854519728454
N	-0.32717090400579	1.29560257161137	-2.96767012685956
C	-0.32525048597270	0.63848443007520	-4.13684022744281
C	-0.38257925477343	1.29245422694338	-5.36393159474307
C	-0.44090547589797	3.37017044318866	-4.16717228741102
H	-0.37805036237740	0.70654479323872	-6.28398295539955
H	-0.47755827075588	3.23656314669350	-6.32287980831245
H	-0.48293481145856	4.45828906342757	-4.15228936852323
H	-0.27264082793402	-0.44687862816971	-4.08491904267226
C	-0.26560347832731	-1.11199551717817	3.53614402797206
C	1.00202090390461	-0.55100652045869	1.58075566302320
N	-0.11256353475981	-0.26240694732573	0.89188605100050
C	-1.32642232321132	-0.40169896901298	1.48921146237952
C	-1.42370519835503	-0.82396712461190	2.81794136625208
C	0.97074012501167	-0.97577231518775	2.90502333148843
H	-2.39917552111672	-0.93439252127511	3.28857266542118
H	-0.33247724459014	-1.45107362042775	4.57133702470352
H	1.90530633002740	-1.20086319482810	3.42006769056290
C	-2.50735008198991	-0.09200935206938	0.65776522269252
N	-2.24606648835958	0.27252480904198	-0.61605652854038
C	-3.24589527608494	0.56592128800473	-1.45046620416996
C	-4.58067270675946	0.51002405436950	-1.05529773409139
C	-4.87189934399786	0.13577244071504	0.25742506952899
C	-3.82392871528773	-0.16714734265425	1.12547060502400

H	-2.96403190625826	0.85636499829680	-2.46330687160766
H	-5.36833842295709	0.75702256748031	-1.76795631483154
H	-5.90443746965419	0.07792421676401	0.60791730717085
H	-4.03390302948982	-0.46755265084157	2.15111397302114
C	0.01665029104173	-1.36023597961000	-1.68291974381658
O	0.14050802820202	-2.45390870549065	-2.03607982322013
H	1.94451577949027	-0.42719414214736	1.05160180073310
C	1.87455746511030	0.60416132790716	-1.43278887389469
O	2.59333636858182	1.41345205998803	-0.57543268323728
O	2.52109435004837	0.12912746108218	-2.34547002092127
H	2.00473464008808	1.78142278132467	0.09994407079388

kk) $^{1}[Ru(bpy)_2(CO)_2]^{2+}$

Ru	0.26030407497020	1.14393210947526	-0.65102191488265
C	-0.22088898203993	3.50035235263254	-4.88074602123644
C	2.14688392480079	4.73017599136193	1.06065470866910
C	1.71301378504006	3.42363219008036	0.85337809678070
N	1.17043385223638	3.03203729828416	-0.30490005865038
C	1.05297160346291	3.90674670220477	-1.33405062852374
C	1.47736837520916	5.22976680593024	-1.19460447172177
C	2.02219151203580	5.64894013838349	0.01892741323547
H	1.80172607338769	2.67325891250509	1.63654330427784
H	1.37109389404030	5.92988240838275	-2.02198058327826
H	2.34975285253367	6.68222936521342	0.14640394428155
H	2.57599008097173	5.01115487398165	2.02296623482088
C	0.44734732018764	3.37025953997197	-2.56635331007257
N	-0.03590290255018	2.10340462743241	-2.48890542970296
C	-0.60651915850386	1.53670701543227	-3.56222541226957
C	-0.72193571632920	2.20283072185229	-4.77747964205970
C	0.36485685308729	4.08899779955137	-3.76100508536299
H	-1.19429224597072	1.69840535912780	-5.62100223213615
H	-0.28156073245651	4.05116456658923	-5.82081888731457

H	0.76739543537672	5.09939230588021	-3.81890942950565
H	-0.97757439880286	0.52032043591207	-3.44600290684329
C	0.78212989530425	0.01800878575927	4.04833885148649
C	1.54974705207105	-0.18416608309003	1.78721290542149
N	0.51690863615483	0.54242651864615	1.33647491680967
C	-0.41801108976612	1.00953068836473	2.20572975951716
C	-0.29890196242241	0.76196093165662	3.57490229961817
C	1.71964608544680	-0.46954489468342	3.13833174169642
H	-1.04005706900816	1.15716168748081	4.26787451999848
H	0.88980212460010	-0.17960683502891	5.11635759334108
H	2.57755416380073	-1.06117826356063	3.45953236881690
C	-1.52178632836172	1.78795952723457	1.61243694668866
N	-1.43486262452064	2.01099496155221	0.27807049487119
C	-2.37926988772791	2.71331263333124	-0.35609803829058
C	-3.48046083456404	3.23905150780911	0.31434909333263
C	-3.59606530611084	3.01379752645260	1.68642071644260
C	-2.60741451959316	2.27879119576157	2.34143962437961
H	-2.25163195539226	2.85398687022840	-1.42828168946618
H	-4.22852771234566	3.80539876329778	-0.24094715472746
H	-4.44803556089772	3.40281991952196	2.24674138110825
H	-2.68673117868144	2.08171797906550	3.40977940928059
C	-0.75872464988734	-0.41348780954880	-1.01706968683328
O	-1.42323168790941	-1.31756296607349	-1.22573792002073
H	2.26130447605825	-0.54288377436318	1.04634283684142
C	1.82639692273245	0.34656081265369	-1.37984915781610
O	2.74506205633331	-0.14038653065316	-1.83996328700134

ll) **^{1}11^{0}**

Ru	-0.30471234045047	1.06445797447218	-0.65850128523122
C	-0.10456236427474	-0.26429226545648	-5.41163970137418
C	-0.56378634323850	5.30102292869196	-2.06138009856311
C	-0.54427991143259	4.13467711070031	-1.30450628016682

N	-0.43761596772481	2.91056647952846	-1.85005955961448
C	-0.36206592055943	2.80607527404765	-3.20391366372744
C	-0.37993277126951	3.93890238274061	-4.02906990173691
C	-0.47748176909302	5.20302211215090	-3.45532616779126
H	-0.61133847574325	4.16081260413099	-0.21647653994553
H	-0.31351403256122	3.82950210601724	-5.11177033680872
H	-0.48842067192371	6.09584272393106	-4.08344623391701
H	-0.64741411232534	6.26749810249184	-1.56123871277054
C	-0.26070349815588	1.42824850561141	-3.69678732549030
N	-0.25011026347735	0.48250557738241	-2.71755708324024
C	-0.17159337249671	-0.81498135632370	-3.07303586435470
C	-0.10057073234102	-1.22987870187520	-4.39675462111091
C	-0.18389833180810	1.07730159940043	-5.05189814640746
H	-0.03969391346373	-2.29512976854318	-4.62684199091507
H	-0.04572517963305	-0.55630000587959	-6.46220886743056
H	-0.18841621011282	1.85244011900962	-5.81844927809444
H	-0.16531281745476	-1.52857108379198	-2.24832016295992
C	-0.00436364881729	-1.87545175004901	3.05242768532620
C	4.05050342191889	1.62262177200010	-0.72945242239192
C	2.70049515660637	1.72675230155720	-1.00208544302948
N	1.74367791718258	1.05516857489658	-0.31017638636717
C	2.15182085375669	0.20539642645375	0.71879918309883
C	3.52103341215944	0.06141152157931	1.02555539789967
C	4.47574999929898	0.76633991876863	0.31424426493953
H	2.33174263684030	2.37137216814272	-1.80217042210586
H	3.81588827124742	-0.60998079304328	1.83412949427310
H	5.53531075429190	0.66223697090417	0.55872083383871
H	4.76531742347607	2.19964468795014	-1.31895679348377
C	1.07060703417498	-0.44667889355414	1.40851453009717
N	-0.18737926121641	-0.07640664635329	0.89456775601767
C	-1.35076315408177	-0.64679963718034	1.44786998692780
C	-1.25699492980881	-1.52565058924975	2.52278291110159
C	1.15670181039750	-1.33085249755211	2.47906299148427

H	-2.16779535851433	-1.94617321786631	2.95459495057250
H	0.06800562173078	-2.56468858794484	3.89520962490941
H	2.13704890992068	-1.60375632440861	2.87616787580857
C	-2.54412529605219	-0.19052398858201	0.78815070260589
N	-2.31181177836837	0.71444515436773	-0.24958706938607
C	-3.38949586852853	1.21038887484403	-0.91380127751538
C	-4.69385154468154	0.87111730545224	-0.61254978151131
C	-4.94051339549902	-0.04830296963970	0.43605574394969
C	-3.86042070582975	-0.57180063200410	1.12475104453999
H	-3.15581613439876	1.90953380654630	-1.71905808140358
H	-5.51013701678357	1.31400293159866	-1.18659437641242
H	-5.95872358637619	-0.34444840814527	0.69924575990124
H	-4.01578027050608	-1.28411414792580	1.93728560096598

mm) ^{1}[Ru(tpy)(bpy)(CO_2)]

Ru	-0.35373417236473	0.51937931714113	-0.84429142074292
C	-0.59737104215342	2.71311867836779	-5.23713240768294
C	-0.72768251137231	4.57675951036168	0.99645560818663
C	-0.64062180186693	3.20367417254907	0.78633970978647
N	-0.58545544617946	2.66437389914697	-0.43736154220954
C	-0.62488022777133	3.46587457815000	-1.52499809622186
C	-0.70874970571293	4.85888191907951	-1.39201408174367
C	-0.75802221899024	5.42075234717834	-0.11849711336404
H	-0.60743969463072	2.50409811802180	1.62373605719131
H	-0.73652164069228	5.49864457624926	-2.27347640051438
H	-0.82291318700789	6.50373615057023	0.00393754905339
H	-0.76902888192361	4.97398155182910	2.01215466343654
C	-0.58279399458973	2.76663808538229	-2.82140345270363
N	-0.51042723801386	1.40799892727417	-2.76016454769184
C	-0.50611933938799	0.70866380403534	-3.91181380109530
C	-0.54407904638419	1.31870907979093	-5.16129807112499
C	-0.62420046437114	3.43891673654825	-4.04936994361713

H	-0.53297637141815	0.69998392878804	-6.06040382212256
H	-0.62515139096290	3.22750962374321	-6.19987143962666
H	-0.68127738765800	4.52665914428785	-4.07495322919918
H	-0.49961276449491	-0.37431593661918	-3.77627295053859
C	0.00951279583977	-1.16837351215796	3.52379065373583
C	3.95607445812563	1.19472908993706	-1.12024061529225
C	2.59550142772688	1.17533552119367	-1.41043720683722
N	1.68245451862248	0.65854137055513	-0.57418449793227
C	2.09858480239520	0.13684582585582	0.62240423799571
C	3.45357154613225	0.12000282696425	0.96835326542312
C	4.39691714930430	0.64829750413059	0.09072966584038
H	2.20905205832999	1.58043999399725	-2.34623192222466
H	3.76524393831059	-0.30611008982621	1.92266299744463
H	5.45661043281899	0.64176317974622	0.35423747347343
H	4.65379976196954	1.62694236405451	-1.83898804561768
C	1.02413735067097	-0.32819396861160	1.50462919386318
N	-0.21143553678446	-0.17291681260285	0.96359420248139
C	-1.34122511432136	-0.50451379050921	1.64308597440799
C	-1.25344718826986	-1.00031275115212	2.94442876924232
C	1.15727254291063	-0.83492553282917	2.79981943254237
H	-2.15441194042063	-1.26863442413386	3.49720888984364
H	0.09841642993704	-1.56748652645442	4.53548138450280
H	2.14400050411532	-0.96680353908195	3.24457003782727
C	-2.55817455210686	-0.31642450335078	0.84333567802217
N	-2.33658483598076	0.11974206113623	-0.43650902477291
C	-3.38028829948518	0.26084243977937	-1.26647913455872
C	-4.69130304830101	0.00957346159068	-0.87113042706296
C	-4.93624543791475	-0.40613099782080	0.44188467488035
C	-3.85326710171537	-0.57231975757681	1.30345499187822
H	-3.14081169063700	0.59732175126642	-2.27533892291796
H	-5.50499833742116	0.14658563276310	-1.58507540805040
H	-5.95181176261338	-0.60617116499228	0.78987732875806
H	-4.00938393422680	-0.91253344624725	2.32792508811717

C	-0.11727442168795	-1.42616860478557	-1.54105681107172
O	0.80585877911508	-2.12109786263885	-1.04410971946281
O	-0.90384241949144	-1.83975841307444	-2.44213616093355

nn) $^{1}[Ru(tpy)(bpy)(CO)]^{2+}$

Ru	-0.29839446069356	0.28095704176294	-0.99557724668042
C	-0.58241584003453	2.68764357927227	-5.26556698400530
C	-0.68055209810029	4.16524316467903	1.09157718107262
C	-0.57897179516912	2.81511236838107	0.79820060374022
N	-0.53234156099542	2.32899452087578	-0.44923117979610
C	-0.59461081184760	3.19445825428731	-1.53563119522600
C	-0.69194239299040	4.59644089687568	-1.28792705578917
C	-0.73296297580500	5.07772738716730	0.00162730616329
H	-0.52647805019489	2.07424241378428	1.59747355155224
H	-0.73541868832285	5.28752561942082	-2.13055865792541
H	-0.80800569635489	6.15244486187165	0.18551622544194
H	-0.71518177429069	4.50393042364955	2.12787846474720
C	-0.55749734527333	2.60124514059529	-2.82971749807111
N	-0.45878238503764	1.21157480439264	-2.88166033981614
C	-0.43450100427573	0.60115999800098	-4.07833924047477
C	-0.49421154972365	1.26971611306930	-5.29103350809432
C	-0.61407688543070	3.33587470077578	-4.05143133690456
H	-0.46817782103782	0.70841802964096	-6.22591294324469
H	-0.62637109730867	3.25989987054575	-6.19551355637848
H	-0.68601536949626	4.42363889208658	-4.01504462662395
H	-0.35873375952396	-0.48707986748078	-4.05310068947140
C	0.04593263549013	-1.03996962813476	3.52820135429452
C	4.00234953210137	1.14487899734632	-1.22509125359399
C	2.65719649171199	1.02355968028742	-1.53888466416649
N	1.74705608960106	0.53313195387444	-0.68539695389567
C	2.13983386660490	0.13902990033338	0.59439156649888
C	3.50936209976314	0.21699879380283	0.94934587437524

C	4.43568926301189	0.71355485273455	0.05385517521345
H	2.27067441532856	1.32814150786152	-2.51337837704594
H	3.82297506607750	-0.11950898042320	1.93898647951756
H	5.48905567948140	0.77876069372291	0.33661122953045
H	4.69710490567510	1.55694803351240	-1.95775144243950
C	1.09045444978033	-0.29754418538580	1.47295514378699
N	-0.16737918466249	-0.26956406504488	0.91281506524507
C	-1.30304154768942	-0.57124125459051	1.58788166523431
C	-1.23265380747663	-0.96340562046023	2.91656577105198
C	1.19327983282118	-0.70832128817781	2.81730533685542
H	-2.13197964795150	-1.21188230491194	3.48008348889916
H	0.12757589215019	-1.36510359910541	4.56690630504586
H	2.17004330381112	-0.76341745121612	3.30079238740285
C	-2.52259944738990	-0.40942590539313	0.77770137264627
N	-2.32609657743010	-0.00348242738807	-0.51747884088841
C	-3.37870999598462	0.18357329753578	-1.32422397298644
C	-4.68783341573164	-0.02305585459169	-0.90358146953924
C	-4.91182723242123	-0.43885609324295	0.41593210795617
C	-3.81970880879903	-0.62945766519144	1.25760640725276
H	-3.14521614657883	0.51374600925304	-2.33753902027983
H	-5.51234971155885	0.14407949191823	-1.59792318633528
H	-5.92489657153700	-0.61387405634780	0.78436438227886
H	-3.96651865070738	-0.95649867192630	2.28741825122557
C	-0.08298062799110	-1.43788140323319	-1.61003641655768
O	0.06520356740685	-2.52320989307179	-1.98848932179855

9) Bibliography

[1] (a) C. Costentin, S. Drouet, G. Passard, M. Robert and J. M. Saveant, Proton-coupled electron transfer cleavage of heavy-atom bonds in electrocatalytic processes. Cleavage of a C-O bond in the catalyzed electrochemical reduction of CO_2, *J. Am. Chem. Soc.*, 2013, **135**(24), 9023-9031.

[2] D.C. Lacy, C. C. L. McCrory and J. C. Peters, Studies of cobalt-mediated electrocatalytic CO_2 reduction using a redox-active ligand, *Inorg. Chem.*, 2014, **53**(10), 4980-4988.

[3] J. Tory, B. Setterfield-Price, R. A. Dryfe and F. Hartl, [M(CO)$_4$(2, 2′-Bipyridine)] (M= Cr, Mo, W) as Efficient Catalysts for Electrochemical Reduction of CO_2 to CO at a Gold Electrode, *ChemElectroChem*, 2015, **2**(2), 213–217.

[4] M. D. Sampson, A. D. Nguyen, K. A. Grice, C. E. Moore, A. L. Rheingold and C. P. Kubiak, Manganese Catalysts with Bulky Bipyridine Ligands for the Electrocatalytic Reduction of Carbon Dioxide: Eliminating Dimerization and Altering Catalysis, *J. Am. Chem. Soc.,* 2014, **136**(14), 5460–5471.

[5] M. L. Clark, P. L. Cheung, M. Lessio, E. A. Carter and C. P. Kubiak, Kinetic and Mechanistic Effects of Bipyridine (Bpy) Substituent, Labile Ligand, and Brønsted Acid on Electrocatalytic CO_2 Reduction by Re (Bpy) Complexes, *ACS Catal.,* 2018, **8**(3), 2021–2029.

[6] L. Chen, Z. Guo, X. G. Wie, C. Gallenkamp, J. Bonin and E. Anxolabéhère-Mallart, Molecular catalysis of the electrochemical and photochemical reduction of CO_2 with earth-abundant metal complexes. Selective production of CO vs HCOOH by switching of the metal center*, J. Am. Chem. Soc.*, 2015, **137**(34), 10918-10921.

[7] H. Ishida, K. Tanaka and T. Tanaka, Electrochemical CO_2 Reduction Catalyzed by Ruthenium Complexes $[Ru (Bpy)_2(CO)_2]^{2+}$ and $[Ru(Bpy)_2(CO)Cl]^{+}$. Effect of PH on the Formation of CO and HCOO, *Organometallics* 1987, **6**(1), 181–186.

[8] Z. Chen, C. Chen, D. R. Weinberg, P. Kang, J. J. Concepcion, D. P. Harrison, M. S. Brookhart and T. J. Meyer, Electrocatalytic Reduction of CO_2 to CO by Polypyridyl Ruthenium Complexes, *Chem. Comm.,* 2011, **47**(47), 12607–12609.

[9] D. Z. Zee, M. Nippe, A. E. King, C. J. Chang and J. R. Long, Tuning Second Coordination Sphere Interactions in Polypyridyl–Iron Complexes to Achieve Selective Electrocatalytic Reduction of Carbon Dioxide to Carbon Monoxide, *Inorg. Chem.,* 2020, **59**(7), 5206–5217.

[10] C. Cometto, L. Chen, P. K. Lo, Z. Guo, K. C. Lau, E. Anxolabéhère-Mallart, C. Fave, T. C. Lau and M. Robert, Highly Selective Molecular Catalysts for the CO_2-to-CO Electrochemical Conversion at Very Low Overpotential. Contrasting Fe vs Co Quaterpyridine Complexes upon Mechanistic Studies, *ACS Catal.,* 2018, **8**(4), 3411–3417.

[11] C. Costentin, S. Drouet, M. Robert and J. M. Savéant, Turnover Numbers, Turnover Frequencies, and Overpotential in Molecular Catalysis of Electrochemical Reactions. Cyclic Voltammetry and Preparative-Scale Electrolysis, *J. Am. Chem. Soc.,* 2012, **134**(27), 11235–

11242.
[12] E. S. Rountree, B. D. McCarthy, T. T. Eisenhart, J. L. Dempsey, Evaluation of Homogeneous Electrocatalysts by Cyclic Voltammetry. *Inorg. Chem.*, 2014, **53**(19), 9983-10002.
[13] T. Mashiko, C. A. Reed, K. J. Haller and W. R. Scheidt, Nature of iron(I) and iron(0) tetraphenylporphyrin complexes. Synthesis and molecular structure of (dibenzo-18-crown-6)bis(tetrahydrofuran)sodium (meso-tetraphenylporphinato)ferrate and bis[tris(tetrahydrofuran)sodium](meso-tetraporphinato)ferrate, *Inorg. Chem.*, 1984, **23**(20), 3192-3196.
[14] Y. Zhang, J. Chen, P. E. Siegbahn and R. Z. Liao, Harnessing Non-Innocent Porphyrin Ligand to Circumvent Fe-Hydride Formation in the Selective Fe-Catalyzed CO_2 Reduction in Aqueous Solution, *ACS Catal.,* 2020, **10**(11), 6332-6345.
[15] J. A. Keith, K. A. Grice, C. P. Kubiak, the selectivity of proton- dependent electrocatalytic CO2 reduction by a fac-Re(bpy)(CO)3Cl, *J. Am. Chem. Soc.*, **2013**, 135, 15823-15829.
[16] S. Kozuch and S. Shaik, A Combined Kinetic− Quantum Mechanical Model for Assessment of Catalytic Cycles: Application to Cross-Coupling and Heck Reactions, *J. Am. Chem. Soc.,* 2006, **128**(10), 3355–3365.
[17] J. A. Christiansen, The Elucidation of Reaction Mechanisms by the Method of Intermediates in Quasi-Stationary Concentrations, In *Advances in Catalysis*; Elsevier, 1953; Vol. 5, pp 311–353.
[18] M. Loipersberger, D. Z. Zee, J. A. Panetier, C. J. Chang, J. R. Long and M. Head-Gordon, Computational Study of an Iron (II) Polypyridine Electrocatalyst for CO_2 Reduction: Key Roles for Intramolecular Interactions in CO_2 Binding and Proton Transfer, *Inorg. Chem.,* 2020, **59**(12), 8146-8160.
[19] K. T. Ngo, M. McKinnon, B. Mahanti, R. Narayanan, C. C. Grills, M. Z. Ertem and J. Rochford, Turning on the Protonation-First Pathway for Electrocatalytic CO_2 Reduction by Manganese Bipyridyl Tricarbonyl Complexes, *J. Am. Chem. Soc.,* 2017, **139**(7), 2604–2618.
